# A Modular and Scalable Route to Protected Cyclopropane Amino Acid Building Blocks

**DOI:** 10.1021/acs.orglett.5c01341

**Published:** 2025-04-24

**Authors:** Charlie T. Swan, Alex G. Edmonds, Stephen P. Argent, Nicholas J. Mitchell

**Affiliations:** School of Chemistry, University of Nottingham, University Park, Nottingham NG7 2RD, United Kingdom

## Abstract

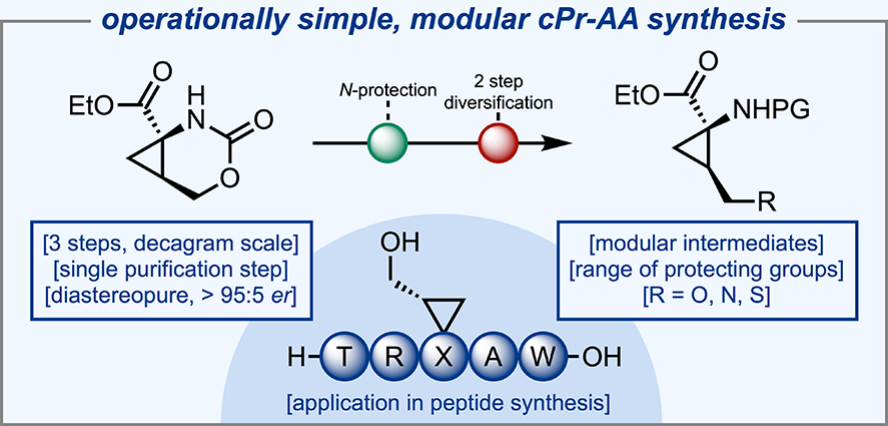

An improved method for the synthesis of noncanonical cyclopropane amino acids from common laboratory reagents is described, avoiding the use of neurotoxic oxidants or precious metal catalysts. Intramolecular isocyanate trapping via a Hofmann rearrangement permits the synthesis of bicyclic carbamates in an enantioenriched and diastereopure manner. Subsequent ring-opening of these species allows access to cyclopropane amino acids which can be further functionalized via oxidation and S_N_2 pathways and incorporated into peptides via solid-phase peptide synthesis.

Noncanonical amino acids (ncAAs) are of increasing interest to medicinal chemists as building blocks for therapeutic peptides.^1^ This is due to their ability to induce conformational changes^2^ and enable the fine-tuning of properties such as stability and permeability, which are traditionally intrinsic drawbacks of classical peptide therapeutics.^1^ Beyond bespoke therapeutics and hormone mimics, integration of ncAAs within antimicrobial peptides (AMPs) may also provide effective tools to combat the growing threat of antimicrobial resistance (AMR), recognized as a global health and socioeconomic crisis.^3−6^

Cyclopropane and its derivatives have been of pharmaceutical interest since the 1930s, with free cyclopropane having been used as a general anesthetic.^7^ It is the most common small ring in pharmaceuticals and agrochemicals and the third most common nonheteroatomic ring system among active pharmaceutical ingredients (APIs).^8^ Its amino acid derivatives have been shown to exhibit substantial levels of bioactivity.^9^ This includes the naturally occurring 1-aminocyclopropane-1-carboxylic acid (ACC, Figure 1), which has been trialed for use as a herbicide,^10^ and coronatine (Figure 1), a toxin produced by the bacterium *Pseudomonas syringae*, containing the coronamic acid fragment.^11^ Additionally, synthetic cyclopropane amino acids have been incorporated into APIs for the treatment of Hepatitis C, such as Grazoprevir (Figure 1) and Simeprevir.^12^

**Figure 1:**
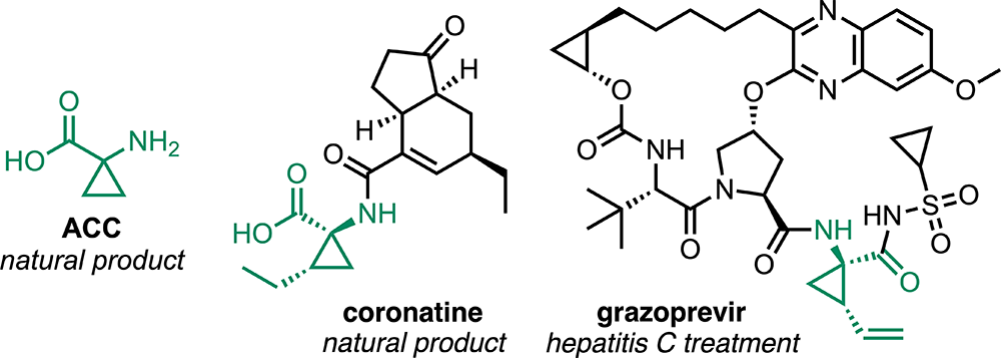
Natural and synthetic structures containing cyclopropane amino acids or derived moieties.

These compounds are of particular interest as the tethered nature of the cyclopropane ring allows for fixed side-chain orientation, which has been shown to increase enzymatic stability as well as receptor selectivity.^9,13−15^ The potential applications of cyclopropane-containing amino acids, in conjunction with the emerging modality of peptide therapeutics, motivated us to investigate operationally simple methods toward these noncanonical residues, while avoiding the use of transition metals and neurotoxic reagents.

Traditional approaches for the synthesis of cyclopropane amino acids can be divided into two general categories:^9^ (1) reactions of C1-equivalents with dehydroamino acids; and (2) formal bisalkylation of malonic acid derivatives or protected amino-esters. The former category can be divided into two subcategories based on the disconnection approach; however, Corey-Chaykovsky reactivity, 1,3-dipolar cycloaddition, or carbene/carbenoid chemistry is generally used.^16^ One disconnection employs an unsubstituted C1-equivalent, paired with a substituted dehydroamino acid or similar,^17^ while the second disconnection pattern requires application of a substituted C1-equivalent and dehydroalanine unit.^14,15,18−22^ However, both methods can suffer from poor diastereo- and enantioselectivities unless transition-metal catalysts and chiral ligands are introduced. This can necessitate the use of supercritical fluid chromatography (SFC) or other diastereomer/enantiomer separation methods.^14,23,24^ The reverse approach using α-diazocarbonyls and olefins has also been implemented.^25−27^

The latter category is more varied in its enantio- and diastereocontrol. Direct alkylation using dihaloalkanes generally has poor diastereocontrol,^9,23^ unless sterically demanding directing groups are introduced. However, utilizing malonates in conjunction with epichlorohydrin as an alkylating agent furnishes a diastereopure bicyclic lactone intermediate (**3**, Scheme 1a)^28−30^ applicable to the synthesis of cyclopropane amino acids. Previous studies have shown that the use of chiral epoxides in this transformation furnishes chiral cyclopropane amino acids.^28−30^

**Scheme 1:**
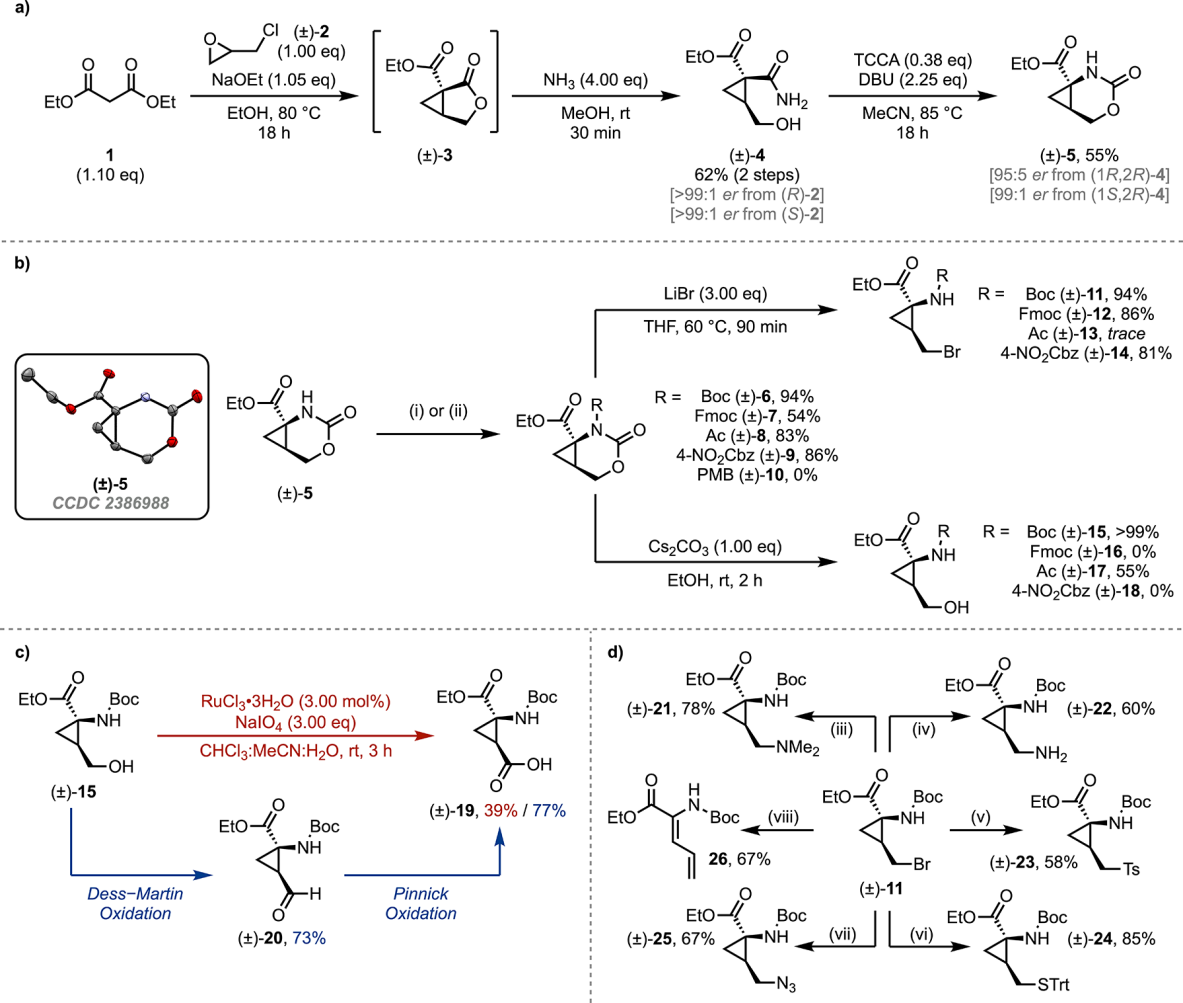
(a) Synthesis of Versatile Cyclic Carbamate **5**; (b) Applications of **5** to the Synthesis of Alkyl Halide and Alcohol Building Blocks; (c) Synthesis of Protected 2,3-Methanoaspartic Acid **19**; (d) Heteroatom Diversification of Alkyl Bromide **11** ORTEP diagram shown in (b) with 50% probability thermal ellipsoids. Reaction conditions: (i) Boc_2_O (2.00 equiv), DMAP (1.00 equiv), DCM, rt, 1 h; (ii) NaH (1.10 equiv), THF, 0 °C, 10 min and then PG_2_O or PG-Cl (2.00 equiv), rt, 2 h; (iii) NHMe_2_ (4.00 equiv), K_2_CO_3_, THF, rt, 18 h; (iv) PhthNK (1.00 equiv), DMF, 70 °C, 2 h and then N_2_H_4_·H_2_O (5.00 equiv), EtOH, 70 °C, 2 h; (v) *p*-TolSO_2_Na (1.20 equiv), DMF, 50 °C, 18 h; (vi) HSC(Ph)_3_ (2.00 equiv), DIPEA (2.00 equiv), DMF, rt, 6 h; (vii) NaN_3_ (1.50 equiv), DMF, 40 °C, 24 h; (viii) Cs_2_CO_3_ (1.50 equiv), DMF, rt, 4 h.

We were initially inspired by the work of Ortuño et al., which identified 2,3-methanohomoserine (**15**) as a suitable intermediate for further functionalization to substituted cyclopropane amino acids.^29^ We utilized a modified version of the route developed by Pirrung and co-workers for access to cyclopropanecarboxamide (**4**). This followed a malonic acid-derived process and was desirable, as the stereochemically pure material could be accessed from either enantiomer of epichlorohydrin (Scheme 1a). An initial objective for this methodology was to reduce the number of chromatographic steps, when compared to previous syntheses.^28,29^ Gratifyingly, it was found that lactone **3** could be used without further purification and amido-ester **4** could be precipitated after the ring-opening in a yield of 62% over 2 steps. This simple purification procedure allowed for the synthesis of **4** on decagram scales (reaction scales of 200 mmol) in a chromatography-free manner.

Attempts to extend the exocyclic chain to pursue glutamic acid analogues, using 2-(2-chloroethyl)oxirane **27** in place of epichlorohydrin, were unsuccessful. In this case, opening of the intermediate oxetane **28** favored the formation of the less strained cyclopentanol **29** over the cyclopropane (Scheme 2). Exposure to acidic conditions failed to afford the bridged lactone analogue.

**Scheme 2:**
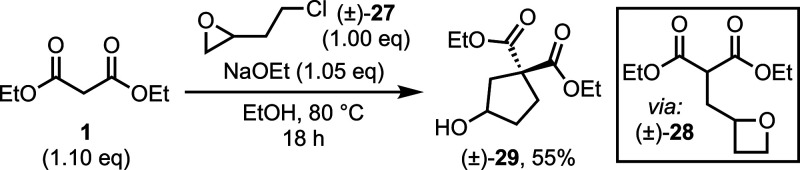
Cyclopentanol Formation from Epichlorohydrin Homologue **27**

We envisaged that subjecting **4** to a Hofmann rearrangement in the absence of *O*-protection would facilitate intramolecular trapping to generate cyclic carbamate **5** (Scheme 1a). Prior work had demonstrated the hydrolysis of *N*-Boc cyclic carbamates of this nature to their corresponding protected amino-alcohols^31^ in the presence of mild base. This suggested that these systems possess a moderate degree of electrophilic character that may permit previously undocumented functionalization.

Acetonitrile was found to be an appropriate non-nucleophilic solvent for this transformation and allowed for a range of oxidants to be trialed. *N*-Bromosuccinimide (NBS) afforded the product in a 40% isolated yield, though it was only applicable to reactions of up to a 5 mmol scale. For larger scales, significant drops in yield were observed (30% isolated on a 50 mmol scale). Rigorous temperature control to negate initial exotherms was unhelpful in increasing the overall yield of the reaction. Phenyliodine bis(trifluoroacetate) (PIFA) was found to be similarly effective for this transformation but was disfavored due to its high molecular weight and consequent generation of large quantities of stoichiometric waste. Trichloroisocyanuric acid (TCCA), in a comparable procedure to Sammakia and co-workers,^32^ was found to be the most effective oxidant, furnishing the desired cyclic carbamate **5** in a 55% yield, which translated well to larger scales (tested up to 75 mmol).

Cyclic carbamate **5** was found to be markedly stable, and decomposition was only observed in the presence of strong acid, while no reaction with heteroatomic nucleophiles was observed. Performing this decomposition in a controlled manner allowed for a deprotection-chlorination strategy to afford alkyl chloride **30** as the hydrochloride salt (Scheme 3). This is analogous to the ring-opening bromination of cyclic carbamates documented by Piper and co-workers.^33^ Milder acids, such as *p*-toluenesulfonic acid, did not facilitate this transformation.

**Scheme 3:**
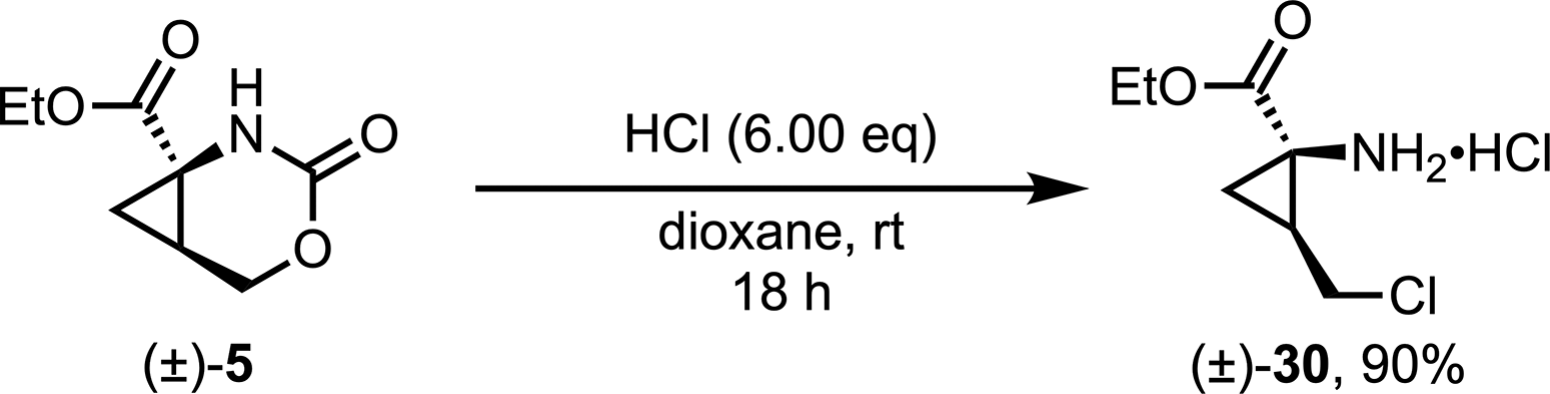
Preparation of Alkyl Chloride Salt **30** via Halogenation of **5** under Acidic Conditions

A variety of protecting groups could be applied to **5**, thus enhancing its reactivity toward ring-opening (Scheme 1b). The *N*-Boc compound **6** could be readily accessed via the reaction with Boc-anhydride and 4-(dimethylamino)pyridine (DMAP). While initial experiments to install exocyclic protecting groups using chloroformates/acid chlorides in the presence of mild base were unsuccessful, it was found that the cyclic carbamate was stable to deprotonation with sodium hydride to form a stable anionic species in solution (dimerization was not observed). The addition of an excess of protecting group electrophile could furnish the desired species in good yields. Even the base-sensitive fluorenylmethoxycarbonyl (Fmoc) protecting group could be applied to this transformation, albeit in a diminished yield. Use of the less electrophilic *para*-methoxybenzyl chloride did not afford any desired product and showed only starting materials by NMR spectroscopic analysis.

A significant breakthrough in the construction of substituted cyclopropane amino acids came in the form of a novel ring-opening bromination, enabled by the reaction of protected cyclic carbamates **6**–**9** with a nucleophilic bromide source. This was applicable to several of the protected carbamates, including the sensitive Fmoc example **7**, with only the less electrophilic acetyl example **8** failing to show appreciable conversion. This bromination was particularly advantageous, as it negates the requirement for any kind of Appel-type process on alcohol **15**, instead providing direct access to the halogenated compound. Reaction with the less nucleophilic lithium chloride returned only starting material, while reaction with sodium iodide under Finkelstein-like conditions afforded ring-opened diene **26** as the major product. This is hypothesized to occur via initial carbamate ring-opening, forming an unstable alkyl iodide which eliminates the labile I^–^ leaving group via a Grob-like process. Extensive attempts to synthesize the fluorinated derivative were unsuccessful via both S_N_2 (**11**) and deoxyfluorination (**15**) approaches. A list of these attempts is summarized in the Supporting Information.

In addition to ring-opening bromination, the aforementioned hydrolysis was applicable.^31^ Besides the *N*-Boc example **6**, it was found that the acetyl protected carbamate **8** could also be opened to its cyclopropylmethyl alcohol analogue **17**. The failure to ring-open **7** under basic conditions was anticipated due to the base sensitivity of the Fmoc functionality; however, comparable sensitivity of the 4-NO_2_Cbz group was not anticipated, with 4-nitrobenzyl alcohol observed as the sole byproduct.

Compounds **11** and **15** are of particular interest for further functionalization. Using an analogous method to Wick and co-workers,^34^ alcohol **15** could be converted to the 2,3-methanoaspartic acid derivative **19** (Scheme 1c). The strongly oxidizing yet functionally tolerant conditions of RuCl_3_/NaIO_4_ were required due to the presence of both base- and acid-sensitive functionalities in the starting alcohol. An alternative and more effective procedure was to initially oxidize alcohol **15** to aldehyde **20** using Dess–Martin periodinane, followed by a successive oxidation to carboxylic acid **19** under Pinnick conditions.

Bromide **11** was of particular interest due to the functionalization opportunities presented by S_N_2 chemistry (Scheme 1d), thus allowing for a single, accessible building block to afford several amino acid analogues. While **11** was found to be unstable in the presence of base, affording the Grob fragmentation product **26**, mildly basic and base-free conditions were well tolerated. This permitted the installation of a variety of amine- and sulfur-derived functionalities, including the formation of the fully protected homocysteine analogue **24**. Unsuccessful transformations for both **11** and **15** are documented in the Supporting Information.

The methods outlined in Scheme 1 were applied to bicyclic phenyl lactone **31** as a potential route to an unreported cyclopropane analogue of β-phenylalanine (Scheme 4). Ring-opening with ammonia was slower due to the deactivating nature of the phenyl substituent; hence, a longer reaction time and greater excess of ammonia was required to access primary amide **32**. The Hofmann rearrangement was particularly effective on this substrate, and the *N*-Boc cyclic carbamate **34** was opened in a yield comparable to that of **6**. Unfortunately, access to the β-amino acid was prohibited by incompatibility with many oxidation conditions (DMP, Bobbitt’s salt, and Swern, as well as Mn and Cr based oxidants), with ring-opening to the conjugated diene observed in many cases. In spite of the inability to oxidize **35** to the acid oxidation state, the β-phenylalaninol analogue remains a compound of interest due to the prevalence of the γ-aminoalcohol moiety and its derivatives within APIs for the treatment of anxiety and depressive disorders.^35−38^

**Scheme 4:**
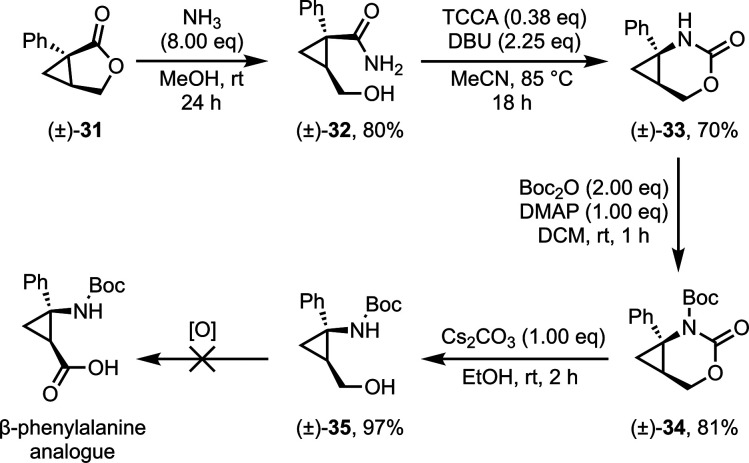
Synthesis of β-Phenylalaninol Analogue **35**

As the Fmoc protecting group is used extensively for solid-phase peptide synthesis (SPPS),^39^ it was desirable to develop a streamlined strategy for the global deprotection and subsequent Fmoc protection of these amino acids to avoid handling the free zwitterionic species. We found that our approach could be tethered to the ring-opening of enantioenriched cyclic carbamate **6**, providing us with a one-pot multi-step procedure to access Fmoc-homoserine analogue **36** in a good yield (average of 86% per step) (Scheme 5). As a proof of concept for the application of these cyclopropane amino acids in SPPS, we synthesized an analogue of the recently reported, functionality-rich, therapeutic peptide Osteostatin,^40^ replacing the serine residue with 2,3-methanohomoserine. The analogue was synthesized in 15% yield from tryptophan-loaded 2-chlorotrityl chloride (CTC) resin. Quantification of deprotection byproducts allowed for the coupling efficiencies of **36** to alanine (A) and arginine (R) to **36** (attached to the peptide) to be determined as quantitative and 68%, respectively,^41^ thus demonstrating outstanding compatibility with universal, automated Fmoc-SPPS methods.

**Scheme 5:**
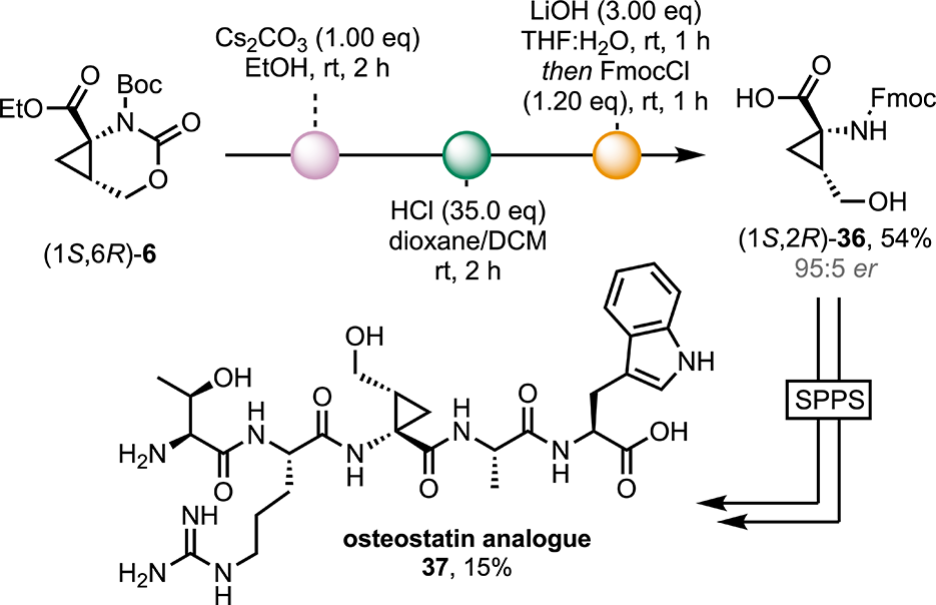
One-Pot, Multi-Step Ring-Opening, Global Deprotection and Fmoc-Installation, and the Subsequent Application of **36** in Solid-Phase Peptide Synthesis

The cyclopropane protons in the Osteostatin analogue could be clearly identified within the ^1^H NMR spectrum, and no elimination products containing the diene or a second diastereomer were observed.

In conclusion, we have developed a convenient and effective strategy to enable access to diversifiable, protected cyclopropane amino acids from common laboratory reagents. This can be achieved both racemically or with excellent stereoretention from enantiopure epichlorohydrin. We have shown the applicability of intramolecular isocyanate trapping in a Hofmann rearrangement to yield cyclic carbamates as versatile building-block intermediates. Once protected, these carbamates can be further functionalized to their ring-opened alcohol or bromide analogues, which themselves can be diversified with heteroatomic nucleophiles. Finally, we have demonstrated a one-pot, multistep process for the conversion of an *N*-Boc cyclic carbamate ethyl ester to an Fmoc-protected cyclopropane analogue of homoserine, which has been successfully incorporated into an analogue of the therapeutic peptide Osteostatin.

## Data Availability

The data underlying this study are available in the published article and its Supporting Information.

## Supporting Information Available

Experimental details, full characterization data, and NMR spectra (PDF)
