# Supplementary material for: A Modular and Scalable Route to Protected Cyclopropane Amino Acid Building Blocks
Source: Org Lett. 2025 Apr 24;27(18):4800–5. doi: 10.1021/acs.orglett.5c01341 (PMC12070460; doi:10.1021/acs.orglett.5c01341)

Supporting Information

A Modular and Scalable Route to Protected Cyclopropane Amino Acid Building Blocks

Charlie T. Swan*, Alex G. Edmonds, Stephen P. Argent and Nicholas J. Mitchell*

School of Chemistry, University of Nottingham, University Park, Nottingham, NG7 2RD, U.K.

Table of Contents

1. *General Information* 1

2. *Synthesis of N-Protected Cyclic Carbamates* 3

(±)-**3** - ethyl (1*SR*,5*RS*) 2-oxo-3-oxabicyclo[3.1.0]hexane-1-carboxylate 3
(±)-**4** - ethyl (1*RS*,2*RS*)-1-carbamoyl-2-(hydroxymethyl)cyclopropane-1-carboxylate 4
(1*R*,2*R*)-**4** - ethyl (1*R*,2*R*)-1-carbamoyl-2-(hydroxymethyl)cyclopropane-1-carboxylate 5
(1*S*,2*S*)-**4** - ethyl (1*S*,2*S*)-1-carbamoyl-2-(hydroxymethyl)cyclopropane-1-carboxylate 6
(±)-**5** - ethyl (1*SR*,6*RS*)-3-oxo-4-oxa-2-azabicyclo[4.1.0]heptane-1-carboxylate 8
(1*S*,6*R*)-**5** - ethyl (1*S*,6*R*)-3-oxo-4-oxa-2-azabicyclo[4.1.0]heptane-1-carboxylate 9
(1*R*,6*S*)-**5** - ethyl (1*R*,6*S*)-3-oxo-4-oxa-2-azabicyclo[4.1.0]heptane-1-carboxylate 10
(±)-**6** - 2-(*tert*-butyl) 1-ethyl (1*SR*,6*RS*)-3-oxo-4-oxa-2-azabicyclo[4.1.0]heptane-1,2-dicarboxylate 11
(±)-**7** - 2-((9*H*-fluoren-9-yl)methyl) 1-ethyl (1*RS*,6*SR*)-3-oxo-4-oxa-2-azabicyclo[4.1.0]heptane-1,2-dicarboxylate 11
(±)-**8** - ethyl (1*RS*,6*SR*)-2-acetyl-3-oxo-4-oxa-2-azabicyclo[4.1.0]heptane-1-carboxylate 12
(±)-**9** - 1-ethyl 2-(4-nitrobenzyl) (1*RS*,6*SR*)-3-oxo-4-oxa-2-azabicyclo[4.1.0]heptane-1,2-dicarboxylate. 13

3. *Ring Opening of Cyclic Carbamates* 14

(±)-**11** - ethyl (1*RS*,2*SR*)-2-(bromomethyl)-1-((*tert*-butoxycarbonyl)amino)cyclopropane-1-carboxylate 14
(±)-**12** - ethyl (1*RS*,2*SR*)-1-((((9*H*-fluoren-9-yl)methoxy)carbonyl)amino)-2-(bromomethyl)cyclopropane-1-carboxylate 14
(±)-**14** - ethyl (1*RS*,2*SR*)-2-(bromomethyl)-1-((((4-nitrobenzyl)oxy)carbonyl)amino)cyclopropane-1-carboxylate 15
(±)-**15** - ethyl (1*SR*,2*RS*)-1-((*tert*-butoxycarbonyl)amino)-2-(hydroxymethyl)cyclopropane-1-carboxylate - *via* hydrolysis 16
(±)-**17** - ethyl (1*RS*,2*SR*)-1-acetamido-2-(hydroxymethyl)cyclopropane-1-carboxylate 16
(±)-**30** - ethyl (1*RS*,2*SR*)-1-amino-2-(chloromethyl)cyclopropane-1-carboxylate hydrochloride 17

4. *Amino Acid Functionalization* 18

(±)-**19** - (1*SR*,2*RS*)-2-((*tert*-butoxycarbonyl)amino)-2-(ethoxycarbonyl)cyclopropane-1-carboxylic acid 18
(±)-**20** - ethyl (1*RS*,2*SR*)-1-((*tert*-butoxycarbonyl)amino)-2-formylcyclopropane-1-carboxylate 19
(±)-**21** - ethyl (1*RS*,2*RS*)-1-((*tert*-butoxycarbonyl)amino)-2-((dimethylamino)methyl)cyclopropane-1-carboxylate 20
(±)-**22** - ethyl (1*RS*,2*RS*)-2-(aminomethyl)-1-((*tert*-butoxycarbonyl)amino)cyclopropane-1-carboxylate. 20
(±)-**23** - ethyl (1*RS*,2*SR*)-1-((*tert*-butoxycarbonyl)amino)-2-(tosylmethyl)cyclopropane-1-carboxylate ... 21
(±)-**24** - ethyl (1*RS*,2*SR*)-1-((*tert*-butoxycarbonyl)amino)-2-((tritylthio)methyl)cyclopropane-1-carboxylate 22
(±)-**25** - ethyl (1*RS*,2*RS*)-2-(azidomethyl)-1-((*tert*-butoxycarbonyl)amino)cyclopropane-1-carboxylate .. 22
26 - ethyl (*Z*)-2-((*tert*-butoxycarbonyl)amino)penta-2,4-dienoate 23
(1*S*,2*R*)-**36** - (1*S*,2*R*)-1-((((9*H*-fluoren-9-yl)methoxy)carbonyl)amino)-2-(hydroxymethyl) cyclopropane-1-carboxylic acid 24

5. *Synthesis of a β-Phenylalaninol Analogue* 26

(±)-**31** - (1*RS*,5*SR*)-1-phenyl-3-oxabicyclo[3.1.0]hexan-2-one 26
(±)-**32** - (1*RS*,2*SR*)-2-(hydroxymethyl)-1-phenylcyclopropane-1-carboxamide 26
(±)-**33** - (1*RS*,6*SR*)-1-phenyl-4-oxa-2-azabicyclo[4.1.0]heptan-3-one 27
(±)-**35** - *tert*-butyl ((1*RS*,2*SR*)-2-(hydroxymethyl)-1-phenylcyclopropyl)carbamate 28

6. *Peptide Synthesis* 30

37 – Osteostatin analogue (H-TRXAW-OH) 31

7. *Miscellaneous Procedures* 34

(±)-**27** - 2-(2-chloroethyl)oxirane 34
(±)-**29** - diethyl 3-hydroxycyclopentane-1,1-dicarboxylate 34
(±)-**SI-1** - ethyl (1*RS*,2*RS*)-2-(((*tert*-butyldimethylsilyl)oxy)methyl)-1-carbamoylcyclopropane-1-carboxylate 35
(±)-**SI-2** - ethyl (1*SR*,2*RS*)-1-((*tert*-butoxycarbonyl)amino)-2-(((*tert*-butyldimethylsilyl)oxy)methyl)cyclopropane-1-carboxylate 36
(±)-**15** - ethyl (1*SR*,2*RS*)-1-((*tert*-butoxycarbonyl)amino)-2-(hydroxymethyl)cyclopropane-1-carboxylate - *via* TBS deprotection of (±)-**SI-2** 37

8. Exploration of Hofmann Rearrangement Conditions ... 38

9. Unsuccessful Transformations ... 39

Unsuccessful Carbamate Transformations ... 39
Unsuccessful Bromide Transformations ... 40
Unsuccessful Alcohol Transformations ... 40
Unsuccessful Aldehyde Transformations ... 40
Miscellaneous Unsuccessful Transformations ... 41
Unsuccessful Fluorination Attempts ... 41

10. X-Ray Diffraction Data Tables ... 43

(±)-**4** - ethyl (1*RS*,2*RS*)-1-carbamoyl-2-(hydroxymethyl)cyclopropane-1-carboxylate ... 43
(±)-**5** - ethyl (1*SR*,6*RS*)-3-oxo-4-oxa-2-azabicyclo[4.1.0]heptane-1-carboxylate ... 44
(±)-**6** - 2-(*tert*-butyl) 1-ethyl (1*SR*,6*RS*)-3-oxo-4-oxa-2-azabicyclo[4.1.0]heptane-1,2-dicarboxylate ... 45
(±)-**11** - ethyl (1*RS*,2*SR*)-2-(bromomethyl)-1-((*tert*-butoxycarbonyl)amino)cyclopropane-1-carboxylate 46
(±)-**19** - (1*SR*,2*RS*)-2-((*tert*-butoxycarbonyl)amino)-2-(ethoxycarbonyl)cyclopropane-1-carboxylic acid 47
(±)-**33** - (1*RS*,6*SR*)-1-phenyl-4-oxa-2-azabicyclo[4.1.0]heptan-3-one ... 48
(±)-**35** - *tert*-butyl ((1*RS*,2*SR*)-2-(hydroxymethyl)-1-phenylcyclopropyl)carbamate ... 49

11. References ... 50

12. NMR Spectra ... 51

1. General Information

Procedures employing oxygen- and/or moisture-sensitive materials were performed with anhydrous solvents (*vide infra*) using standard Schlenk techniques (atmosphere of anhydrous dinitrogen). Analytical thin-layer chromatography was performed on precoated aluminium-backed plates (Silica Gel 60 F254; Merck) and visualized using a combination of UV light (254 nm) and aqueous basic potassium permanganate or ethanolic phosphomolybdic acid stains. Flash column chromatography was performed using Sigma-Aldrich Supelco 60 Å silica gel (230 - 400 mesh).

NMR spectra were recorded at 25 °C on a Bruker Avance 500 or 400 spectrometer (^{1}H, 500 / 400 MHz, $^{13}C\{^{1}H\}$, 125 / 101 MHz). Chemical shifts are reported in ppm; coupling constants, *J*, are reported in Hz and are uncorrected for digitization. The following abbreviations (and their combinations) are used to label the multiplicities: s (singlet), d (doublet), t (triplet), q (quartet), quint (quintet), m (multiplet), br (broad) and app (apparent). ^{1}H and $^{13}C\{^{1}H\}$ chemical shifts are reported relative to tetramethylsilane, and are referenced to the appropriate residual solvent peaks:

- $CDCl_3$: δ_H = 7.26 ppm, δ_C = 77.16 ppm
- $(CD_3)_2SO$: δ_H = 2.50 ppm, δ_C = 39.52 ppm

Infrared spectra of neat compounds were recorded over the range 4000-800 cm^{-1} using a Bruker Alpha FTIR spectrometer fitted with a Bruker Platinum ATR Quicksnap™ diamond cell.

Melting points were measured using a Gallenkamp MF-370 melting point apparatus, with mercury thermometer, in open capillaries.

High resolution electrospray ionization mass spectra (HRMS) were recorded using a Bruker ESI-TOF MicroTOF II spectrometer.

Polarimetric measurements were measured and calculated using an ADP440+ polarimeter.

Analytical HPLC was performed on a Thermo Ultimate 3000 mHPLC system equipped with PDA eλ detector (λ = 210 – 400 nm). Peptides were analyzed using a Waters Sunfire 5 µm, 2.1 x 150 mm column (C-18) at a flow rate of 0.6 mL/min. The mobile phase was composed of 0.1% trifluoroacetic acid in water (Solvent A) and 0.1% trifluoroacetic acid in acetonitrile (Solvent B). Analysis of the chromatograms was conducted using Chromeleon 7 software. Preparative reverse-phase HPLC was performed using a Waters 1525 binary pump HPLC equipped with a dual wavelength UV detector set to 210 nm and 280 nm. Peptides were purified on a Waters Sunfire 5 µm, 19 x 150 mm (C-18)

preparative column operating at a flow rate of 6 mL/min using a mobile phase of 0.1% trifluoroacetic acid in water (Solvent A) and 0.1% trifluoroacetic acid in acetonitrile (Solvent B) using the gradient specified.
Chiral HPLC analysis was carried out using an Agilent 1260 Infinity II LC system with Phenomenex chiral columns. Chiral SFC analysis was carried out using an Agilent 1290 Infinity Chiral SFC system.

Automated Fmoc-SPPS was carried out on a Biotage Initiator$^+$ Alstra microwave peptide synthesizer. Standardized amino acid couplings were performed for 15 min at 50 °C under microwave irradiation in the presence of amino acid (0.5 M in DMF, 4 eq.), Oxyma Pure (0.5 M in DMF, 4 eq.) and diisopropylcarbodiimide (0.5 M in DMF, 4 eq.).

X-ray measurements were made on crystals coated in Fomblin vacuum grease and mounted on a glass needle using Cu-Kα (λ = 1.54184 Å) radiation on SuperNova Atlas or XtalLAB PRO MM007 PILATUS3 R 200K diffractometers at 120(2) K; with the exception of **4** which was collected at 220(2) K. Structures were solved with Olex2 software and refined with ShelXL3 software using Least Squares minimisation.

Reagent grade solvents (Fisher Technical or Sigma Aldrich) were employed. THF was dried using an Inert PureSolv Grubbs-type system (alumina columns, argon atmosphere). Anhydrous CH_2Cl_2, *t*-BuOH and MeCN were purchased from Sigma Aldrich. Absolute EtOH was dried over 3 Å molecular sieves for 48 h prior to use. Solvents were degassed *via* sparging with nitrogen for 30 min. Unless stated otherwise, all reagents were used as received from commercial sources. (+)- and (−)-epichlorohydrin were purchased from Fluorochem.

2. Synthesis of *N*-Protected Cyclic Carbamates

(±)-**3** - ethyl (1*SR*,5*RS*) 2-oxo-3-oxabicyclo[3.1.0]hexane-1-carboxylate

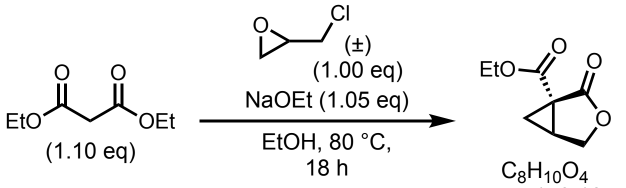

$C_8H_{10}O_4$
mw. 170.16
(±)-**3**, 46%

A flame-dried Schlenk RBF, sealed with Suba-Seal septum, was evacuated and backfilled thrice with anhydrous dinitrogen. Anhydrous ethanol (100 mL) was added and the flask was cooled to 0 °C. The septum was removed and sodium hydride (2.10 g, 52.5 mmol, 60% dispersion in mineral oil) was added in three portions over 15 min and allowed to stir until dissolved (approx. 5 min). To the sodium ethoxide solution was added diethyl malonate (8.35 mL, 55.0 mmol) in one portion. The flask was warmed to room temperature and allowed to stir for 10 min. (±)-Epichlorohydrin (3.91 mL, 50.0 mmol) was added dropwise (approx. 13 min) and stirred for 15 min at room temperature, before the reaction vessel was placed into a preheated DrySyn heating block at 80 °C and stirred for 18 h. The reaction was cooled to room temperature, before being cooled to 0 °C, and the white suspension was filtered through a pad of celite and washed through with ethanol (2 × 50 mL). The pale-yellow solution was concentrated *in vacuo*. To the crude oil was added water (50 mL) and DCM (50 mL); the organic layer was separated, and the aqueous layer was extracted with DCM (2 × 50 mL). The combined organics were dried over anhydrous Na_2SO_4, filtered and concentrated *in vacuo*. The crude residue was purified *via* silica gel column chromatography (17 → 50% EtOAc in cyclohexane) to afford the title compound as a colourless oil (3.89 g, 23.1 mmol, 46%).

Characterisation data were consistent with the literature values: ^{1}H NMR and ^{13}C{^{1}H} NMR.[1]

^{1}H NMR (400 MHz, CDCl$_3$): δ 4.36 (dd, *J* = 9.4, 4.8 Hz, 1H), 4.26 (dq, *J* = 7.2, 2.3 Hz, 2H), 4.18 (d, *J* = 9.4 Hz, 1H), 2.77–2.68 (m, 1H), 2.07 (dd, *J* = 8.2, 4.8 Hz, 1H), 1.37 (app. t, *J* = 5.1 Hz, 1H), 1.31 (t, *J* = 7.2 Hz, 3H).

^{13}C{^{1}H} NMR (101 MHz, CDCl$_3$): δ 170.6, 166.9, 67.1, 62.2, 29.5, 28.1, 20.9, 14.2.

ṽ (ATR)/cm^{-1}: 2983, 1774, 1719, 1468, 1446, 1402, 1383, 1310, 1267, 1186, 1114, 1085, 1041, 1013, 993, 947, 912, 847, 800.

HRMS: (ESI$^+$, *m/z*) Calcd. for $C_8H_{11}O_4^+$ ([M+H]$^+$) 171.0652. Found:171.0659.

(±)-**4** - ethyl (1*RS*,2*RS*)-1-carbamoyl-2-(hydroxymethyl)cyclopropane-1-carboxylate

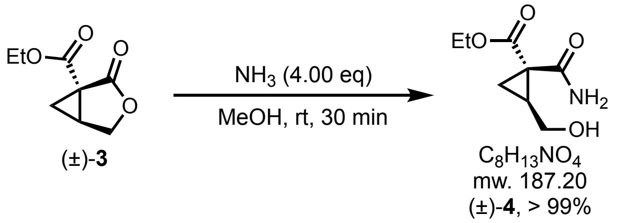

To (±)-**3** (2.02 g, 12.0 mmol) was added ammonia solution (6.86 mL, 48.0 mmol, 7 N in methanol). The reaction was stirred at room temperature for 30 min. Concentration *in vacuo* afforded the title compound as a colourless oil, which crystallised upon standing and was used without further purification (2.25 g, 12.0 mmol, > 99%).

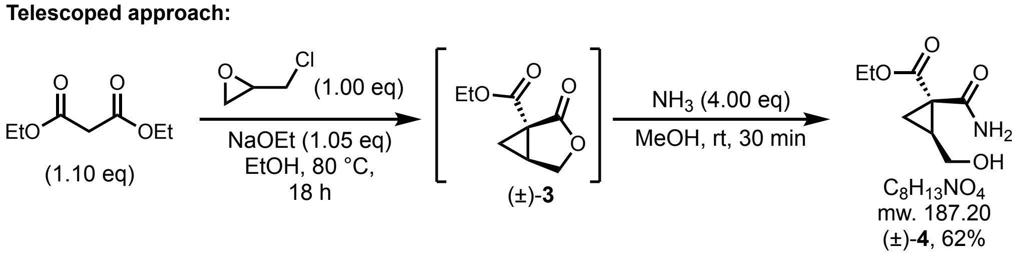

A flame-dried round bottom flask with T connection adapter was sealed with Suba-Seal septum and evacuated and backfilled thrice with anhydrous dinitrogen. Anhydrous ethanol (400 mL) was added, and the flask was cooled to 0 °C. The septum was removed and sodium* (4.83 g, 210 mmol) was added in five portions over 3 h and allowed to stir until dissolved (approx. 1 h). To the sodium ethoxide solution was added diethyl malonate (33.4 mL, 220 mmol) in one portion. The flask was warmed to room temperature and allowed to stir for 10 min. (±)-Epichlorohydrin (15.6 mL, 210 mmol) was added dropwise (approx. 30 min) and stirred for 15 min at room temperature, before the reaction vessel was placed into a preheated DrySyn heating block at 80 °C and stirred for 18 h. The reaction was cooled to room temperature, before being cooled to 0 °C, and the white suspension was filtered through a pad of celite and washed through with ethanol (2 × 200 mL). The pale-yellow solution was concentrated *in vacuo*. To the crude oil was added water (200 mL) and DCM (200 mL); the organic layer was separated, and the aqueous layer was extracted with DCM (2 × 200 mL). The combined organics were dried over anhydrous Na_2SO_4, filtered and concentrated *in vacuo*. To the crude residue was added ammonia solution (114 mL, 798 mmol; 7 N in methanol). The reaction was stirred for 30 min at room temperature, then concentrated *in vacuo*. To the crude oil was added MTBE† (200 mL) and the mixture was stirred at 0 °C for 15 min. Filtration of the precipitated solid, and washing of the filtrate with MTBE (250 mL) afforded the title compound as a colourless solid (23.2 g, 124 mmol, 62% over 2 steps).

* [WARNING]: Sodium metal is highly reactive and a leading cause of laboratory fires. Always handle under inert hydrocarbon solvent and cut with dry tools. Keep away from sources of ignition and flammable materials. Avoid contact with water.

† MTBE may be substituted for Et_2O.

X-ray quality crystals were grown *via* recrystallisation from hot CPME; forming as colourless microcrystals.

m.p./°C: 82–84.

^{1}H NMR (400 MHz, CDCl$_3$): δ 8.32 (br s, 1H), 5.77 (br s, 1H), 4.16 (q, *J* = 7.1 Hz, 2H), 4.01–3.91 (m, 1H), 3.83–3.69 (m, 1H), 2.96 (dd, *J* = 7.6, 5.4 Hz, 1H), 2.33–2.21 (m, 1H), 1.93 (dd, *J* = 8.1, 4.4 Hz, 1H), 1.83 (dd, *J* = 9.7, 4.4 Hz, 1H), 1.26 (t, *J* = 7.2 Hz, 3H).

^{13}C{^{1}H} NMR (101 MHz, CDCl$_3$): δ 172.1, 170.7, 61.8, 59.7, 35.6, 32.1, 20.2, 14.1.

ṽ (ATR)/cm^{-1}: 3393, 3251 (br), 2978, 1702, 1655, 1577, 1471, 1407, 1367, 1303, 1242, 1145, 1112, 1062, 1027, 1145, 1112, 1062, 1027, 985, 879, 863, 841, 811.

HRMS: (ESI$^+$, *m/z*) Calcd. for $C_8H_{14}NO_4^+$ ([M+H]$^+$) 188.0917. Found: 188.0916.

(1*R*,2*R*)-**4** - ethyl (1*R*,2*R*)-1-carbamoyl-2-(hydroxymethyl)cyclopropane-1-carboxylate

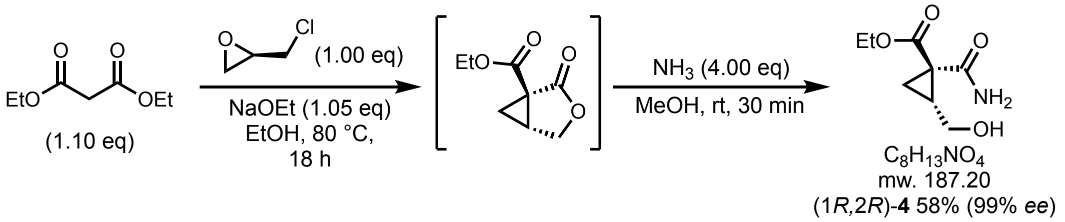

A flame-dried Schlenk RBF was sealed with Suba-Seal septum and evacuated and backfilled thrice with anhydrous dinitrogen. Anhydrous ethanol (80 mL) was added, and the flask was cooled to 0 °C. The septum was removed and sodium (970 mg, 42.0 mmol) was added in five portions over 2 h and allowed to stir until dissolved (approx. 30 min). To the sodium ethoxide solution was added diethyl malonate (6.68 mL, 44.0 mmol) in one portion. The flask was warmed to room temperature and allowed to stir for 10 min. (*R*)-(−)-epichlorohydrin (3.13 mL, 40.0 mmol) was added dropwise (approx. 5 min) and stirred for 15 min at room temperature, before the reaction vessel was placed into a preheated DrySyn heating block at 80 °C and stirred for 18 h. The reaction was cooled to room temperature, before being cooled to 0 °C, and the white suspension was filtered through a pad of celite and washed through with ethanol (2 × 40 mL). The pale-yellow solution was concentrated *in vacuo*. To the crude oil was added water (40 mL) and DCM (40 mL); the organic layer was separated, and the aqueous layer was extracted with DCM (2 × 40 mL). The combined organics were dried over anhydrous Na_2SO_4, filtered and concentrated *in vacuo*. To the crude residue was added ammonia solution (22.9 mL, 80.0 mmol; 7 N in methanol). The reaction was stirred for 30 min at room temperature and concentrated *in vacuo*. To the crude oil was added Et_2O (40 mL) and the mixture was stirred at 0 °C for 15 min (agitation with a spatula may be required). Filtration of the precipitated solid and washing of the filtrate with Et_2O (40 mL) afforded the title compound as a colourless solid (4.33 g, 23.2 mmol, 58% over 2 steps, 99% *ee*).

Characterisation data were consistent with (±)-4.

$[\alpha]_D^{21}$ = −26.7 (c = 0.31, $CHCl_3$).

(1*S*,2*S*)-**4** - ethyl (1*S*,2*S*)-1-carbamoyl-2-(hydroxymethyl)cyclopropane-1-carboxylate

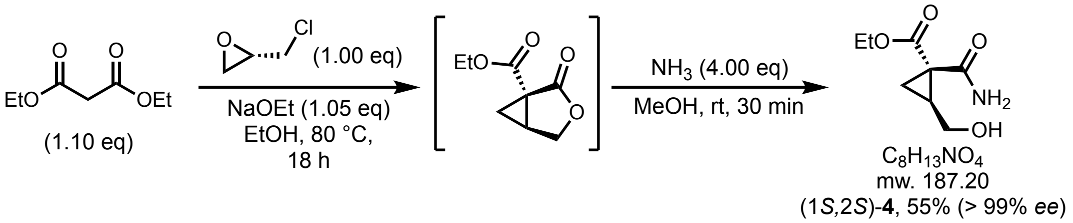

A flame-dried Schlenk RBF was sealed with Suba-Seal septum and evacuated and backfilled thrice with anhydrous dinitrogen. Anhydrous ethanol (20 mL) was added, and the flask was cooled to 0 °C. The septum was removed and sodium (490 mg, 21.0 mmol) was added in five portions over 2 h and allowed to stir until dissolved (approx. 30 min). To the sodium ethoxide solution was added diethyl malonate (3.34 mL, 22.0 mmol) in one portion. The flask was warmed to room temperature and allowed to stir for 10 min. (*S*)-(+)-epichlorohydrin (1.57 mL, 20.0 mmol) was added dropwise (approx. 3 min) and stirred for 15 min at room temperature, before the reaction vessel was placed into a preheated DrySyn heating block at 80 °C and stirred for 18 h. The reaction was cooled to room temperature, before being cooled to 0 °C, and the white suspension was filtered through a pad of celite and washed through with ethanol (2 × 20 mL). The pale-yellow solution was concentrated *in vacuo*. To the crude oil was added water (20 mL) and DCM (20 mL); the organic layer was separated, and the aqueous layer was extracted with DCM (2 × 20 mL). The combined organics were dried over anhydrous Na_2SO_4, filtered and concentrated *in vacuo*. To the crude residue was added ammonia solution (11.4 mL, 80.0 mmol; 7 N in methanol). The reaction was stirred for 30 min at room temperature and concentrated *in vacuo*. To the crude oil was added Et_2O (40 mL) and the mixture was stirred at 0 °C for 15 min (agitation with a spatula may be required). Filtration of the precipitated solid and washing of the filtrate with Et_2O (40 mL) afforded the title compound as a colourless solid (2.06 g, 11.0 mmol, 55% over 2 steps, > 99% *ee*).
Characterisation data were consistent with (±)-4.
$[\alpha]_D^{22}$ = +27.7 (c = 0.17, $CHCl_3$).

Quantification of *ee* for (±)-4, (1*R*,2*R*)-4 and (1*S*,2*S*)-4

Due to the highly polar nature of **4**, separation could not be achieved using reverse-phase Chiral HPLC, and thus a diversification to a separable intermediate was performed.

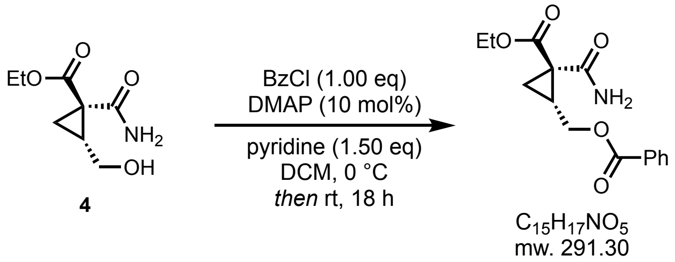

(±)-(1*RS*,2*RS*)-2-carbamoyl-2-(ethoxycarbonyl)cyclopropyl)methyl benzoate

To a stirred solution of (±)-**4** (94 mg, 0.50 mmol), DMAP (6.10 mg, 0.05 mmol), pyridine (64.0 µL, 0.75 mmol) and DCM (1 mL) at 0 °C, was added benzoyl chloride (58 µL, 0.50 mmol). The reaction

was warmed to room temperature and stirred for 18 h. DCM (10 mL) was added and the organic layer was washed with HCl (2 × 10 mL; 1 M aq. solution), $NaHCO_3$ (2 × 10 mL; sat. aq. solution) and brine (10 mL). The organic layer was dried over Na_2SO_4, filtered and concentrated *in vacuo*. The crude residue was purified *via* silica gel column chromatography (50% EtOAc in cyclohexane) to afford the title compound as a colourless solid (97 mg, 0.30 mmol, 59%).

The above procedure was also applied to (1*R*,2*R*)-**4** and (1*S*,2*S*)-**4**.

m.p./°C: 92–95.

^{1}H NMR (400 MHz, CDCl$_3$): δ 8.33 (br s, 1H), 8.08–7.99 (m, 2H), 7.55 (app. t, *J* = 7.4 Hz, 1H), 7.42 (app. t, *J* = 7.6 Hz, 2H), 5.90 (br s, 1H), 4.77 (dd, *J* = 11.8, 5.8 Hz, 1H), 4.35 (dd, *J* = 11.8, 8.9 Hz, 1H), 4.23–4.08 (m, 2H), 2.42–2.30 (m, 1H), 2.02 (dd, *J* = 7.9, 4.3 Hz, 1H), 1.86 (dd, *J* = 9.5, 4.3 Hz, 1H), 1.25 (t, *J* = 7.1 Hz, 3H).

^{13}C{^{1}H} NMR (101 MHz, CDCl$_3$): δ 172.0, 168.6, 166.4, 133.1, 130.2, 129.8, 128.5, 62.4, 61.9, 31.6, 31.3, 20.1, 14.2.

ṽ (ATR)/cm^{-1}: 3422, 3187 (br), 2982, 2915, 1780, 1702, 1661, 1602, 1568, 1491, 1475, 1445, 1412, 1397, 1335, 1315, 1303, 1264, 1150, 1107, 1070, 1030, 1019, 996, 962, 935, 879, 861, 840, 813, 804.

HRMS: (ESI$^+$, *m/z*) Calcd. for $C_{15}H_{18}NO_5^+$ ([M+H]$^+$) 292.1179. Found: 292.1184.

Chiral HPLC: Phenomenex Lux i-Amylose-1 (25 cm); 27.5% MeCN:water (0.1% TFA), 1.0 mL/min, 250 nm.

Racemate (1*RS*,2*RS*) – t_1 = 31.5 min; t_2 = 34.0 min

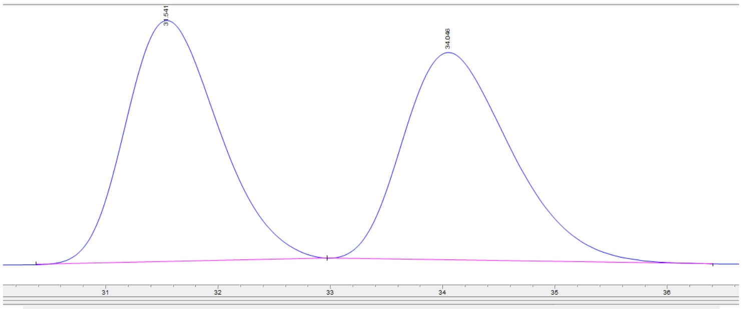

#	Time	Type	Area	Height	Width	Area%	Symmetry
1	31.541	BB	701.1	12.1	0.8608	50.174	0.773
2	34.046	BB	696.2	10.4	0.9514	49.826	0.689

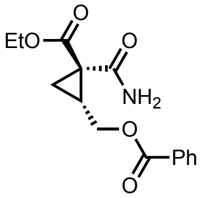

(±) 0% *ee*

(1*R*,2*R*) – t_{min} = 32.5 min; t_{maj} = 34.7 min

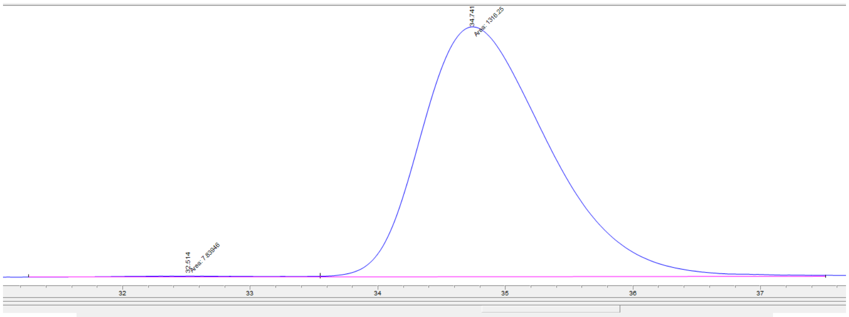

#	Time	Type	Area	Height	Width	Area%	Symmetry
1	32.514	MM	7.8	8.6E-2	1.5111	0.592	0.894
2	34.741	MM	1316.3	19	1.1545	99.408	0.656

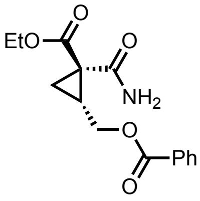

(1*R*,2*R*) 99% *ee*

$[\alpha]_D^{23}$ = +22.7 (c = 0.16, $CHCl_3$).

(1*S*,2*S*) – t_{major} = 31.4 min; t_{minor} = not detected

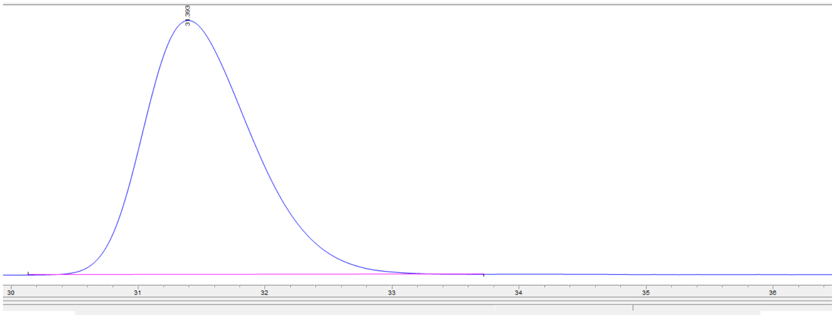

#	Time	Type	Area	Height	Width	Area%	Symmetry
1	31.393	BB	1328.3	22.3	0.9005	100.000	0.671

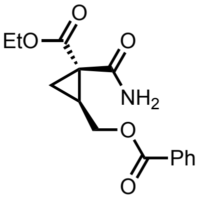

(1*S*,2*S*) > 99% *ee*

$[\alpha]_D^{23}$ = −21.2 (c = 0.15, $CHCl_3$).

(±)-**5** - ethyl (1*SR*,6*RS*)-3-oxo-4-oxa-2-azabicyclo[4.1.0]heptane-1-carboxylate

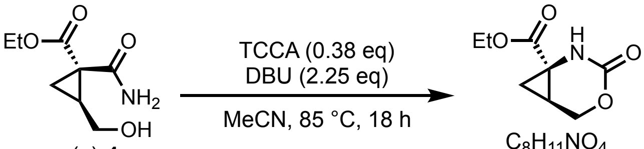

(±)-**4**

$C_8H_{11}NO_4$
mw. 185.18
(±)-**5**, 55%

To (±)-**4** (14.0 g, 75.0 mmol) in MeCN (150 mL) was added DBU (25.2 mL, 169 mmol), and the flask was placed in a room temperature water bath. A first portion of trichloroisocyanuric acid (TCCA; 3.37 g, 14.5 mmol) was added, then the reaction was stirred for 15 min. A second portion of TCCA (3.25 g, 14.0 mmol) was added, and the reaction was stirred for 5 min. The reaction flask was placed in a preheated DrySyn heating block at 85 °C and stirred for 18 h. The reaction mixture was cooled to room temperature, concentrated *in vacuo* and purified *via* silica gel column chromatography (20% EtOAc in DCM) to afford the title compound as a pale brown solid (7.64 g, 41.3 mmol, 55%).

X-ray quality crystals were grown *via* recrystallisation from DCM:cyclohexane (layered); forming as colourless needles.

m.p./°C: 83–85.

^{1}H NMR (400 MHz, CDCl$_3$): δ 6.11 (br s, 1H), 4.60 (dd, J = 11.8, 5.1 Hz, 1H), 4.22 (app. qd, J = 7.2, 1.6 Hz, 2H), 4.17 (dd, J = 11.8, 4.1 Hz, 1H), 2.24–2.13 (m, 1H), 1.69 (dd, J = 9.2, 5.3 Hz, 1H), 1.34–1.28 (m, 1H), 1.28 (t, J = 7.2 Hz, 3H).

^{13}C{^{1}H} NMR (101 MHz, CDCl$_3$): δ 169.9, 153.2, 66.9, 62.4, 38.4, 22.4, 19.4, 14.2.

ṽ (ATR)/cm^{-1}: 3269 (br), 2983, 1708 (br), 1469, 1408, 1371, 1303, 1261, 1173, 1150, 1117, 1078, 1016, 969, 924, 886, 864, 816.

HRMS: (ESI$^+$, *m/z*) Calcd. for $C_8H_{12}NO_4^+$ ([M+H]$^+$) 186.0761. Found: 186.0760.

Chiral HPLC: Phenomenex i-Cellulose-5 (25 cm); 30% MeCN:water (0.1% TFA), 1.0 mL/min, 210 nm. ***t_1 = 5.4 min; t_2 = 6.1 min.***

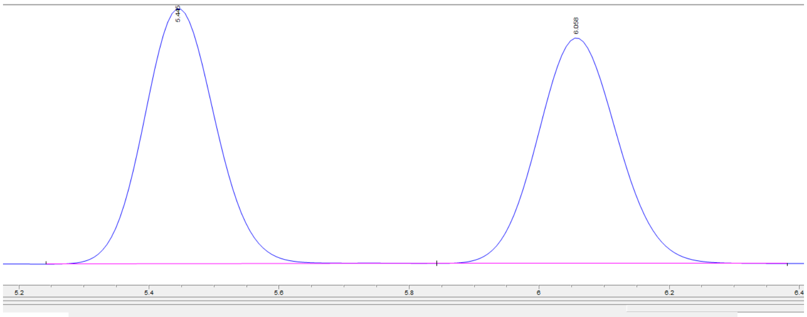

#	Time	Type	Area	Height	Width	Area%	Symmetry
1	5.445	BB	812.7	105.1	0.1222	50.405	0.903
2	6.058	BB	799.7	92.9	0.1345	49.595	0.918

(1*S*,6*R*)-**5** - ethyl (1*S*,6*R*)-3-oxo-4-oxa-2-azabicyclo[4.1.0]heptane-1-carboxylate

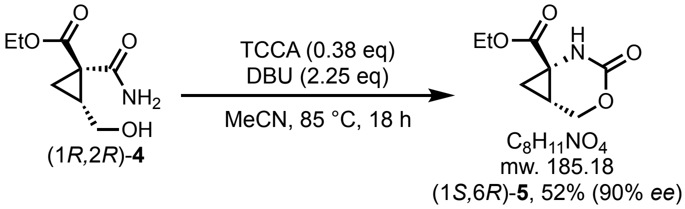

To (1*R*,2*R*)-**4** (3.00 g, 16.0 mmol) in MeCN (32 mL) was added DBU (5.38 mL, 36.0 mmol), and the flask was placed in a room temperature water bath. A first portion of trichloroisocyanuric acid (TCCA; 705 mg, 3.04 mmol) was added, then the reaction was stirred for 15 min. A second portion of TCCA (705 mg, 3.04 mmol) was added, and the reaction was stirred for 5 min. The reaction flask was placed in a preheated DrySyn heating block at 85 °C and stirred for 18 h. The reaction mixture was cooled to room temperature, concentrated *in vacuo* and purified *via* silica gel column chromatography (20% EtOAc in DCM) to afford the title compound as a pale brown solid (1.54 g, 8.32 mmol, 55%, 90% *ee*).

Characterisation data were consistent with (±)-5.

Chiral HPLC: Phenomenex i-Cellulose-5 (25 cm); 30% MeCN:water (0.1% TFA), 1.0 mL/min, 210 nm. ***t_{min} = 5.5 min; t_{maj} = 6.0 min.***

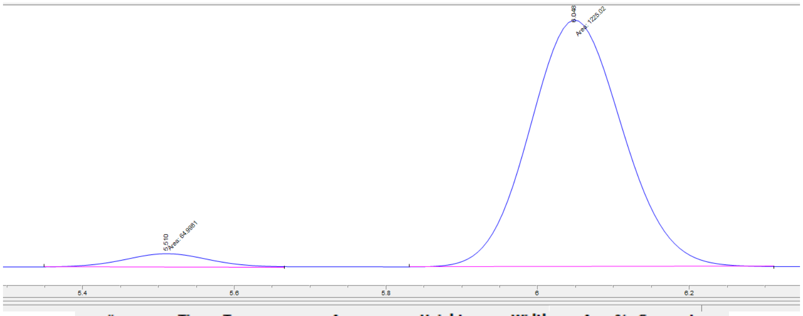

#	Time	Type	Area	Height	Width	Area%	Symmetry
1	5.51	MM	65	8	0.1362	5.039	0.927
2	6.048	MM	1225	143	0.1428	94.961	0.92

$[\alpha]_D^{22}$ = −64.1 (c = 0.24, $CHCl_3$).

(1*R*,6*S*)-**5** - ethyl (1*R*,6*S*)-3-oxo-4-oxa-2-azabicyclo[4.1.0]heptane-1-carboxylate

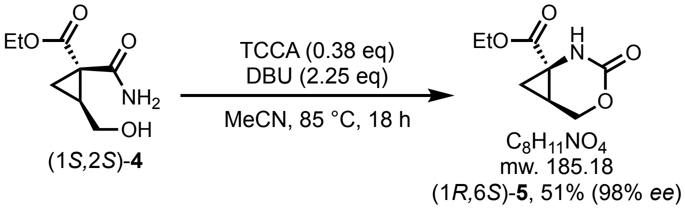

To (1*S*,2*S*)-**4** (936 mg, 5.00 mmol) in MeCN (10 mL) was added DBU (1.68 mL, 11.3 mmol), and the flask was placed in a room temperature water bath. A first portion of trichloroisocyanuric acid (TCCA; 221 mg, 1.91 mmol) was added, then the reaction was stirred for 15 min. A second portion of TCCA (221 mg, 1.91 mmol) was added, and the reaction was stirred for 5 min. The reaction flask was placed in a preheated DrySyn heating block at 85 °C and stirred for 18 h. The reaction mixture was cooled to room temperature, concentrated *in vacuo* and purified *via* silica gel column chromatography (20% EtOAc in DCM) to afford the title compound as a pale brown solid (472 mg, 2.55 mmol, 51%, 98% *ee*).

Characterisation data were consistent with (±)-5.

Chiral HPLC: Phenomenex i-Cellulose-5 (25 cm); 30% MeCN:water (0.1% TFA), 1.0 mL/min, 210 nm. **t_{maj} = 5.4 min; t_{min} = 6.0 min.**

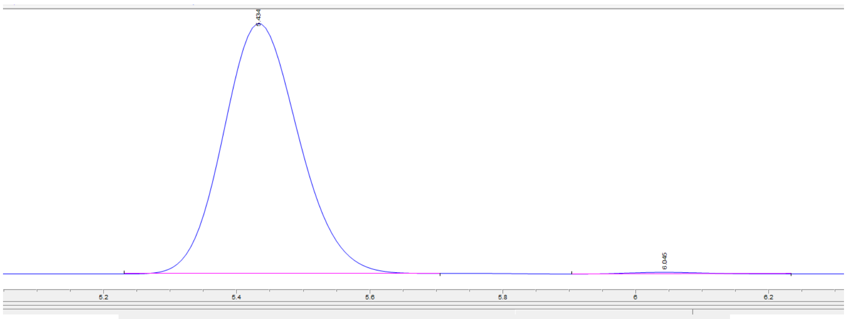

#	Time	Type	Area	Height	Width	Area%	Symmetry
1	5.434	BB	1291.1	162.8	0.1245	99.350	0.862
2	6.045	BB	8.4	1.1	0.1244	0.650	0.911

$[\alpha]_D^{23}$ = +65.3 (c = 0.48, $CHCl_3$).

(±)-**6** - 2-(*tert*-butyl) 1-ethyl (1*SR*,6*RS*)-3-oxo-4-oxa-2-azabicyclo[4.1.0]heptane-1,2-dicarboxylate

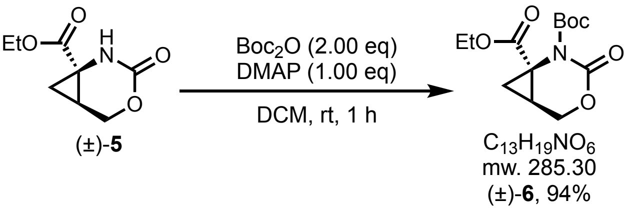

To (±)-**5** (1.85 g, 10.0 mmol) in DCM (67 mL) was added DMAP (1.22 g, 10.0 mmol) and di-*tert*-butyl dicarbonate (4.37 g, 20.0 mmol). The reaction was stirred for 1 h then concentrated *in vacuo*. The crude residue was purified by silica gel column chromatography (20% EtOAc in cyclohexane) to afford the title compound as a colourless solid (2.67 g, 9.36 mmol, 94%).

X-ray quality crystals were grown *via* recrystallisation from hot MTBE; forming as colourless needles.

m.p./°C: 84–86.

^{1}H NMR (400 MHz, $CDCl_3$): δ 4.70 (dd, *J* = 12.1, 8.0 Hz, 1H), 4.25 (app. dq, *J* = 10.7, 7.1 Hz, 1H), 4.15 (dq, *J* = 10.7, 7.1 Hz, 1H), 3.75 (dd, *J* = 12.1, 8.7 Hz, 1H), 2.29 (app. qd, *J* = 8.3, 5.8 Hz, 1H), 2.17 (dd, *J* = 8.3, 5.8 Hz, 1H), 1.51 (s, 9H), 1.32–1.25 (m, 1H), 1.26 (t, *J* = 7.1 Hz, 3H).

^{13}C{^{1}H} NMR (101 MHz, $CDCl_3$): δ 169.7, 150.9, 150.4, 84.1, 69.8, 62.3, 41.6, 28.0, 25.2, 24.9, 14.3.

ṽ (ATR)/cm^{-1}: 3006, 2982, 2932, 1764, 1727, 1469, 1410, 1392, 1369, 1336, 1304, 1287, 1266, 1233, 1184, 1154, 1088, 1059, 1020, 996, 939, 904, 867, 850, 820.

HRMS: (ESI$^+$, *m/z*) Calcd. for $C_{13}H_{19}NNaO_6^+$ ([M+Na]$^+$) 308.1105. Found: 308.1117.

(1*S*,6*R*)-**6** was synthesised *via* the above procedure from (1*S*,6*R*)-**5**, and its characterisation data were consistent with (±)-**6**. $[\alpha]_D^{22}$ = −47.1 (c = 0.50, $CHCl_3$).

(±)-**7** - 2-((9*H*-fluoren-9-yl)methyl) 1-ethyl (1*RS*,6*SR*)-3-oxo-4-oxa-2-azabicyclo[4.1.0]heptane-1,2-dicarboxylate

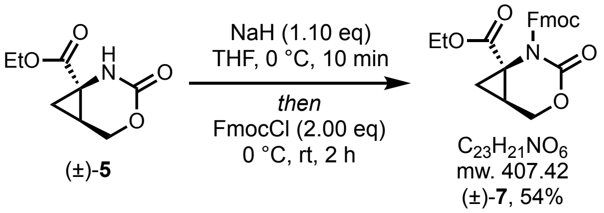

To a flame-dried Schlenk tube was added (±)-**5** (370 mg, 2.00 mmol), the tube was evacuated and backfilled thrice with anhydrous dinitrogen. Anhydrous THF (20 mL) was added, and the reaction was stirred at room temperature until solubilised. The tube was cooled to 0 °C and sodium hydride (88.0 mg, 2.20 mmol; 60% dispersion in mineral oil) was added. The reaction was stirred at 0 °C for 10 min, before FmocCl (1.04 g, 4.00 mmol) was added and the reaction was stirred at room temperature for 2 h. The reaction mixture was quenched with NH_4Cl (15 mL, sat. aq. solution) and extracted with DCM (3 × 15 mL). The combined organics were washed with brine (15 mL), dried over Na_2SO_4, filtered and concentrated *in vacuo*. The crude residue was purified by silica gel column chromatography (35% EtOAc in cyclohexane) affording the title compound as a colourless solid (437 mg, 1.07 mmol, 54%).

m.p./°C: 139–142.

^{1}H NMR (400 MHz, CDCl$_3$): δ 7.80–7.73 (m, 2H), 7.69–7.58 (m, 2H), 7.41 (app. t, *J* = 7.5 Hz, 2H), 7.33 (app. tdd, *J* = 7.5, 2.6, 1.2 Hz, 2H), 4.81 (dd, *J* = 10.7, 5.8 Hz, 1H), 4.68 (app. dd, *J* = 12.2, 8.0 Hz, 1H), 4.54 (dd, *J* = 10.7, 5.8 Hz, 1H), 4.27 (t, *J* = 5.8 Hz, 1H), 4.07–3.90 (m, 2H), 3.66 (app. dd, *J* = 12.2, 8.8 Hz, 1H), 2.30–2.17 (m, 1H), 1.83 (dd, *J* = 8.3, 6.0 Hz, 1H), 1.14 (t, *J* = 7.1 Hz, 3H), 0.96 (app. t, *J* = 6.1 Hz, 1H).

^{13}C{^{1}H} NMR (101 MHz, CDCl$_3$, rotamers present): δ 169.0, 152.5, 150.0, 143.5, 143.4, 141.67, 141.5, 128.0, 127.37, 127.35, 125.0, 124.9, 120.2, 70.0, 68.9, 62.4, 46.9, 41.4, 25.2, 24.8, 14.2.

ṽ (ATR)/cm^{-1}: 3101, 3052, 3018, 2981, 2906, 1823, 1799, 1740, 1716, 1685, 1615, 1467, 1449, 1410, 1397, 1382, 1373, 1344, 1275, 1265, 1186, 1151, 1105, 1081, 1066, 1029, 1007, 988, 964, 938, 916, 863, 846, 813.

HRMS: (ESI$^+$, *m/z*) Calcd. for $C_{23}H_{21}NNaO_6^+$ ([M+Na]$^+$) 430.1261. Found: 430.1267.

(±)-**8** - ethyl (1*RS*,6*SR*)-2-acetyl-3-oxo-4-oxa-2-azabicyclo[4.1.0]heptane-1-carboxylate

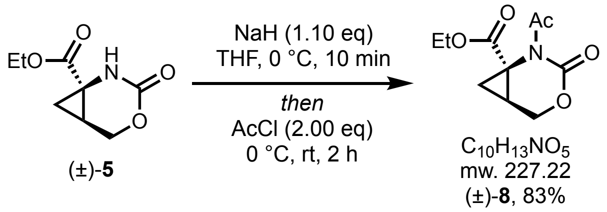

To a flame-dried Schlenk tube was added (±)-**5** (370 mg, 2.00 mmol), the tube was evacuated and backfilled thrice with anhydrous dinitrogen. Anhydrous THF (20 mL) was added, and the reaction was stirred at room temperature until solubilised. The tube was cooled to 0 °C and sodium hydride (88.0 mg, 2.20 mmol; 60% dispersion in mineral oil) was added. The reaction was stirred at 0 °C for 10 min, before AcCl (284 µL, 4.00 mmol) was added and the reaction was stirred at room temperature for 2 h. The reaction mixture was quenched with NH_4Cl (15 mL, sat. aq. solution) and extracted with DCM (3 × 15 mL). The combined organics were washed with brine (15 mL), dried over Na_2SO_4, filtered and concentrated *in vacuo*. The crude residue was purified by silica gel column chromatography (40% EtOAc in cyclohexane) affording the title compound as a colourless oil (377 mg, 1.66 mmol, 83%).

^{1}H NMR (400 MHz, CDCl$_3$): δ 4.82–4.70 (m, 1H), 4.24–4.04 (m, 2H), 3.78–3.67 (m, 1H), 2.47 (s, 3H), 2.30–2.21 (m, 2H), 1.20 (t, *J* = 7.2 Hz, 3H), 1.20–1.17 (m, 1H).

^{13}C{^{1}H} NMR (101 MHz, CDCl$_3$): δ 172.2, 169.0, 152.6, 70.3, 62.3, 40.5, 25.1, 25.0, 23.2, 14.1.

ṽ (ATR)/cm^{-1}: 2984, 1746, 1710, 1548, 1468, 1445, 1407, 1393, 1371, 1328, 1242 , 1179, 1160, 1094, 1059, 1021, 984, 955, 935, 909, 864, 839, 804.

HRMS: (ESI$^+$, *m/z*) Calcd. for $C_{10}H_{13}NNaO_5^+$ ([M+Na]$^+$) 250.0686. Found: 250.0685.

(±)-**9** - 1-ethyl 2-(4-nitrobenzyl) (1*RS*,6*SR*)-3-oxo-4-oxa-2-azabicyclo[4.1.0]heptane-1,2-dicarboxylate

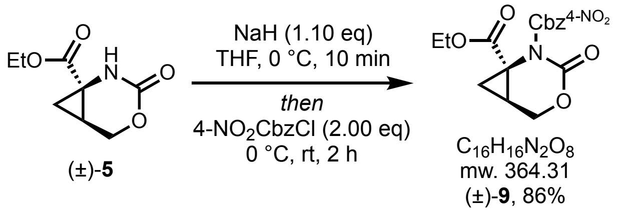

To a flame-dried Schlenk tube was added (±)-**5** (370 mg, 2.00 mmol), the tube was evacuated and backfilled thrice with anhydrous dinitrogen. Anhydrous THF (20 mL) was added, and the reaction was stirred at room temperature until solubilised. The tube was cooled to 0 °C and sodium hydride (88.0 mg, 2.20 mmol; 60% dispersion in mineral oil) was added. The reaction was stirred at 0 °C for 10 min, before 4-nitrobenzyl chloroformate (862 mg, 4.00 mmol) was added and the reaction was stirred at room temperature for 2 h. The reaction mixture was quenched with NH_4Cl (15 mL, sat. aq. solution) and extracted with DCM (3 × 15 mL). The combined organics were washed with brine (15 mL), dried over Na_2SO_4, filtered and concentrated *in vacuo*. The crude residue was purified by silica gel column chromatography (50% EtOAc in cyclohexane) affording the title compound as a colourless solid (628 mg, 1.72 mmol, 86%).

m.p./°C: 154–157.

^{1}H NMR (400 MHz, $CDCl_3$): δ 8.24 (app. d, *J* = 8.8 Hz, 2H), 7.60 (app. d, *J* = 8.8 Hz, 2H), 5.46 (d, *J* = 13.6 Hz, 1H), 5.35 (d, *J* = 13.6 Hz, 1H), 4.77 (dd, *J* = 12.2, 7.9 Hz, 1H), 4.27–4.07 (m, 2H), 3.79 (dd, *J* = 12.2, 8.8 Hz, 1H), 2.44–2.32 (m, 1H), 2.25 (dd, *J* = 8.4, 5.8 Hz, 1H), 1.37 (app. t, *J* = 5.8 Hz, 1H), 1.18 (t, *J* = 7.1 Hz, 3H).

^{13}C{^{1}H} NMR (101 MHz, $CDCl_3$): δ 169.1, 152.7, 149.9, 148.0, 142.3, 128.2, 124.0, 70.2, 67.6, 62.6, 41.8, 25.3, 24.6, 14.2.

ṽ (ATR)/cm^{-1}: 3115, 3084, 2963, 2917, 2850, 1792, 1725, 1708, 1605, 1515, 1475, 1451, 1406, 1393, 1375, 1348, 1337, 1308, 1286, 1263, 1208, 1189, 1157, 1106, 1077, 1056, 1009, 963, 941, 904, 851, 841, 802.

HRMS: (ESI$^+$, *m/z*) Calcd. for $C_{16}H_{17}N_2O_8^+$ ([M+H]$^+$) 365.0979. Found: 365.0982.

3. Ring Opening of Cyclic Carbamates

(±)-**11** - ethyl (1*RS*,2*SR*)-2-(bromomethyl)-1-((*tert*-butoxycarbonyl)amino)cyclopropane-1-carboxylate

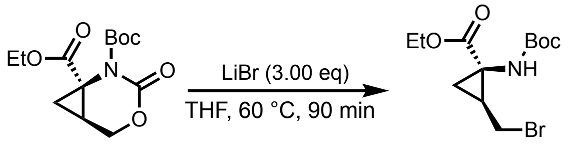

(±)-**6**

$C_{12}H_{20}BrNO_4$
mw. 322.20
(±)-**11**, 94%

To a solution of (±)-**6** (1.43 g, 5.00 mmol) in THF (10 mL) was added lithium bromide (1.30 g, 15.0 mmol). The reaction mixture was heated at 60 °C for 90 min. Concentration *in vacuo* and purification of the crude residue *via* silica gel column chromatography‡ (15% EtOAc in cyclohexane) afforded the title compound as a colourless oil (1.51 g, 4.70 mmol, 94%), which crystallised upon storage at –18 °C. X-ray quality crystals were grown *via* recrystallisation from DCM:cyclohexane (layered); forming as colourless needles.

m.p./°C: 64–68.

^{1}H NMR (400 MHz, CDCl$_3$): δ 5.24 (br s, 1H), 4.15 (app. quint, *J* = 7.0 Hz, 2H), 3.63–3.54 (m, 1H), 3.44–3.35 (m, 1H), 2.22 (app. quint, *J* = 8.1 Hz, 1H), 1.85 (app. s, 1H), 1.45 (s, 9H), 1.25 (t, *J* = 7.1 Hz, 3H), 1.17 (app. s, 1H).

^{13}C{^{1}H} NMR (101 MHz, CDCl$_3$): δ 171.9, 156.5, 80.5, 61.8, 40.6, 32.4, 29.7, 28.4, 24.7, 14.3.

$\tilde{\nu}$ (ATR)/cm^{-1}: 3352, 2977, 2936, 1724, 1687, 1508, 1474, 1443, 1389, 1367, 1336, 1298, 1249, 1189, 1161, 1111, 1079, 1058, 1018, 997, 954, 921, 876, 858, 819.

HRMS: (ESI$^+$, *m/z*) Calcd. for $C_{12}H_{20}{}^{79}BrNNaO_4^+$ ([M+Na]$^+$) 344.0468. Found: 344.0459.

(±)-**12** - ethyl (1*RS*,2*SR*)-1-((((9*H*-fluoren-9-yl)methoxy)carbonyl)amino)-2-(bromomethyl)cyclopropane-1-carboxylate

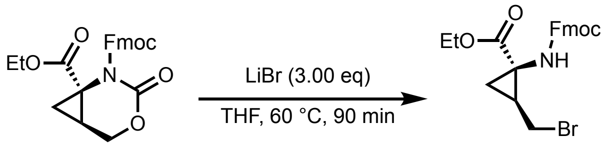

(±)-**7**

$C_{22}H_{22}BrNO_4$
mw. 444.33
(±)-**12**, 86%

To a solution of (±)-**12** (163 mg, 0.40 mmol) in THF (0.8 mL) was added lithium bromide (104 mg, 1.20 mmol). The reaction mixture was heated at 60 °C for 90 min. Concentration *in vacuo* and purification of the crude residue *via* silica gel column chromatography (20% EtOAc in cyclohexane) afforded the title compound as a colourless solid (153 mg, 0.34 mmol, 86%).

‡ Purification could be expedited by dissolving the crude residue in DCM and passing through a small silica plug, then rinsing with DCM, although a 10 – 15% relative decrease in yield was observed over 5-runs.

m.p./°C: 130–133.

^{1}H NMR (400 MHz, CDCl$_3$, rotamers present[§]): δ 7.77 (app. d, *J* = 7.5 Hz, 2H), 7.67–7.49 (m, 2H), 7.41 (app. t, *J* = 7.5 Hz, 2H), 7.32 (app. t, *J* = 7.5 Hz, 2H), 5.50 (br s, 1H), 4.69–4.33 (m, 2H), 4.33–3.76 (m, 3H), 3.52 (app. br s, 1H), 3.36 (app. br s, 1H), 2.28 (app. br s, 1H), 2.09–1.80 (m, 1H), 1.36–1.03 (m, 4H).

^{13}C{^{1}H} NMR (101 MHz, CDCl$_3$, rotamers present): δ 171.5, 157.0, 143.9, 143.8, 141.5, 127.9, 127.2, 125.2 (br), 120.1 (br), 67.2, 62.0, 47.3, 40.7, 32.1, 29.8, 24.6, 14.3.

ṽ (ATR)/cm^{-1}: 3309, 3003, 2960, 2939, 2885, 1724, 1694, 1522, 1479, 1461, 1447, 1397, 1383, 1367, 1331, 1298, 1279, 1254, 1220, 1200, 1185, 1116, 1102, 1091, 1054, 1019, 994, 982, 944, 858, 823.

HRMS: (ESI$^+$, *m/z*) Calcd. for $C_{22}H_{23}{}^{79}BrNO_4{}^+$ ([M+H]$^+$) 444.0805. Found: 444.0796.

(±)-**14** - ethyl (1*RS*,2*SR*)-2-(bromomethyl)-1-((((4-nitrobenzyl)oxy)carbonyl)amino)cyclopropane-1-carboxylate

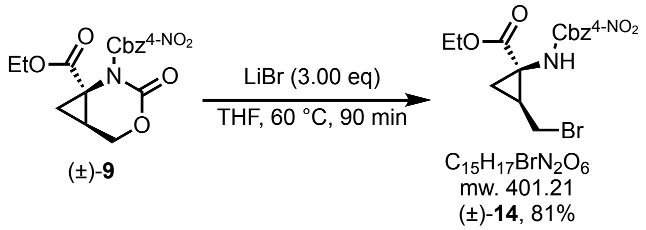

To a solution of (±)-**9** (146 mg, 0.40 mmol) in THF (0.8 mL) was added lithium bromide (104 mg, 1.20 mmol). The reaction mixture was heated at 60 °C for 90 min. Concentration *in vacuo* and purification of the crude residue *via* silica gel column chromatography (25% EtOAc in cyclohexane) afforded the title compound as a colourless oil (131 mg, 0.33 mmol, 81%).

^{1}H NMR (400 MHz, CDCl$_3$): δ 8.20 (app. d, *J* = 8.6 Hz, 2H), 7.52 (app. d, *J* = 8.4 Hz, 2H), 5.65 (br s, 1H), 5.34–5.12 (m, 2H), 4.14 (q, *J* = 7.2 Hz, 2H), 3.66–3.57 (m, 1H), 3.33 (app. t, *J* = 10.0 Hz, 1H), 2.33–2.20 (m, 1H), 1.91 (dd, *J* = 9.4, 5.7 Hz, 1H), 1.29–1.22 (m, 1H), 1.20 (t, *J* = 7.2 Hz, 3H).

^{13}C{^{1}H} NMR (101 MHz, CDCl$_3$): δ 171.3, 156.7, 147.7, 143.6, 128.2, 123.9, 65.7, 62.0, 40.6, 32.0, 29.7, 24.6, 14.2.

ṽ (ATR)/cm^{-1}: 3331, 2981, 1721, 1607, 1520, 1443, 1382, 1368, 1346, 1274, 1232, 1178, 1109, 1081, 1057, 1016, 944, 907, 857, 804.

HRMS: (ESI$^+$, *m/z*) Calcd. for $C_{15}H_{18}{}^{79}BrN_2O_6{}^+$ ([M+H]$^+$) 401.0343. Found: 401.0340.

[§] Large amount of signal broadening and poor resolution observed in ^{1}H NMR.

(±)-**15** - ethyl (1*SR*,2*RS*)-1-((*tert*-butoxycarbonyl)amino)-2-(hydroxymethyl)cyclopropane-1-carboxylate - *via* hydrolysis

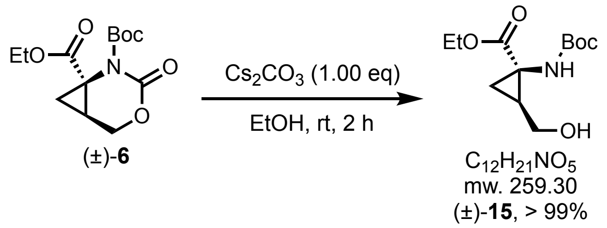

To (±)-**6** (1.71 g, 6.00 mmol) in EtOH (60 mL) was added Cs_2CO_3 (1.96 g, 6.00 mmol). The reaction was stirred for 2 h at room temperature. The reaction was diluted with water (50 mL) and extracted with DCM (3 × 75 mL). The combined organics were washed with brine (50 mL), dried over Na_2SO_4 and filtered. Concentration *in vacuo* afforded the title compound as a colourless oil (1.56 g, 6.00 mmol, > 99%).

Characterisation data were consistent with the literature values: ^{1}H NMR, ^{13}C{^{1}H} NMR and IR.[2]

^{1}H NMR (400 MHz, CDCl$_3$): δ 5.13 (br s, 1H), 4.25–4.06 (m, 2H), 3.97 (ddd, *J* = 12.1, 11.2, 3.4 Hz, 1H), 3.74 (d, *J* = 11.2 Hz, 1H), 3.21 (app. t, *J* = 11.0 Hz, 1H), 2.35–2.18 (m, 1H), 1.54 (dd, *J* = 9.8, 4.9 Hz, 1H), 1.47 (s, 9H) 1.24 (t, *J* = 7.1 Hz, 3H), 0.81–0.74 (m, 1H).

^{13}C{^{1}H} NMR (101 MHz, CDCl$_3$): δ 172.4, 158.4, 81.4, 61.7, 61.6, 38.6, 31.2, 28.3, 19.2, 14.3.

ṽ (ATR)/cm^{-1}: 3350 (br), 2979, 2933, 1724, 1692, 1506, 1457, 1393, 1367, 1326, 1286, 1251, 1158, 1086, 1026, 966, 915, 860, 832.

HRMS: (ESI$^+$, *m/z*) Calcd. for $C_{12}H_{22}NO_5^+$ ([M+H]$^+$) 260.1492. Found: 260.1494.

(±)-**17** - ethyl (1*RS*,2*SR*)-1-acetamido-2-(hydroxymethyl)cyclopropane-1-carboxylate

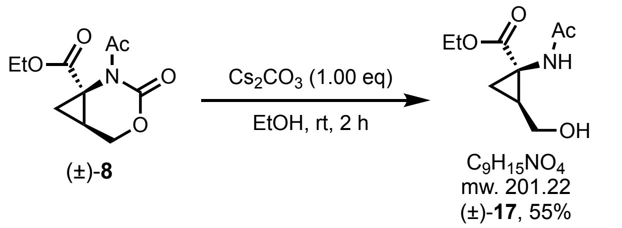

To (±)-**8** (91 mg, 0.40 mmol) in EtOH (4 mL) was added Cs_2CO_3 (130 mg, 0.40 mmol). The reaction was stirred for 2 h at room temperature. The reaction was concentrated to dryness, diluted with water (10 mL) and extracted with DCM (3 × 12 mL). The combined organics were dried over Na_2SO_4 and filtered. Concentration *in vacuo* afforded the title compound as a colourless oil (44 mg, 0.22 mmol, 55%).

^{1}H NMR (400 MHz, CDCl$_3$): δ 6.50 (s, 1H), 4.13 (q, *J* = 7.1 Hz, 2H), 4.00–3.91 (m, 1H), 3.79–3.55 (m, 1H), 3.06 (dd, *J* = 11.9, 10.8 Hz, 1H), 2.35–2.23 (m, 1H), 2.08 (s, 3H), 1.57 (dd, *J* = 9.8, 5.1 Hz, 1H), 1.22 (t, *J* = 7.1 Hz, 3H), 0.81 (dd, *J* = 7.8, 5.1 Hz, 1H).

^{13}C{^{1}H} NMR (101 MHz, CDCl$_3$): δ 174.3, 171.6, 61.8, 61.6, 38.0, 30.7, 23.2, 19.0, 14.2.

ṽ (ATR)/cm^{-1}: 3390 (v br), 2955, 2924, 2853, 1731 (br), 1458, 1376, 1263, 1160, 1095, 1022, 913.

HRMS: (ESI$^+$, *m/z*) Calcd. for $C_9H_{16}NO_4$ ([M+H]$^+$) 202.1074. Found: 202.1067.

(±)-**30** - ethyl (1*RS*,2*SR*)-1-amino-2-(chloromethyl)cyclopropane-1-carboxylate hydrochloride

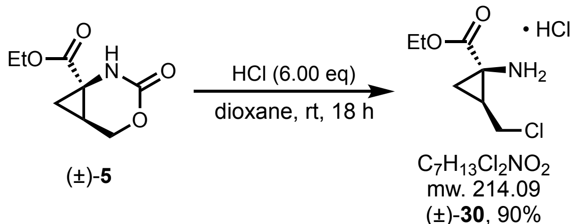

To (±)-**5** (740 mg, 4.00 mmol) was added HCl (6.00 mL, 24.0 mmol; 4 N in dioxane). The reaction mixture was stirred at room temperature for 18 h. Concentration to dryness, dilution with MTBE (15 mL) and filtration, afforded the title compound as a pale orange solid (770 mg, 3.60 mmol, 90%).

m.p./°C: 132–136 (decomp.).

^{1}H NMR (400 MHz, CDCl$_3$): δ 9.34 (s, 3H), 4.29 (app. qd, *J* = 7.1, 5.2 Hz, 2H), 4.15 (dd, *J* = 12.3, 8.5 Hz, 1H), 4.02 (dd, *J* = 12.3, 7.8 Hz, 1H), 2.33–2.20 (m, 1H), 1.85–1.77 (m, 2H), 1.32 (t, *J* = 7.1 Hz, 3H).

^{13}C{^{1}H} NMR (101 MHz, CDCl$_3$): δ 168.1, 63.3, 41.8, 39.5, 28.1, 19.9, 14.2.

ṽ (ATR)/cm^{-1}: 3092, 2981 (v br), 2832 (v br), 1729, 1581, 1556, 1503, 1469, 1443, 1371, 1334, 1273, 1226, 1196, 1140, 1120, 1098, 1066, 1032, 998, 942, 858.

HRMS: (ESI$^+$, *m/z*) Calcd. for $C_7H_{13}{}^{35}ClNO_2^+$ ([M−Cl]$^+$) 178.0629. Found: 178.0646.

4. Amino Acid Functionalization

(±)-**19** - (1*SR*,2*RS*)-2-((*tert*-butoxycarbonyl)amino)-2-(ethoxycarbonyl)cyclopropane-1-carboxylic acid

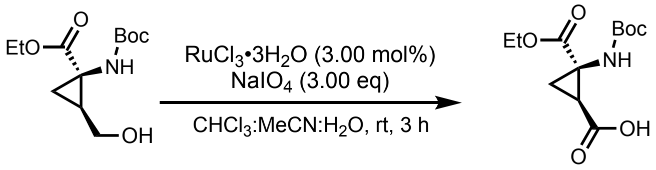

(±)-**15**

$C_{12}H_{19}NO_6$
mw. 273.29
(±)-**19**, 39%

***Via* Oxidation from alcohol:** To a solution of (±)-**15** (246 mg, 0.95 mmol) in $CHCl_3$[**] (1.9 mL), MeCN (1.9 mL) and water (1.4 mL) was added $NaIO_4$ (610 mg, 2.85 mmol). Ruthenium(III) chloride trihydrate (7.45 mg, 0.03 mmol, 3.00 mol%) was added and the reaction mixture was stirred vigorously at room temperature for 3 h. The mixture was diluted with DCM (15 mL) and water (15 mL), the organic layer was separated, and the aqueous layer was extracted with DCM (2 × 15 mL). The combined organics were dried over Na_2SO_4, filtered and concentrated *in vacuo*. The crude black residue was dissolved in Et_2O (20 mL), passed through a celite pad and concentrated. The pale cream residue was dissolved in EtOAc (15 mL) and extracted with $NaHCO_3$ (3 × 15 mL, sat. aq. solution). The basic aqueous layer was carefully acidified to pH 1 with HCl (2 M aq. solution) and extracted with DCM (5 × 15 mL). The combined organics were dried over Na_2SO_4, filtered and concentrated to afford a foamy cream residue, which was recrystallised from DCM:cyclohexane (layered) to afford the title compound as a colourless crystalline solid (103 mg, 0.38 mmol, 39%).

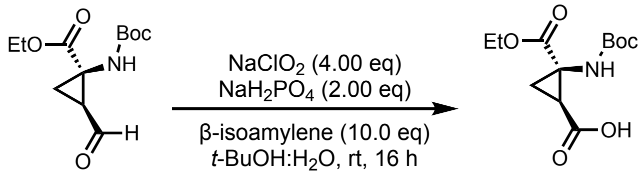

(±)-**20**

$C_{12}H_{19}NO_6$
mw. 273.29
(±)-**19**, 77%

***Via* Oxidation from aldehyde:** A solution of (±)-**20** (257 mg, 1.00 mmol), $NaClO_2$ (453 mg, 4.00 mmol; 80% purity), NaH_2PO_4 (250 mg, 2.00 mmol) and β-isoamylene (1.06 mL, 10.0 mmol) in *t*-BuOH (8 mL) and water (2 mL) was stirred at room temperature for 16 h. The reaction mixture was diluted with brine (30 mL) and extracted with $CHCl_3$ (3 × 15 mL). The combined organics were dried over Na_2SO_4, filtered and concentrated *in vacuo*. The crude residue was purified *via* silica gel column chromatography (55% EtOAc in cyclohexane w/ 0.5% AcOH) afforded the title compound as a fluffy colourless solid (211 mg, 0.77 mmol, 77%).

m.p./°C: 150–153.

[**] $CHCl_3$ contains EtOH as a stabiliser, and thus was washed with 3-times its own volume of water, dried over Na_2SO_4 and filtered. Alternatively, CCl_4 may be used with no deleterious effect.

^{1}H NMR (500 MHz, CDCl$_3$, rotamers observed): δ 11.21 (v br s, 1H), 6.79 (br s, 0.7H), 5.32 (br s, 0.3H), 4.25–4.12 (m, 2H), 2.72–2.56 (m, 0.3H), 2.56–2.38 (m, 0.7H), 1.95–1.89 (m, 1H), 1.79–1.71 (m, 0.3H), 1.62–1.56 (m, 0.7H), 1.43 (s, 9H), 1.27 (t, J = 7.1 Hz, 3H).

^{13}C{^{1}H} NMR (126 MHz, CDCl$_3$): δ 172.4, 170.7, 158.4, 82.1, 62.2, 40.8, 29.1, 28.3, 23.1, 14.4.

ṽ (ATR)/cm^{-1}: 3356, 2987, 2939, 1727, 1693, 1514, 1446, 1430, 1393, 1371, 1338, 1299, 1252, 1228, 1185, 1164, 1088, 1073, 1030, 996, 964, 919, 872, 856, 841, 817.

HRMS: (ESI$^+$, *m/z*) Calcd. for $C_{12}H_{19}NNaO_6^+$ ([M+Na]$^+$) 296.1105. Found: 296.1120.

(±)-**20** - ethyl (1*RS*,2*SR*)-1-((*tert*-butoxycarbonyl)amino)-2-formylcyclopropane-1-carboxylate

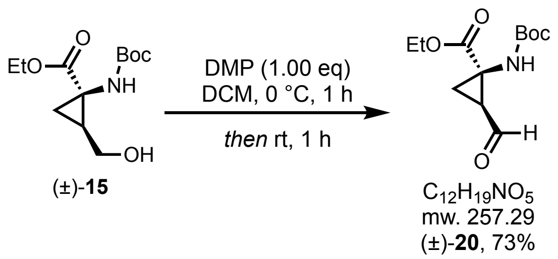

To a suspension of (±)-**15** (778 mg, 3.00 mmol) in DCM (30 mL) at 0 °C was added Dess-Martin periodinane (DMP; 1.27 g, 3.00 mmol), and the reaction was stirred for 1 h. The reaction was then warmed to room temperature and stirred for 1 h. The reaction mixture was quenched with $NaHCO_3$ (20 mL, sat. aq. solution) and $Na_2S_2O_3$ (20 mL, 12% aq. solution). The mixture was extracted with $CHCl_3$ (3 × 30 mL), and the combined organics were dried over Na_2SO_4, filtered and concentrated *in vacuo*. The crude residue was purified by silica gel column chromatography (30% EtOAc in cyclohexane) to afford the title compound as a colourless oil (541 mg, 2.19 mmol, 73%), which crystallised upon storage at –18 °C.

m.p./°C: 77–79.

^{1}H NMR (500 MHz, CDCl$_3$, rotamers observed): δ 9.38 (br s, 0.70H), 9.17 (br s, 0.3H), 5.54 (br s, 0.3H), 5.42 (br s, 0.7H), 4.22–4.11 (m, 2H), 2.83 (s, 0.7H), 2.65 (s, 0.3H), 1.87–1.79 (m, 2H), 1.37 (s, 9H), 1.22 (t, J = 7.1 Hz, 3H).

^{13}C{^{1}H} NMR (126 MHz, CDCl$_3$, rotamers observed): δ 197.1, 195.8, 170.3, 155.8, 155.4, 81.5, 80.7, 62.2, 41.9, 36.4, 35.8, 28.2, 21.1, 20.3, 14.1.

ṽ (ATR)/cm^{-1}: 3346, 3301, 3099, 2980, 2937, 1725, 1691, 1509, 1458, 1391, 1366, 1339, 1285, 1246, 1157, 1083, 1068, 1024, 1010, 993, 949, 919, 869, 853, 835.

HRMS: (ESI$^+$, *m/z*) Calcd. for $C_{12}H_{19}NNaO_5^+$ ([M+Na]$^+$) 280.1155. Found: 280.1152.

(±)-**21** - ethyl (1*RS*,2*RS*)-1-((*tert*-butoxycarbonyl)amino)-2-((dimethylamino)methyl)cyclopropane-1-carboxylate

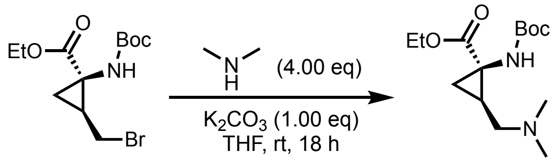

(±)-**11**

$C_{14}H_{26}N_2O_4$
mw. 286.37
(±)-**21**, 78%

To (±)-**11** (161 mg, 0.50 mmol) and K_2CO_3 (69.0 mg, 0.50 mmol) was added dimethylamine (1 mL. 2.00 mmol; 2.00 M in THF). The reaction mixture was stirred at room temperature for 18 h, diluted with water (10 mL) and extracted with DCM (3 × 10 mL). The combined organic layers were extracted with HCl (3 × 15 mL; 2 M aq. solution), and the combined acidic aqueous layers were carefully (managing gas evolution) neutralised with Na_2CO_3. The neutralised aqueous layer was extracted with DCM (3 × 15 mL), dried over Na_2SO_4, filtered and concentrated to afford the title compound as a colourless oil (111 mg, 0.39 mmol, 78%).

^{1}H NMR (400 MHz, CDCl$_3$): δ 5.76 (s, 1H), 4.23–4.06 (m, 2H), 2.47–2.36 (m, 2H), 2.23 (s, 6H), 1.84–1.71 (m, 2H), 1.44 (s, 9H), 1.31–1.19 (t, *J* = 7.2 Hz, 3H), 1.12–0.90 (m, 1H).

^{13}C{^{1}H} NMR (101 MHz, CDCl$_3$): δ 173.1, 156.6, 79.8, 61.4, 59.0, 45.4, 37.9, 28.4, 25.3, 23.5, 14.3.

ṽ (ATR)/cm^{-1}: 3350, 2977, 2819, 2774, 1711 (br), 1467, 1391, 1366, 1333, 1266, 1247, 1160, 1096, 1071, 1023, 970, 915, 848.

HRMS: (ESI$^+$, *m/z*) Calcd. for $C_{14}H_{27}N_2O_4^+$ ([M+H]$^+$) 287.1965. Found: 287.1963.

(±)-**22** - ethyl (1*RS*,2*RS*)-2-(aminomethyl)-1-((*tert*-butoxycarbonyl)amino)cyclopropane-1-carboxylate

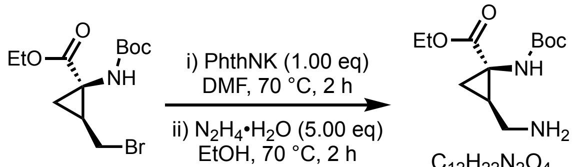

(±)-**11**

$C_{12}H_{22}N_2O_4$
mw. 258.32
(±)-**22**, 60%

A solution of (±)-**11** (161 mg, 0.50 mmol) and potassium phthalimide (93 mg, 0.50 mmol) in DMF (2 mL) was stirred at 70 °C for 2 h. The reaction mixture was diluted with water (8 mL) and extracted with EtOAc (3 × 8 mL). The combined organics were washed with brine (10 mL), dried over Na_2SO_4, filtered and concentrated *in vacuo*. The crude residue was dissolved in EtOH (2.5 mL), hydrazine monohydrate (95 µL, 2.50 mmol) was added, and the reaction mixture was heated to 70 °C for 2 h. The reaction mixture was cooled to room temperature and concentrated *in vacuo*. The resulting residue was dissolved in DCM (8 mL) and was extracted with HCl (3 × 8 mL; 2 M aq. solution). The combined acidic aqueous layers were carefully (managing gas evolution) neutralised with Na_2CO_3. The neutralised aqueous layer was extracted with DCM (3 × 10 mL), dried over Na_2SO_4, filtered and concentrated *in vacuo*. The crude

residue was purified *via* silica gel column chromatography (17.5% MeOH in EtOAc w/ 1% TEA) affording the title compound as a colourless solid (78 mg, 0.30 mmol, 60%).

m.p./°C: 43–46.

^{1}H NMR (400 MHz, CDCl$_3$): δ 5.29 (s, 1H), 4.13 (q, J = 7.1 Hz, 2H), 3.02 (app. dd, J = 13.6, 5.1 Hz, 1H), 2.73–2.40 (m, 1H), 1.99–1.83 (m, 1H), 1.68 (v br s, 2H), 1.57 (dd, J = 9.5, 4.9 Hz, 1H), 1.45 (s, 9H), 1.23 (t, J = 7.1 Hz, 3H), 0.85 (dd, J = 7.6, 4.9 Hz, 1H).

^{13}C{^{1}H} NMR (101 MHz, CDCl$_3$): δ 172.9, 156.9, 80.2, 61.5, 41.6, 38.5, 31.1, 28.4, 21.1, 14.3.

ṽ (ATR)/cm^{-1}: 3365, 3180 (br), 2982, 2935, 2869, 2247, 1718, 1682, 1587, 1535, 1469, 1415, 1391, 1366, 1289, 1251, 1163, 1151, 1079, 1055, 1025, 1005, 969, 949, 923, 904, 875, 861, 824.

HRMS: (ESI$^+$, *m/z*) Calcd. for $C_{12}H_{23}N_2O_4^+$ ([M+H]$^+$) 259.1652. Found: 259.1658.

(±)-**23** - ethyl (1*RS*,2*SR*)-1-((*tert*-butoxycarbonyl)amino)-2-(tosylmethyl)cyclopropane-1-carboxylate

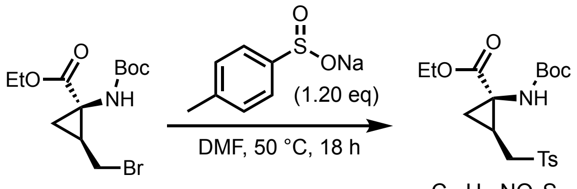

(±)-**11**

$C_{19}H_{27}NO_6S$
mw. 397.49
(±)-**23**, 58%

To (±)-**11** (161 mg, 0.50 mmol) in DMF (2.5 mL) was added sodium *p*-toluenesulfinate (107 mg, 0.60 mmol). The reaction mixture was heated to 50 °C and stirred for 18 h. Water (15 mL) was added, and the mixture was extracted with DCM (3 × 10 mL). The combined organics were washed with water (4 × 10 mL), dried over Na_2SO_4, filtered and concentrated *in vacuo*. The crude residue was purified *via* silica gel column chromatography (0 → 25% EtOAc in cyclohexane) to afford the title compound as a colourless solid (115 mg, 0.29 mmol, 58%). The remainder of the mass balance was largely accounted for by the *O*-alkylated product, which has a slightly higher R_f (0.35 vs 0.22; 25% EtOAc in cyclohexane).

m.p./°C: 127–130.

^{1}H NMR (500 MHz, CDCl$_3$): δ 7.76 (d, J = 8.1 Hz, 2H), 7.36 (d, J = 8.1 Hz, 2H), 5.42 (br s, 1H), 4.20–4.08 (m, 2H), 3.37–3.18 (m, 2H), 2.45 (s, 3H), 1.81–1.67 (m, 2H), 1.43 (s, 9H), 1.24 (t, J = 7.1 Hz, 3H), 1.11–1.07 (m, 1H).

^{13}C{^{1}H} NMR (126 MHz, CDCl$_3$): δ 171.9, 156.6, 145.3, 135.6, 130.2, 128.4, 80.4, 61.9, 55.9, 38.1, 28.4, 22.1, 21.8, 21.3, 14.3.

ṽ (ATR)/cm^{-1}: 3289, 2975, 2923, 1727, 1687, 1597, 1521, 1456, 1393, 1384, 1367, 1316, 1297, 1269, 1249, 1198, 1183, 1164, 1143, 1118, 1084, 1057, 1038, 1017, 1000, 961, 919, 881, 858, 844, 823, 808.

HRMS: (ESI$^+$, *m/z*) Calcd. for $C_{19}H_{27}NNaO_6S^+$ ([M+Na]$^+$) 420.1451. Found: 420.1447.

(±)-**24** - ethyl (1*RS*,2*SR*)-1-((*tert*-butoxycarbonyl)amino)-2-((tritylthio)methyl)cyclopropane-1-carboxylate

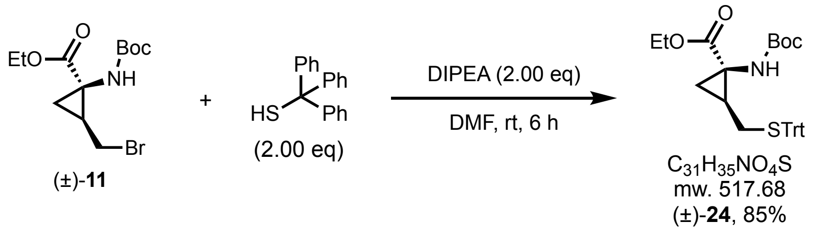

To a solution of (±)-**11** (161 mg, 0.50 mmol) and triphenylmethanethiol (276 mg, 1.00 mmol[††]) in DMF (0.42 mL) was added DIPEA (174 µL, 1.00 mmol), and the reaction mixture was stirred at room temperature for 6 h. The reaction mixture was diluted with brine (10 mL) and extracted with EtOAc (3 × 12 mL). The combined organics were dried over anhydrous Na_2SO_4, filtered and concentrated *in vacuo*. The crude reside was purified *via* column chromatography on silica gel (15% EtOAc in cyclohexane), to afford the title compound as a foamy colourless solid (220 mg, 0.42 mmol, 85%).

m.p./°C: 52–56.

^{1}H NMR (400 MHz, $CDCl_3$, rotamers present): δ 7.41 (app. d, *J* = 7.7 Hz, 6H), 7.29 (app. t, *J* = 7.5 Hz, 6H), 7.22 (app. t, *J* = 7.2 Hz, 3H), 5.14 (s, 0.8H), 4.76 (br s, 0.2H), 4.29–3.97 (m, 2H), 2.53–2.44 (m, 1H), 2.06–1.80 (m, 1H), 1.72–1.59 (m, 2H), 1.45 (s, 5H), 1.43 (s, 4H), 1.22 (t, *J* = 7.1 Hz, 3H), 0.94–0.62 (m, 1H). Boc protons (1.45, 1.43 ppm) are inequivalent due to restricted rotation.

^{13}C{^{1}H} NMR (101 MHz, $CDCl_3$, rotamers present): δ 172.5, 156.5, 144.7, 130.2, 129.6, 128.1, 126.9, 120.5, 115.4, 80.0, 66.8, 61.5, 38.6, 32.1, 28.5, 27.1, 26.1, 23.9, 14.2.

ṽ (ATR)/cm^{-1}: 3361, 3058, 2978, 2931, 1722, 1595, 1487, 1444, 1391, 1367, 1272, 1244, 1159, 1097, 1051, 1032, 1002, 960, 910, 856, 815.

HRMS: (ESI$^+$, *m/z*) Calcd. for $C_{31}H_{35}NNaO_4S^+$ ([M+Na]$^+$) 540.2179. Found: 540.2164.

(±)-**25** - ethyl (1*RS*,2*RS*)-2-(azidomethyl)-1-((*tert*-butoxycarbonyl)amino)cyclopropane-1-carboxylate

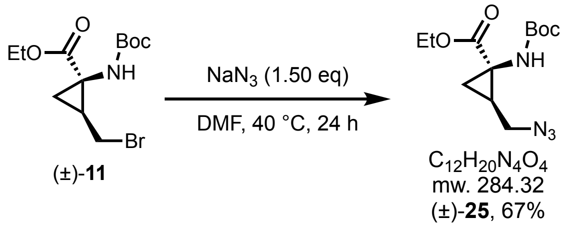

To a solution of (±)-**11** (161 mg, 0.50 mmol, 1.00 eq.) in DMF (1 mL) was added sodium azide[‡‡] (65.0 mg, 0.75 mmol, 1.50 eq.) in a single portion. The reaction mixture was stirred at 40 °C for 24 h, then H_2O (10 mL) was added. The reaction mixture was extracted with diethyl ether (3 × 10 mL), and the

[††] Decreasing the amount of triphenylmethanethiol resulted in the formation of inseparable isomeric products.
[‡‡] [WARNING]: Sodium azide is a precursor to highly explosive metal azides and will react with acid to form highly toxic and explosive hydrazoic acid gas. Appropriate safety precautions should be implemented.[3]

combined organic extracts were dried over anhydrous $MgSO_4$, filtered and concentrated *in vacuo* to afford a yellow oil. The crude material was purified by silica gel column chromatography (20% EtOAc in cyclohexane) affording the title compound as a colourless oil (96 mg, 0.34 mmol, 67%).
Characterisation data were consistent with the literature values: $^{13}C\{^1H\}$ NMR and IR.[2] Some slight differences in chemical shift were observed for the ^1H NMR data.

^{1}H NMR (400 MHz, $CDCl_3$): δ 5.07 (br s, 1H), 4.16 (q, *J* = 7.2 Hz, 2H), 3.49–3.45 (m, 1H), 3.39–3.33 (m, 1H), 2.04 (app. quint, *J* = 7.7 Hz, 1H), 1.77–1.75 (m, 1H), 1.45 (s, 9H), 1.25 (t, *J* = 7.2 Hz, 3H), 1.13–1.09 (m, 1H).

^{13}C NMR (101 MHz, $CDCl_3$): δ 172.2, 156.5, 80.6, 61.8, 50.8, 38.2, 28.3, 26.2, 21.7, 14.2.

ṽ (ATR)/cm^{-1}: 3340, 2979, 2931, 2873, 2093, 1704, 1496, 1455, 1392, 1367, 1328, 1273, 1246, 1162, 1081, 1023, 971, 916, 860.

HRMS: (ESI^+, *m/z*) Calcd. for $C_{12}H_{21}N_4O_4^+$ ($[M+H]^+$) 285.1557. Found: 285.1561.

26 - ethyl (*Z*)-2-((*tert*-butoxycarbonyl)amino)penta-2,4-dienoate

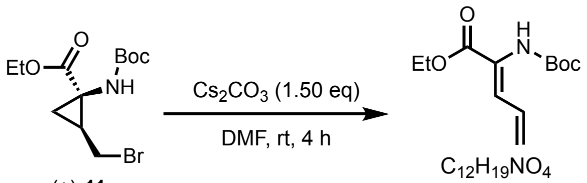

(±)-**11**

$C_{12}H_{19}NO_4$
mw. 241.29
26, 67%

To (±)-**11** (161 mg, 0.50 mmol) in DMF (2.5 mL) was added Cs_2CO_3 (244 mg, 0.75 mmol), and the reaction mixture was stirred at room temperature for 4 h. The reaction mixture was diluted with brine (10 mL) and extracted with DCM (3 × 10 mL). The combined organics were washed with brine (10 mL), dried over anhydrous Na_2SO_4, filtered and concentrated *in vacuo*. The crude reside was purified *via* silica gel column chromatography (10% EtOAc in cyclohexane) to afford the title compound as a colourless oil (81 mg, 0.34 mmol, 67%).

This compound readily degrades at room temperature to a complex mixture, storage at –18°C or lower is advised.

No NoE observed between terminal alkene protons and NHBoc or CO_2Et; however, diene proton data is consistent with the previously reported (*Z*)-methyl ester analogue.[4]

^{1}H NMR (500 MHz, $CDCl_3$): δ 6.91 (d, *J* = 11.3 Hz, 1H), 6.57 (ddd, *J* = 17.0, 11.3, 10.0 Hz, 1H), 6.22 (br s, 1H), 5.56 (app. d, *J* = 17.0 Hz, 1H), 5.44 (app. dd, *J* = 10.0, 1H), 4.25 (q, *J* = 7.1 Hz, 2H), 1.46 (s, 9H), 1.32 (t, *J* = 7.1 Hz, 3H).

$^{13}C\{^1H\}$ NMR (126 MHz, $CDCl_3$): δ 165.3, 153.3, 132.0, 130.4, 124.7, 124.1, 81.0, 61.7, 28.3, 14.4.

ṽ (ATR)/cm^{-1}: 3342, 2988, 2942, 1708, 1643, 1603, 1493, 1396, 1371, 1349, 1248, 1161, 1948, 1932, 817, 857.

HRMS: (ESI$^+$, *m/z*) Calcd. for $C_{12}H_{19}NNaO_4^+$ ([M+Na]$^+$) 264.1206. Found: 264.1200.

(1*S*,2*R*)-**36** - (1*S*,2*R*)-1-((((9*H*-fluoren-9-yl)methoxy)carbonyl)amino)-2-(hydroxymethyl) cyclopropane-1-carboxylic acid

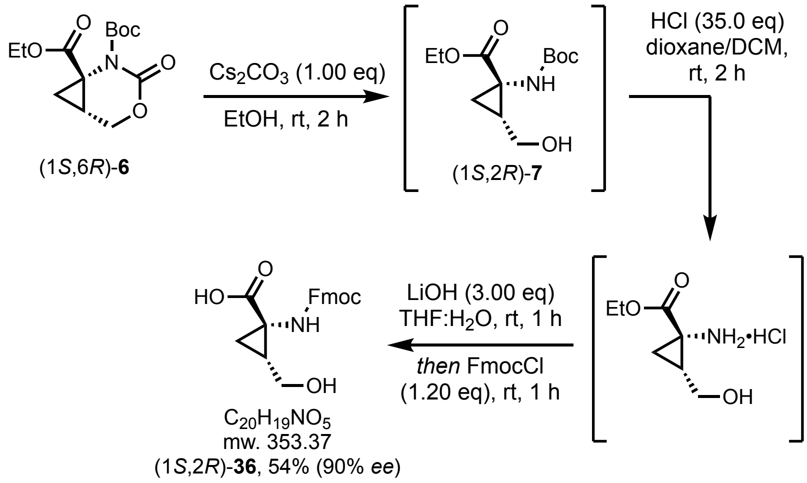

To a solution of (1*S*,6*R*)-**6** (285 mg, 1.00 mmol) in EtOH (10 mL) was added Cs_2CO_3 (326 mg, 1.00 mmol), and the reaction mixture was stirred at room temperature for 2 h. The mixture was concentrated to dryness, diluted with DCM (18 mL). The suspension was cooled to 0 °C and HCl (8.75 mL, 35.0 mmol; 4 M solution in dioxane) was added. The white suspension was stirred at room temperature for 2 h, before being concentrated *in vacuo*. The crude off-white solid was suspended in THF (25 mL) and LiOH (9 mL, 3.00 mmol; 0.33 M aq. solution) was added. The reaction was stirred at room temperature for 1 h, before FmocCl (343 mg, 1.20 mmol) was added in a single portion. The reaction was stirred for 1 h. The reaction mixture was acidified to pH 5 with HCl (1.00 M aq. solution) and extracted with EtOAc (3 × 40 mL). The combined organics were dried over anhydrous Na_2SO_4, filtered and concentrated *in vacuo*. The crude residue was purified *via* silica gel column chromatography (50 → 70% EtOAc in cyclohexane w/ 0.5% AcOH) to afford the title compound as a colourless solid (287 mg, 0.54 mmol, 54%, 90% *ee*).

m.p./°C: 213–217.

^{1}H NMR (400 MHz, DMSO-*d6*, rotamers present): δ 12.47 (s, 1H), 7.89 (app. d, *J* = 7.4 Hz, 2H), 7.77–7.62 (m, 3H), 7.42 (app. t, *J* = 7.4 Hz, 2H), 7.33 (app. t, *J* = 7.2 Hz, 2H), 4.58–4.13 (m, 4H), 3.58–3.30 (m, 2H), 1.87–1.75 (m, 1H), 1.45 (dd, *J* = 9.5, 4.8 Hz, 0.8H), 1.40–1.33 (m, 0.2H), 0.88 (dd, *J* = 7.6, 4.8 Hz, 0.8H), 0.77 (app. br s, 0.2H).

^{13}C{^{1}H} NMR (101 MHz, DMSO-*d6*, rotamers present): δ 174.4, 174.1, 157.2, 156.9, 143.79, 143.75, 140.8, 127.7, 127.1, 125.26, 125.23, 120.14, 120.05, 66.3, 65.6, 60.1, 60.0, 46.7, 37.3, 29.5, 19.8.

ṽ (ATR)/cm^{-1}: 3293, 2947, 2873, 2589, 1702, 1516, 1479, 1432, 1380, 1319, 1284, 1254, 1230, 1181, 1152, 1107, 1058, 1029, 1010, 964, 944, 913, 886, 878, 827.

HRMS: (ESI$^+$, *m/z*) Calcd. for $C_{20}H_{19}NNaO_5^+$ ([M+Na]$^+$) 376.1155. Found: 376.1161.

Chiral SFC[§§]: CHIRALPAK IC (25 cm); 25% MeOH:CO_2, 3.0 mL/min, 254 nm. ***t*$_{maj}$ = 3.8 min; *t*$_{min}$ = 4.2 min.** The racemate was synthesised *via* the analogous procedure from (1*RS*,2*SR*)-**6**.

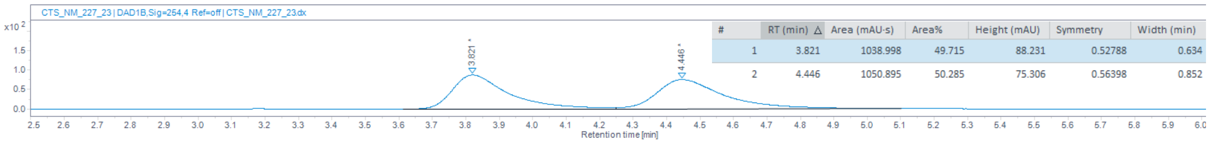

#	RT (min)	Area (mAU·s)	Area%	Height (mAU)	Symmetry	Width (min)
1	3.821	1038.998	49.715	88.231	0.52788	0.634
2	4.446	1050.895	50.285	75.306	0.56398	0.852

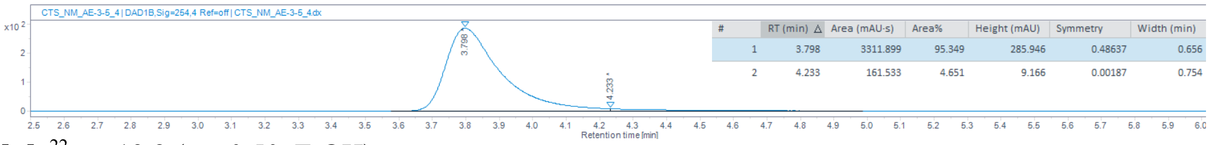

#	RT (min)	Area (mAU·s)	Area%	Height (mAU)	Symmetry	Width (min)
1	3.798	3311.899	95.349	285.946	0.48637	0.656
2	4.233	161.533	4.651	9.166	0.00187	0.754

$[\alpha]_D^{22}$ = −13.2 (c = 0.50, EtOH).

§§ Separation of these enantiomers proved challenging. Mosher ester analysis was unsuccessful. Chiral HPLC (MeCN/H_2O/0.1% TFA) was unsuccessful with Phenomenex i-Cell-5, i-Amy-1, Cell-2, Cell-3 and Cell-4 columns. Chiral SFC (CO_2/MeOH) was unsuccessful with CHIRALPAK IE, CHIRAL ART Amylose-SA and CHIRALCEL OD-H columns. Due to the tailing observed in the peak of the major enantiomer, a time-based integration approach has been used.

5. Synthesis of a β-Phenylalaninol Analogue

(±)-**31** - (1*RS*,5*SR*)-1-phenyl-3-oxabicyclo[3.1.0]hexan-2-one

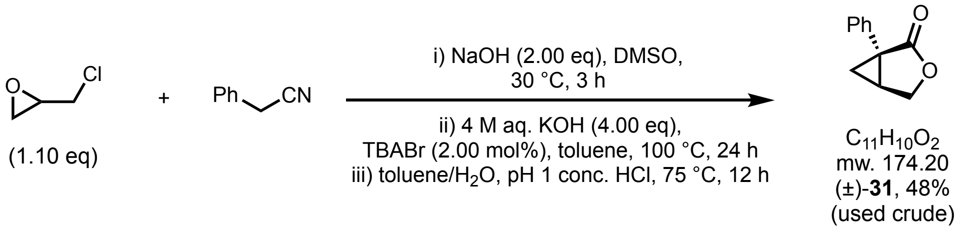

Following an adapted literature procedure.[5] A solution of (±)-epichlorohydrin (4.31 mL, 55.0 mmol), phenylacetonitrile (5.74 mL, 50.0 mmol) and sodium hydroxide (4.00 g, 100 mmol) in DMSO (25 mL) was stirred at 30 °C for 3 h. The reaction mixture was cooled to 0 °C, and toluene (25 mL) and water (50 mL) were added. The organic phase was separated, and the aqueous phase was extracted with toluene (3 × 25 mL). To the combined organic phases was added potassium hydroxide (11.2 g, 200 mmol) and tetra-*n*-butylammonium bromide (TBABr; 322 mg, 1.00 mmol), and the resulting mixture was stirred at 100 °C for 24 h. The reaction mixture was cooled to room temperature and extracted with water (50 mL). To the aqueous extracts was added toluene (50 mL). The mixture was acidified to pH 1 with HCl (conc. aq. solution) and stirred at 75 °C for 12 h. After cooling to room temperature, the organic phase was separated, and the aqueous phase was extracted with toluene (3 × 50 mL). The combined organic phases were washed with $NaHCO_3$ (3 × 50 mL, sat. aq. solution), dried over $MgSO_4$, filtered and concentrated *in vacuo* to afford the title compound as a brown oil (4.14 g, 23.8 mmol, 48%) which was used without further purification.

Characterisation data were consistent with the literature values: ^{1}H NMR, ^{13}C{^{1}H} NMR, IR and HRMS.[6]

^{1}H NMR (400 MHz, $CDCl_3$): δ 7.44–7.41 (m, 2H), 7.35 (app. tt, *J* = 6.4, 1.1 Hz, 2H), 7.32–7.27 (m, 1H), 4.46 (dd, *J* = 9.3, 4.6 Hz, 1H), 4.28 (d, *J* = 9.3 Hz, 1H), 2.56 (app. dt, *J* = 7.8, 4.6 Hz, 1H), 1.64 (dd, *J* = 7.8, 4.7 Hz, 1H), 1.36 (app. t, *J* = 4.7 Hz, 1H).

^{13}C{^{1}H} NMR (101 MHz, $CDCl_3$): δ 176.1, 134.2, 128.7, 128.4, 127.7, 68.2, 31.8, 25.2, 20.2.

$\tilde{\nu}$ (ATR)/cm^{-1}: 3061, 2039, 2972, 1759, 1501, 1363, 1300, 1111, 1081, 1039, 985.

HRMS: (ESI$^+$, *m/z*) Calcd. for $C_{11}H_{11}O_2^+$ ([M+H]$^+$) 175.0754. Found: 175.0761.

(±)-**32** - (1*RS*,2*SR*)-2-(hydroxymethyl)-1-phenylcyclopropane-1-carboxamide

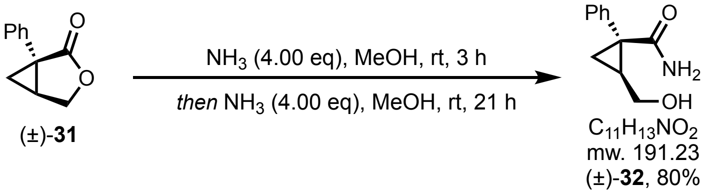

To (±)-**31** (1.74 g, 10.0 mmol) was added ammonia (5.71 mL, 40.0 mmol; 7N in MeOH), and the mixture was stirred at room temperature for 3 h. A second portion of ammonia solution (5.71 mL, 40.0

mmol; 7N in MeOH) was added, and the reaction mixture was stirred for a further 21 h. The reaction mixture was concentrated *in vacuo*, and the resulting residue was washed with Et_2O (3 × 10 mL) to afford the title compound as an amorphous colourless solid (1.53 g, 8.00 mmol, 80%) which was used without further purification.

m.p./°C: 129–132.

^{1}H NMR (400 MHz, $CDCl_3$): δ 7.44–7.41 (m, 2H), 7.36 (app. tt, *J* = 6.4, 1.0 Hz, 2H), 7.33–7.28 (m, 1H), 5.76 (br s, 1H), 5.49 (br s, 1H), 4.10 (dd, *J* = 12.1, 4.1 Hz, 1H), 3.86 (app. t, *J* = 10.4 Hz, 1H), 3.09 (br s, 1H), 1.90–1.83 (m, 1H), 1.78 (dd, *J* = 7.1, 4.5 Hz, 1H), 1.33 (dd, *J* = 9.2, 4.5 Hz, 1H).

^{13}C{^{1}H} NMR (101 MHz, $CDCl_3$): δ 175.8, 140.8, 130.3, 129.2, 128.1, 60.6, 35.3, 31.4, 18.4.

ṽ (ATR)/cm^{-1}: 3468, 3287, 3171, 2854, 1677, 1602, 1387, 1110, 1025, 953.

HRMS: (ESI$^+$, *m/z*) Calcd. for $C_{11}H_{14}NO_2^+$ ([M+H]$^+$) 192.1019. Found: 192.1023.

(±)-**33** - (1*RS*,6*SR*)-1-phenyl-4-oxa-2-azabicyclo[4.1.0]heptan-3-one

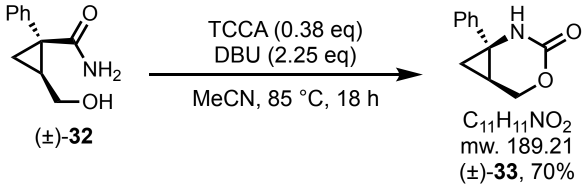

To (±)-32 (1.43 g, 7.50 mmol) in MeCN (15 mL) was added DBU (2.52 mL, 16.9 mmol), and the flask was placed in a room temperature water bath. A first portion of trichloroisocyanuric acid (TCCA; 337 mg, 1.45 mmol) was added, then the reaction was stirred for 15 min. A second portion of TCCA (325 mg, 1.40 mmol) was added, and the reaction was stirred for 5 min. The reaction flask was placed in a preheated DrySyn heating block at 85 °C and stirred for 18 h. The reaction mixture was cooled to room temperature, concentrated *in vacuo* and purified *via* silica gel column chromatography (10% EtOAc in DCM) to afford the title compound as an amorphous off-white solid (994 mg, 5.25 mmol, 70%).
X-ray quality crystals were grown *via* recrystallisation from DCM:cyclohexane (layered); forming as colourless needles.

m.p./°C: 89–91.

^{1}H NMR (400 MHz, $CDCl_3$): δ 7.39–7.35 (m, 2H), 7.33–7.28 (m, 3H), 5.51 (br s, 1H), 4.79–4.74 (m, 1H), 4.25–4.21 (m, 1H), 1.73–1.66 (m, 2H), 1.31–1.25 (m, 1H).

^{13}C{^{1}H} NMR (101 MHz, $CDCl_3$): δ 154.7, 139.7, 129.2, 128.1, 126.4, 68.7, 41.0, 20.4, 19.5.

ṽ (ATR)/cm^{-1}: 3246, 3120, 1704, 1472, 1450, 1412, 1301, 1017.

HRMS: (ESI$^+$, *m/z*) Calcd. for $C_{11}H_{12}NO_2^+$ ([M+H]$^+$) 190.0863. Found: 190.0864.

(±)-**34** - tert-butyl (1*RS*,6*SR*)-3-oxo-1-phenyl-4-oxa-2-azabicyclo[4.1.0]heptane-2-carboxylate

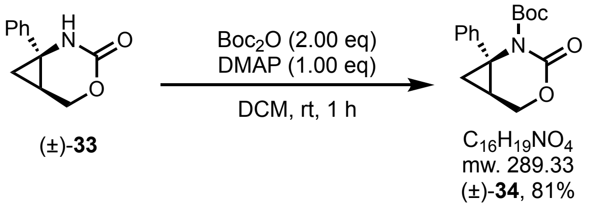

To (±)-**33** (662 mg, 3.50 mmol) in DCM (24 mL) was added DMAP (428 mg, 3.50 mmol) and di-*tert*-butyl dicarbonate (1.52 g, 7.00 mmol). The reaction was stirred for 1 h and concentrated *in vacuo*. The crude residue was purified by silica gel column chromatography (30% EtOAc in DCM) to afford the title compound as a colourless oil (816 mg, 2.82 mmol, 81%).

m.p./°C: 81–84.

^{1}H NMR (400 MHz, CDCl$_3$): δ 7.33–7.29 (m, 2H), 7.26–7.19 (m, 3H), 4.76 (dd, *J* = 12.1, 8.2 Hz, 1H), 3.89 (dd, *J* = 12.1, 8.3 Hz, 1H), 2.09 (dd, *J* = 8.2, 6.4 Hz, 1H), 1.92 (dddd, *J* = 8.3, 8.2, 8.2, 5.7 Hz), 1.40 (s, 9H), 1.40–1.36 (m, 1H).

^{13}C{^{1}H} NMR (101 MHz, CDCl$_3$): δ 152.2, 151.2, 140.1, 128.7, 127.4, 125.6, 83.6, 70.6, 43.5, 28.0, 25.8, 24.2.

ṽ (ATR)/cm^{-1}: 2979, 1783, 1757, 1730, 1453, 1396, 1368, 1286, 1150, 1073, 998, 837.

HRMS: (ESI$^+$, *m/z*) Calcd. for $C_{16}H_{20}NO_4^+$ ([M+H]$^+$) 290.1387. Found: 290.1375.

(±)-**35** - *tert*-butyl ((1*RS*,2*SR*)-2-(hydroxymethyl)-1-phenylcyclopropyl)carbamate

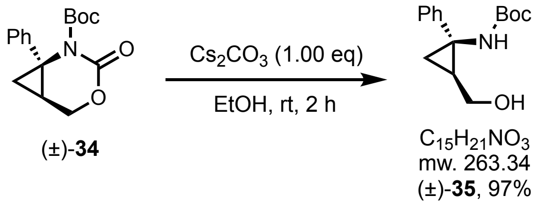

To (±)-**34** (1.14 g, 3.95 mmol) in EtOH (40 mL) was added Cs_2CO_3 (1.39 g, 3.95 mmol). The reaction was stirred for 2 h at room temperature. The reaction was diluted with water (40 mL) and extracted with DCM (3 × 40 mL). The combined organics were dried over $MgSO_4$ and filtered. Concentration *in vacuo* afforded the title compound as a colourless amorphous solid (1.01 g, 3.84 mmol, 97%).

X-ray quality crystals were grown *via* recrystallisation from DCM:cyclohexane (layered); forming as large colourless needles.

m.p./°C: 130–134.

^{1}H NMR (400 MHz, CDCl$_3$): δ 7.33–7.62 (m, 4H), 7.23–7.19 (m, 1H), 5.33 (br s, 1H), 4.06 (app. td, *J* = 11.7, 3.5 Hz, 1H), 3.88 (app. d, *J* = 11.4 Hz, 1H), 3.31 (app. t, *J* = 11.1 Hz, 1H), 1.97 (dddd, *J* = 10.1, 10.1, 6.7, 3.3 Hz, 1H), 1.45 (s, 9H), 1.23 (dd, *J* = 9.6, 5.4 Hz, 1H), 0.82 (dd, *J* = 7.1, 5.4 Hz, 1H).

$^{13}C\{^1H\}$ NMR (101 MHz, $CDCl_3$): δ 157.7, 142.9, 128.6, 126.8, 125.9, 81.0, 62.6, 39.8, 30.9, 28.4, 19.8.

$\tilde{\nu}$ (ATR)/cm^{-1}: 3305 (v br), 2978, 2931, 2874, 1684, 1495, 1366, 1249, 1163, 1076, 1023.

HRMS: (ESI$^+$, *m/z*) Calcd. for $C_{15}H_{22}NO_3^+$ ([M+H]$^+$) 264.1594. Found: 264.1591.

6. Peptide Synthesis

Solid Phase Peptide Synthesis (SPPS)

Resin preloading, deprotection and cleavage reactions were conducted in fritted syringes (Torviq, USA). Syringes were capped and agitated on a rotating table at room temperature during coupling.

Preloading 2-Chlorotrityl Chloride Resin

2-Chlorotrityl chloride resin was swollen in DCM for 30 min then washed with DCM (2 × 3 mL). A solution of Fmoc-W-OH (0.5 eq. relative to resin functionalization) and $i\text{Pr}_2\text{NEt}$ (2.0 eq. relative to resin functionalization) in DCM (final concentration 0.1 M of amino acid) was added and the resin shaken at room temperature for 16 h. The resin was washed with DMF (5 × 3 mL) and DCM (5 × 3 mL). The resin was treated with a solution of DCM/MeOH/$i\text{Pr}_2\text{NEt}$ (17:2:1 v/v/v, 3 mL) for 1 h and washed with DMF (5 × 5 mL), DCM (5 × 5 mL), and DMF (5 × 5 mL).

Deprotection and Estimation of Amino Acid Loading

The resin was treated with 20% piperidine/DMF (2 × 3 mL, 3 min) and 50 µL of the combined deprotection solution was diluted to 10 mL using 20% piperidine/DMF. The UV absorbance of the resulting piperidine-fulvene adduct was measured (λ = 301 nm, ε = 7800 M^{-1} cm^{-1}) to determine the loading of the resin. The deprotected resin was washed with DMF (5 × 5 mL), DCM (5 × 5 mL) and DMF (5 × 5 mL).

Automated Fmoc-SPPS

Automated Fmoc-SPPS was carried out on a Biotage Initiator+ Alstra microwave peptide synthesizer. Standardized amino acid couplings were performed for 15 min at 50 °C under microwave irradiation in the presence of amino acid (0.5 M in DMF, 4 eq.), Oxyma Pure (0.5 M in DMF, 4 eq.) and diisopropylcarbodiimide (0.5 M in DMF, 4 eq.).

Cleavage

A mixture of TFA, thioanisole, triisopropylsilane (TIS) and water (85:5:5:5 v/v/v/v, 6 mL) was added to the resin. After shaking for 4 h, the resin was washed with TFA (3 × 2 mL).

Work-up

The combined cleavage solutions were concentrated under a stream of nitrogen to < 5 mL. 40 mL of diethyl ether was added to precipitate the peptide and the suspension centrifuged. The pellet was then dissolved in water–acetonitrile (95:5 v/v) containing 0.1% TFA, filtered and purified by preparative HPLC.

37 – Osteostatin analogue (H-TRXAW-OH)

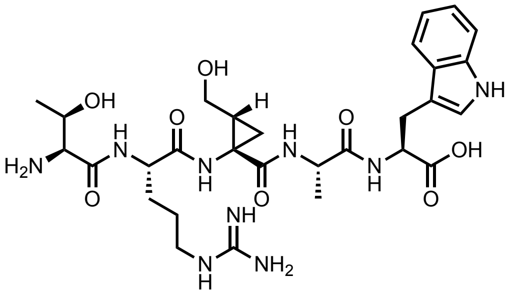

Osteostatin Analogue **H-TRXAW-OH** (**37**) was synthesized on 2-CTC resin (606 mg, 0.80 mmol) using standard Fmoc-SPPS procedures as described above. Residues were coupled using 4.0 eq. of amino acid relative to the estimated Fmoc loading, except for Fmoc-X-OH, which was used at 2.0 eq. relative to the estimated Fmoc loading. Double coupling was applied for Fmoc-X-OH and Fmoc-R-OH. Coupling efficiencies were estimated at each step using the standard estimation of amino acid loading procedure as described above:

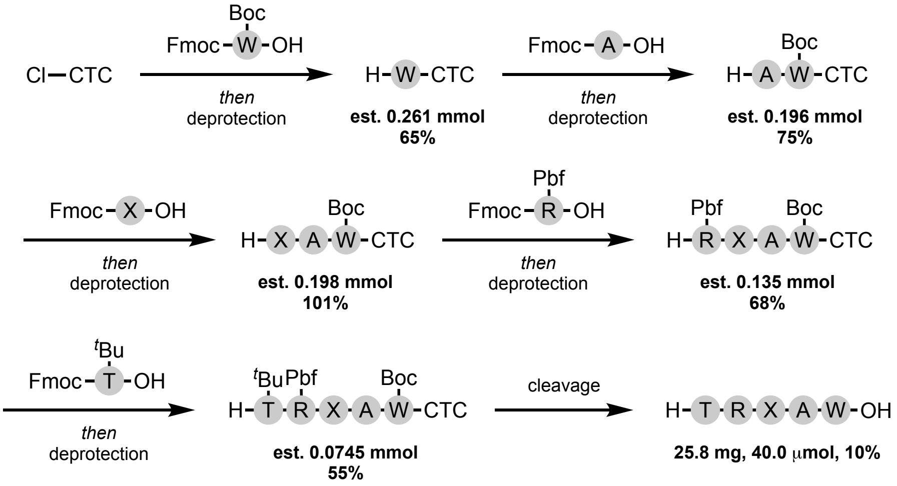

Scheme SI-1. Synthesis of H-TRXAW-OH and estimated amino acid loadings at each step. *Note*: *possible over-estimation of Fmoc-R(Pbf)-OH coupling due to unprotected X side-chain.*

The crude peptide was cleaved and purified by preparative HPLC (5 to 40% B over 30 min) and lyophilized to produce the desired peptide (25.8 mg, 40.0 µmol, 15% yield from H_2N-**W**(Boc)-CTC).

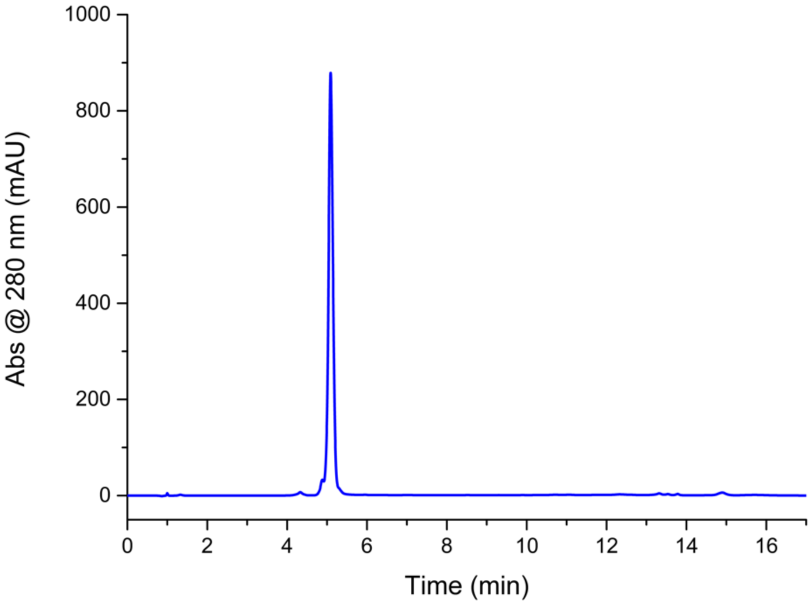

Figure SI-1 Analytical HPLC trace of pure H–TRXAW–OH. Analytical gradient 5–60% B over 5 min 0.6 ml/min, 280 nm. Elution time: 5.090 min.

^{1}H NMR (400 MHz, DMSO-d_6): δ 10.87 (s,1H, W-ArN*H*), 8.88 (d, J = 6.1 Hz, 1H, R N*H*), 8.85 (s, 1H, X-N*H*), 8.21 (d, J = 7.7 Hz, 1H, W-N*H*), 8.17–8.16 (m, 2H, T-NH_2), 7.79 (t, J = 5.7 Hz, 1H, RCH_2N*H*), 7.51 (d, J = 7.7 Hz, 1H, W-5-*H*), 7.33 (d, J = 7.8 Hz, 1H, W-8-*H*), 7.21 (d, J = 7.4 Hz, 1H, A-N*H*), 7.15 (s, 1H, W-2-*H*), 7.05 (dd, J = 7.8, 7.6 Hz, 1H, W-7-*H*), 6.98 (dd, J = 7.7, 7.6 Hz, 1H, W-6-*H*), 5.58 (br s, 1H, O*H*), 4.43 (ddd, J = 7.8, 7.7, 5.5 Hz, 1H, W-α-*H*), 4.36 (dq, J = 7.4, 7.1 Hz, 1H, A-α-*H*), 4.17 (ddd, J = 6.2, 6.2, 6.1 Hz, 1H, R-α-*H*), 3.79 (dq, J = 6.6, 6.4 Hz, 1H, T-C*H*CH_3OH), 3.58 (dd, J = 6.4, 6.2 Hz, 1H, T-α-*H*), 3.31–3.28 (m, 1H, X-CH_2OH and W-CH_2), 3.03 (dd, J = 14.7, 7.8 Hz, 1H, W-CH_2), 1.90 (dddd, J = 8.6, 7.6, 7.5, 7.3 Hz, 1H, X-C*H*), 1.71–1.47 (m, 4H, R-(CH_2)$_2$$CH_2$N), 1.29 (dd, J = 8.6, 4.5 Hz, 1H, X-CypCH_2), 1.20 (d, J = 7.1 Hz, 3H, A-CH_3), 1.16 (d, J = 6.6 Hz, 3H, T-CH_3), 0.73 (dd, J = 7.5, 4.6 Hz, 1H, X-CypCH_2).

^{13}C{^{1}H} NMR (101 MHz, DMSO-d_6): δ 173.6 (*C*=O), 173.2 (*C*=O), 172.2 (*C*=O), 169.8 (*C*=O), 167.5 (*C*=O), 156.8 (R-NH*C*NH_2=NH), 136.0 (W-*C*9), 127.2 (W-*C*4), 123.8 (W-*C*2), 120.9 (W-*C*7), 118.4 (W-*C*6), 118.1 (W-*C*5), 111.4 (W-*C*8), 109.5 (W-*C*3), 66.0 (T-*C*HCH_3OH), 60.1 (X-CH_2OH), 58.1 (T-α-*C*), 53.4 (R-α-*C*), 53.0 (W-α-*C*), 48.1 (A-α-*C*), 40.4 (R-CH_2N), 37.7 (X-α-*C*), 28.3 (X-*C*H), 28.1 (R-*C*H_2), 27.0 (W-*C*H_2), 25.1 (R-*C*H_2), 19.8 (T-*C*H_3), 18.9 (X-Cyp*C*H_2), 18.3 (A-*C*H_3).

HRMS: (ESI$^+$, *m/z*) Calcd. for $C_{29}H_{44}N_9O_8^+$ ([M+H]$^+$) 646.3307. Found: 646.3298 (1.50 ppm error). Calcd. for $C_{29}H_{45}N_9O_8^{2+}$ ([M+2H]$^{2+}$) 323.6690. Found: 323.6699 (2.76 ppm error).

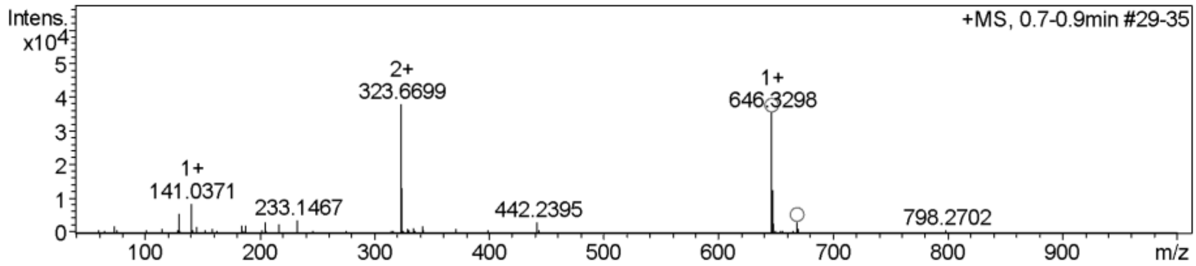

7. Miscellaneous Procedures

(±)-**27** - 2-(2-chloroethyl)oxirane

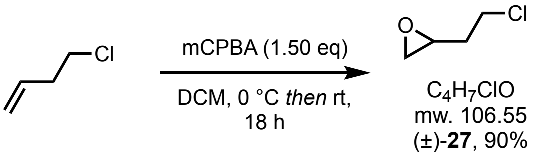

A solution of 4-chloro-1-butene (2.02 mL, 20.0 mmol) in DCM (20 mL) was cooled to 0 °C, and *m*CPBA (6.09 g, 30.0 mmol; 85% purity) was added in five portions over 15 min. The reaction was warmed to room temperature and stirred for 18 h. The reaction was quenched with NaOH (40 mL, 2 M aq. solution), and the layers were separated. The aqueous layer was extracted with DCM (2 × 20 mL) and the combined organic layers were washed with NaOH (80 mL, 2M aq. solution), and washed sequentially with water (5 × 40 mL), until the aq. layer was pH 7. The organic layer was dried over Na_2SO_4, filtered and concentrated *in vacuo* to afford the title compound as a colourless liquid (1.92 g, 18.0 mmol, 90%). Due to the title compound's partial volatility, the compound was isolated as a 58% solution in DCM; and used without further purification.
Characterisation data were consistent with the literature values: ^{1}H NMR and ^{13}C{^{1}H} NMR.[7]

^{1}H NMR (400 MHz, $CDCl_3$): δ 3.72–3.62 (m, 2H), 3.09 (app. ddt, *J* = 6.7, 4.2, 2.1 Hz, 1H), 2.83 (app. t, *J* = 4.4 Hz, 1H), 2.56 (dd, *J* = 5.0, 2.6 Hz, 1H), 2.07 (dtd, *J* = 14.6, 7.2, 4.5 Hz, 1H), 1.93 (app. dq, *J* = 14.6, 6.0 Hz, 1H).

^{13}C{^{1}H} NMR (101 MHz, $CDCl_3$): δ 49.9, 47.2, 41.4, 35.7.

ṽ (ATR)/cm^{-1}: 3055, 2997, 2966, 2926, 1484, 1448, 1426, 1413, 1325, 1294, 1261, 1242, 1216, 1134, 1090, 1067, 1021, 986, 961, 909, 867, 858, 834, 821.

(±)-**29** - diethyl 3-hydroxycyclopentane-1,1-dicarboxylate

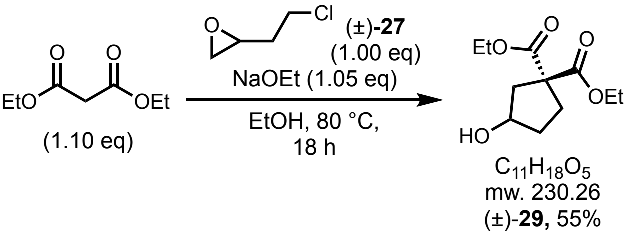

A flame-dried Schlenk RBF, sealed with Suba-Seal septum, was evacuated and backfilled thrice with anhydrous dinitrogen. Anhydrous ethanol (24 mL) was added, and the flask was cooled to 0 °C. The septum was removed and sodium hydride (504 mg, 12.6 mmol, 60% dispersion in mineral oil) was added in three portions over 15 min and allowed to stir until dissolved (approx. 5 min). To the sodium ethoxide solution was added diethyl malonate (2.00 mL, 13.2 mmol) in one portion. The flask was warmed to room temperature and allowed to stir for 10 min. 2-(2-chloroethyl)oxirane (±)-**27** (1.13 mL, 12.0 mmol) was added dropwise (approx. 5 min) and stirred for 15 min at room temperature, before the

reaction vessel was placed into a preheated DrySyn heating block at 80 °C and stirred for 18 h. The reaction was cooled to room temperature, before being cooled to 0 °C, and the white suspension was filtered through a pad of celite and washed through with ethanol (2 × 12 mL). The pale-yellow solution was concentrated *in vacuo*. To the crude oil was added water (30 mL) and DCM (25 mL); the organic layer was separated, and the aqueous layer was extracted with DCM (2 × 25 mL). The combined organics were dried over anhydrous Na_2SO_4, filtered and concentrated *in vacuo*. The crude residue was purified *via* silica gel column chromatography (30% EtOAc in cyclohexane) to afford the title compound as a colourless oil (1.53 g, 6.66 mmol, 55%).
Attempts to cyclise the title compound to the bridged lactone under both basic or acidic conditions were unsuccessful.

^{1}H NMR (400 MHz, $CDCl_3$): δ 4.39 (br s, 1H), 4.28–4.11 (m, 4H), 2.52–2.38 (m, 1H), 2.38–2.32 (m, 2H), 2.32–2.21 (m, 2H), 2.00–1.86 (m, 1H), 1.83–1.71 (m, 1H), 1.31–1.19 (m, 6H).

^{13}C{^{1}H} NMR (101 MHz, $CDCl_3$): δ 173.5, 172.3, 73.4, 61.9, 61.6, 59.3, 43.4, 35.3, 32.1, 14.12, 14.10.

ṽ (ATR)/cm^{-1}: 3435 (v br), 2982, 2907, 1721, 1465, 1446, 1390, 1367, 1297, 1250, 1211, 1175, 1155, 1096, 1076, 1039, 964, 946, 861.

HRMS: (ESI$^+$, *m/z*) Calcd. for $C_{11}H_{18}NaO_5^+$ ($[M+Na]^+$) 253.1047. Found: 253.1036.

(±)-**SI-1** - ethyl (1*RS*,2*RS*)-2-(((*tert*-butyldimethylsilyl)oxy)methyl)-1-carbamoylcyclopropane-1-carboxylate

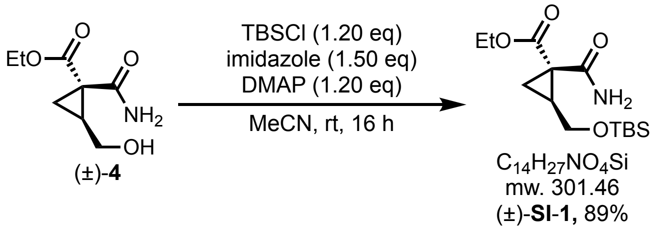

To a flame-dried Schlenk RBF was added (±)-**4** (936 mg, 5.00 mmol), imidazole (511 mg, 7.50 mmol) and DMAP (733 mg, 6.00 mmol). The flask was evacuated and backfilled thrice with anhydrous dinitrogen. TBSCl (904 mg, 6.00 mmol) was added under a flow of nitrogen and the solids were solubilised in anhydrous MeCN (25 mL). The reaction mixture was stirred for at room temperature for 16 h. The reaction mixture was diluted with water (10 mL) and extracted with DCM (3 × 15 mL). The combined organic layers were washed with HCl (2 × 15 mL; 1 M aq. solution), brine (15 mL), dried over anhydrous Na_2SO_4, filtered and concentrated *in vacuo*. The crude residue was purified *via* silica gel column chromatography (25% EtOAc in cyclohexane) to afford the title compound as a colourless solid (1.35 g, 4.47 mmol, 89%).

m.p./°C: 58–60.

^{1}H NMR (400 MHz, CDCl$_3$): δ 8.09 (br s, 1H), 5.57 (br s, 1H), 4.16 (q, *J* = 7.1 Hz, 2H), 3.92 (dd, *J* = 11.3, 5.4 Hz, 1H), 3.65 (dd, *J* = 11.3, 8.5 Hz, 1H), 2.11 (app. dtd, *J* = 9.3, 8.3, 5.4 Hz, 1H), 1.80–1.69 (m, 2H), 1.25 (t, *J* = 7.1 Hz, 3H), 0.88 (s, 9H), 0.05 (s, 3H), 0.04 (s, 3H).

^{13}C{^{1}H} NMR (101 MHz, CDCl$_3$): δ 172.4, 168.8, 61.6, 61.1, 35.3, 32.1, 26.0, 19.5, 18.4, 14.2, -5.16, -5.21.

$\tilde{\nu}$ (ATR)/cm^{-1}: 3187, 2997, 2950, 2927, 2882, 2855, 1712, 1667, 1576, 1468, 1441, 1412, 1394, 1369, 1298, 1252, 1149, 1108, 1084, 1055, 1039, 1015, 938, 889, 863, 831.

HRMS: (ESI$^+$, *m/z*) Calcd. for $C_{14}H_{27}NNaO_4Si^+$ ([M+Na]$^+$) 324.1602. Found: 324.1587.

(±)-**SI-2** - ethyl (1*SR*,2*RS*)-1-((*tert*-butoxycarbonyl)amino)-2-(((*tert*-butyldimethylsilyl)oxy)methyl)cyclopropane-1-carboxylate

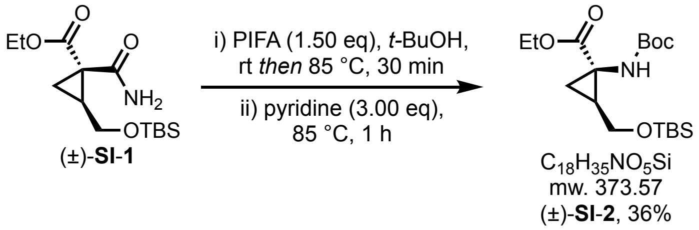

Using PIFA: To a flame-dried Schlenk RBF was added (±)-**SI-1** (1.11 g, 3.68 mmol). The flask was evacuated and backfilled thrice with anhydrous dinitrogen. Anhydrous *t*-BuOH (9.2 mL) was added, the reaction was placed in a 25 °C heating block and the vessel was wrapped in aluminium foil. Bis(trifluoroacetoxy)iodo)benzene (PIFA; 2.37 g, 5.52 mmol) was added in four portions – under a flow of nitrogen – over 30 min, with stirring. The reaction mixture was heated to 85 °C and stirred for a further 30 min. Anhydrous pyridine (893 µL, 11.0 mmol) was added in one portion*** and the reaction stirred for 1 h. The reaction mixture was cooled to room temperature, diluted with $NaHCO_3$ (50 mL, sat. aq. solution) and extracted with EtOAc (4 × 50 mL). The combined organic layers were washed with $Na_2S_2O_3$ (50 mL, 12% aq. solution), brine (50 mL), dried over Na_2SO_4, filtered and concentrated *in vacuo*. The crude residue was purified by silica gel column chromatography (8% EtOAc in cyclohexane) to afford the title compound as a colourless oil (494 mg, 1.32 mmol, 36%).

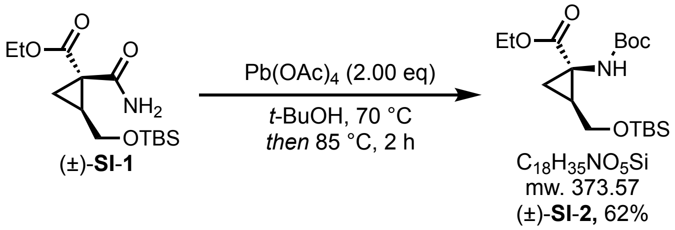

Using Pb(OAc)$_4$: To a flame-dried Schlenk RBF was added (±)-**SI-1** (301 mg, 1.00 mmol). The flask was evacuated and backfilled thrice with anhydrous dinitrogen. Anhydrous *t*-BuOH (4 mL) was added, and the reaction was heated to 70 °C. Lead(IV) acetate (887 mg, 2.00 mL) was added under a flow of nitrogen and the reaction was heated to 85 °C, with stirring, for 2 h. The reaction mixture was cooled

*** 10 °C exotherm observed on pyridine addition.

to room temperature and $NaHCO_3$ (500 mg, 5.95 mmol) was added. The mixture was diluted with $Na_2S_2O_3$ (20 mL, 12% aq. solution) and extracted with EtOAc (4 × 10 mL). The combined organics were washed with brine (20 mL), dried over Na_2SO_4, filtered and concentrated *in vacuo*. The crude residue was purified by silica gel column chromatography (8% EtOAc in cyclohexane) to afford the title compound as a colourless oil (232 mg, 0.62 mmol, 62%).

Only ^{1}H NMR data has been previously reported for this compound.[8] Observed ^{1}H data is largely consistent; though a number of small shift discrepancies and difference in the location of TBS dimethyl ^{1}H environment are observed.

^{1}H NMR (400 MHz, $CDCl_3$, rotamers present): δ 5.30 (br s, 0.75H), 5.10 (br s, 0.25H), 4.21–4.06 (m, 2H), 3.97 (dd, J = 11.4, 5.6 Hz, 1H), 3.47 (app. t, J = 10.4 Hz, 1H), 1.93–1.83 (m, 1H), 1.81–1.73 (m, 1H), 1.44 (s, 9H), 1.26–1.20 (m, 3H), 1.12 (s, 1H), 0.88 (s, 9H), 0.06 (s, 3H), 0.05 (s, 3H).

^{13}C{^{1}H} NMR (101 MHz, $CDCl_3$): δ 173.0, 156.6, 79.8, 63.0, 61.4, 38.1, 29.6, 28.4, 26.0, 22.0, 18.3, 14.3, -5.1.

ṽ (ATR)/cm^{-1}: 3434, 3357, 2956, 2930, 2857, 1725 (br), 1473, 1391, 1367, 1334, 1249, 1163, 1081, 1047, 969, 939, 834, 814.

HRMS: (ESI$^+$, *m/z*) Calcd. for $C_{18}H_{36}NO_5Si^+$ ([M+H]$^+$) 374.2357. Found: 374.2345.

(±)-**15** - ethyl (1*SR*,2*RS*)-1-((*tert*-butoxycarbonyl)amino)-2-(hydroxymethyl)cyclopropane-1-carboxylate - *via* TBS deprotection of (±)-**SI-2**

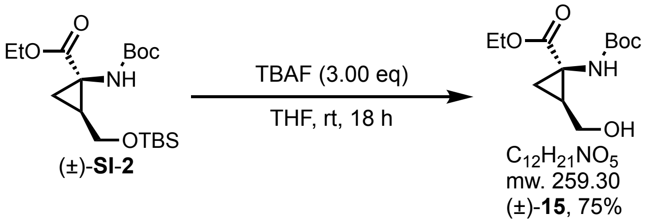

To (±)-**SI-2** (1.07 g, 2.86 mmol) in THF (20 mL) was added TBAF (8.58 mL, 8.58 mmol, 1 M solution in THF). The reaction mixture was stirred at room temperature for 16 h. The reaction was diluted with $NaHCO_3$ (50 mL, sat. aq. solution), and extracted with DCM (3 × 30 mL). The combined organics were washed with brine (50 mL), dried over Na_2SO_4, filtered and concentrated *in vacuo*. The crude residue was purified *via* silica gel column chromatography (30% EtOAc in cyclohexane) to afford the title compound as a colourless oil (557 mg, 2.15 mmol, 75%).

Characterisation data were consistent with the literature values: ^{1}H NMR, ^{13}C{^{1}H} NMR and IR.[2]

^{1}H NMR (400 MHz, $CDCl_3$): δ 5.13 (br s, 1H), 4.25–4.06 (m, 2H), 3.97 (ddd, J = 12.1, 11.2, 3.4 Hz, 1H), 3.74 (d, J = 11.2 Hz, 1H), 3.21 (app. t, J = 11.0 Hz, 1H), 2.35–2.18 (m, 1H), 1.54 (dd, J = 9.8, 4.9 Hz, 1H), 1.47 (s, 9H) 1.24 (t, J = 7.1 Hz, 3H), 0.81–0.74 (m, 1H).

^{13}C{^{1}H} NMR (101 MHz, $CDCl_3$): δ 172.4, 158.4, 81.4, 61.7, 61.6, 38.6, 31.2, 28.3, 19.2, 14.3.

ṽ (ATR)/cm^{-1}: 3350 (br), 2979, 2933, 1724, 1692, 1506, 1457, 1393, 1367, 1326, 1286, 1251, 1158, 1086, 1026, 966, 915, 860, 832.

HRMS: (ESI$^+$, *m/z*) Calcd. for $C_{12}H_{22}NO_5^+$ ([M+H]$^+$) 260.1492. Found: 260.1494.

8. Exploration of Hofmann Rearrangement Conditions

The following conditions were explored for the Hofmann rearrangement, including the comparison of intermolecular and intramolecular quenching. Entries 4 and 6 were repeated at the larger scales of 5 mmol and 20 mmol to ensure their feasibility; Entry 4 was only found to be reproducible up to 5 mmol scale, before the yields began to drop off considerably, with just a 30% yield at 20 mmol scale. Controlling exotherms, addition portion sizes and reaction times did not aid reproducibility at larger scales.

As previously stated by Crane and co-workers,[9] several oxidants (TCCA, NBC, NCS) were found to be incompatible with *tert*-butanol, forming black amorphous aggregates.

Attempts to perform the Hofmann rearrangement of (±)-**SI-1** in the presence of additional water, to permit hydrolysis of the isocyanate to the free amine, were unsuccessful across a variety of solvent and base systems.

Table SI-1. Screening Hofmann rearrangement conditions [1.00 mmol].

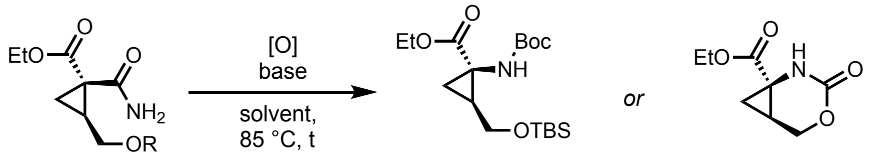

R = H, (±)-**4**
TBS, (±)-**SI-1**

(±)-**SI-2**

(±)-**5**

Entry	[O] (eq)	R	Solvent	Base (eq)	t	Prod	% Yield
1	$Pb(OAc)_4$ (2.00)	TBS (**SI-1**)	*t*-BuOH	---	2 h	**SI-2**	64
2	NBS (2.00)	TBS (**SI-1**)	*t*-BuOH	DBU (2.00)	2 h	**SI-2**	0
3	PIFA (1.50)	TBS (**SI-1**)	*t*-BuOH	pyridine (3.00)	2 h	**SI-2**	36
4	NBS (2.00)	H (**4**)	MeCN	DBU (2.00)	1 h	**5**	40
5	PIFA (1.50)	H (**4**)	MeCN	pyridine (3.00)	2 h	5	45
6	TCCA (0.38)	H (**4**)	MeCN	DBU (2.25)	18 h	5	55*

Despite the slightly lower yield for Entry 6, compared with Entry 1; the method is preferable due to avoidance of neurotoxic $Pb(OAc)_4$, and thus the generation of stoichiometric lead waste; and removal of the highly atom-inefficient TBS protection stage required for the generation of (±)-15. In addition, to the versatility of the cyclic carbamate, and ease of handling – due to its crystalline solid state.

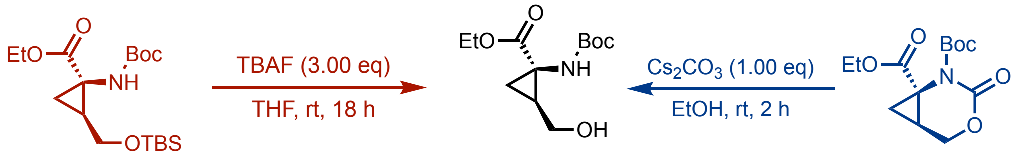

(±)-**SI-2**, 62%

(±)-**15**, 75% / > 99%

(±)-**6**, 94%

9. Unsuccessful Transformations

Many seemingly trivial transformations were trialled on the diversifiable substrates within this paper. These and some accompanying observations are documented below.

Unsuccessful Carbamate Transformations

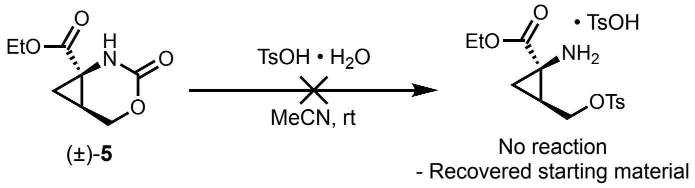

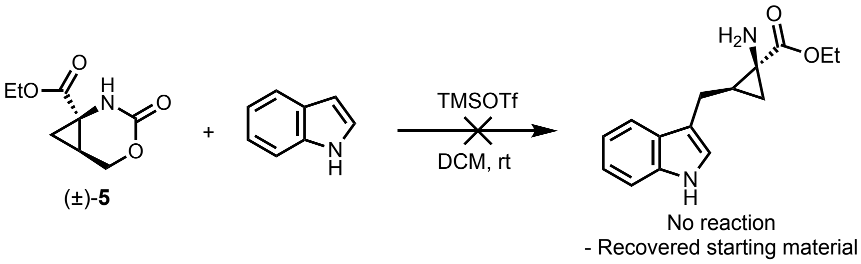

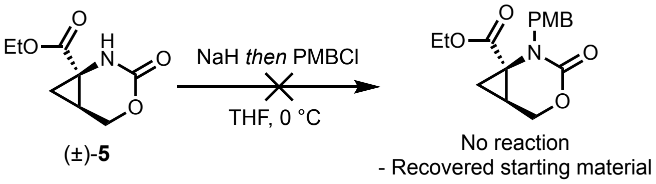

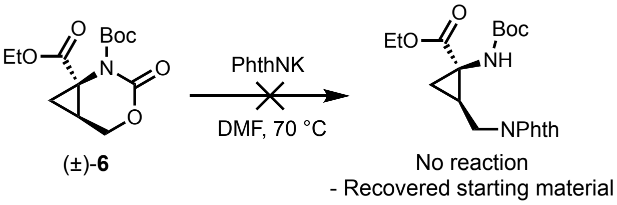

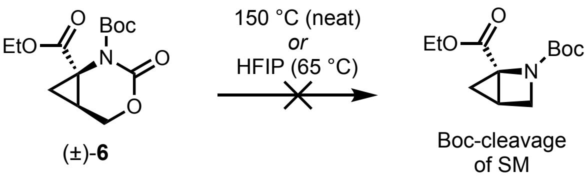

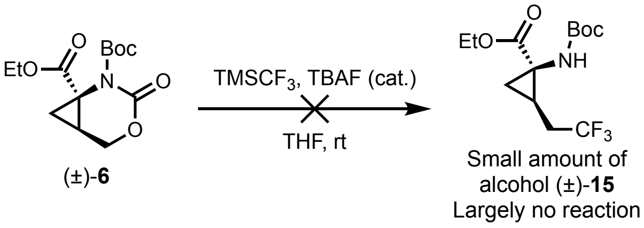

Unsuccessful Bromide Transformations

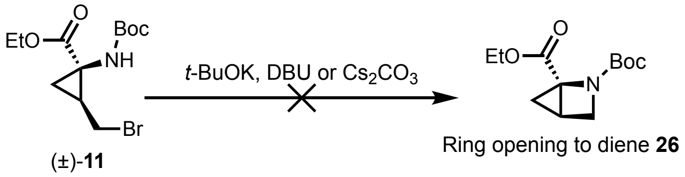

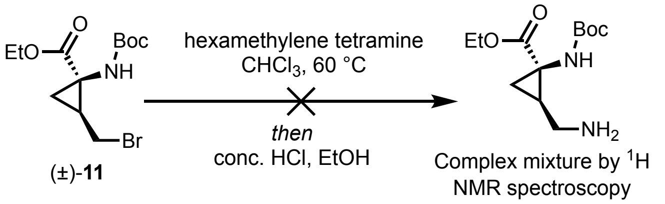

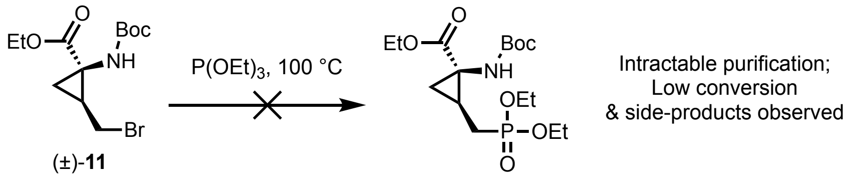

Unsuccessful Alcohol Transformations

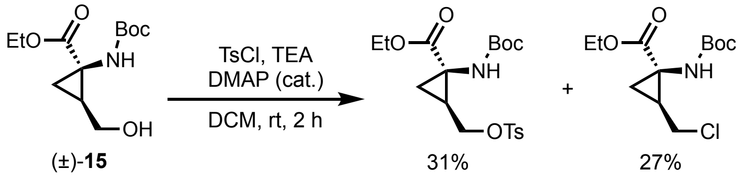

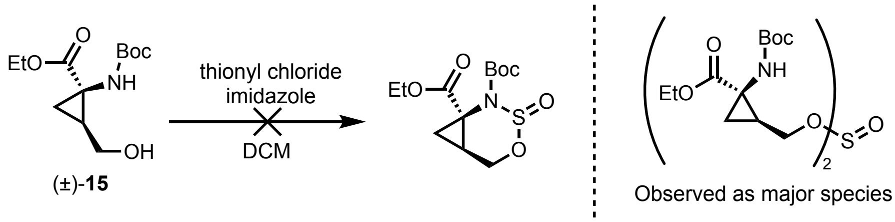

Unsuccessful Aldehyde Transformations

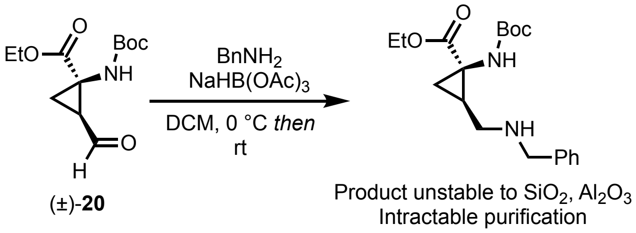

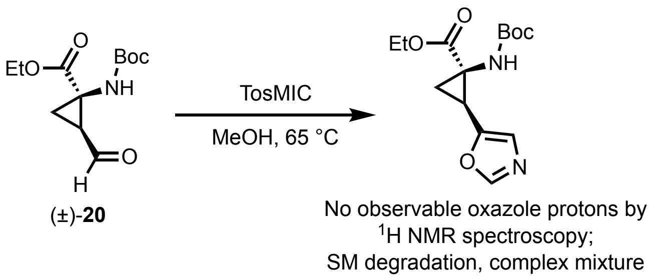

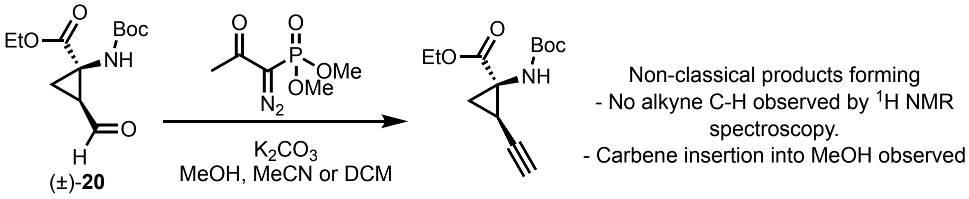

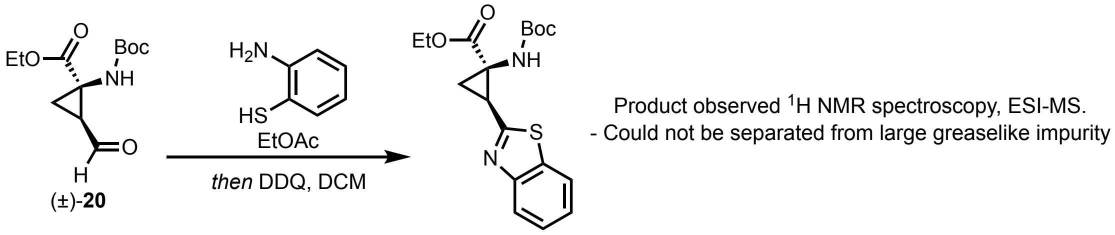

Miscellaneous Unsuccessful Transformations

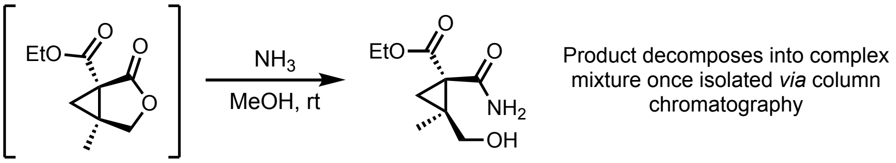

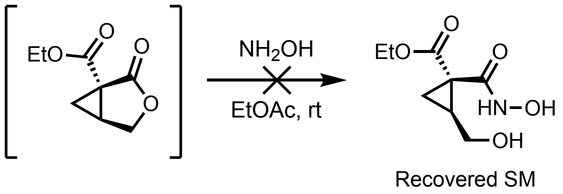

Unsuccessful Fluorination Attempts

Due to the benefits of fluorine incorporation into drug like molecules,[10] simple methods for its incorporation into our amino acid scaffolds are highly sought after. Many of the unsuccessful attempts are documented here:

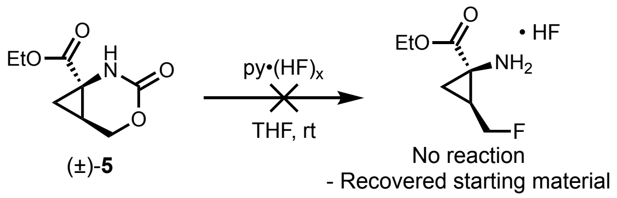

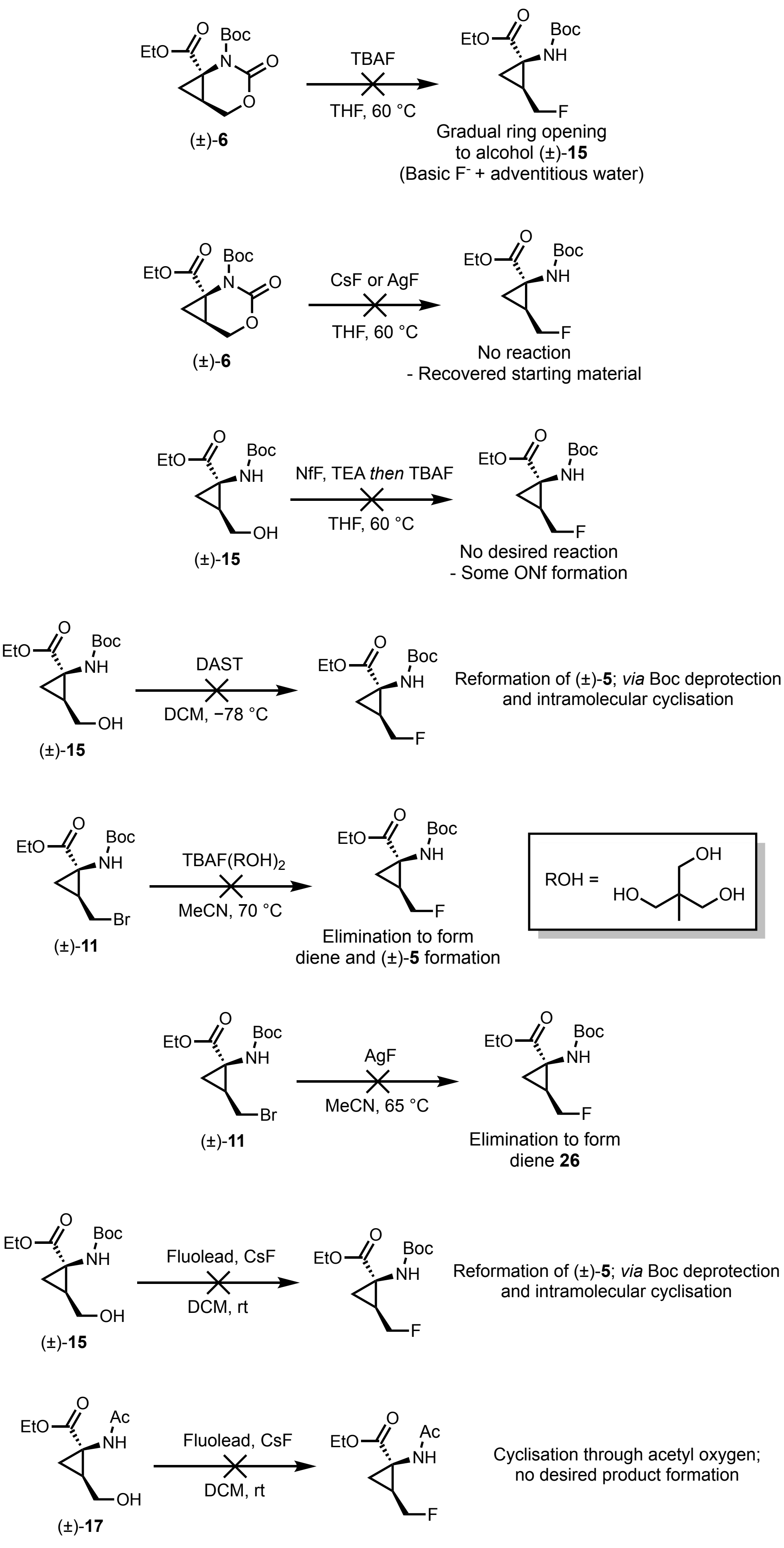

(±)-6
TBAF
THF, 60 °C
Gradual ring opening to alcohol (±)-15 (Basic F⁻ + adventitious water)
(±)-6
CsF or AgF
THF, 60 °C
No reaction - Recovered starting material
(±)-15
NfF, TEA then TBAF
THF, 60 °C
No desired reaction - Some ONf formation
(±)-15
DAST
DCM, −78 °C
Reformation of (±)-5; via Boc deprotection and intramolecular cyclisation
(±)-11
TBAF(ROH)2
MeCN, 70 °C
Elimination to form diene and (±)-5 formation
ROH =
(±)-11
AgF
MeCN, 65 °C
Elimination to form diene 26
(±)-15
Fluolead, CsF
DCM, rt
Reformation of (±)-5; via Boc deprotection and intramolecular cyclisation
(±)-17
Fluolead, CsF
DCM, rt
Cyclisation through acetyl oxygen; no desired product formation

10. X-Ray Diffraction Data Tables

(±)-**4** - ethyl (1*RS*,2*RS*)-1-carbamoyl-2-(hydroxymethyl)cyclopropane-1-carboxylate

H atoms omitted for clarity. Data obtained and solved by Dr Stephen Argent [CCDC 2386984]. ORTEP diagram shown with 50% probability thermal ellipsoids.

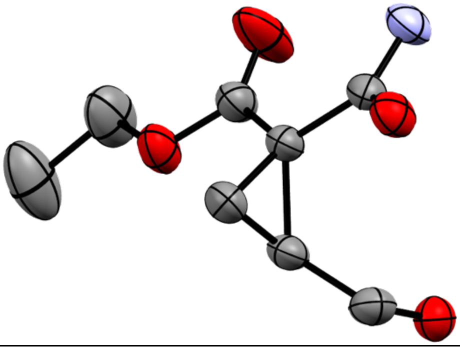

Empirical formula	$C_8H_{13}NO_4$
Formula weight	187.19
Temperature/K	220(2)
Crystal system	triclinic
Space group	P-1
a/Å	6.53940(10)
b/Å	8.73660(10)
c/Å	9.4989(2)
α/°	106.948(2)
β/°	101.010(2)
γ/°	105.559(2)
Volume/Å^3	478.289(15)
Z	2
ρ_{calc}g/cm^3	1.300
μ/mm^{-1}	0.885
F(000)	200.0
Crystal size/mm^3	0.279 × 0.099 × 0.038
Radiation	Cu Kα (λ = 1.54184)
2Θ range for data collection/°	10.18 to 158.186
Index ranges	$-8 \leq h \leq 8$, $-11 \leq k \leq 11$, $-12 \leq l \leq 12$
Reflections collected	8889
Independent reflections	2008 [R_{int} = 0.0164, R_{sigma} = 0.0112]
Data/restraints/parameters	2008/3/128
Goodness-of-fit on F^2	1.036
Final R indexes [I>=2σ (I)]	R_1 = 0.0410, wR_2 = 0.1126
Final R indexes [all data]	R_1 = 0.0425, wR_2 = 0.1140
Largest diff. peak/hole / e Å^{-3}	0.23/-0.26

(±)-**5** - ethyl (1*SR*,6*RS*)-3-oxo-4-oxa-2-azabicyclo[4.1.0]heptane-1-carboxylate

H atoms omitted for clarity. Data obtained and solved by Dr Stephen Argent [CCDC 2386988]. ORTEP diagram shown with 50% probability thermal ellipsoids.

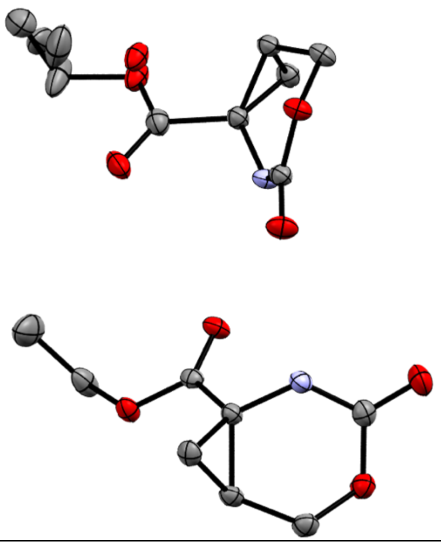

Empirical formula	$C_8H_{11}NO_4$
Formula weight	185.18
Temperature/K	120(2)
Crystal system	triclinic
Space group	P-1
a/Å	9.0721(4)
b/Å	9.8864(6)
c/Å	10.7300(7)
α/°	76.838(5)
β/°	68.454(5)
γ/°	73.225(5)
Volume/Å^3	849.00(9)
Z	4
ρ_{calc}g/cm^3	1.449
μ/mm^{-1}	0.997
F(000)	392.0
Crystal size/mm^3	0.198 × 0.144 × 0.113
Radiation	Cu Kα (λ = 1.54184)
2Θ range for data collection/°	8.944 to 144.87
Index ranges	$-11 \leq h \leq 11$, $-12 \leq k \leq 12$, $-12 \leq l \leq 12$
Reflections collected	12933
Independent reflections	3290 [R_{int} = 0.0212, R_{sigma} = 0.0175]
Data/restraints/parameters	3290/157/267
Goodness-of-fit on F^2	1.059
Final R indexes [I>=2σ (I)]	R_1 = 0.0356, wR_2 = 0.0944
Final R indexes [all data]	R_1 = 0.0379, wR_2 = 0.0965
Largest diff. peak/hole / e Å^{-3}	0.32/-0.39

(±)-**6** - 2-(*tert*-butyl) 1-ethyl (1*SR*,6*RS*)-3-oxo-4-oxa-2-azabicyclo[4.1.0]heptane-1,2-dicarboxylate

H atoms omitted for clarity. Data obtained and solved by Dr Stephen Argent [CCDC 2386986]. ORTEP diagram shown with 50% probability thermal ellipsoids.

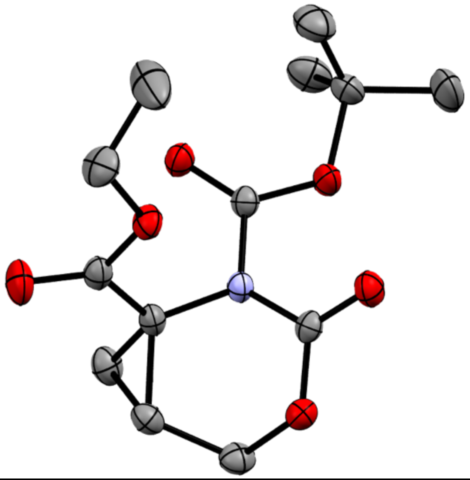

Empirical formula	$C_{13}H_{19}NO_6$
Formula weight	285.29
Temperature/K	120(2)
Crystal system	monoclinic
Space group	$P2_1/n$
a/Å	10.9263(2)
b/Å	8.14150(10)
c/Å	17.1356(4)
α/°	90
β/°	108.434(2)
γ/°	90
Volume/Å^3	1446.11(5)
Z	4
ρ_{calc}g/cm^3	1.310
μ/mm^{-1}	0.879
F(000)	608.0
Crystal size/mm^3	0.22 × 0.12 × 0.07
Radiation	Cu Kα (λ = 1.54184)
2Θ range for data collection/°	8.544 to 145.39
Index ranges	$-13 \le h \le 13$, $-10 \le k \le 10$, $-20 \le l \le 18$
Reflections collected	21598
Independent reflections	2861 [R_{int} = 0.0196, R_{sigma} = 0.0099]
Data/restraints/parameters	2861/0/185
Goodness-of-fit on F^2	1.074
Final R indexes [I>=2σ (I)]	R_1 = 0.0345, wR_2 = 0.0908
Final R indexes [all data]	R_1 = 0.0358, wR_2 = 0.0921
Largest diff. peak/hole / e Å^{-3}	0.23/-0.24

(±)-**11** - ethyl (1*RS*,2*SR*)-2-(bromomethyl)-1-((*tert*-butoxycarbonyl)amino)cyclopropane-1-carboxylate

H atoms omitted for clarity. Data obtained and solved by Dr Stephen Argent [CCDC 2386989]. ORTEP diagram shown with 50% probability thermal ellipsoids.

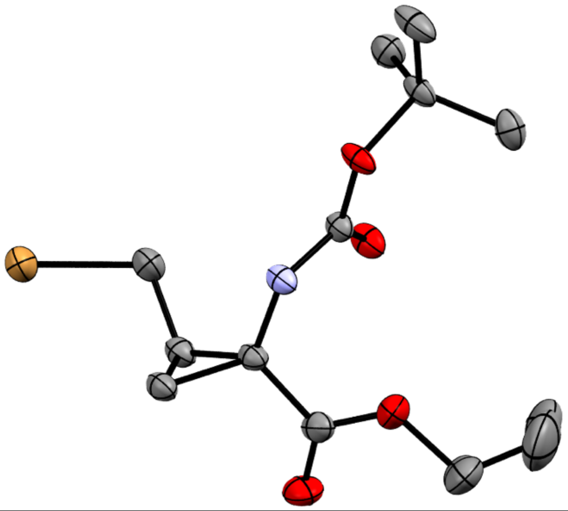

Empirical formula	$C_{12}H_{20}NO_4Br$
Formula weight	322.20
Temperature/K	120(2)
Crystal system	monoclinic
Space group	I2/a
a/Å	19.7593(3)
b/Å	5.07840(10)
c/Å	30.5806(5)
α/°	90
β/°	99.141(2)
γ/°	90
Volume/Å^3	3029.66(9)
Z	8
ρ_{calc}g/cm^3	1.413
μ/mm^{-1}	3.771
F(000)	1328.0
Crystal size/mm^3	0.169 × 0.038 × 0.038
Radiation	Cu Kα (λ = 1.54184)
2Θ range for data collection/°	9.066 to 145.518
Index ranges	$-24 \leq h \leq 24$, $-6 \leq k \leq 6$, $-37 \leq l \leq 37$
Reflections collected	22481
Independent reflections	2998 [R_{int} = 0.0295, R_{sigma} = 0.0154]
Data/restraints/parameters	2998/70/190
Goodness-of-fit on F^2	1.030
Final R indexes [I>=2σ (I)]	R_1 = 0.0264, wR_2 = 0.0694
Final R indexes [all data]	R_1 = 0.0275, wR_2 = 0.0704
Largest diff. peak/hole / e Å^{-3}	0.55/-0.60

(±)-**19** - (1*SR*,2*RS*)-2-((*tert*-butoxycarbonyl)amino)-2-(ethoxycarbonyl)cyclopropane-1-carboxylic acid

H atoms omitted for clarity. Data obtained and solved by Dr Stephen Argent [CCDC 2386985]. ORTEP diagram shown with 50% probability thermal ellipsoids.

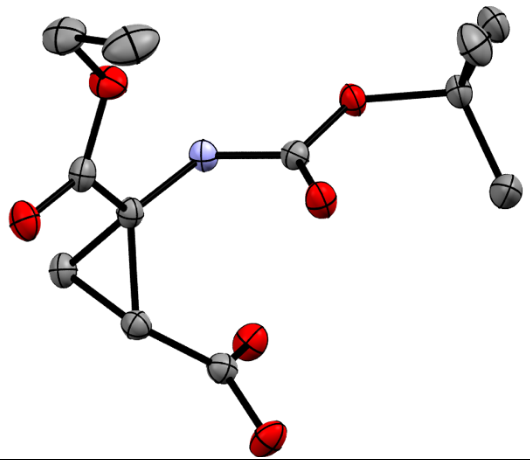

Empirical formula	$C_{12}H_{19}NO_6$
Formula weight	273.28
Temperature/K	120(2)
Crystal system	triclinic
Space group	P-1
a/Å	8.9590(2)
b/Å	9.3692(2)
c/Å	9.6826(2)
α/°	64.354(2)
β/°	75.592(2)
γ/°	82.809(2)
Volume/Å^3	709.49(3)
Z	2
ρ_{calc}g/cm^3	1.279
μ/mm^{-1}	0.871
F(000)	292.0
Crystal size/mm^3	0.659 × 0.056 × 0.033
Radiation	Cu Kα (λ = 1.54184)
2Θ range for data collection/°	10.196 to 151.266
Index ranges	$-10 \leq h \leq 9$, $-11 \leq k \leq 11$, $-12 \leq l \leq 12$
Reflections collected	13045
Independent reflections	2853 [R_{int} = 0.0251, R_{sigma} = 0.0170]
Data/restraints/parameters	2853/2/182
Goodness-of-fit on F^2	1.091
Final R indexes [I>=2σ (I)]	R_1 = 0.0340, wR_2 = 0.0920
Final R indexes [all data]	R_1 = 0.0360, wR_2 = 0.0941
Largest diff. peak/hole / e Å^{-3}	0.27/-0.27

(±)-**33** - (1*RS*,6*SR*)-1-phenyl-4-oxa-2-azabicyclo[4.1.0]heptan-3-one

H atoms omitted for clarity. Data obtained and solved by Dr Stephen Argent [CCDC 2386990]. ORTEP diagram shown with 50% probability thermal ellipsoids.

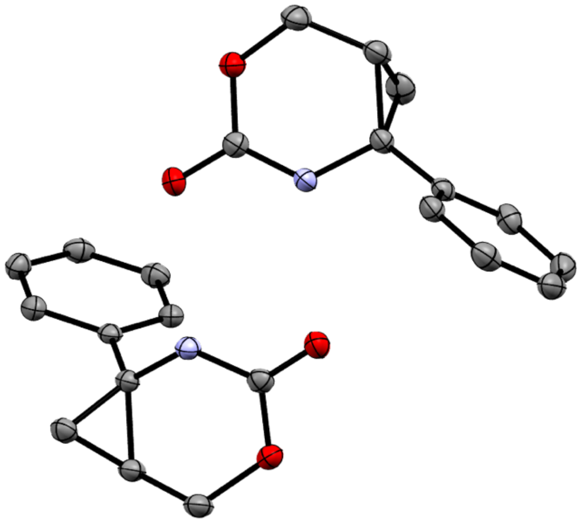

Empirical formula	$C_{11}H_{11}NO_2$
Formula weight	189.21
Temperature/K	120(2)
Crystal system	monoclinic
Space group	$P2_1/n$
a/Å	15.0632(2)
b/Å	7.33630(10)
c/Å	17.1436(2)
α/°	90
β/°	98.0240(10)
γ/°	90
Volume/Å^3	1875.96(4)
Z	8
ρ_{calc}g/cm^3	1.340
μ/mm^{-1}	0.758
F(000)	800.0
Crystal size/mm^3	0.252 × 0.168 × 0.079
Radiation	Cu Kα (λ = 1.54184)
2Θ range for data collection/°	7.324 to 145.722
Index ranges	$-18 \le h \le 18$, $-8 \le k \le 8$, $-21 \le l \le 21$
Reflections collected	27870
Independent reflections	3704 [R_{int} = 0.0261, R_{sigma} = 0.0134]
Data/restraints/parameters	3704/2/259
Goodness-of-fit on F^2	1.036
Final R indexes [I>=2σ (I)]	R_1 = 0.0327, wR_2 = 0.0836
Final R indexes [all data]	R_1 = 0.0345, wR_2 = 0.0851
Largest diff. peak/hole / e Å^{-3}	0.23/-0.24

(±)-**35** - *tert*-butyl ((1*RS*,2*SR*)-2-(hydroxymethyl)-1-phenylcyclopropyl)carbamate

H atoms omitted for clarity. Data obtained and solved by Dr Stephen Argent [CCDC 2386987]. ORTEP diagram shown with 50% probability thermal ellipsoids.

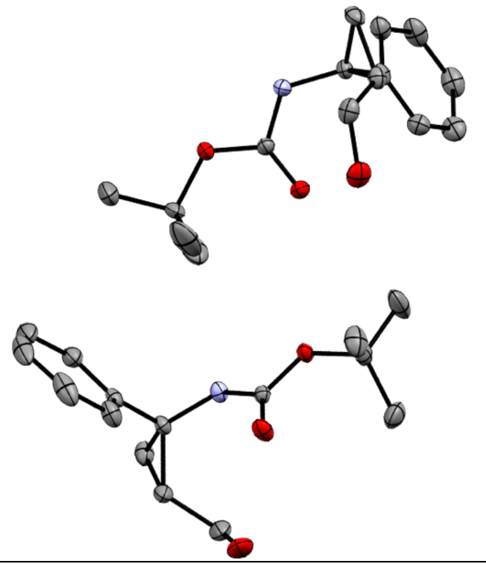

Empirical formula	$C_{15}H_{21}NO_3$
Formula weight	263.33
Temperature/K	120(2)
Crystal system	monoclinic
Space group	$P2_1/n$
a/Å	14.3663(6)
b/Å	12.2533(4)
c/Å	17.9300(9)
α/°	90
β/°	113.301(6)
γ/°	90
Volume/Å^3	2898.9(2)
Z	8
ρ_{calc}g/cm^3	1.207
μ/mm^{-1}	0.675
F(000)	1136.0
Crystal size/mm^3	0.163 × 0.134 × 0.057
Radiation	Cu Kα (λ = 1.54184)
2Θ range for data collection/°	8.996 to 144.444
Index ranges	$-17 \leq h \leq 17$, $-15 \leq k \leq 15$, $-21 \leq l \leq 21$
Reflections collected	41804
Independent reflections	5656 [R_{int} = 0.0761, R_{sigma} = 0.0340]
Data/restraints/parameters	5656/0/351
Goodness-of-fit on F^2	1.506
Final R indexes [I>=2σ (I)]	R_1 = 0.1391, wR_2 = 0.3365
Final R indexes [all data]	R_1 = 0.1461, wR_2 = 0.3464
Largest diff. peak/hole / e Å^{-3}	2.14/-0.39

11. References

(1) Klimczyk, S.; Misale, A.; Huang, X.; Maulide, N. Dimeric TADDOL Phosphoramidites in Asymmetric Catalysis: Domino Deracemization and Cyclopropanation of Sulfonium Ylides. *Angew. Chem. Int. Ed.* **2015**, *54*, 10365–10369. https://doi.org/10.1002/anie.201503851.

(2) Burgess, K.; Lim, D.; Ho, K.-K.; Ke, C.-Y. Asymmetric Syntheses of Protected Derivatives of Carnosadine and Its Stereoisomers as Conformationally Constrained Surrogates for Arginine. *J. Org. Chem.* **1994**, *59*, 2179–2185. https://doi.org/10.1021/jo00087a039.

(3) Turnbull, K. Sodium Azide. In *Encyclopedia of Reagents for Organic Synthesis*; John Wiley & Sons, Ltd: Chichester, 2008. https://doi.org/10.1002/047084289X.rs045.

(4) Hoshina, Y.; Doi, T.; Takahashi, T. Synthesis of the Octahydroindole Unit of Aeruginosins via Asymmetric Hydrogenation of the Diels–Alder Adducts of 2-Amido-2,4-Pentadienoate. *Tetrahedron* **2007**, *63*, 12740–12746. https://doi.org/10.1016/j.tet.2007.09.078.

(5) Thirumalai, R. S.; Eswariah, S.; Satyanarayana, R. Thirumalai, R. S.; Eswariah, S.; Satyanarayana, R. PROCESS FOR THE PREPARATION OF (±M1R(S), 2SRR)L-2-(AMINOMETHYL)-N,N-DIETHYL-L-PHENYLCYCLOPROPANE CARBOXAMIDE HYDROCHLORIDE. WO2012046247A2.

(6) Doyle, M. P.; Davies, S. B.; Hu, W. Dirhodium(II) Tetrakis[Methyl 2-Oxaazetidine-4-Carboxylate]: A Chiral Dirhodium(II) Carboxamidate of Exceptional Reactivity and Selectivity. *Org. Lett.* **2000**, *2*, 1145–1147. https://doi.org/10.1021/ol005730q.

(7) Podunavac, M.; Mailyan, A. K.; Jackson, J. J.; Lovy, A.; Farias, P.; Huerta, H.; Molgó, J.; Cardenas, C.; Zakarian, A. Scalable Total Synthesis, IP3R Inhibitory Activity of Desmethylxestospongin B, and Effect on Mitochondrial Function and Cancer Cell Survival. *Angew. Chem. Int. Ed.* **2021**, *60*, 11278–11282. https://doi.org/10.1002/anie.202102259.

(8) Minagawa, K.; Ansan, T.; Suzuki, N.; Ueda, T.; Fumoto, M.; Owada, T. CARNOSADINE LACTAM DERIVATIVE HAVING ANTIBACTERIAL ACTIVITY. JP2013079392W, 2016.

(9) Crane, Z. D.; Nichols, P. J.; Sammakia, T.; Stengel, P. J. Synthesis of Methyl-1-(*tert*-Butoxycarbonylamino)-2-Vinylcyclopropanecarboxylate via a Hofmann Rearrangement Utilizing Trichloroisocyanuric Acid as an Oxidant. *J. Org. Chem.* **2011**, *76*, 277–280. https://doi.org/10.1021/jo101504e.

(10) Inoue, M.; Sumii, Y.; Shibata, N. Contribution of Organofluorine Compounds to Pharmaceuticals. *ACS Omega* **2020**, *5*, 10633–10640. https://doi.org/10.1021/acsomega.0c00830.

12. NMR Spectra

(±)-**3** - ethyl (1*SR*,5*RS*) 2-oxo-3-oxabicyclo[3.1.0]hexane-1-carboxylate

^{1}H NMR (400 MHz, CDCl$_3$):

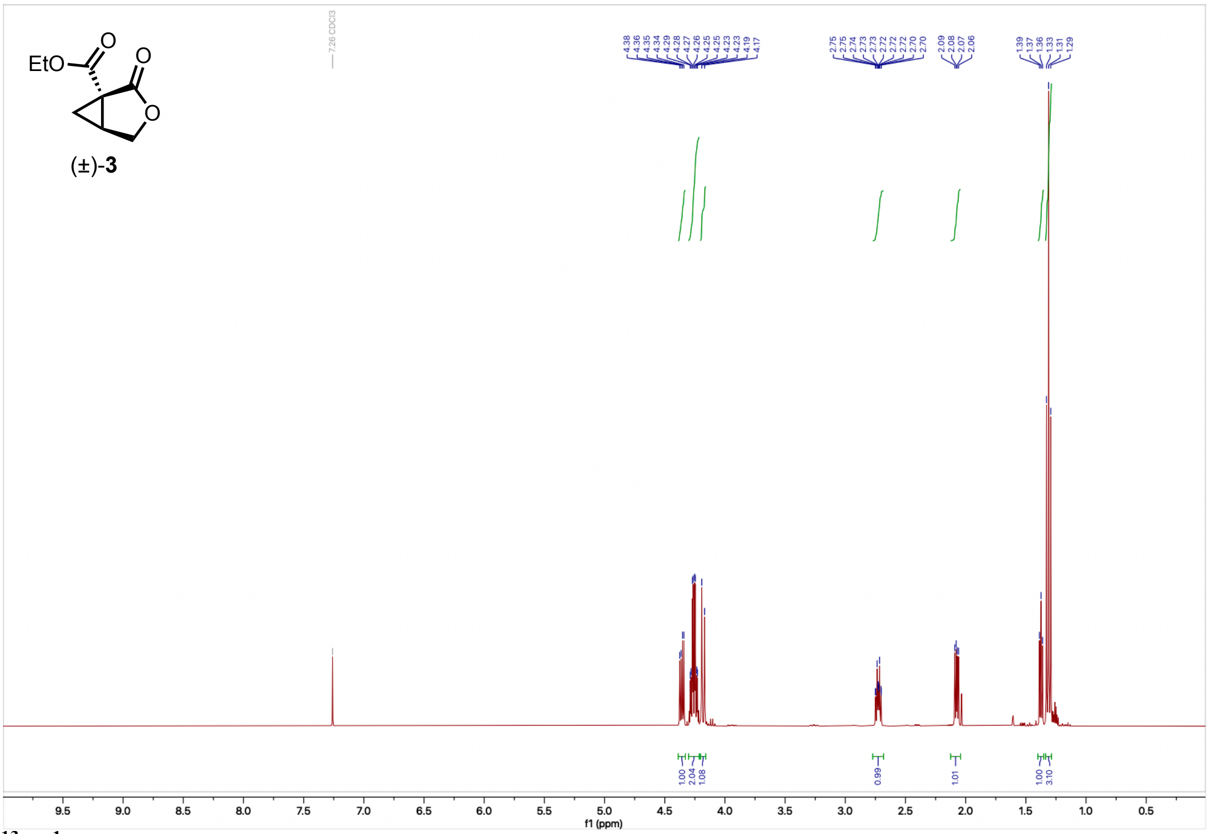

^{13}C{^{1}H} NMR (101 MHz, CDCl$_3$):

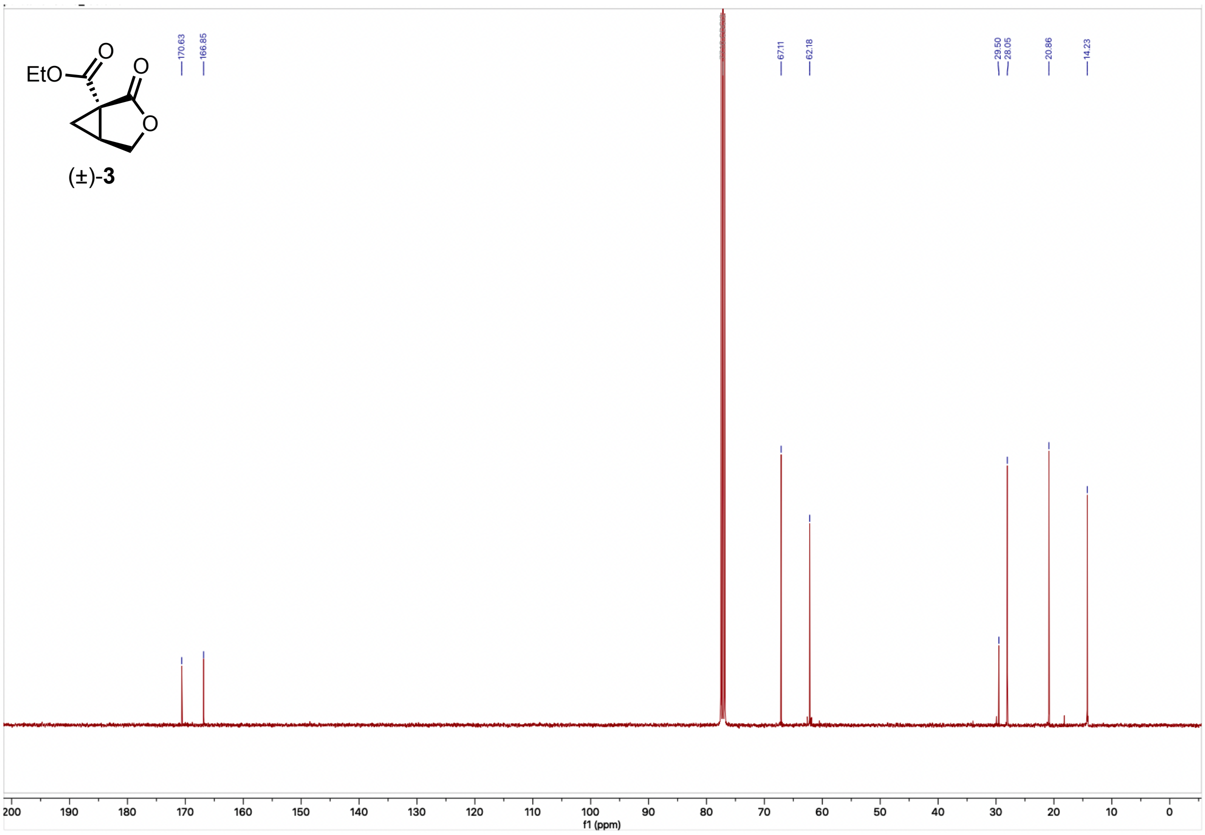

(±)-**4** - ethyl (1*RS*,2*RS*)-1-carbamoyl-2-(hydroxymethyl)cyclopropane-1-carboxylate

^{1}H NMR (400 MHz, CDCl$_3$):

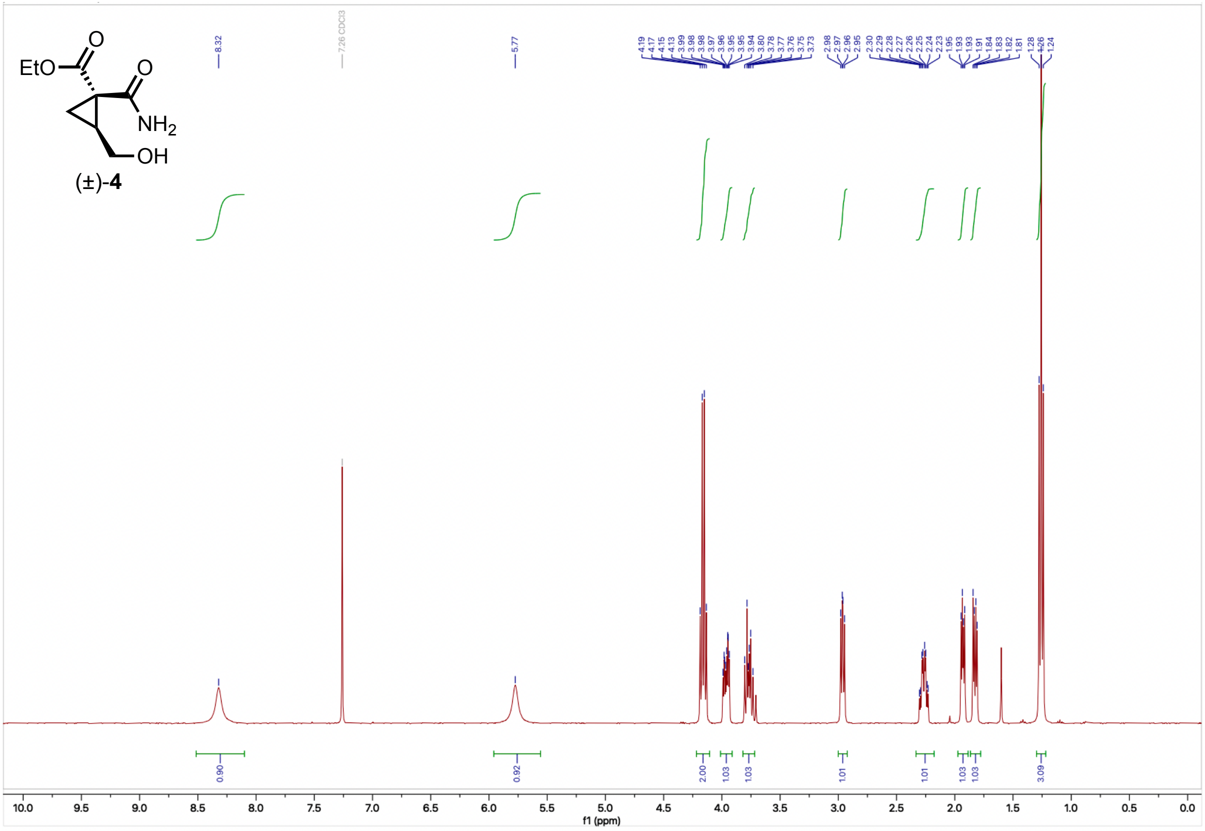

^{13}C{^{1}H} NMR (101 MHz, CDCl$_3$):

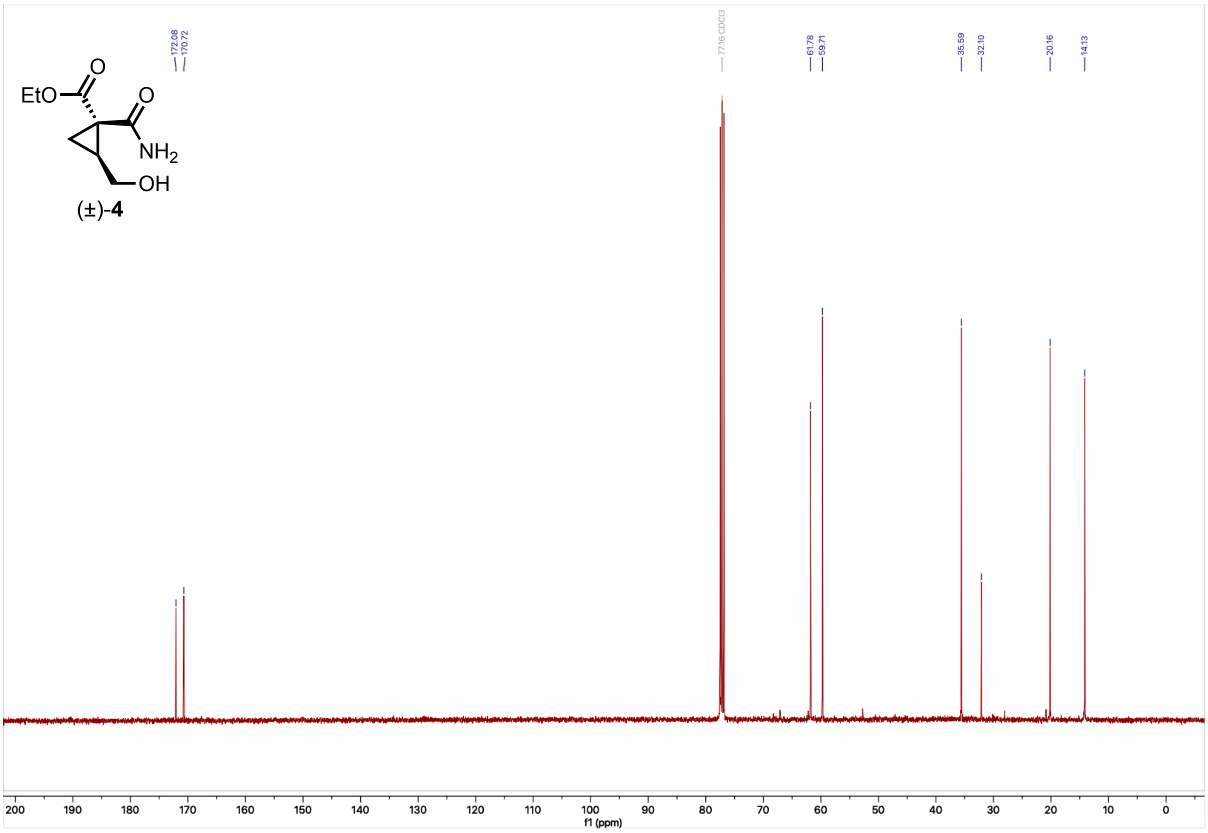

(±)-(*1RS,2RS*)-2-carbamoyl-2-(ethoxycarbonyl)cyclopropyl)methyl benzoate

^{1}H NMR (400 MHz, CDCl$_3$):

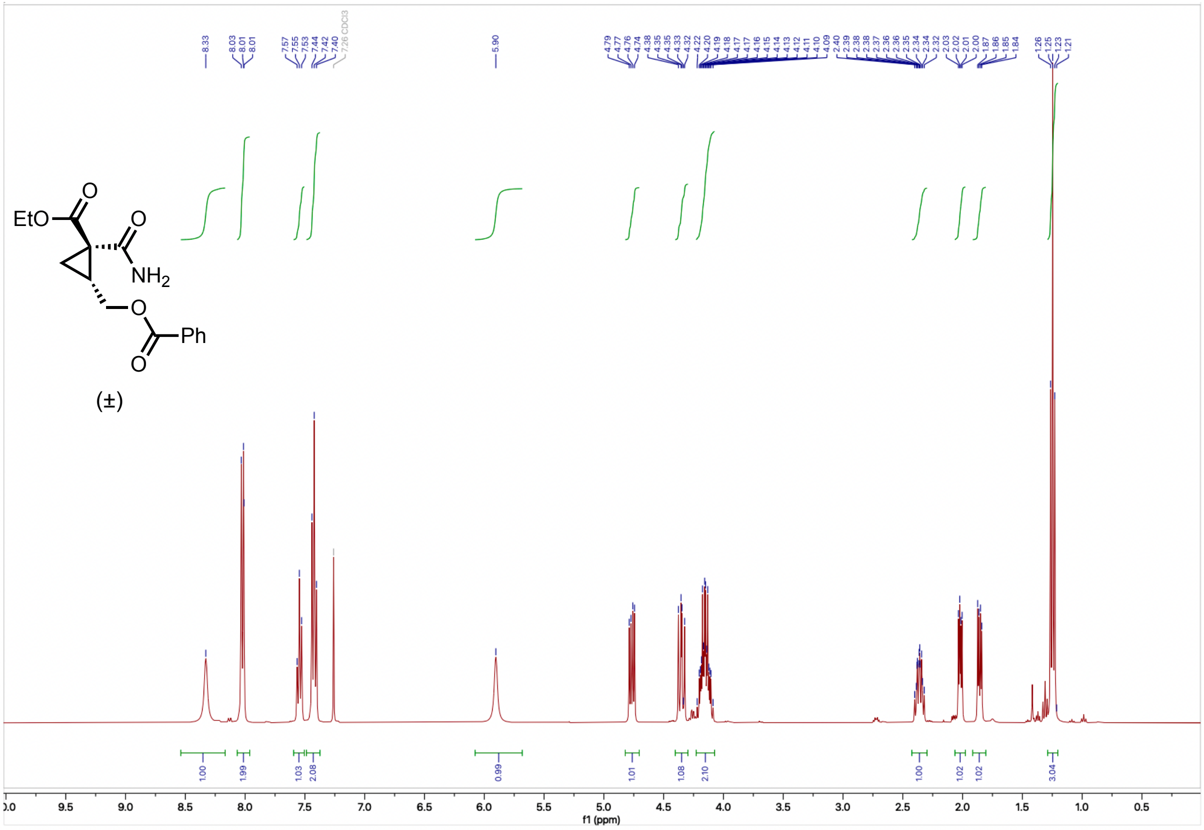

^{13}C{^{1}H} NMR (101 MHz, CDCl$_3$):

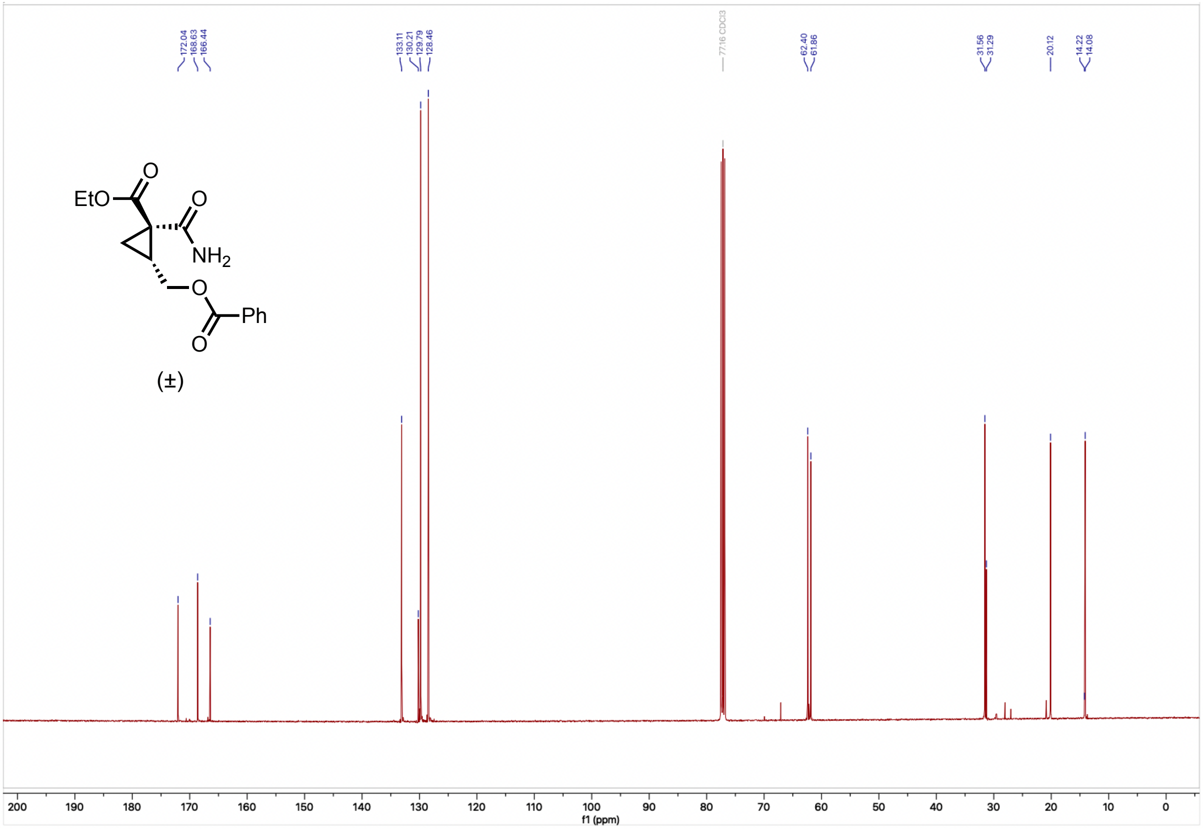

(±)-**5** - ethyl (1*SR*,6*RS*)-3-oxo-4-oxa-2-azabicyclo[4.1.0]heptane-1-carboxylate

^{1}H NMR (400 MHz, $CDCl_3$):

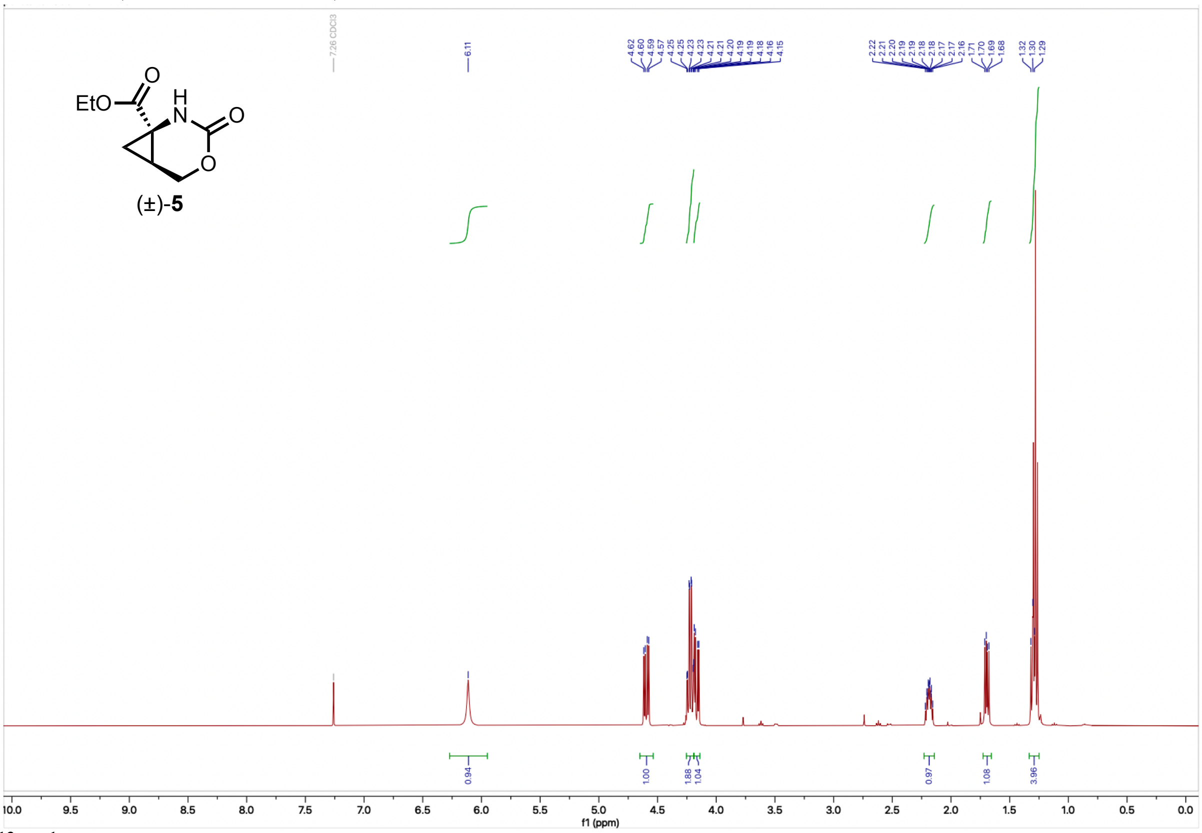

^{13}C{^{1}H} NMR (101 MHz, $CDCl_3$):

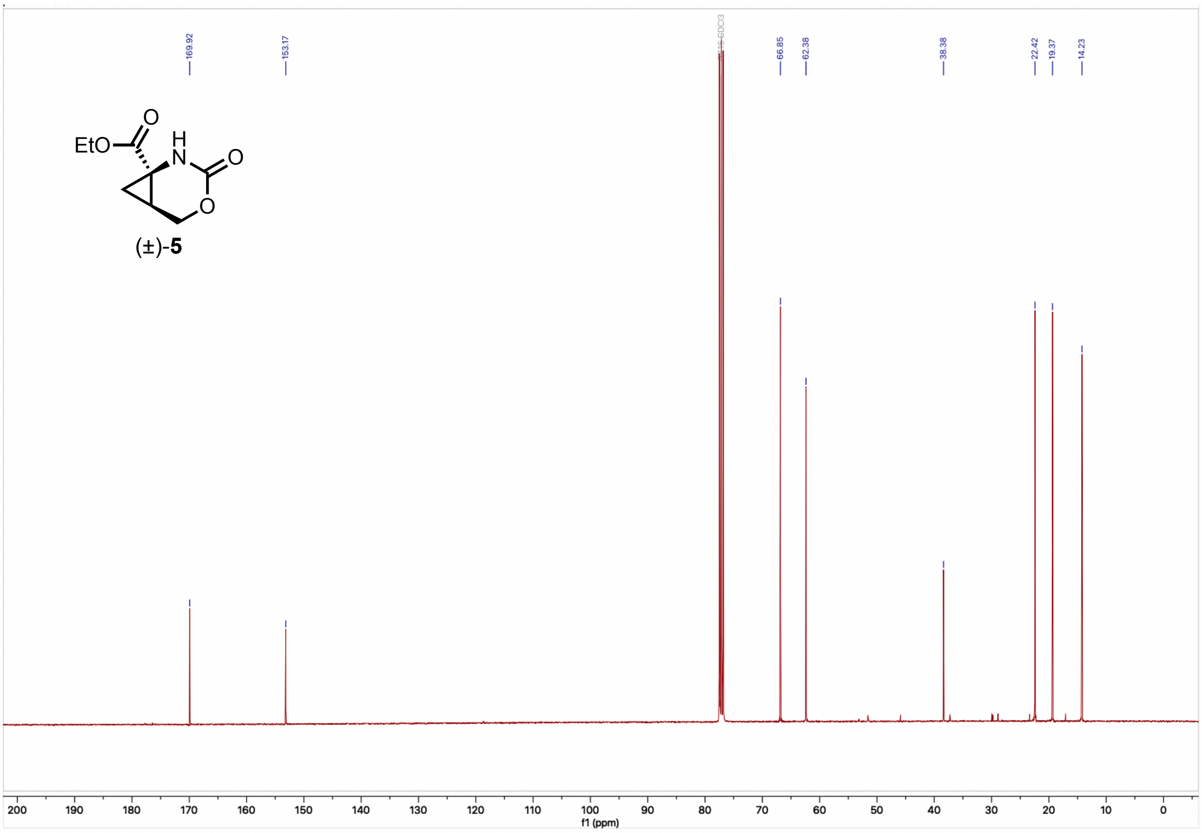

(±)-**6** - 2-(*tert*-butyl) 1-ethyl (1*SR*,6*RS*)-3-oxo-4-oxa-2-azabicyclo[4.1.0]heptane-1,2-dicarboxylate

^{1}H NMR (400 MHz, $CDCl_3$):

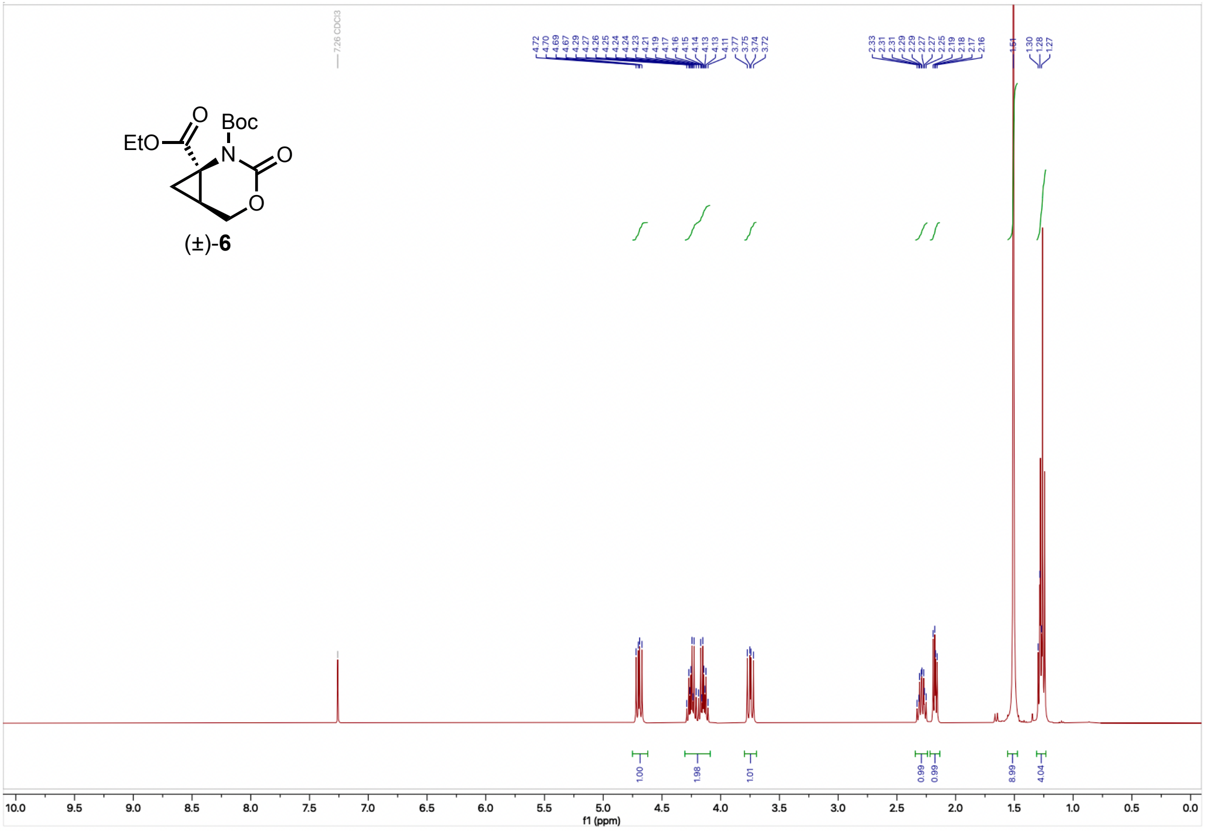

^{13}C{^{1}H} NMR (101 MHz, $CDCl_3$):

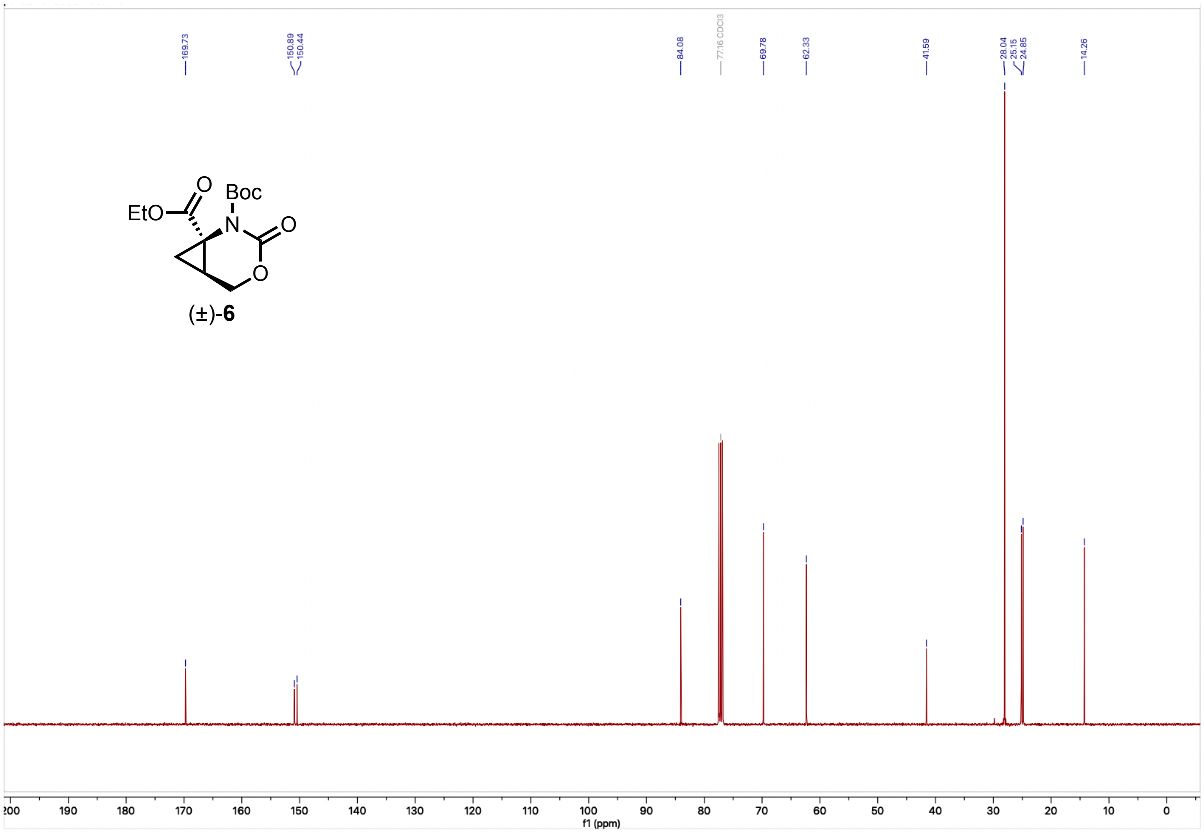

(±)-**7** - 2-((9*H*-fluoren-9-yl)methyl) 1-ethyl (1*RS*,6*SR*)-3-oxo-4-oxa-2-azabicyclo[4.1.0]heptane-1,2-dicarboxylate

^{1}H NMR (400 MHz, $CDCl_3$):

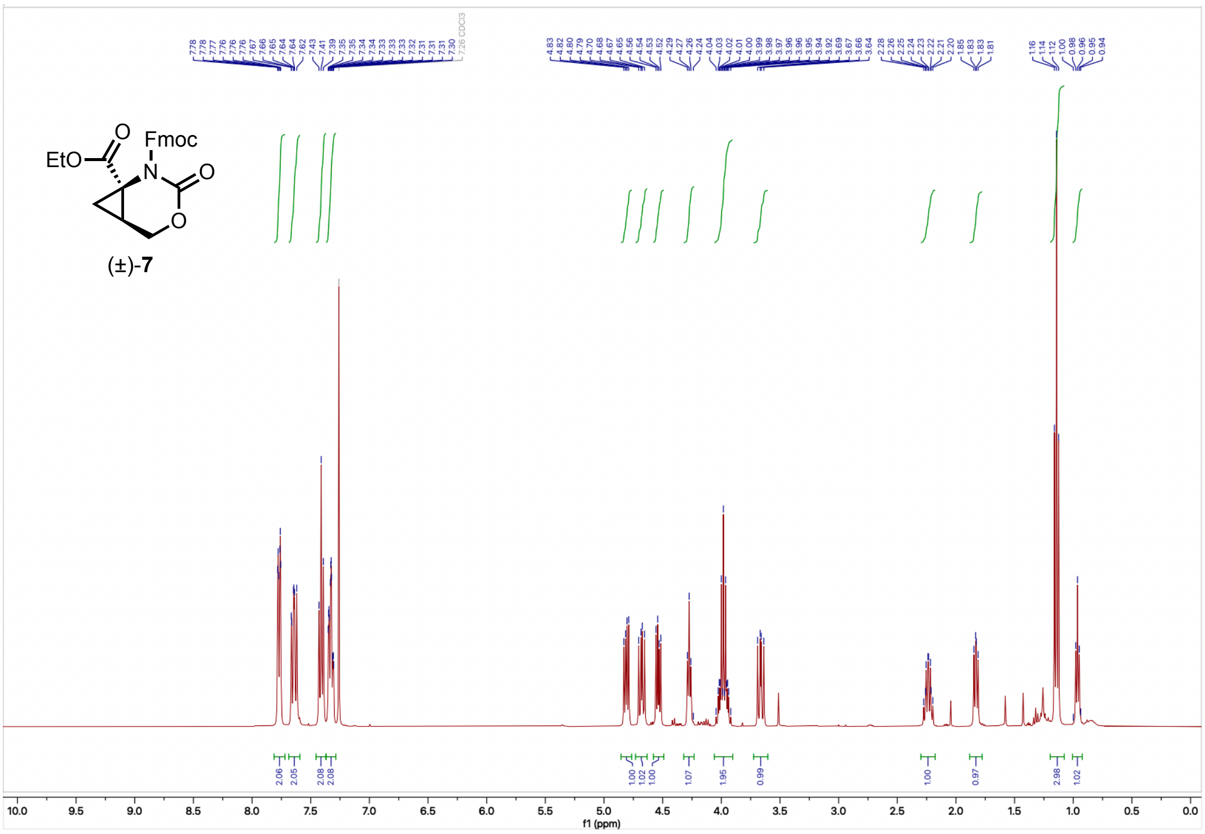

^{13}C{^{1}H} NMR (101 MHz, $CDCl_3$, rotamers present):

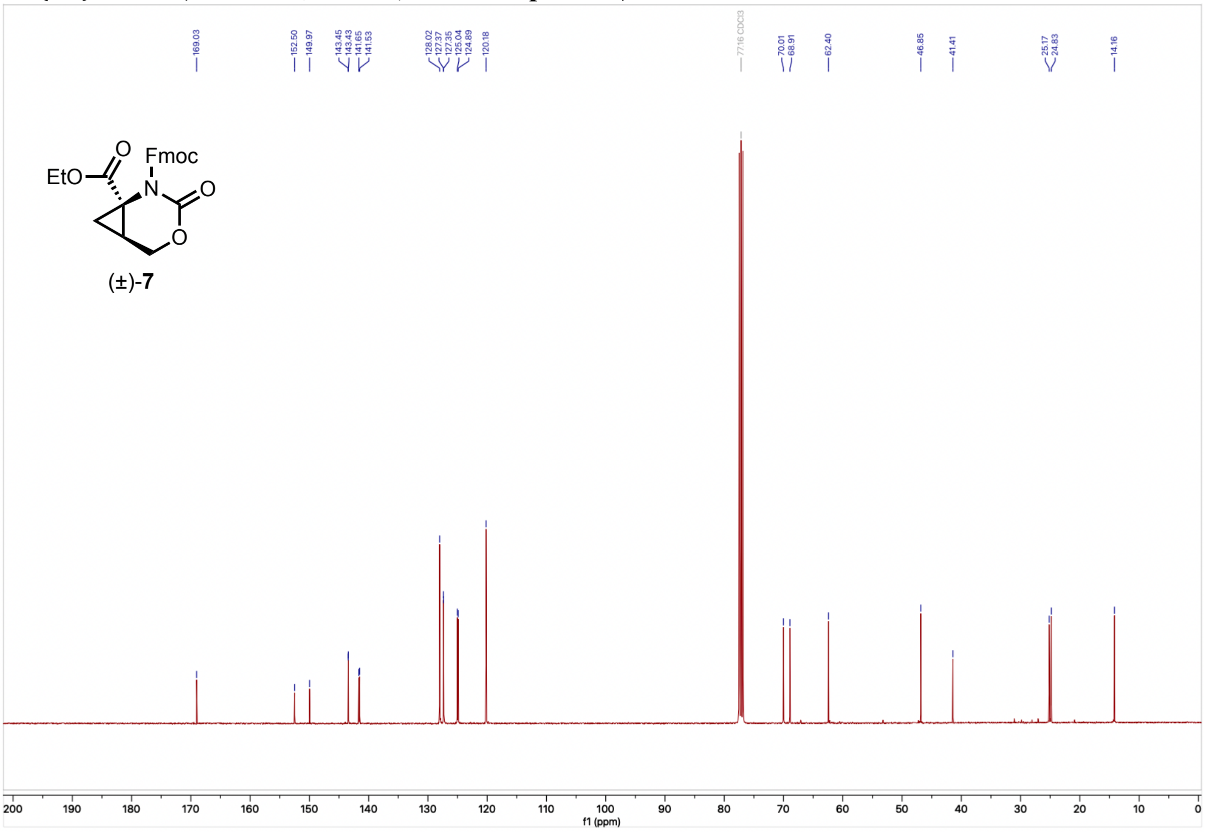

(±)-**8** - ethyl (1*RS*,6*SR*)-2-acetyl-3-oxo-4-oxa-2-azabicyclo[4.1.0]heptane-1-carboxylate

^{1}H NMR (400 MHz, CDCl$_3$):

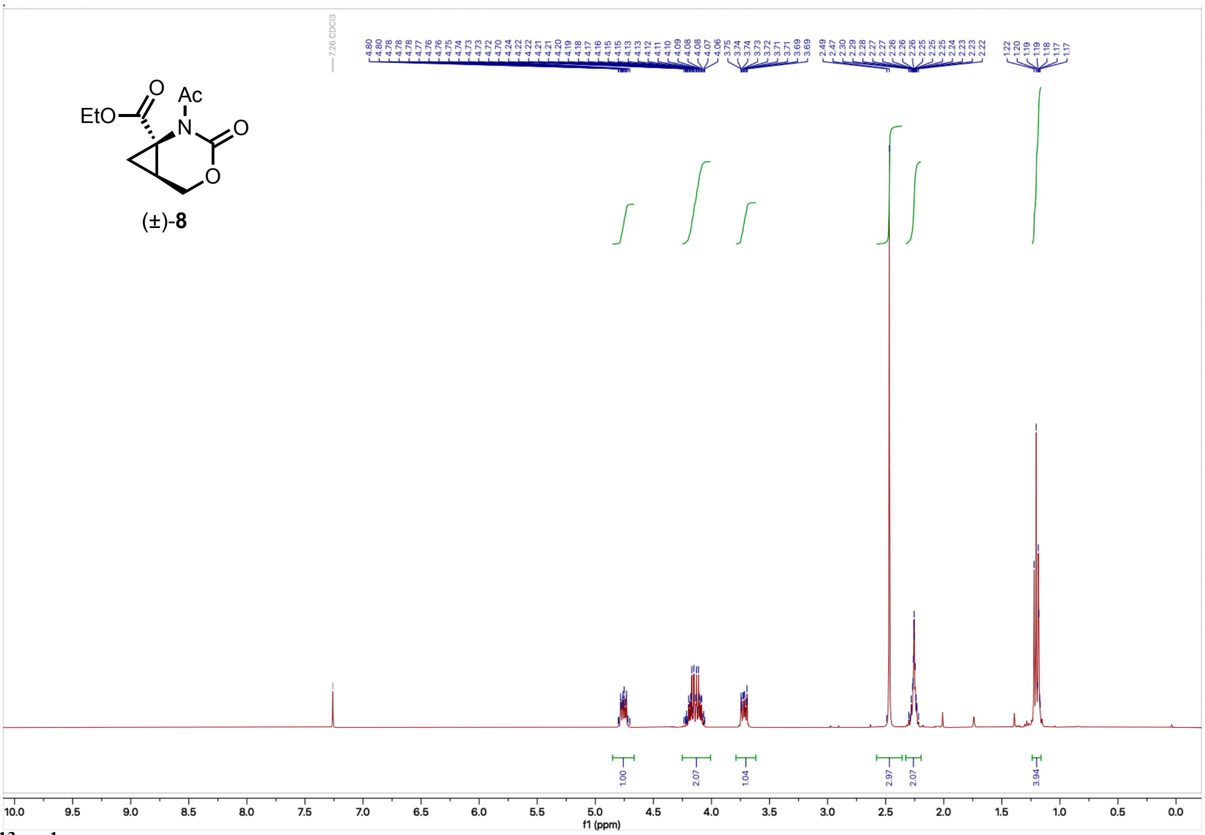

^{13}C{^{1}H} NMR (101 MHz, CDCl$_3$):

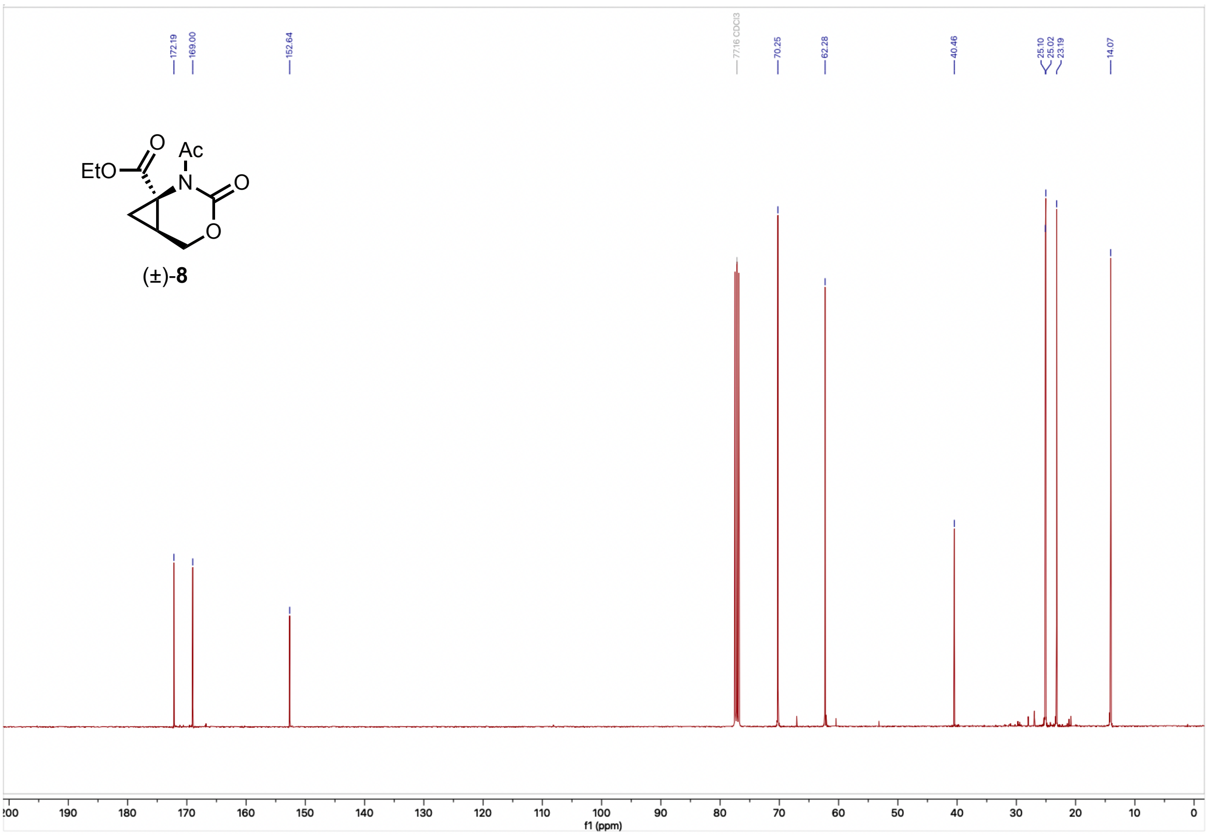

(±)-**9** - 1-ethyl 2-(4-nitrobenzyl) (1*RS*,6*SR*)-3-oxo-4-oxa-2-azabicyclo[4.1.0]heptane-1,2-dicarboxylate

^{1}H NMR (400 MHz, CDCl$_3$):

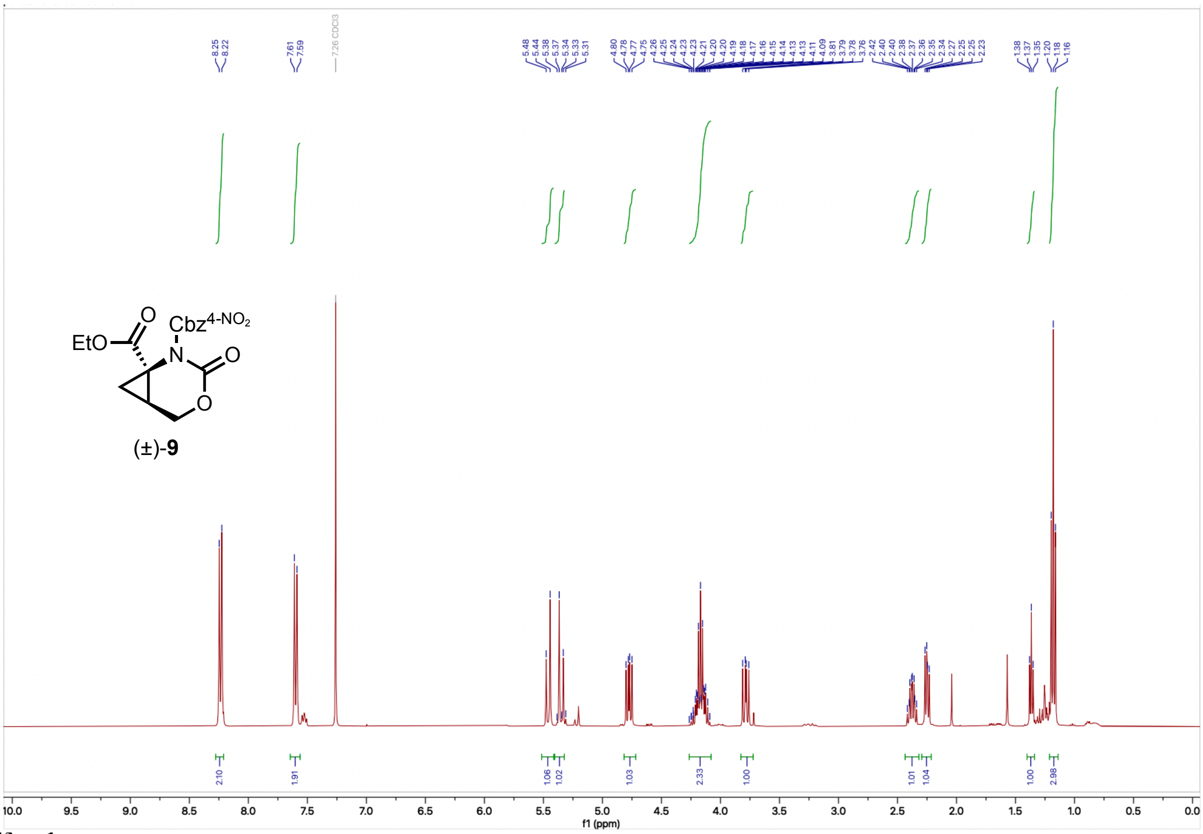

^{13}C{^{1}H} NMR (101 MHz, CDCl$_3$):

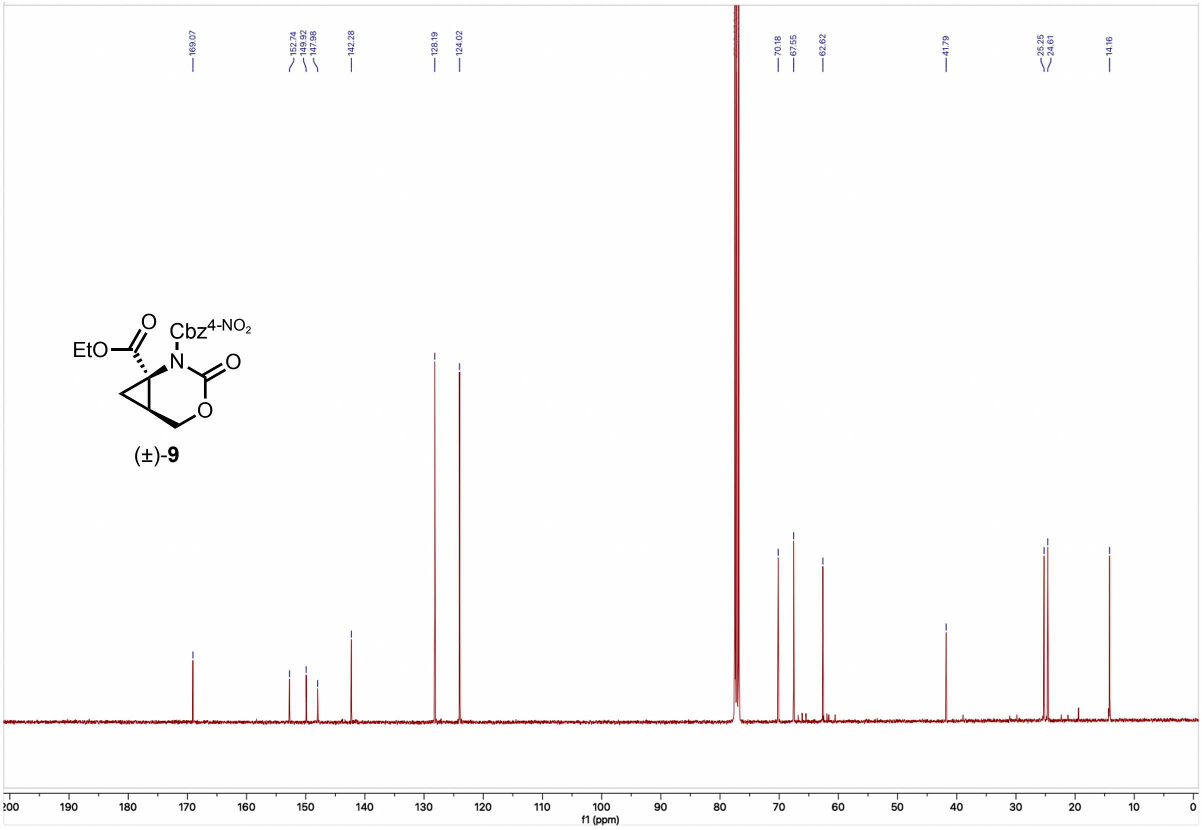

(±)-**11** - ethyl (1*RS*,2*SR*)-2-(bromomethyl)-1-((*tert*-butoxycarbonyl)amino)cyclopropane-1-carboxylate

^{1}H NMR (400 MHz, CDCl$_3$):

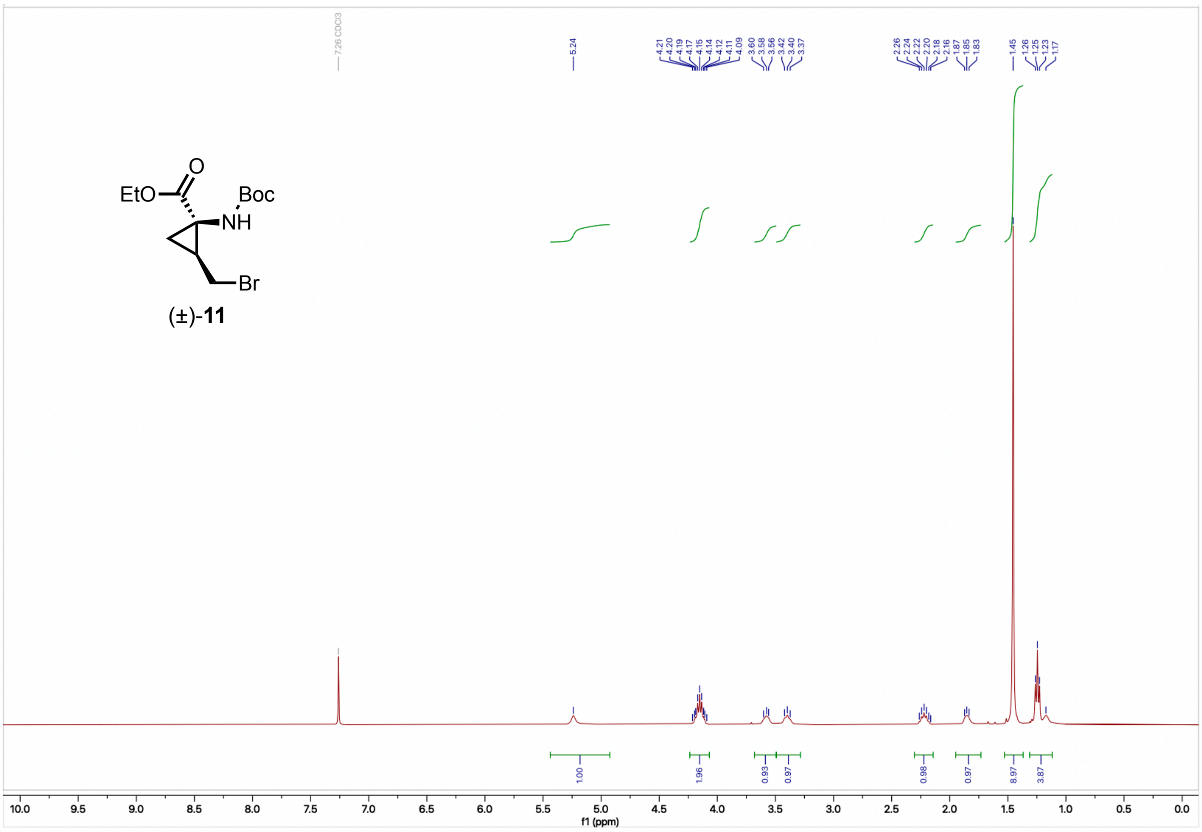

^{13}C{^{1}H} NMR (101 MHz, CDCl$_3$):

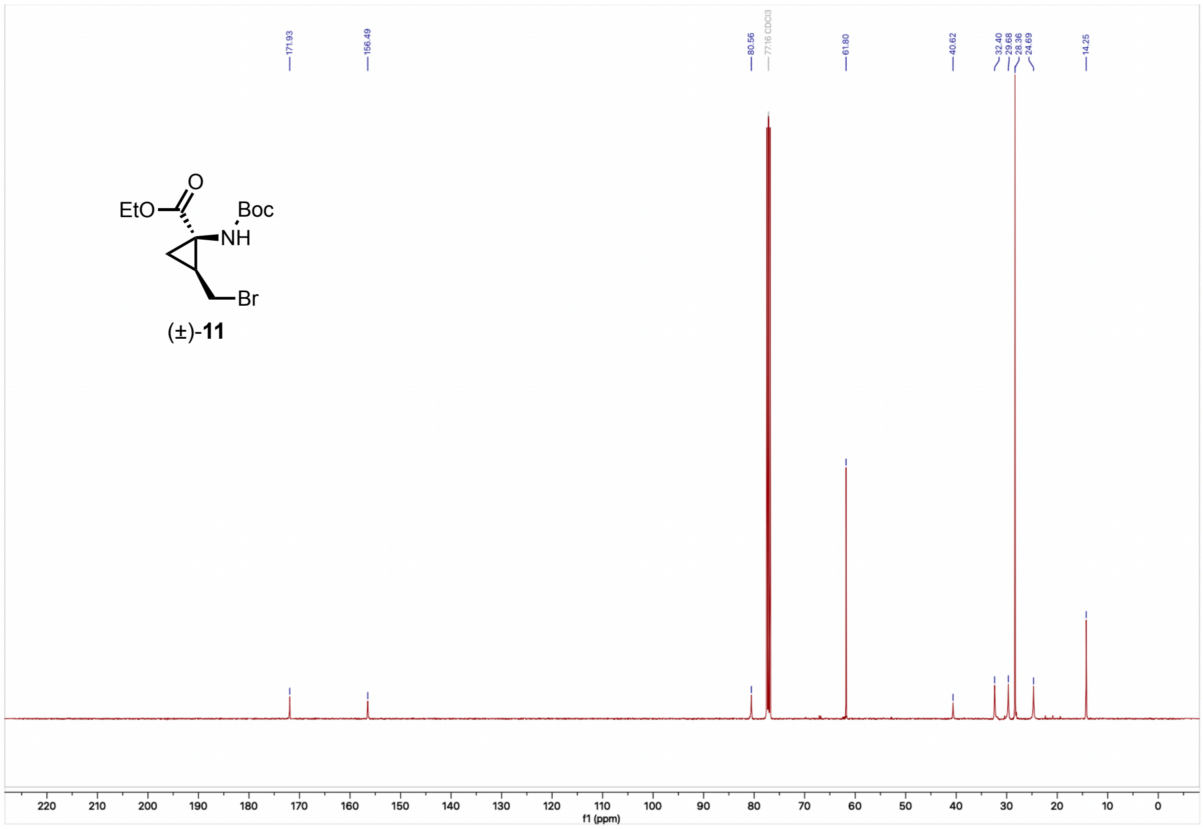

(±)-**12** - ethyl (1*RS*,2*SR*)-1-((((9*H*-fluoren-9-yl)methoxy)carbonyl)amino)-2-(bromomethyl)cyclopropane-1-carboxylate

^{1}H NMR (400 MHz, CDCl$_3$, rotamers present):

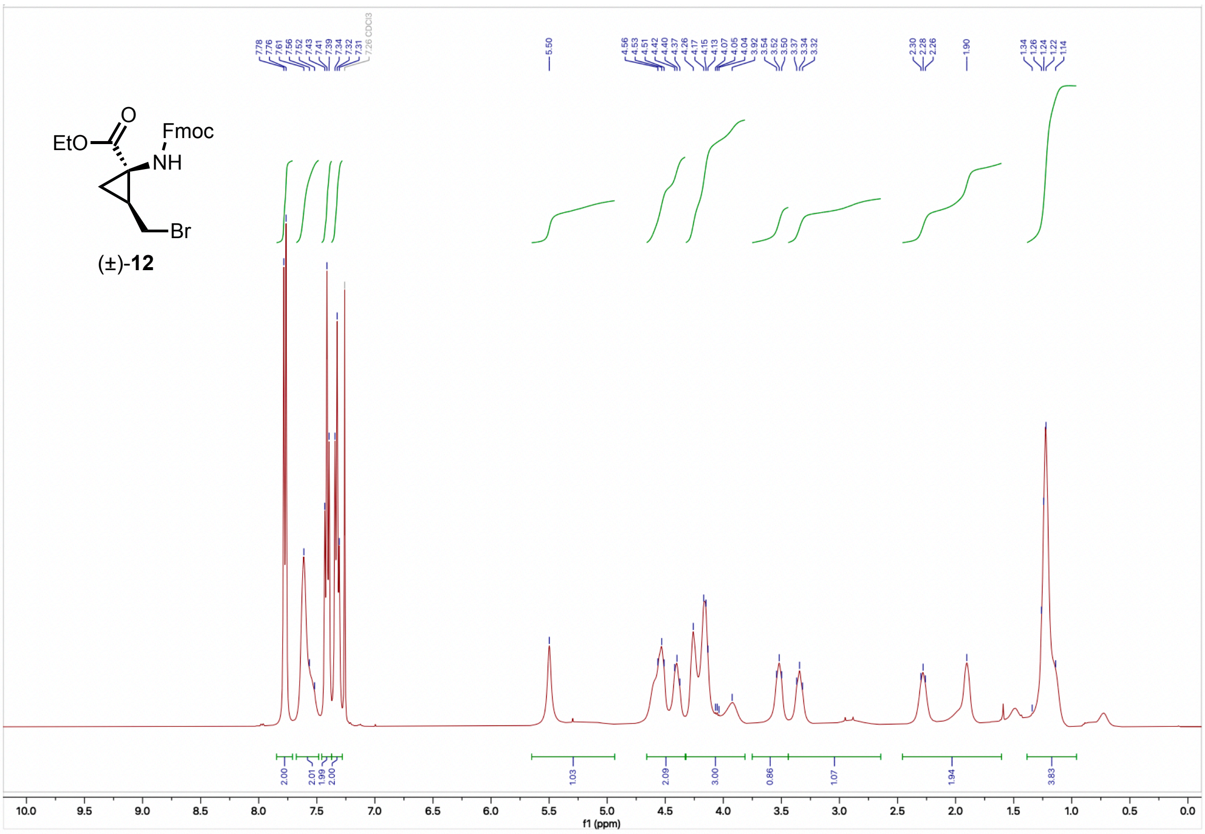

^{13}C{^{1}H} NMR (101 MHz, CDCl$_3$, rotamers present):

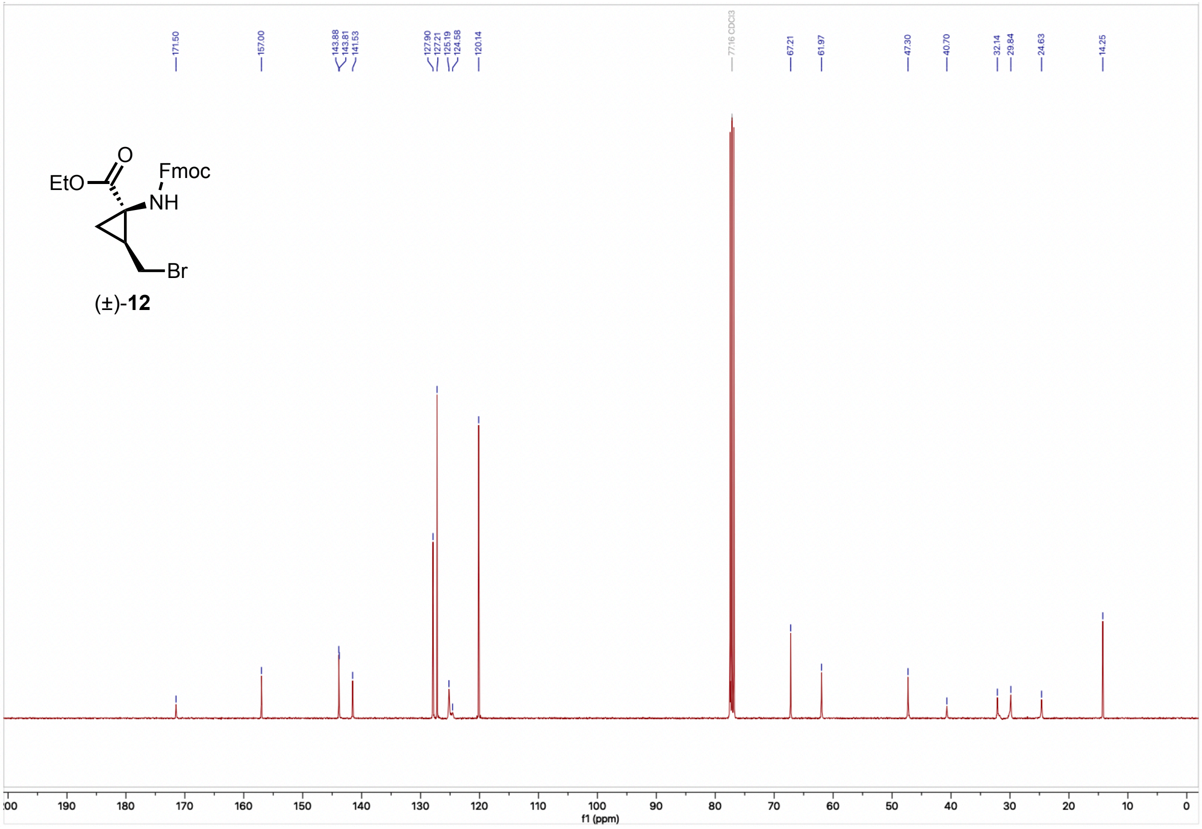

(±)-**14** - ethyl (1*RS*,2*SR*)-2-(bromomethyl)-1-((((4-nitrobenzyl)oxy)carbonyl)amino)cyclopropane-1-carboxylate

^{1}H NMR (400 MHz, $CDCl_3$):

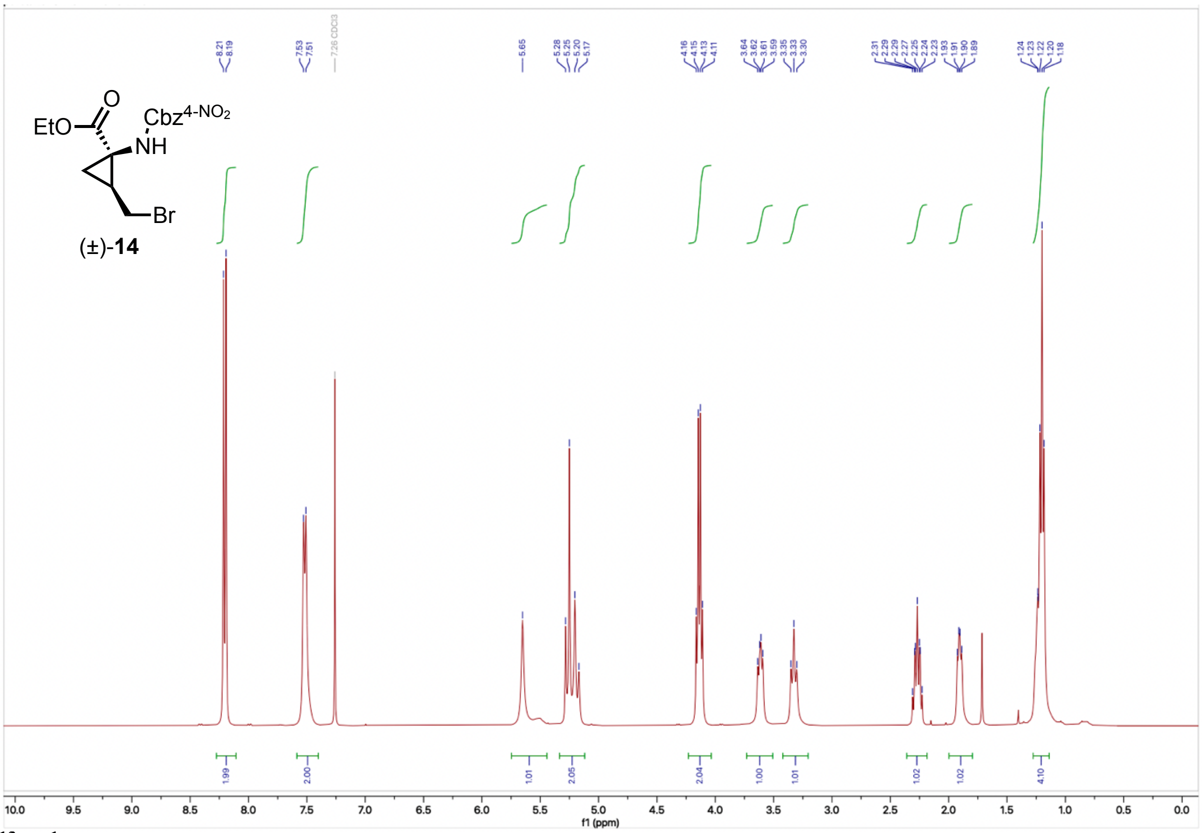

^{13}C{^{1}H} NMR (101 MHz, $CDCl_3$):

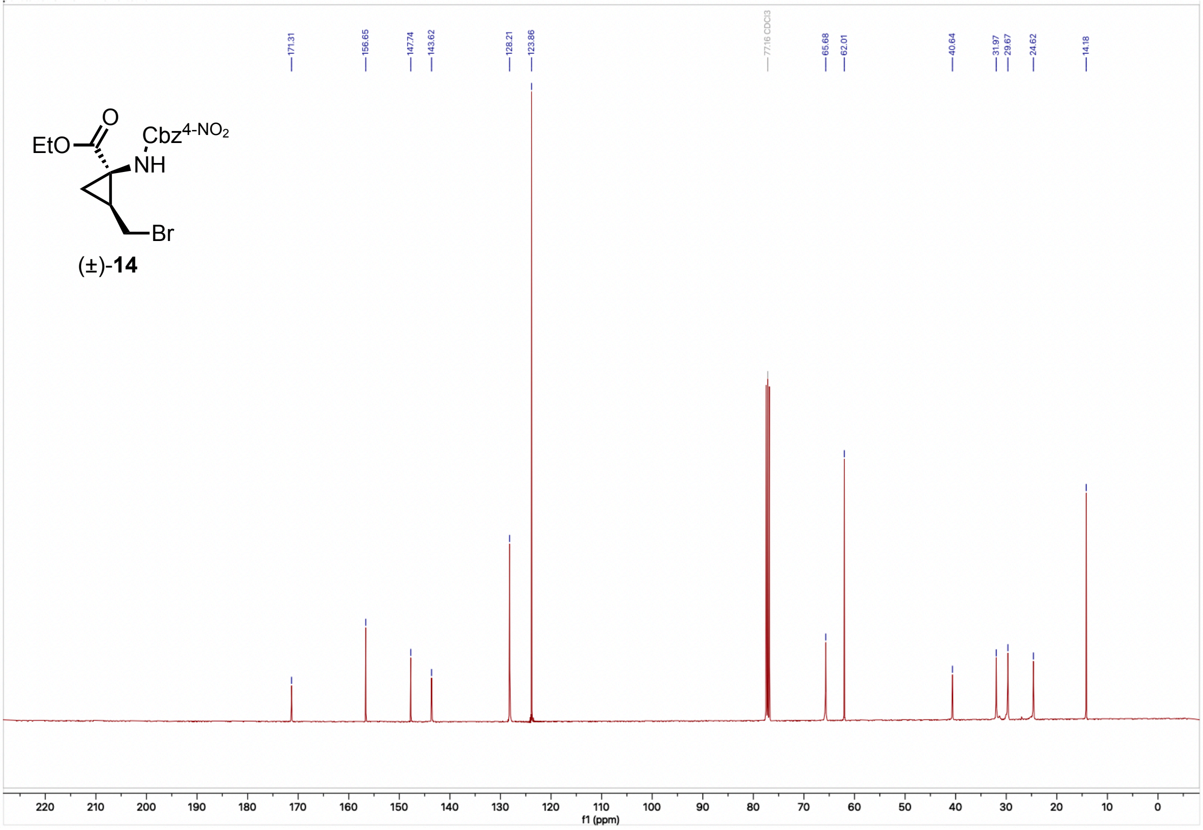

(±)-**15** - ethyl (1*SR*,2*RS*)-1-((*tert*-butoxycarbonyl)amino)-2-(hydroxymethyl)cyclopropane-1-carboxylate

^{1}H NMR (400 MHz, CDCl$_3$):

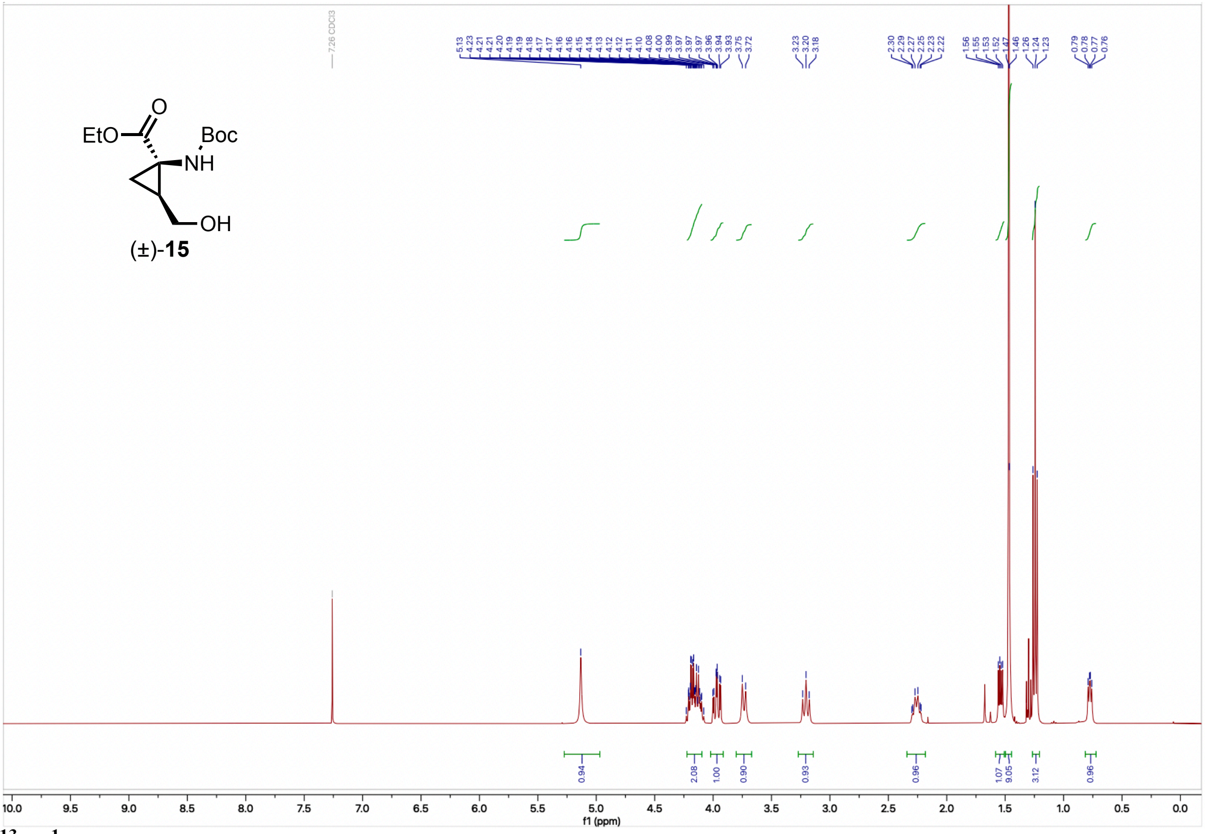

^{13}C{^{1}H} NMR (101 MHz, CDCl$_3$):

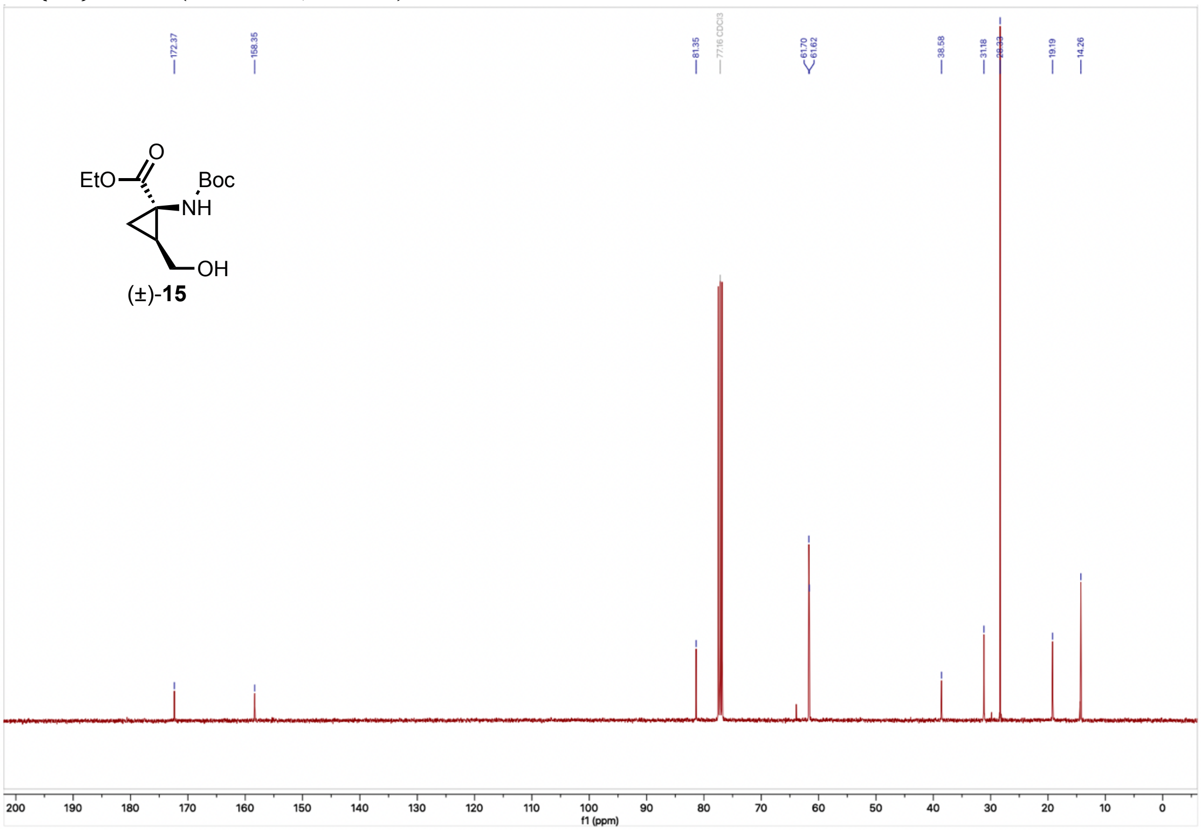

(±)-**17** - ethyl (1*RS*,2*SR*)-1-acetamido-2-(hydroxymethyl)cyclopropane-1-carboxylate

^{1}H NMR (400 MHz, CDCl$_3$):

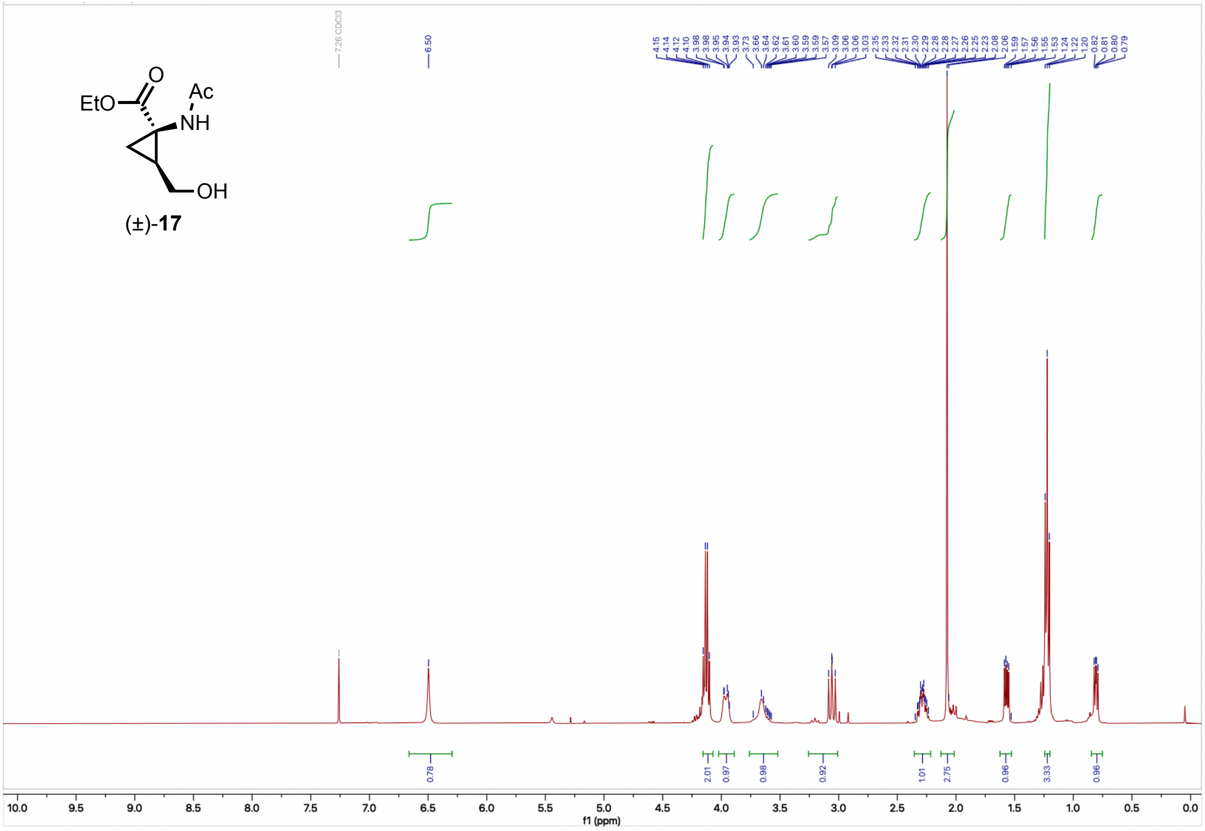

^{13}C{^{1}H} NMR (101 MHz, CDCl$_3$):

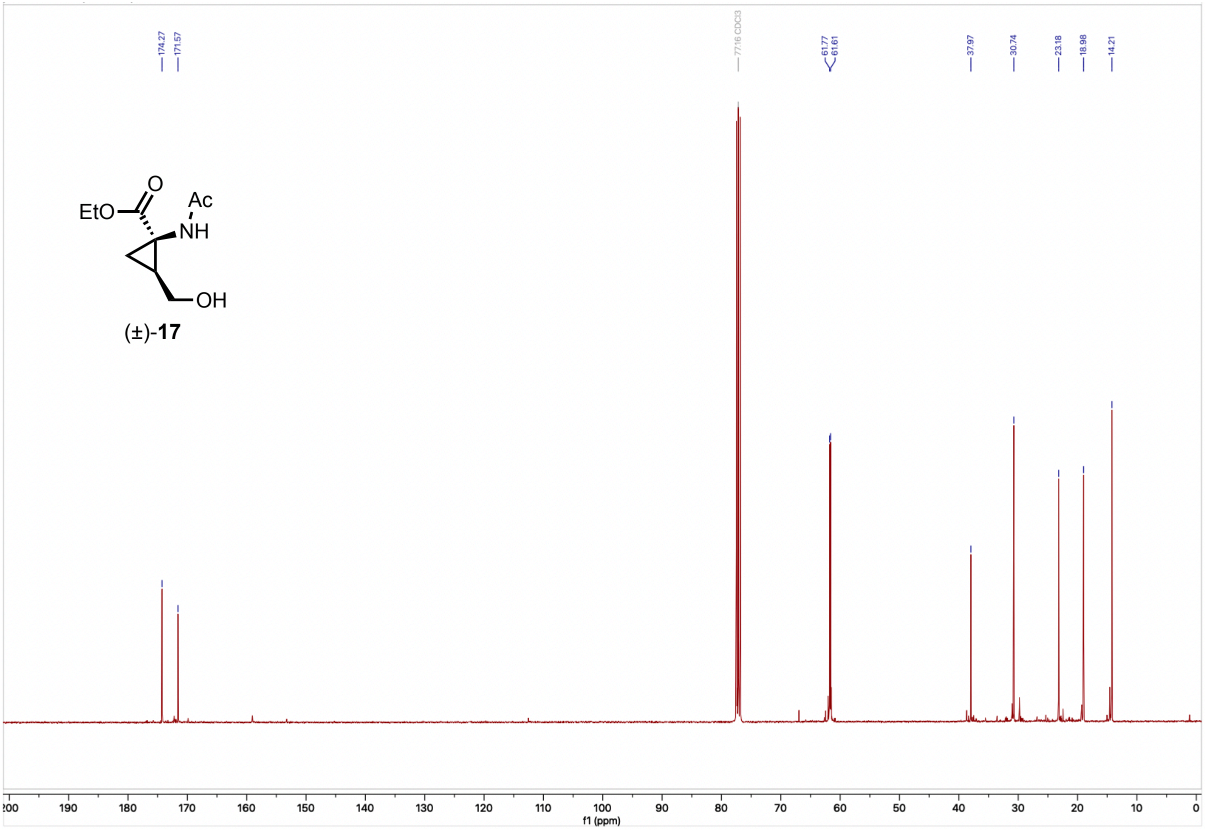

(±)-**19** - (1*SR*,2*RS*)-2-((*tert*-butoxycarbonyl)amino)-2-(ethoxycarbonyl)cyclopropane-1-carboxylic acid

^{1}H NMR (500 MHz, $CDCl_3$, rotamers observed):

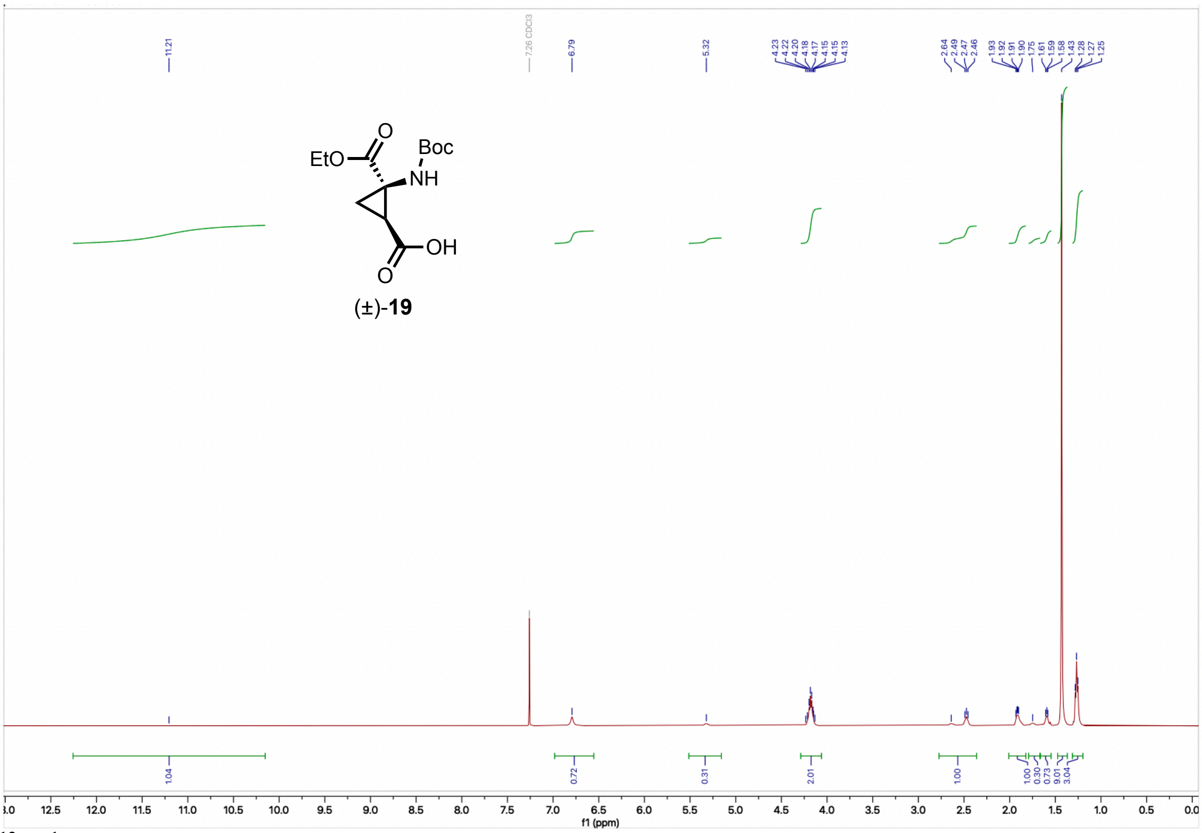

^{13}C{^{1}H} NMR (126 MHz, $CDCl_3$):

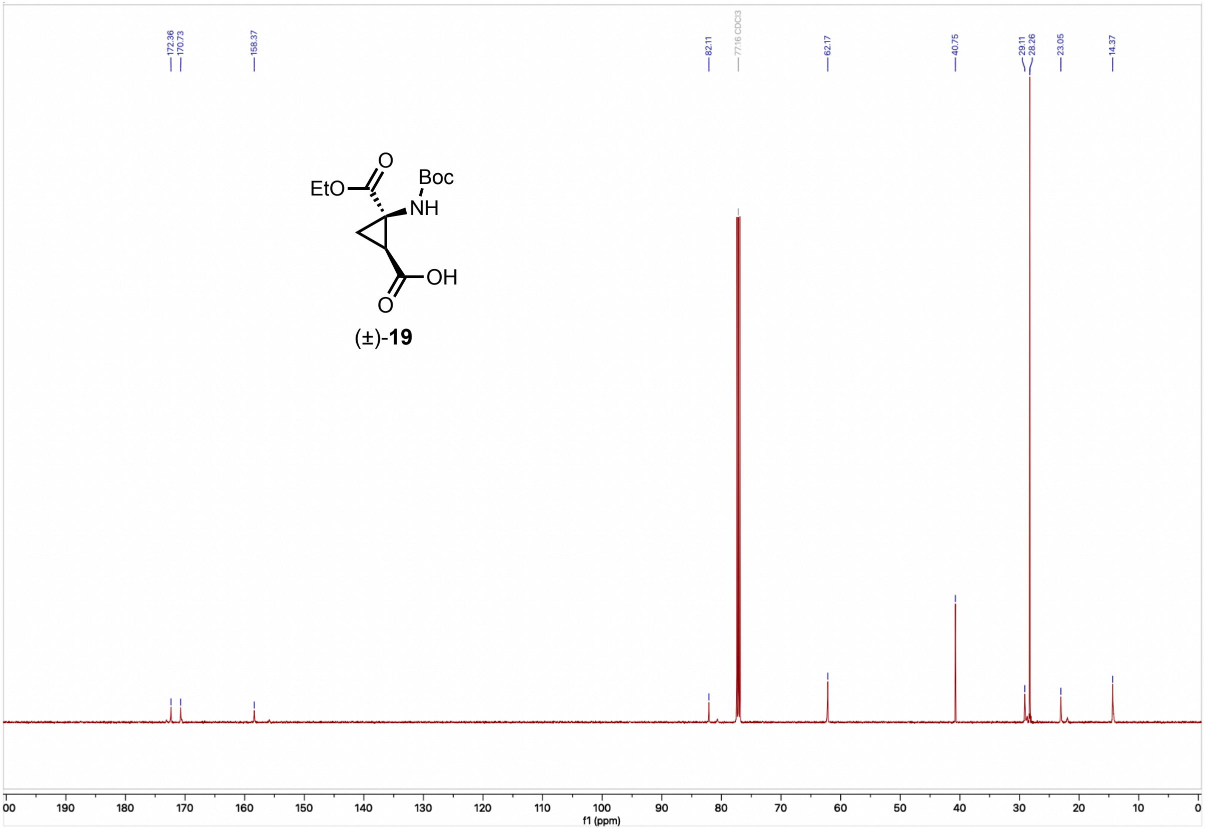

(±)-**20** - ethyl (1*RS*,2*SR*)-1-((*tert*-butoxycarbonyl)amino)-2-formylcyclopropane-1-carboxylate

^{1}H NMR (500 MHz, CDCl$_3$, rotamers observed):

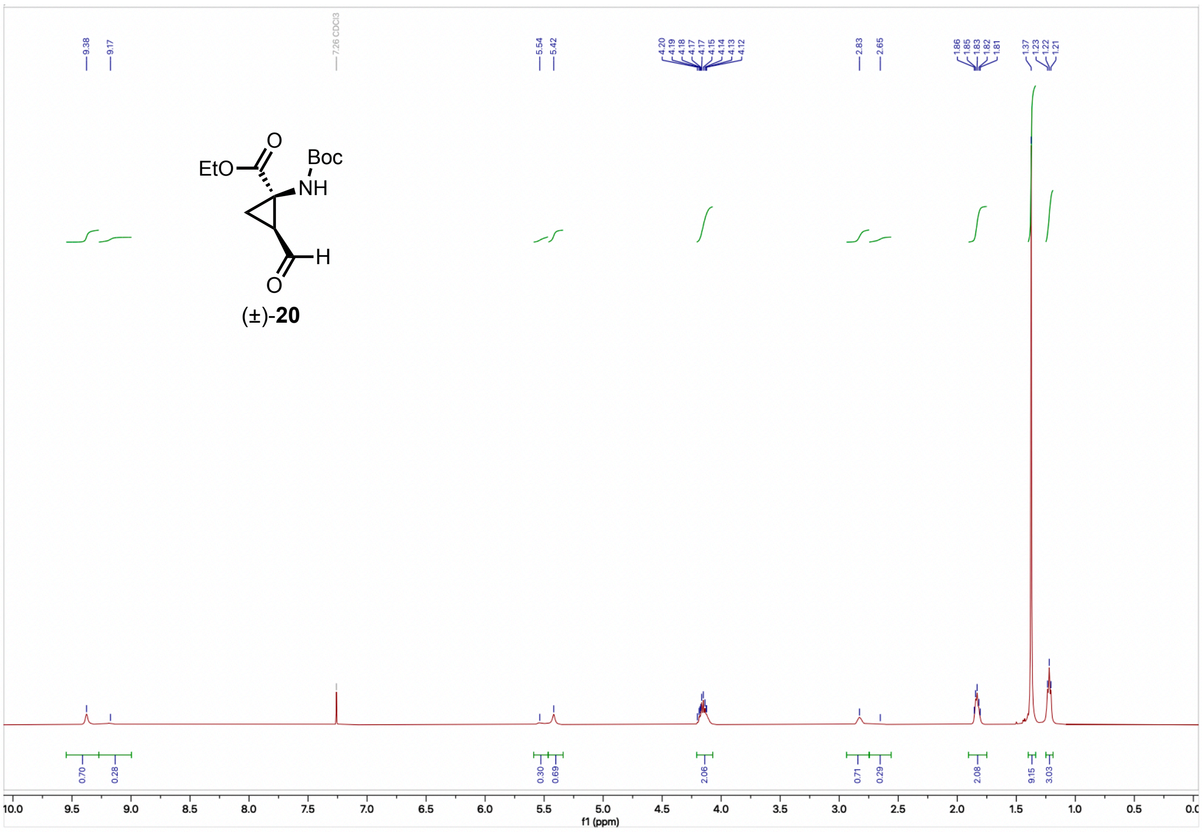

^{13}C{^{1}H} NMR (126 MHz, CDCl$_3$, rotamers observed):

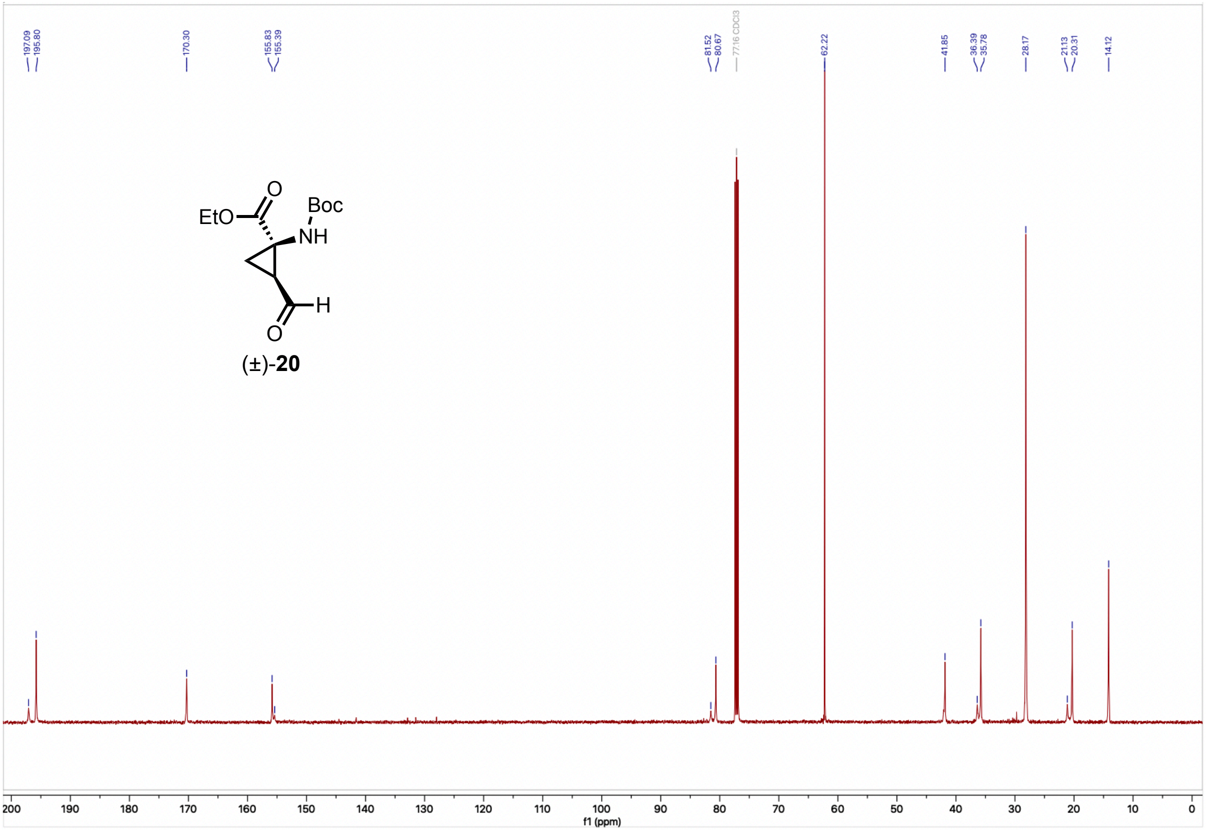

(±)-**21** - ethyl (1*RS*,2*RS*)-1-((*tert*-butoxycarbonyl)amino)-2-((dimethylamino)methyl)cyclopropane-1-carboxylate

^{1}H NMR (400 MHz, CDCl$_3$):

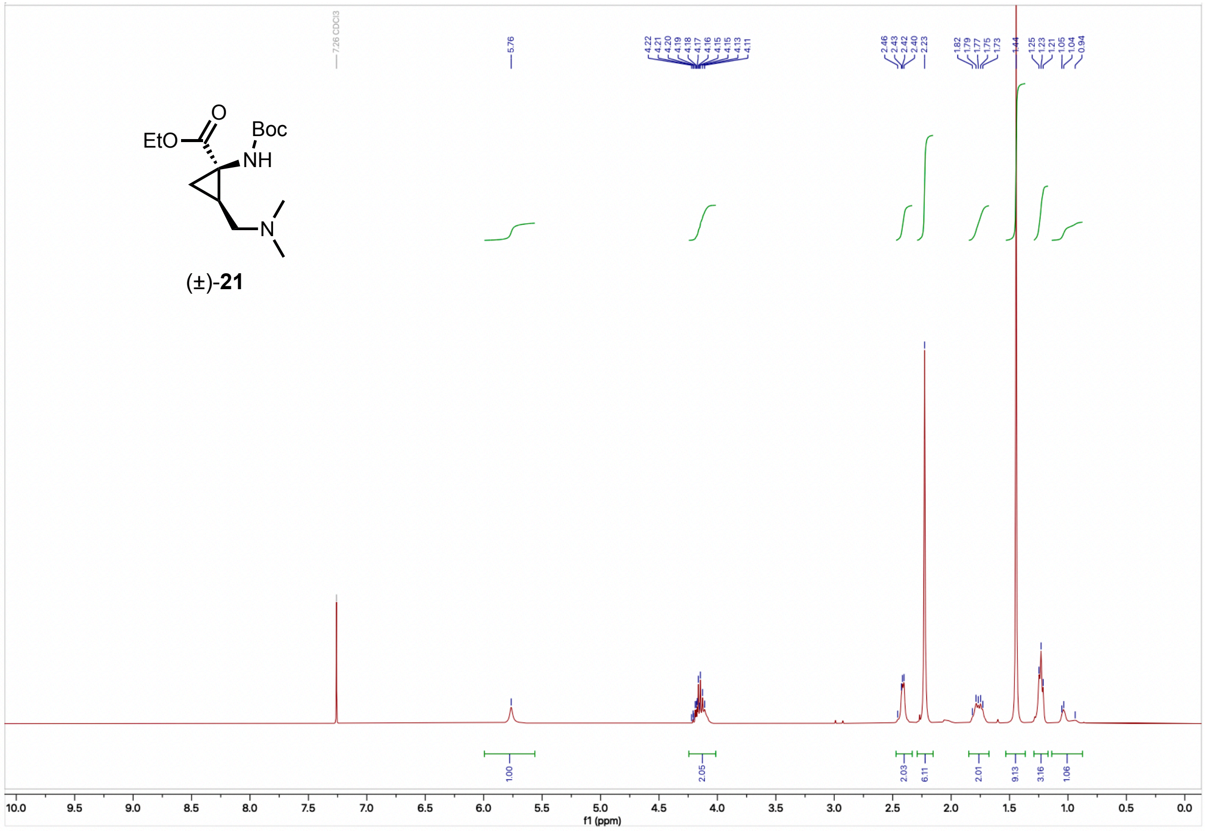

^{13}C{^{1}H} NMR (101 MHz, CDCl$_3$):

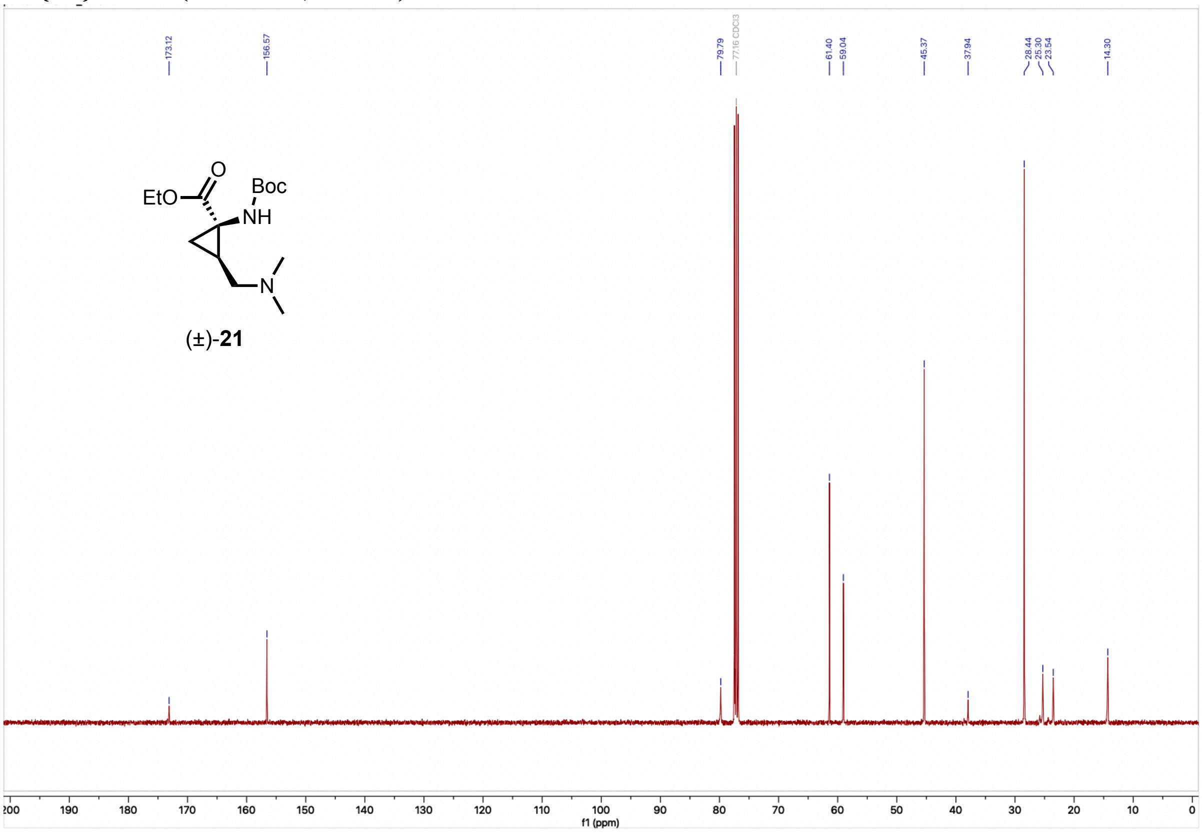

(±)-**22** - ethyl (1*RS*,2*RS*)-2-(aminomethyl)-1-((*tert*-butoxycarbonyl)amino)cyclopropane-1-carboxylate

^{1}H NMR (400 MHz, $CDCl_3$):

^{13}C{^{1}H} NMR (101 MHz, $CDCl_3$):

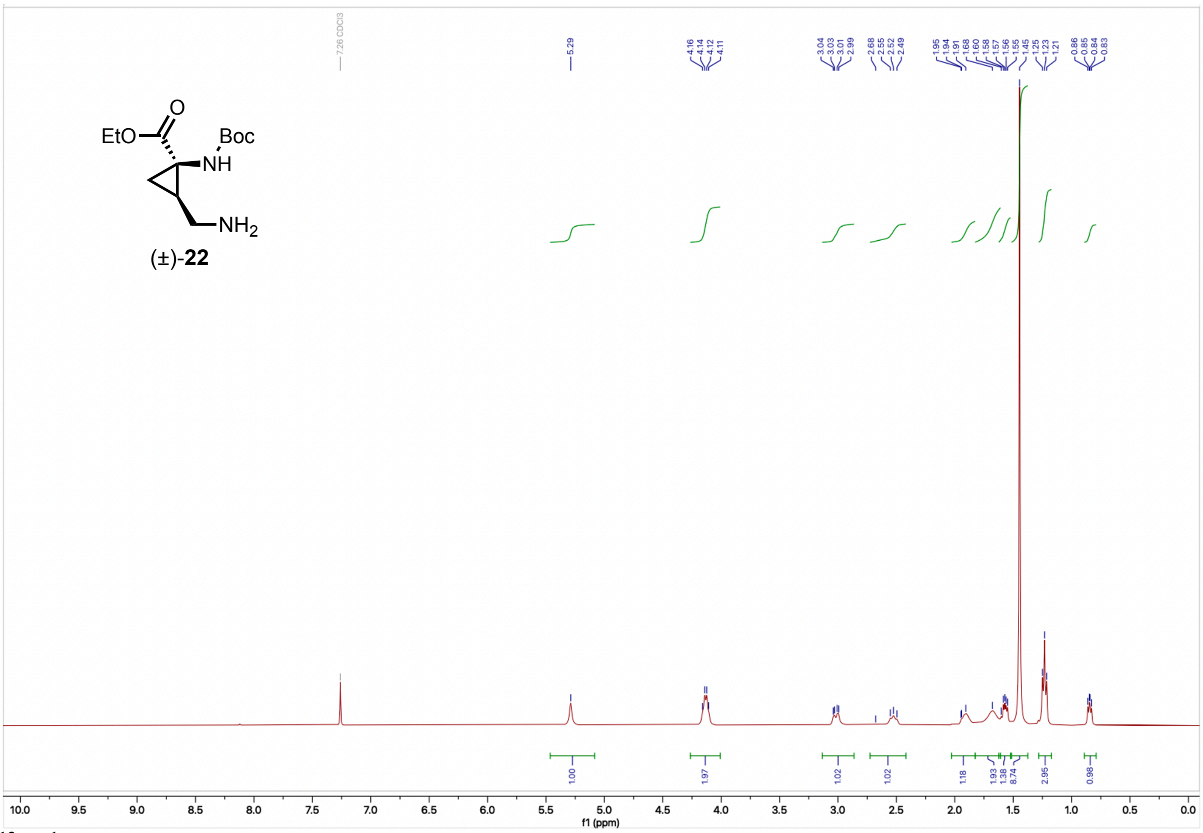

^{13}C{^{1}H} NMR (101 MHz, $CDCl_3$):

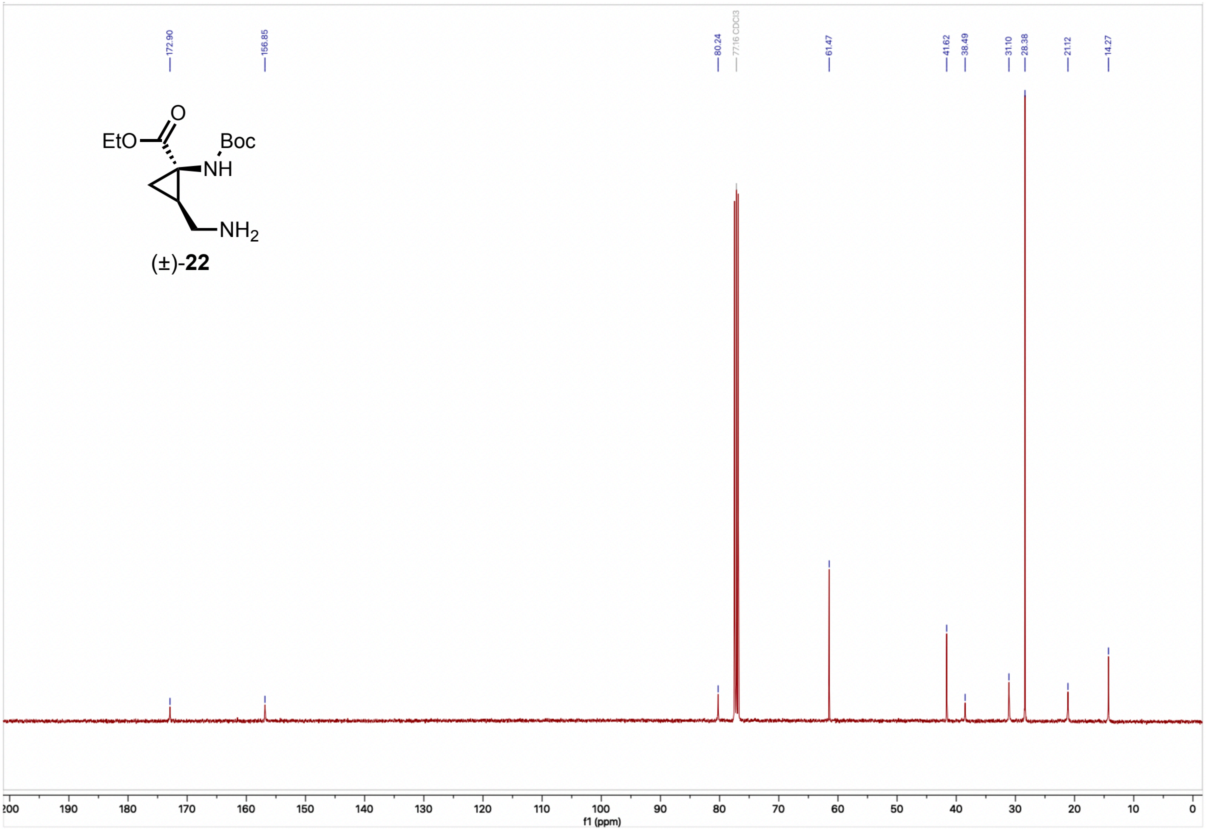

(±)-**23** - ethyl (1*RS*,2*SR*)-1-((*tert*-butoxycarbonyl)amino)-2-(tosylmethyl)cyclopropane-1-carboxylate

^{1}H NMR (500 MHz, $CDCl_3$):

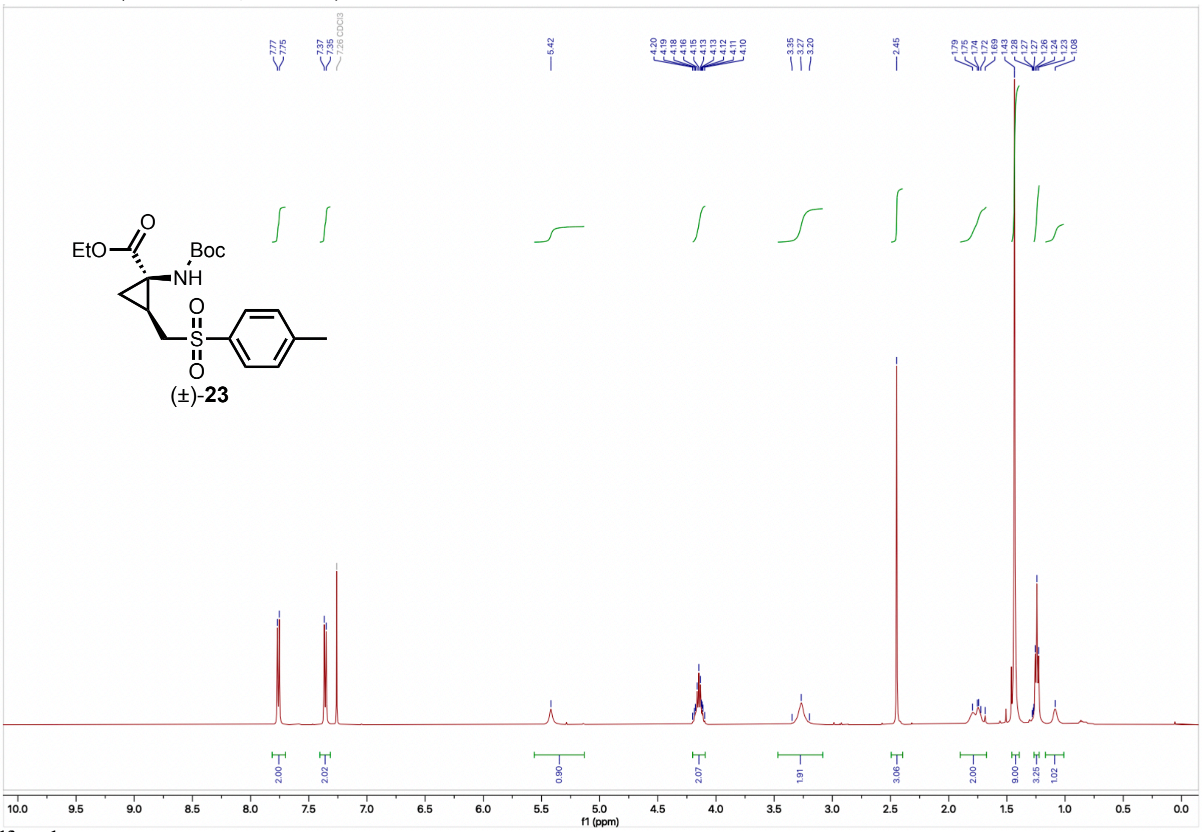

^{13}C{^{1}H} NMR (126 MHz, $CDCl_3$):

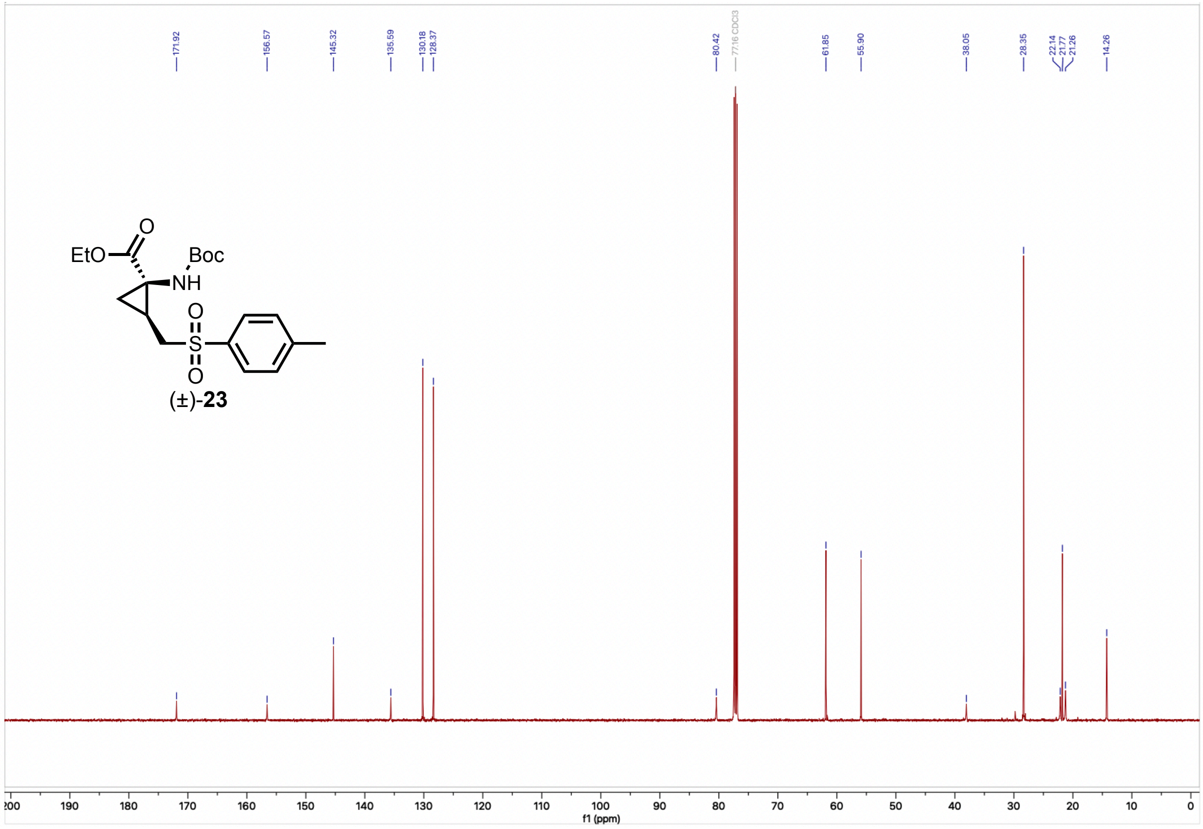

(±)-**24** - ethyl (1*RS*,2*SR*)-1-((*tert*-butoxycarbonyl)amino)-2-((tritylthio)methyl)cyclopropane-1-carboxylate

^{1}H NMR (400 MHz, CDCl$_3$, rotamers present):

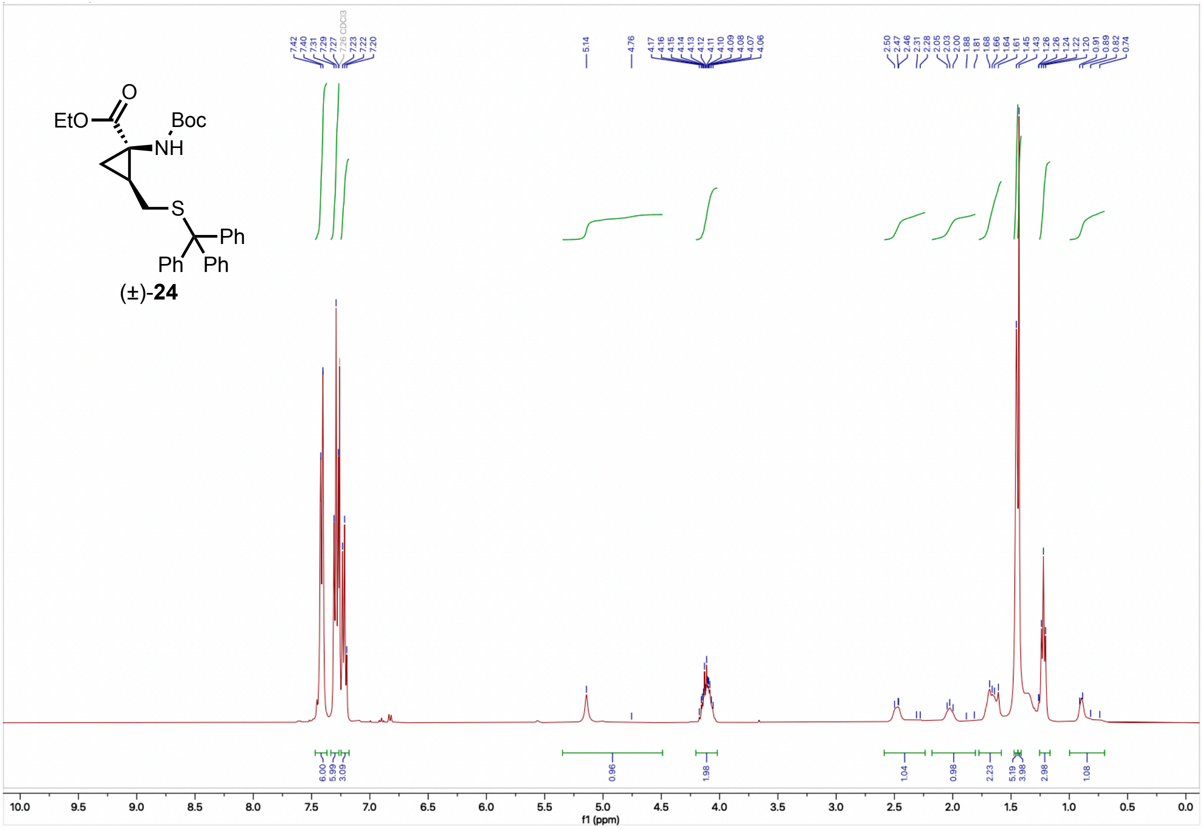

^{13}C{^{1}H} NMR (101 MHz, CDCl$_3$, rotamers present):

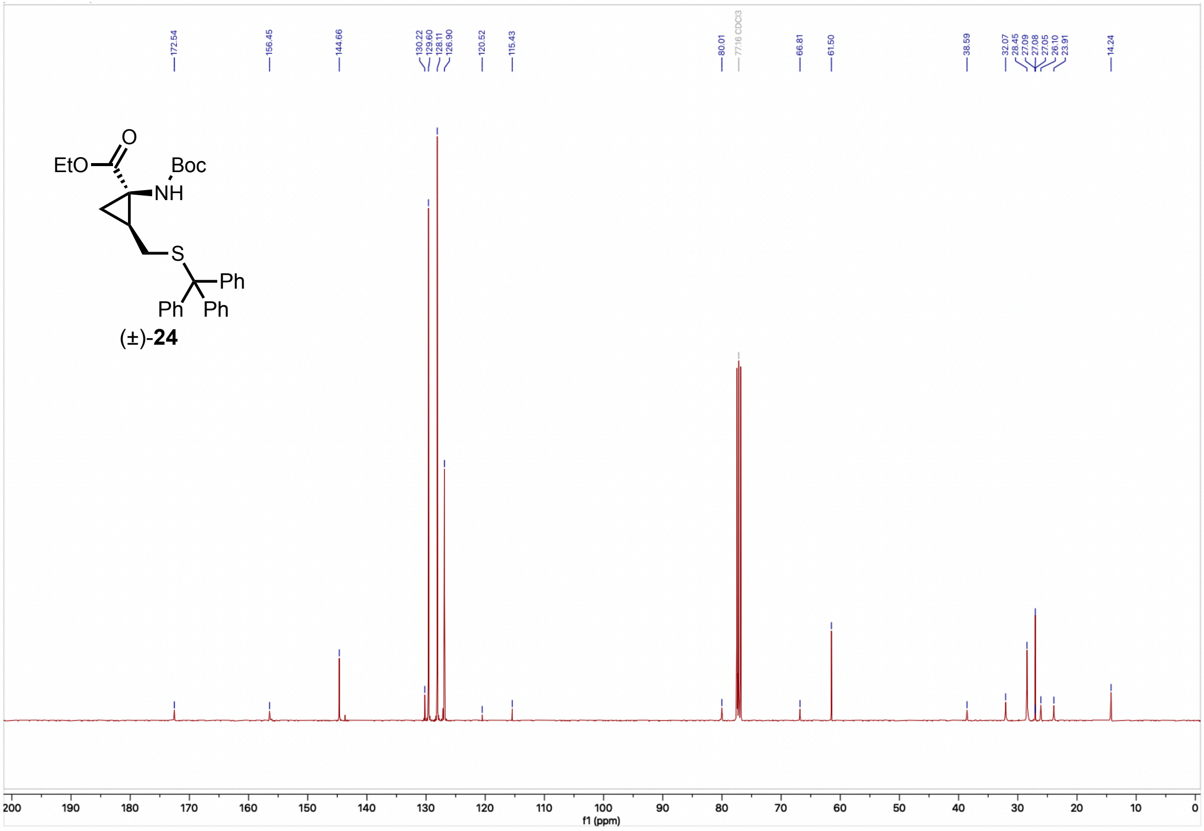

(±)-**25** - ethyl (1*RS*,2*RS*)-2-(azidomethyl)-1-((*tert*-butoxycarbonyl)amino)cyclopropane-1-carboxylate

^{1}H NMR (400 MHz, CDCl$_3$):

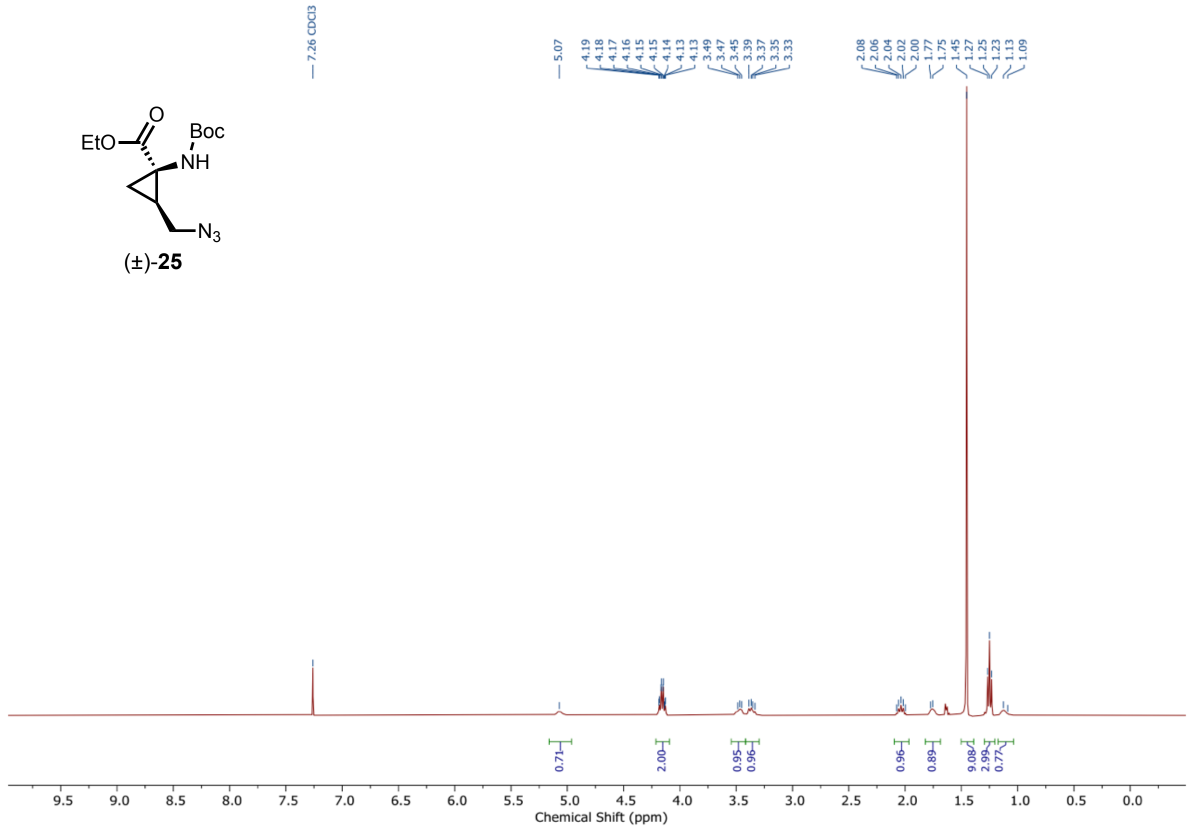

^{13}C NMR (101 MHz, CDCl$_3$):

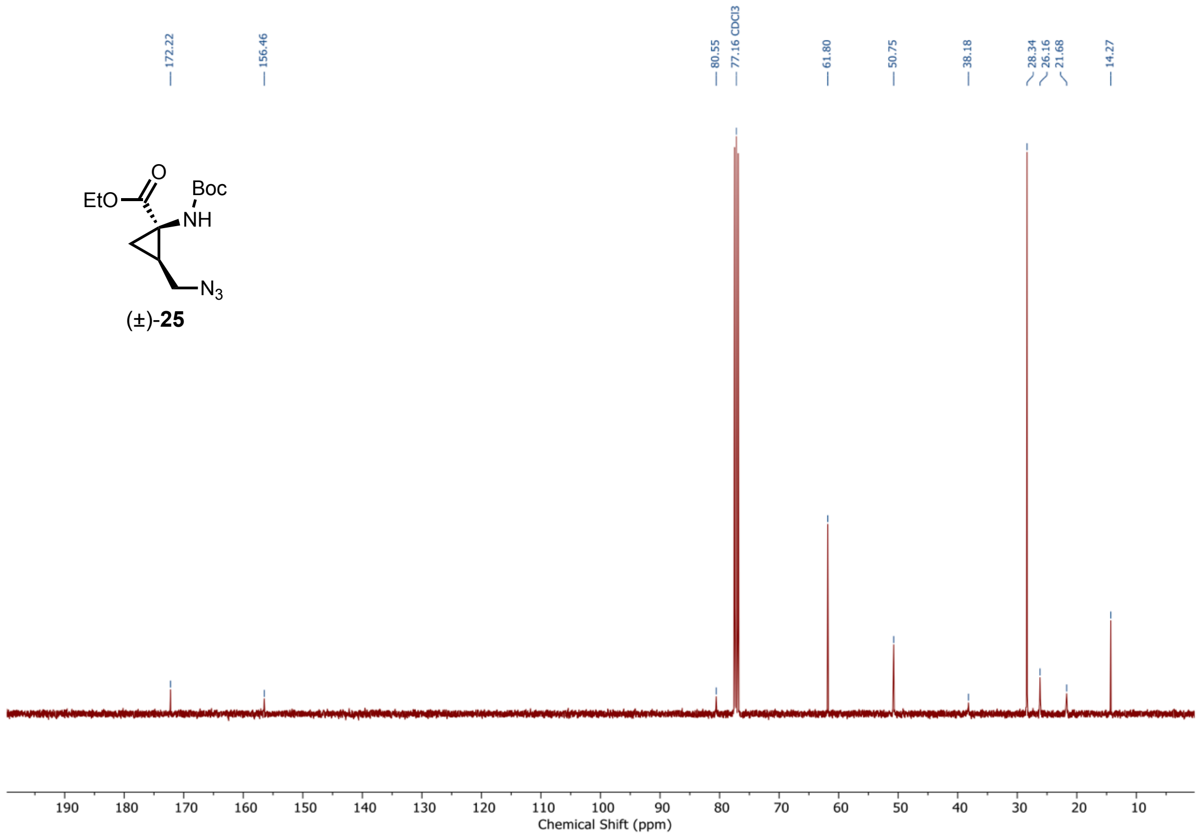

26 - ethyl (*Z*)-2-((*tert*-butoxycarbonyl)amino)penta-2,4-dienoate

^{1}H NMR (500 MHz, CDCl$_3$):

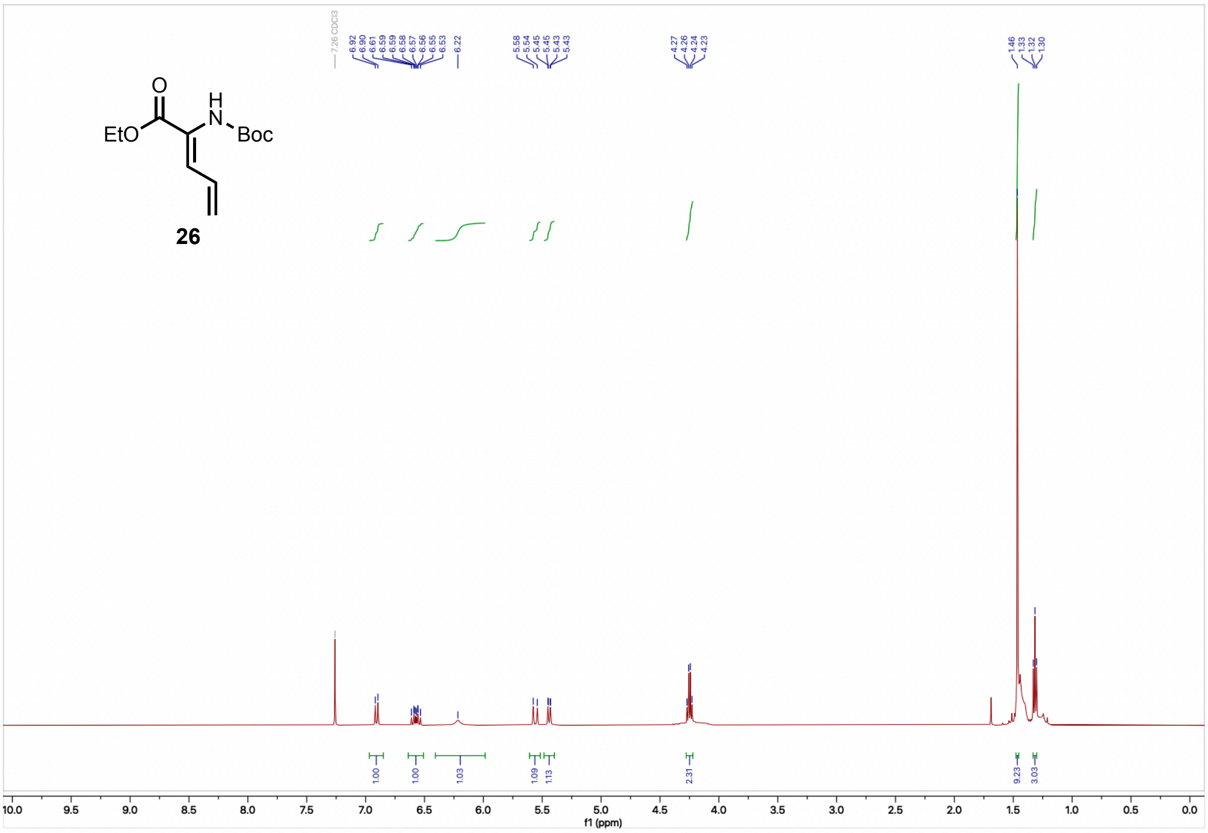

^{13}C{^{1}H} NMR (126 MHz, CDCl$_3$):

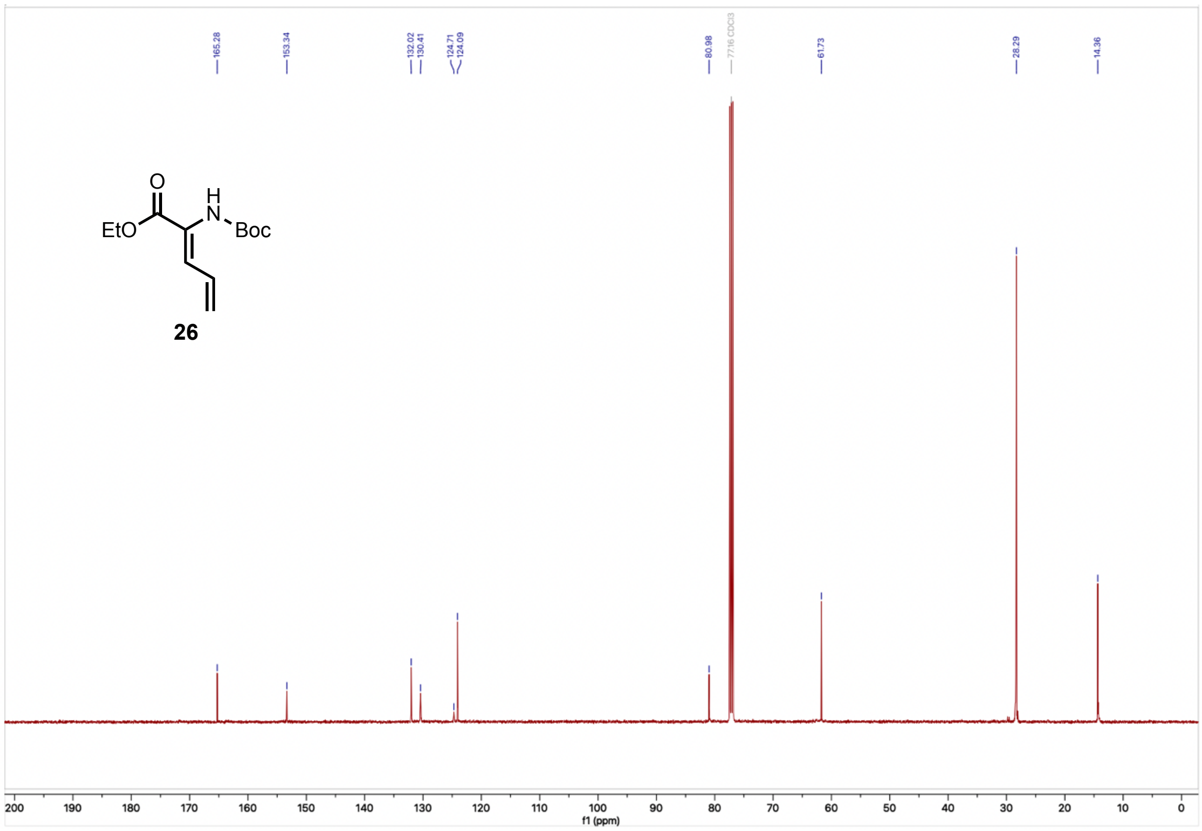

(±)-**27** - 2-(2-chloroethyl)oxirane

^{1}H NMR (400 MHz, $CDCl_3$):

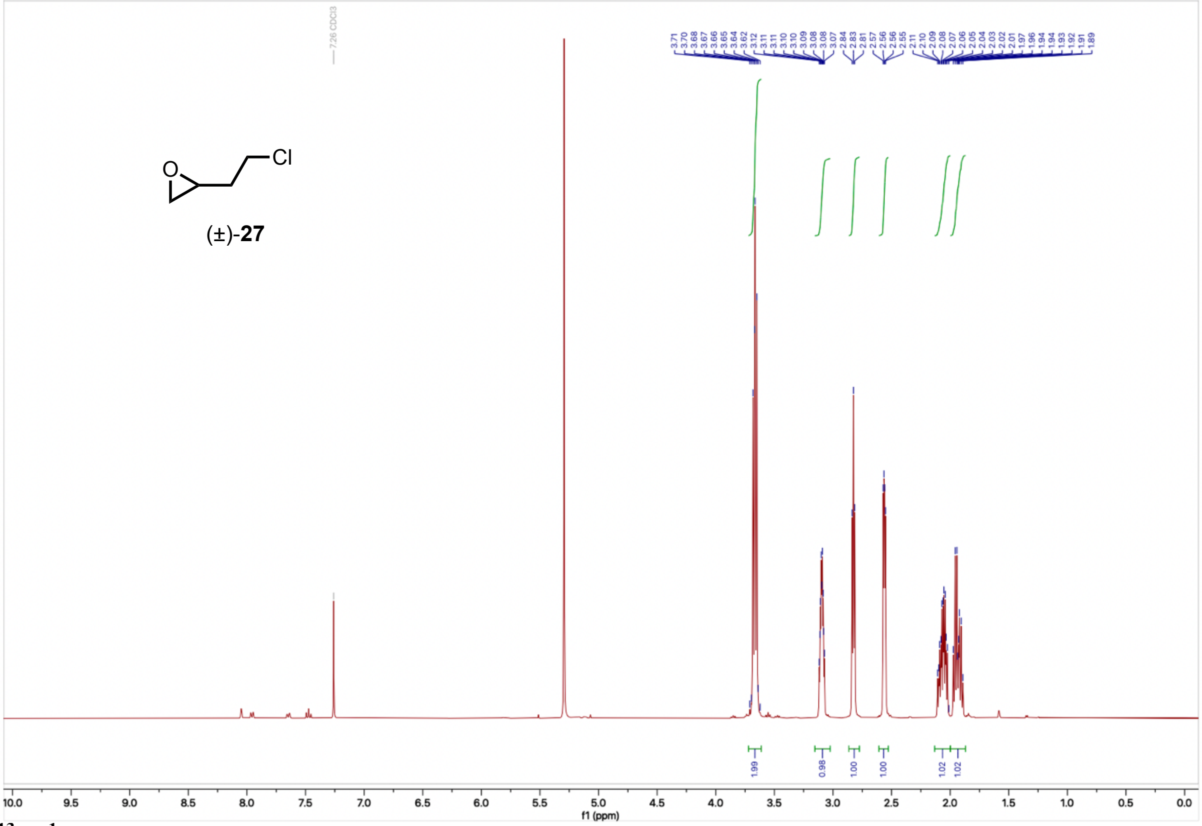

^{13}C{^{1}H} NMR (101 MHz, $CDCl_3$):

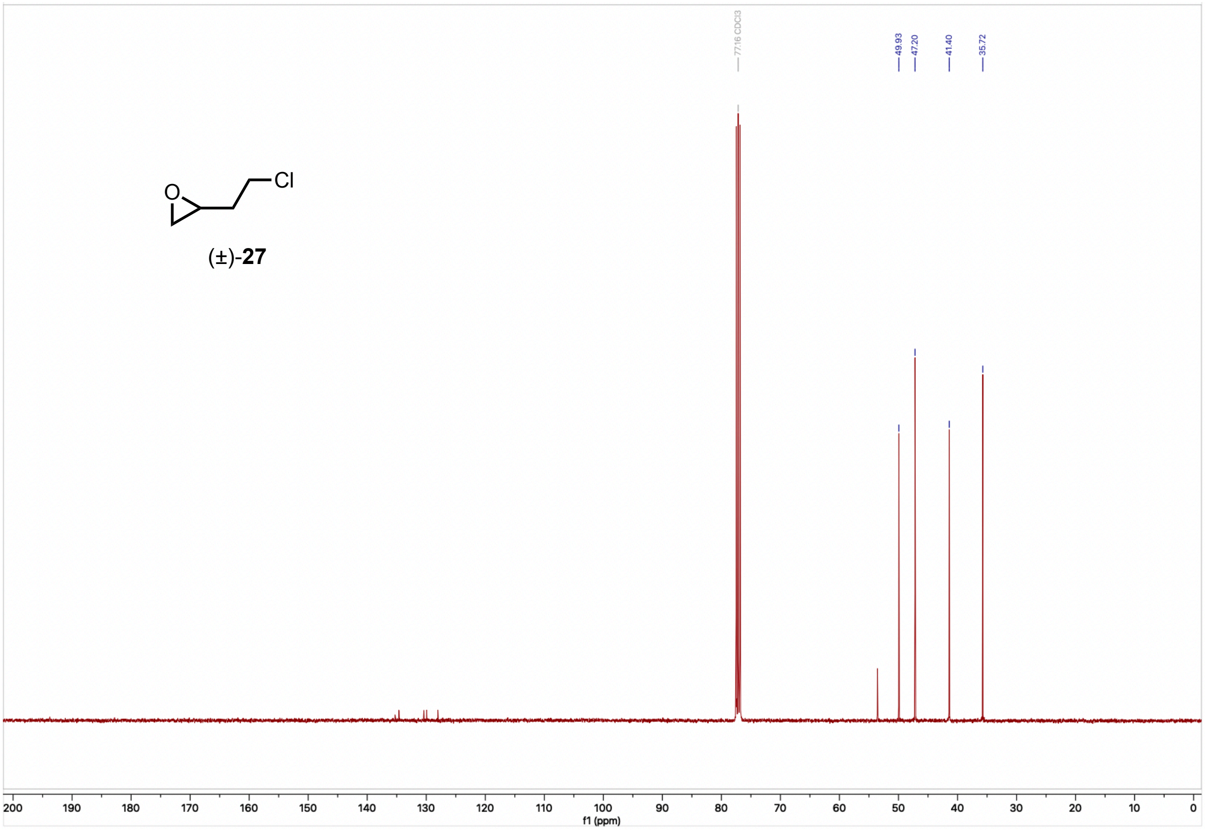

(±)-**29** - diethyl 3-hydroxycyclopentane-1,1-dicarboxylate

^{1}H NMR (400 MHz, CDCl$_3$):

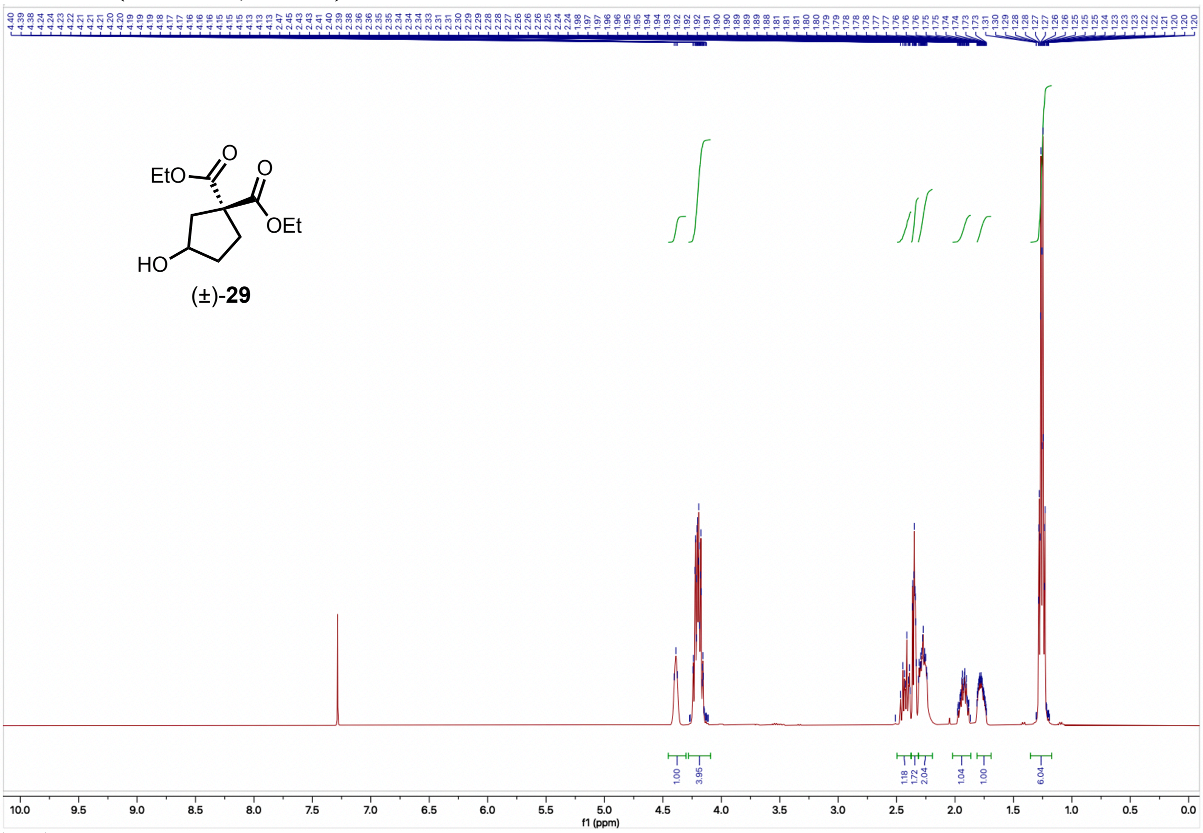

^{1}H-^{1}H COSY:

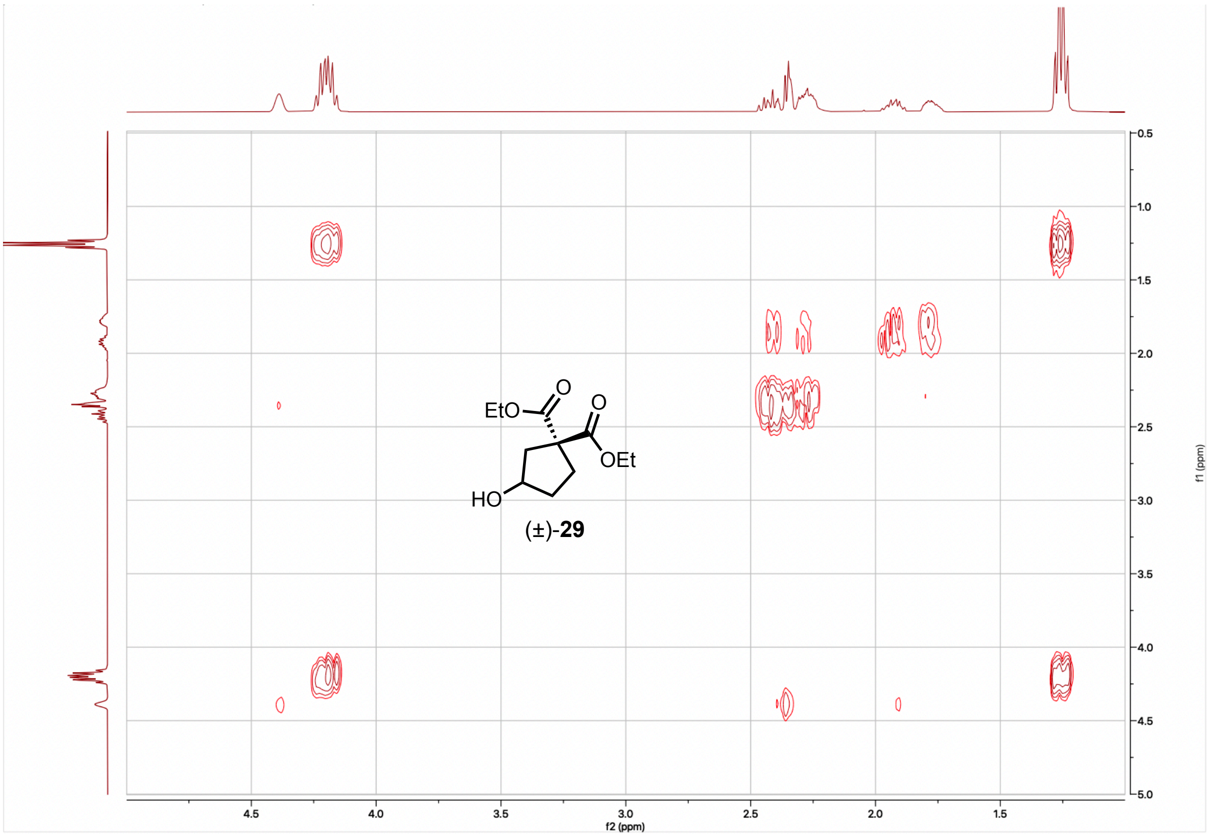

$^{13}C\{^{1}H\}$ NMR (101 MHz, $CDCl_3$):

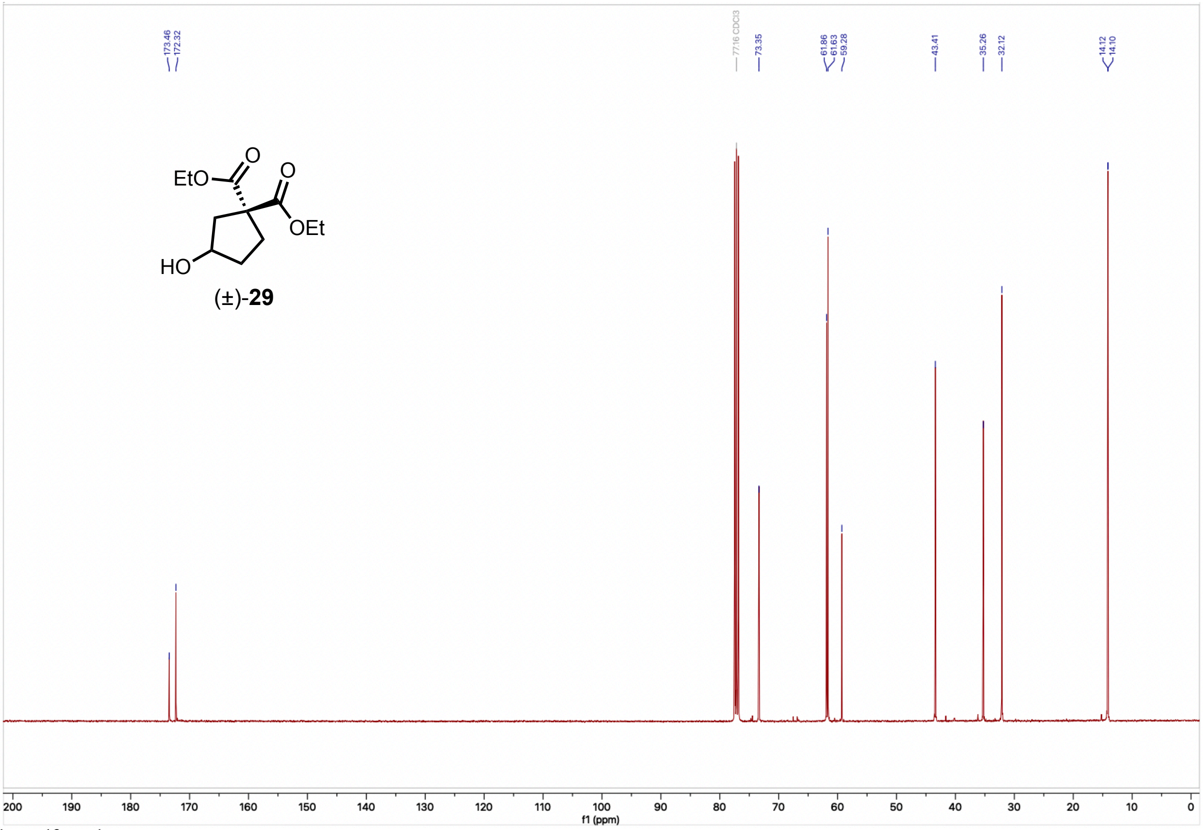

^{1}H-$^{13}C\{^{1}H\}$ HSQC:

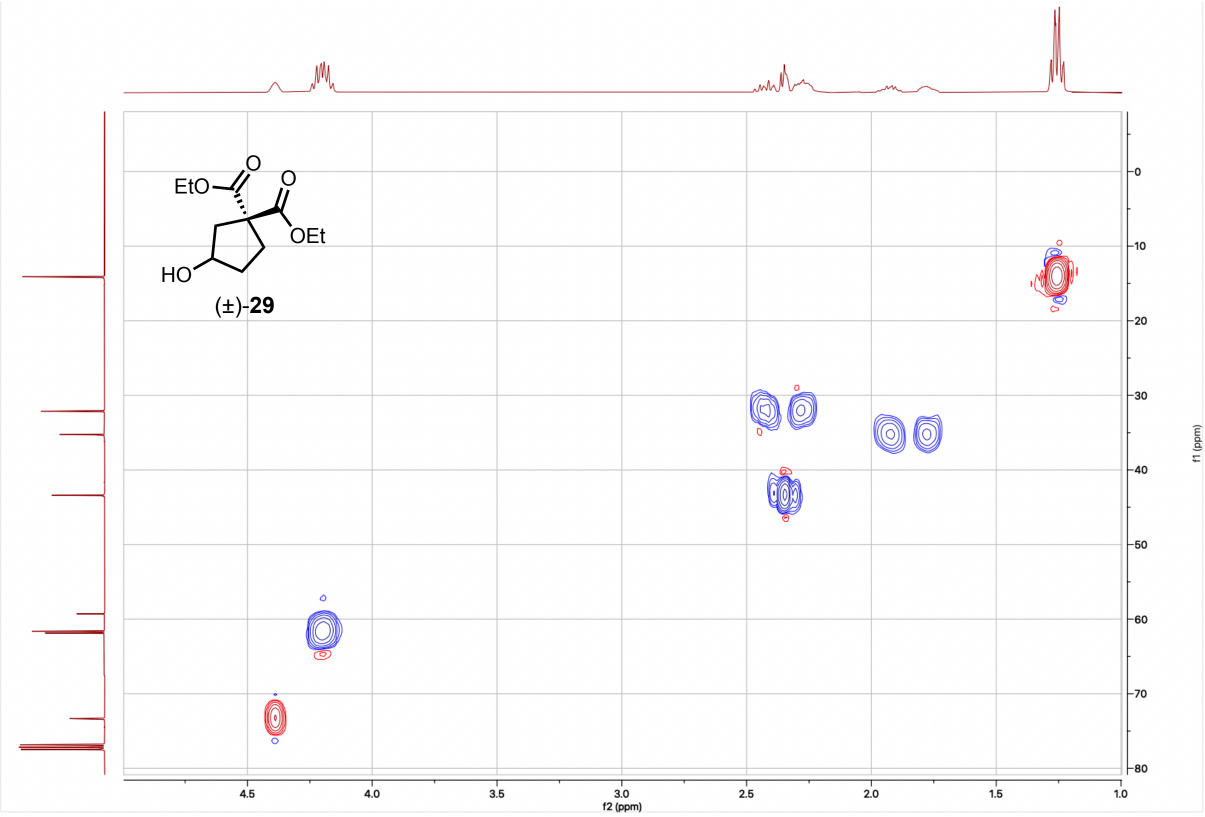

^{1}H-$^{13}C\{^{1}H\}$ HMBC:

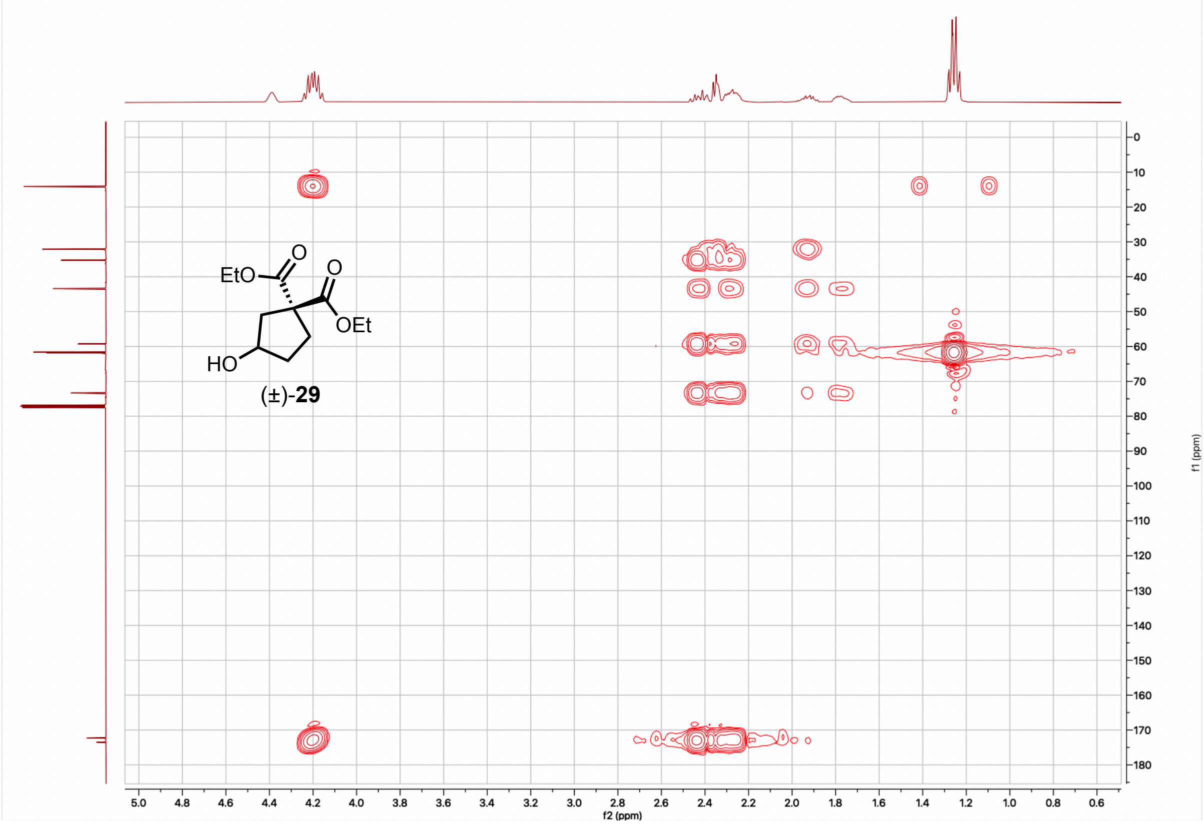

(±)-**30** - ethyl (1*RS*,2*SR*)-1-amino-2-(chloromethyl)cyclopropane-1-carboxylate hydrochloride

^{1}H NMR (400 MHz, $CDCl_3$):

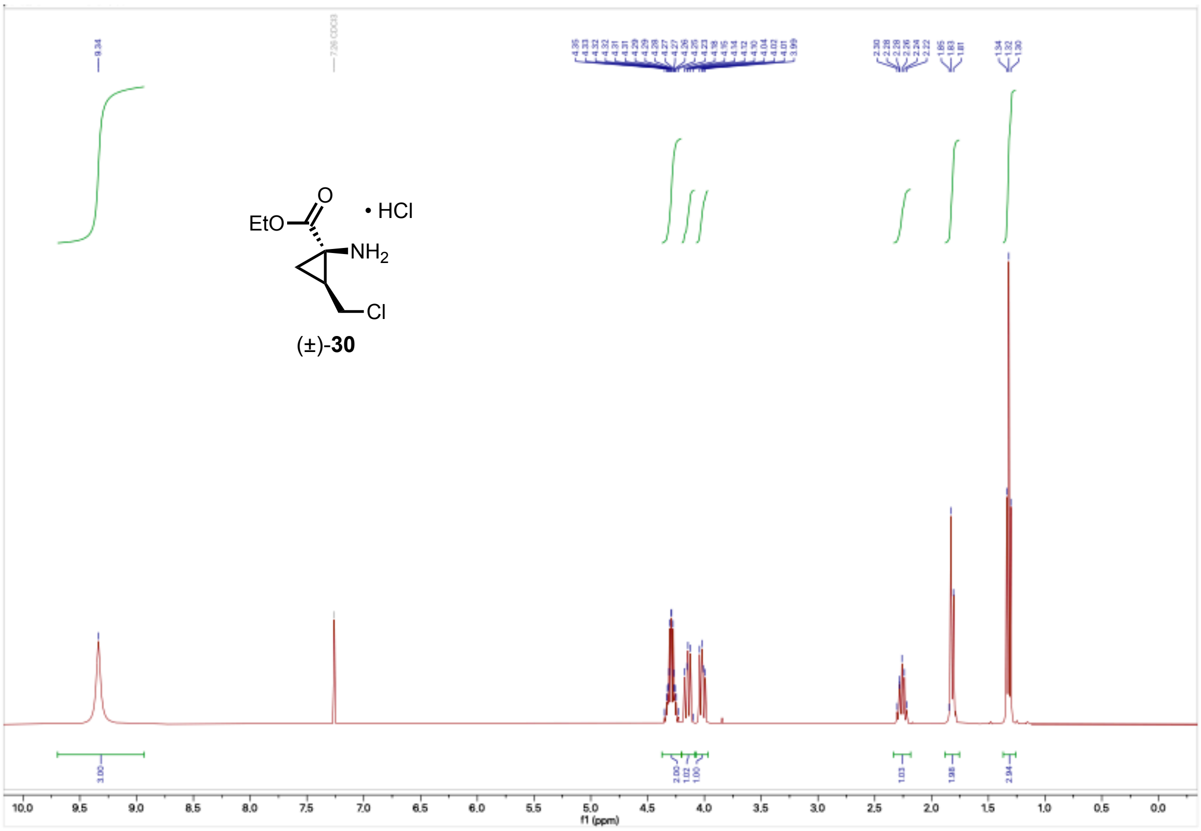

$^{13}C\{^{1}H\}$ NMR (101 MHz, $CDCl_3$):

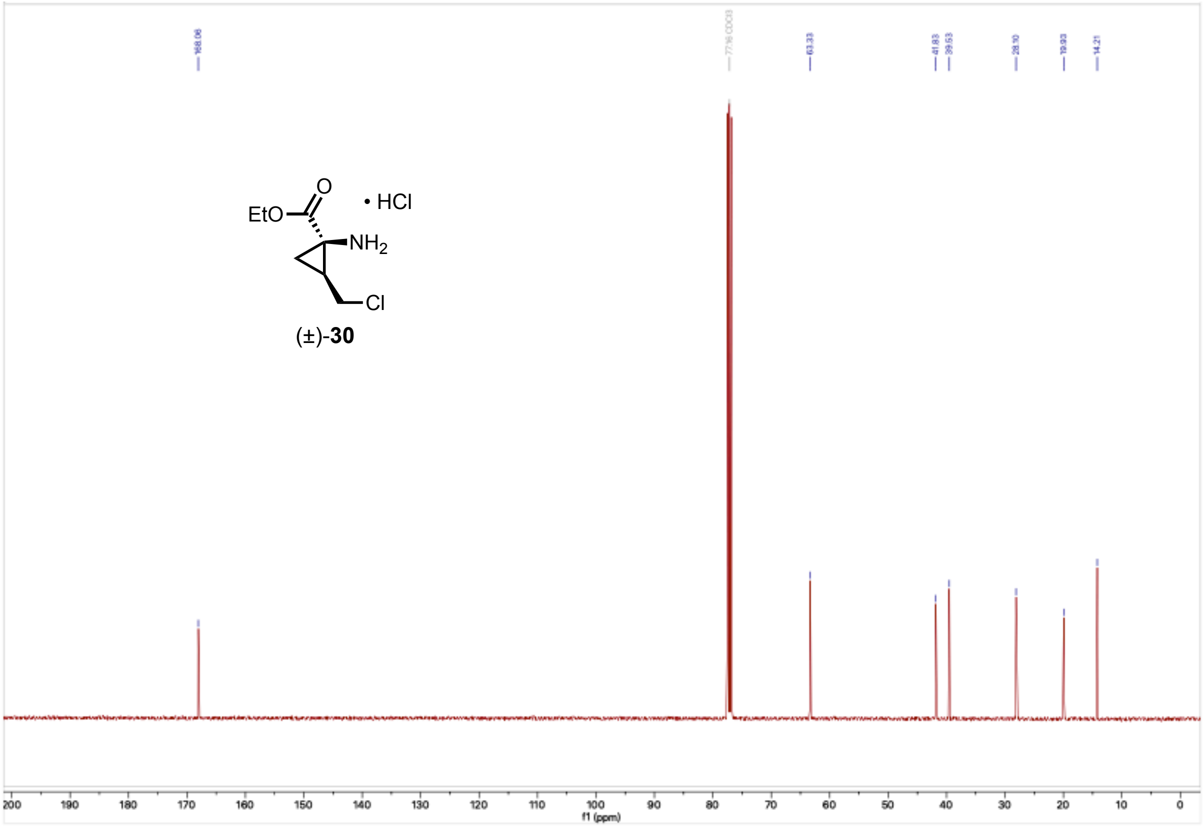

(±)-**31** - (1*RS*,5*SR*)-1-phenyl-3-oxabicyclo[3.1.0]hexan-2-one

^{1}H NMR (400 MHz, $CDCl_3$):

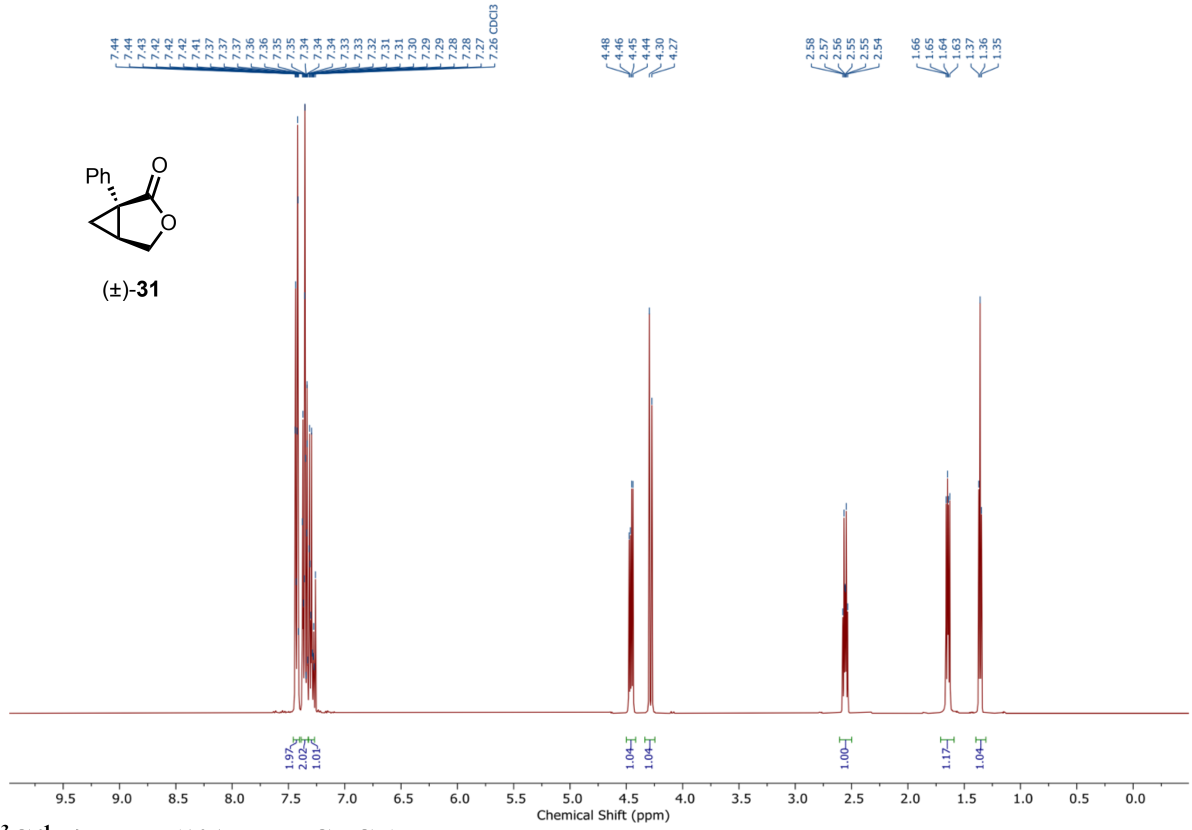

^{13}C{^{1}H} NMR (101 MHz, $CDCl_3$):

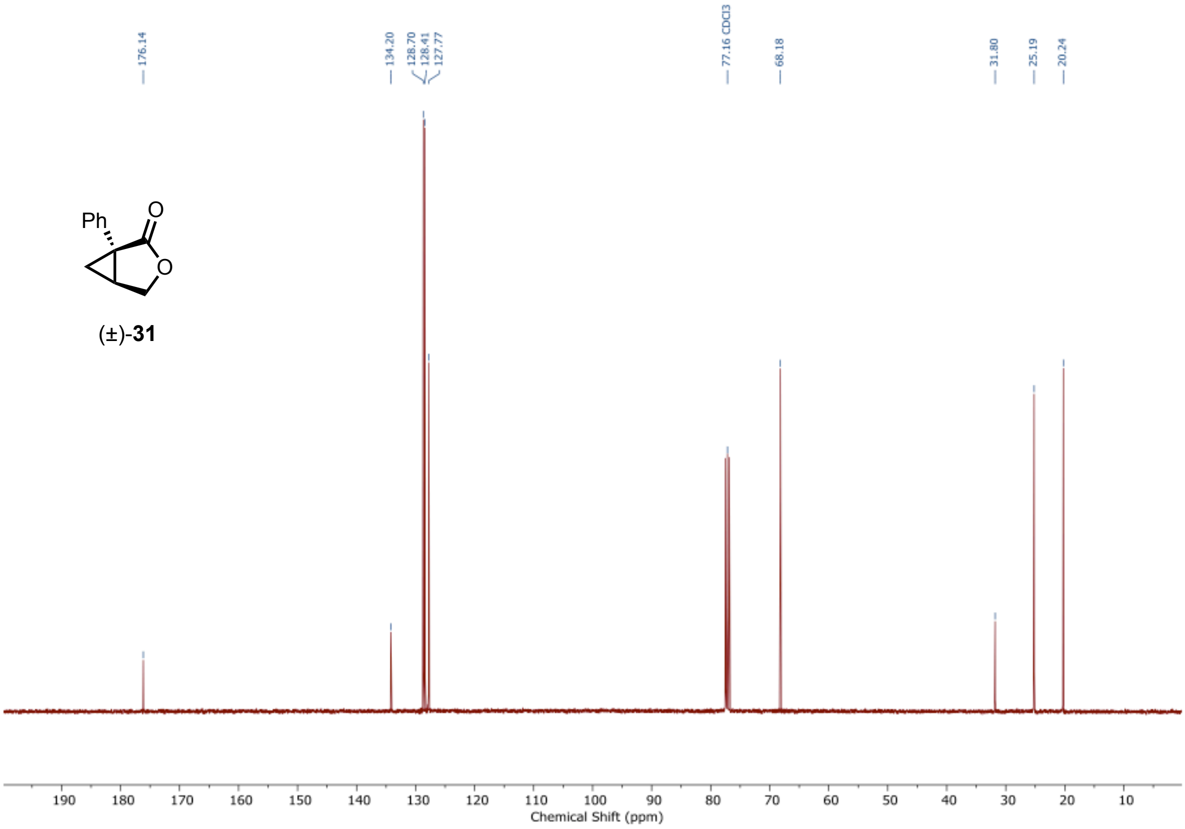

(±)-**32** - (1*RS*,2*SR*)-2-(hydroxymethyl)-1-phenylcyclopropane-1-carboxamide

^{1}H NMR (400 MHz, CDCl$_3$):

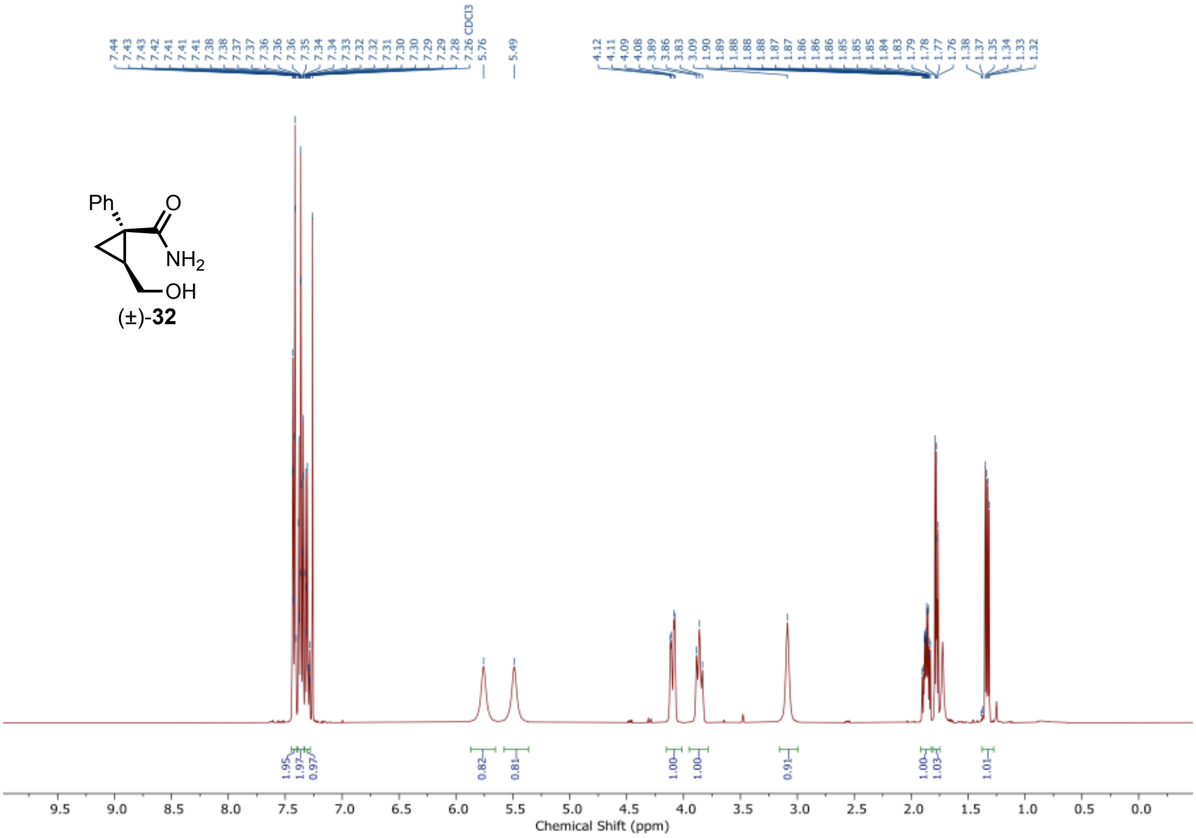

^{13}C{^{1}H} NMR (101 MHz, CDCl$_3$):

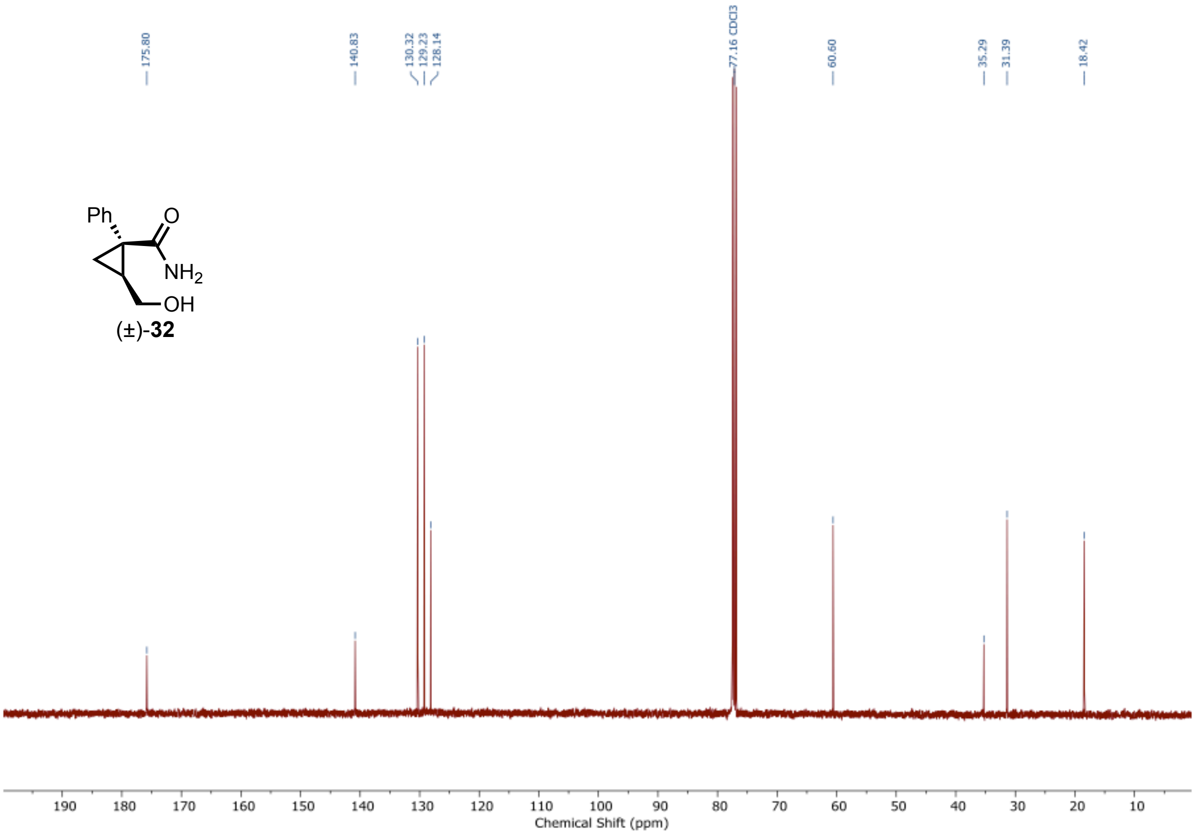

(±)-**33** - (1*RS*,6*SR*)-1-phenyl-4-oxa-2-azabicyclo[4.1.0]heptan-3-one

^{1}H NMR (400 MHz, CDCl$_3$):

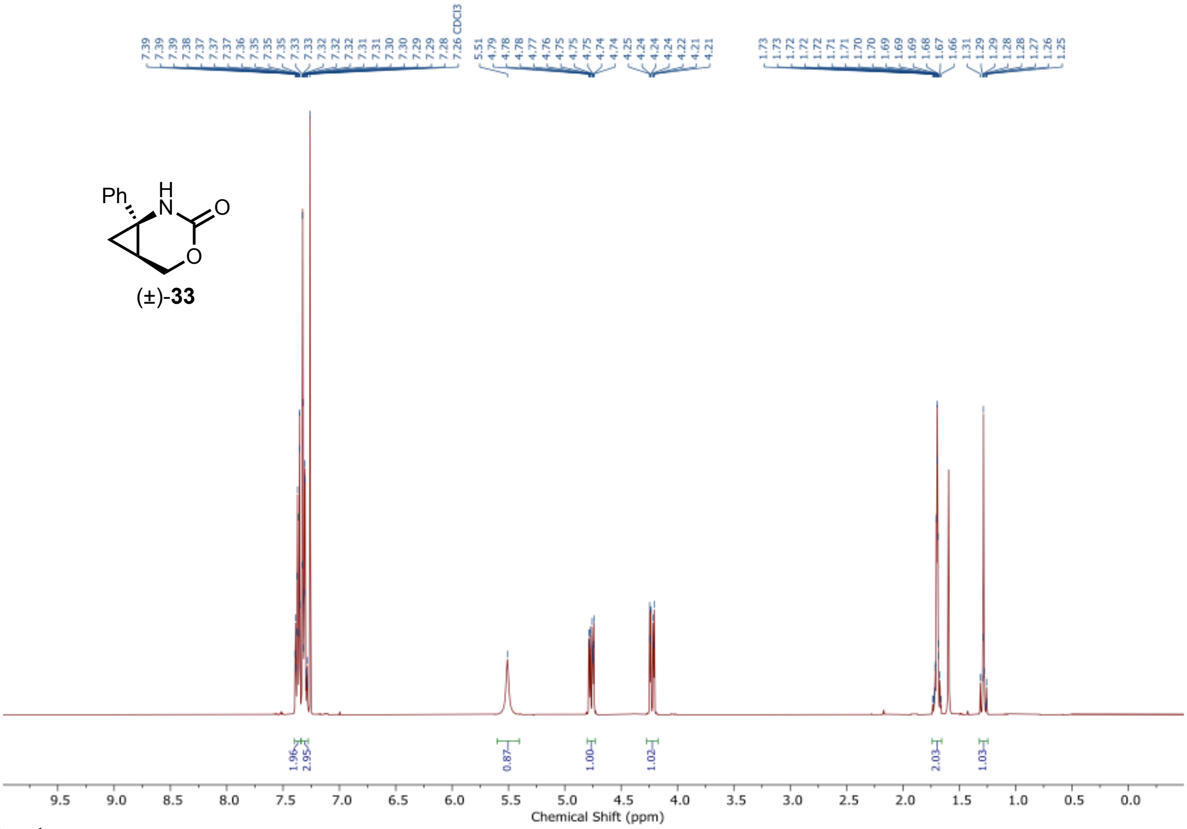

^{13}C{^{1}H} NMR (101 MHz, CDCl$_3$):

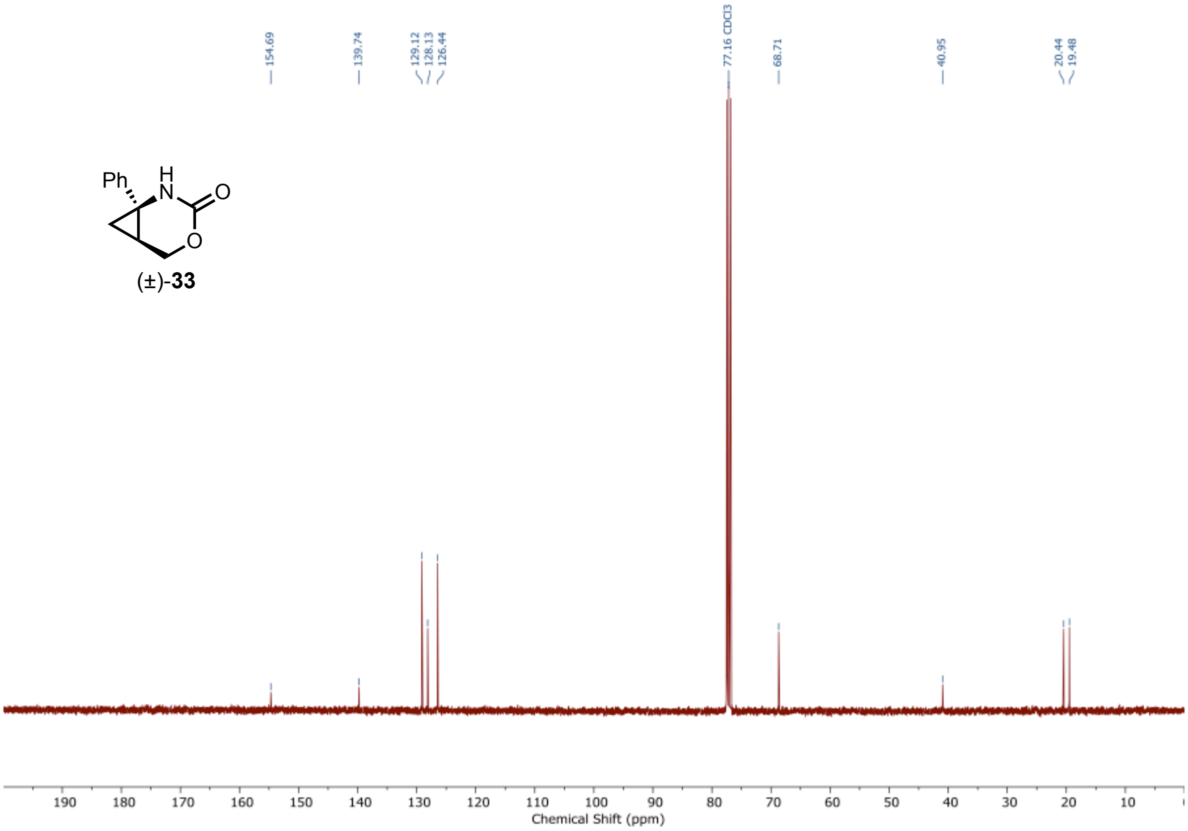

(±)-**34** - tert-butyl (1*RS*,6*SR*)-3-oxo-1-phenyl-4-oxa-2-azabicyclo[4.1.0]heptane-2-carboxylate

^{1}H NMR (400 MHz, CDCl$_3$):

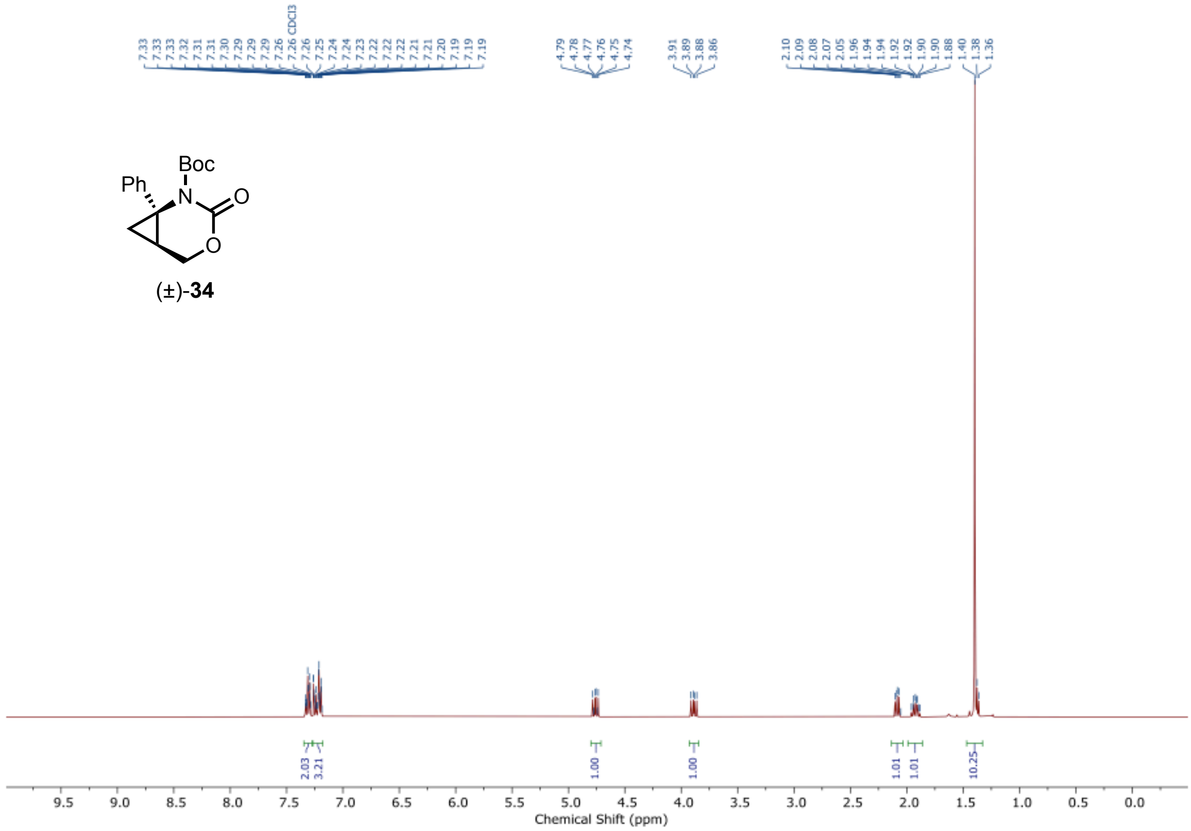

^{13}C{^{1}H} NMR (101 MHz, CDCl$_3$):

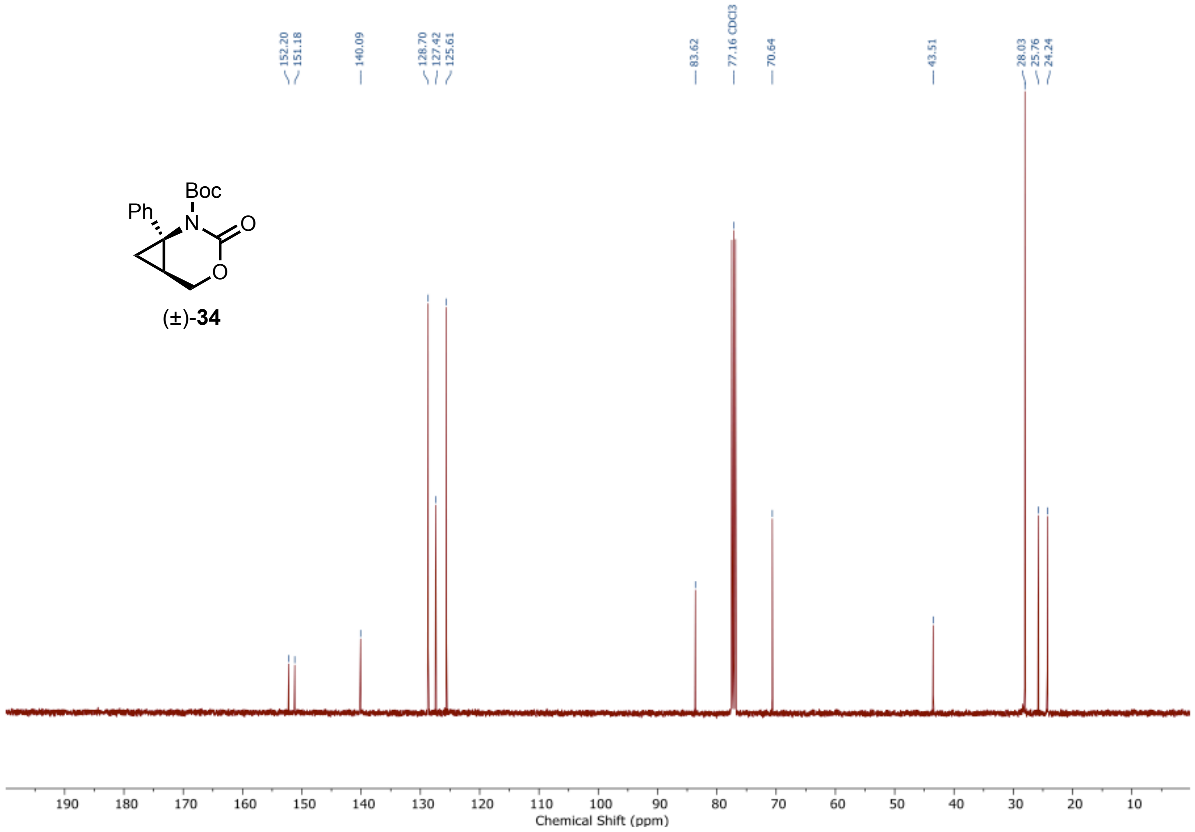

(±)-**35** - *tert*-butyl ((1*RS*,2*SR*)-2-(hydroxymethyl)-1-phenylcyclopropyl)carbamate

^{1}H NMR (400 MHz, $CDCl_3$):

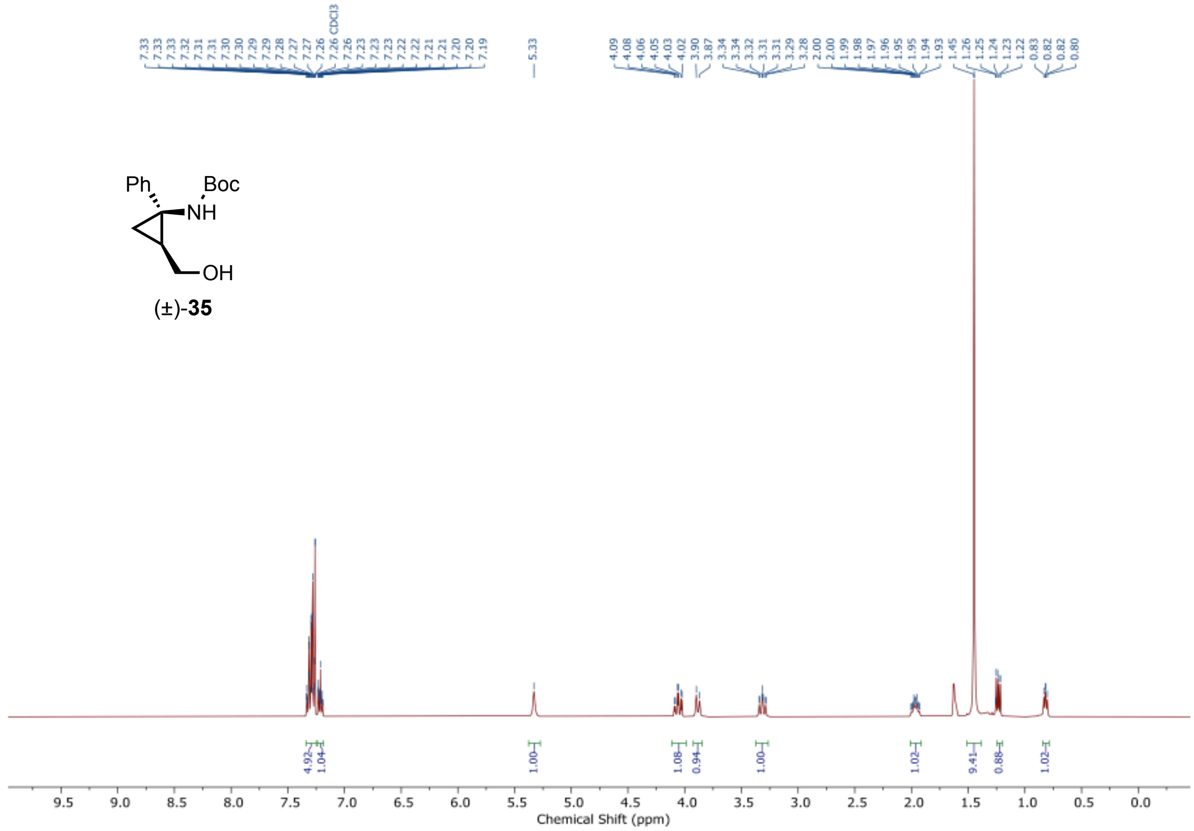

^{13}C{^{1}H} NMR (101 MHz, $CDCl_3$):

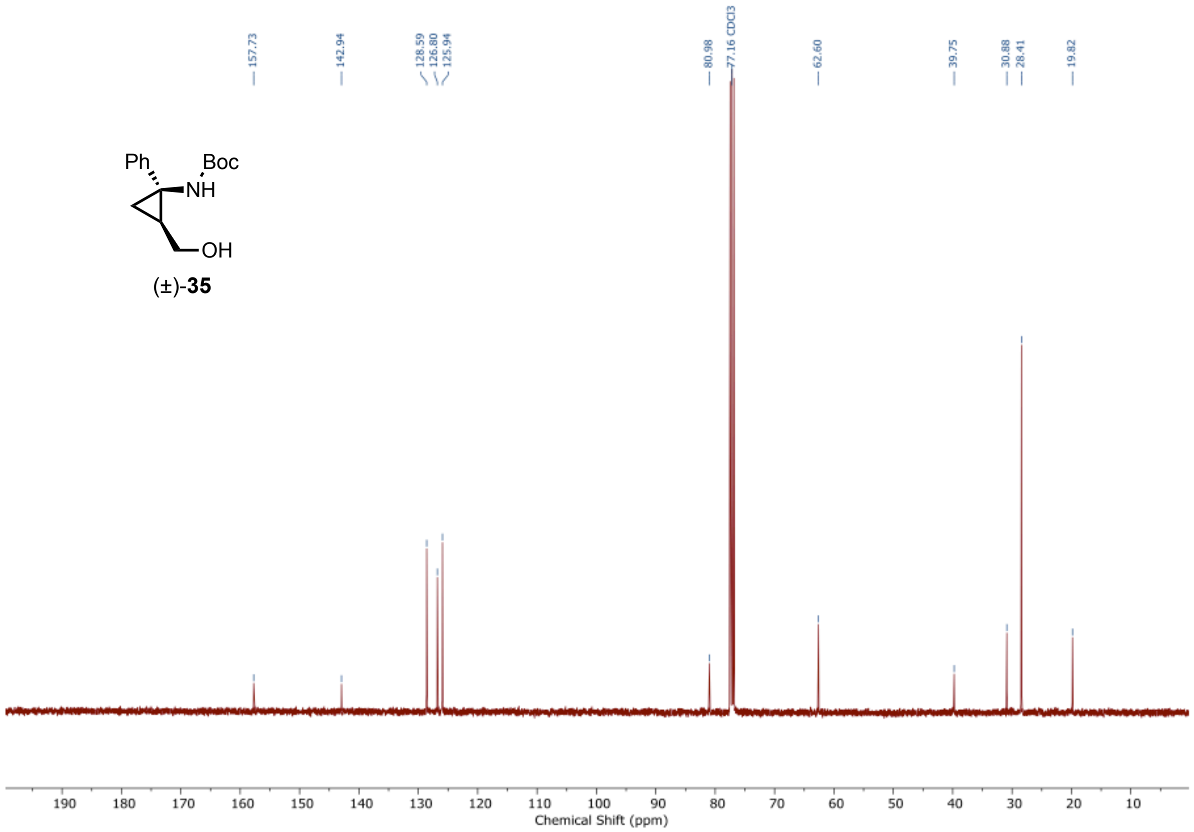

(1*S*,2*R*)-**36** - (1*S*,2*R*)-1-((((9*H*-fluoren-9-yl)methoxy)carbonyl)amino)-2-(hydroxymethyl)cyclopropane-1-carboxylic acid

^{1}H NMR (400 MHz, DMSO-*d*6, rotamers present):

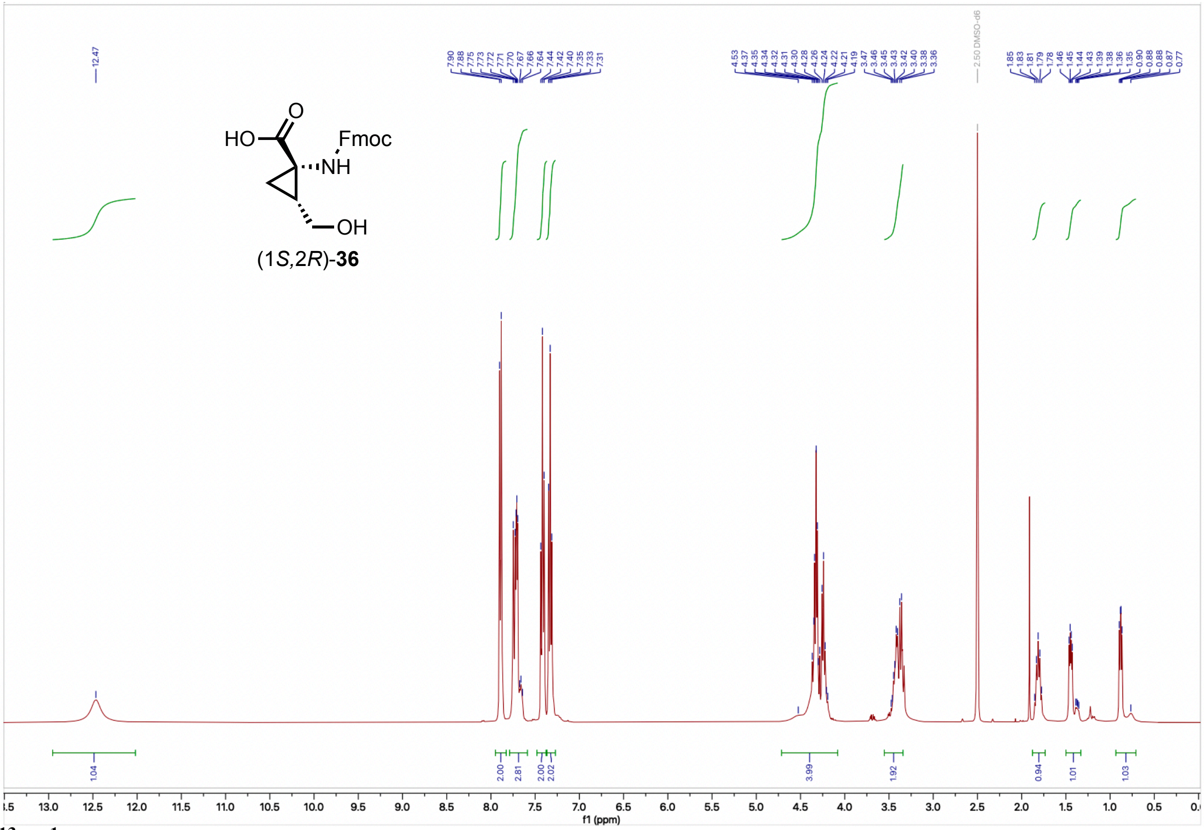

^{13}C{^{1}H} NMR (101 MHz, DMSO-*d*6, rotamers present):

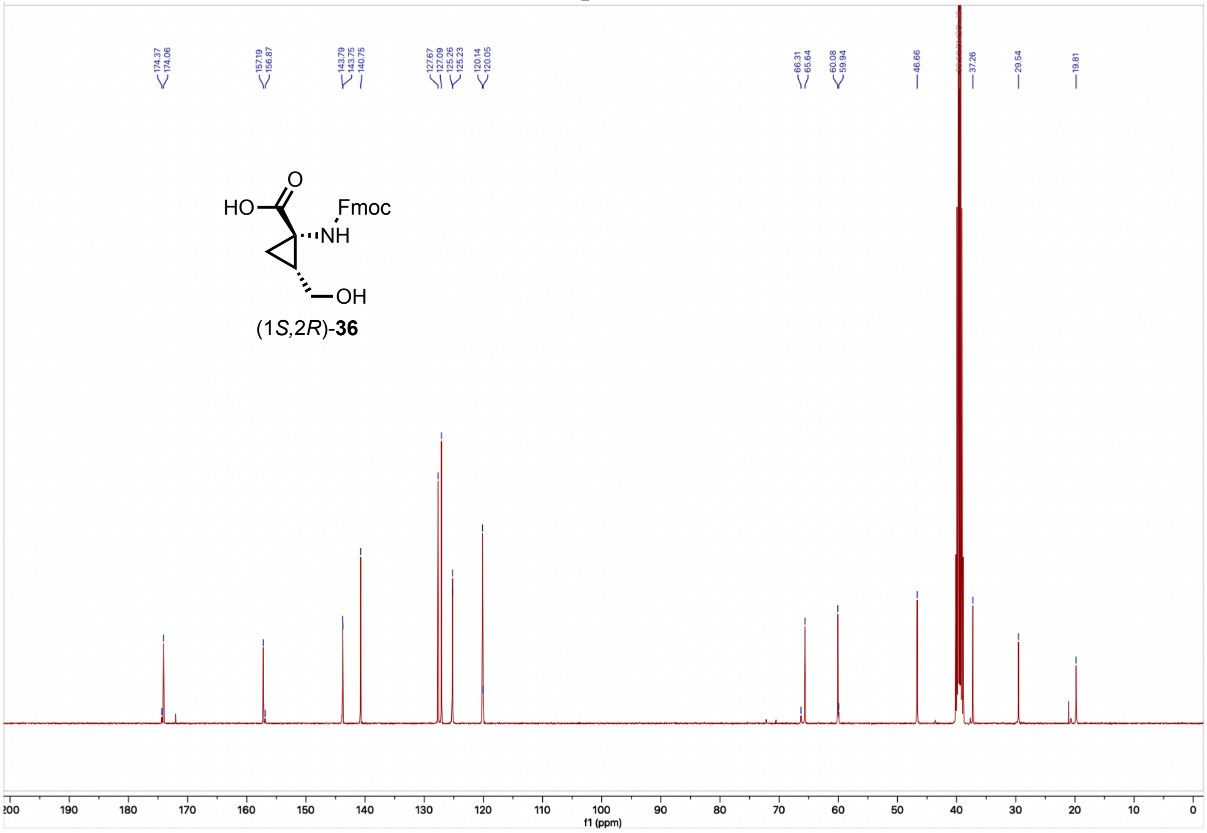

(±)-**37** - Osteostatin analogue (H-TRXAW-OH)

^{1}H NMR (400 MHz, DMSO-*d6*):

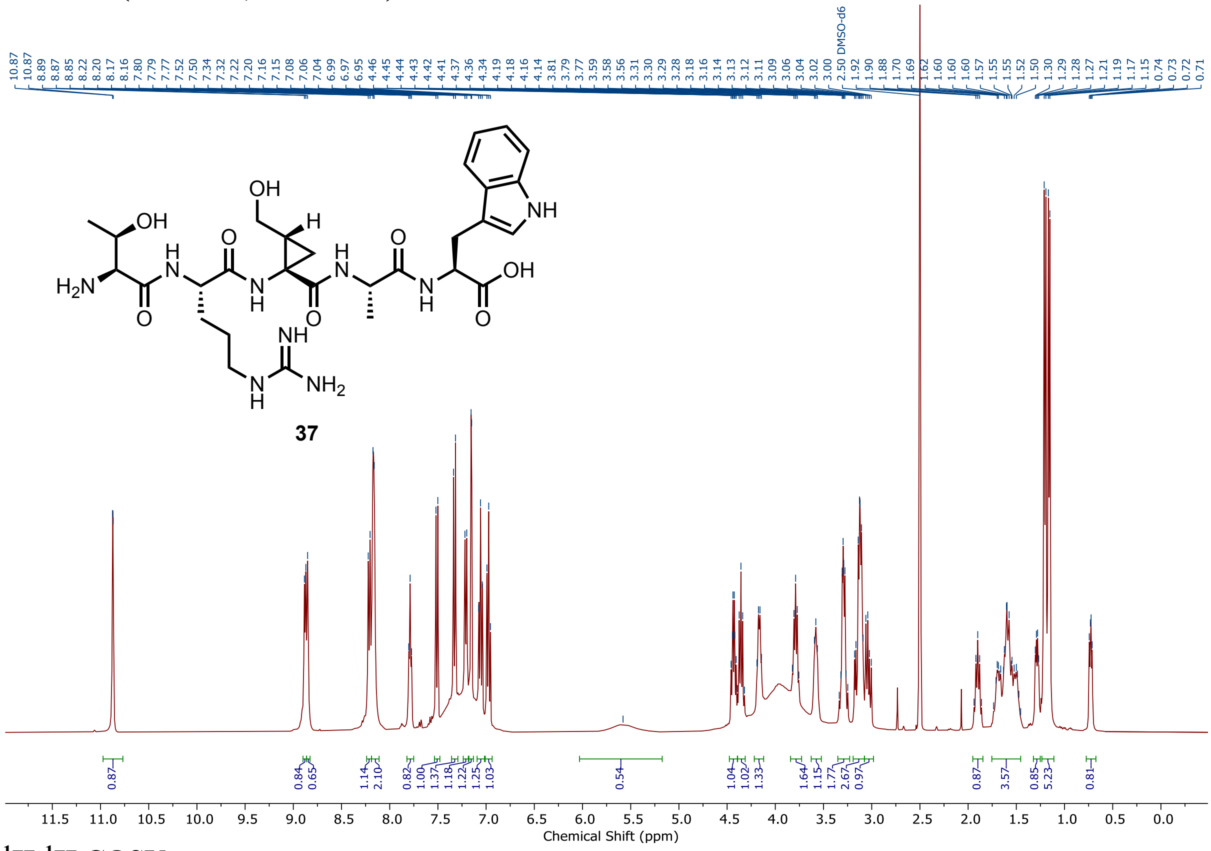

^{1}H-^{1}H COSY:

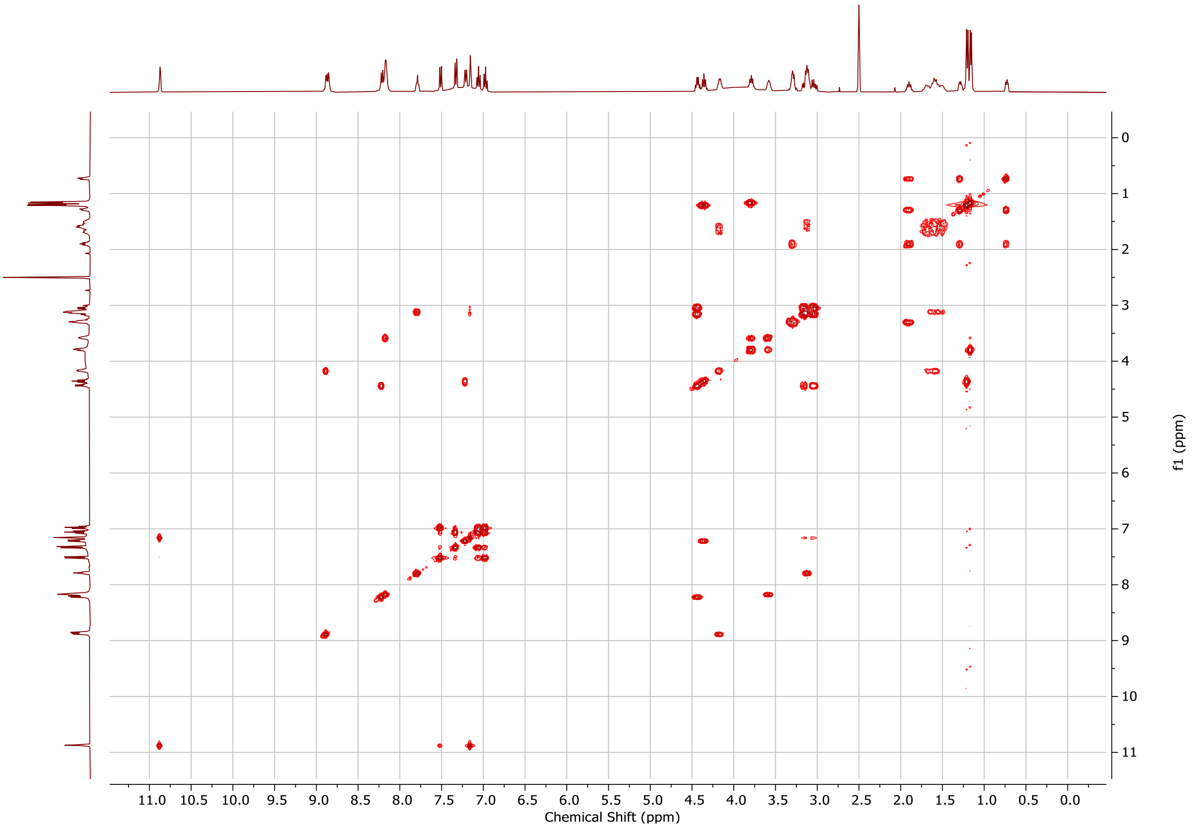

$^{13}C\{^{1}H\}$ NMR (101 MHz, DMSO-*d6*):

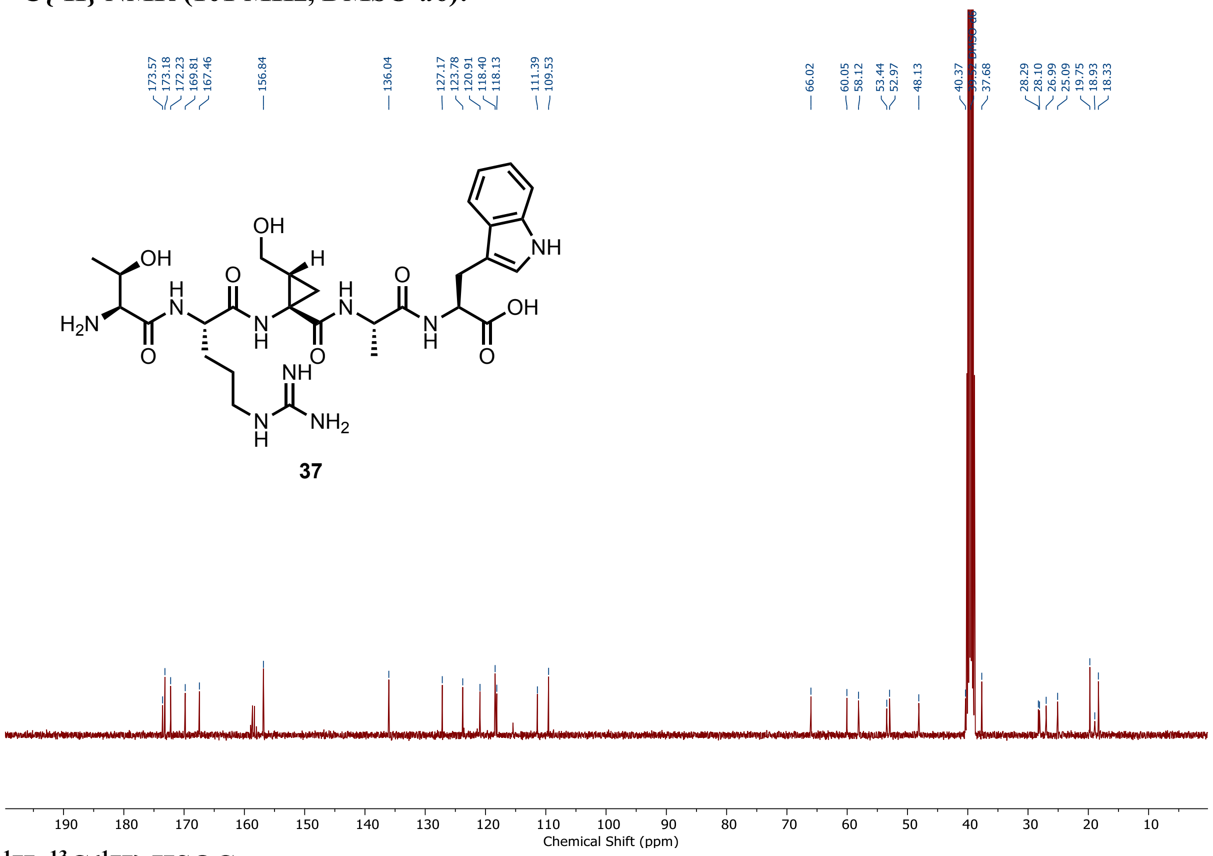

^{1}H-$^{13}C\{^{1}H\}$ HSQC:

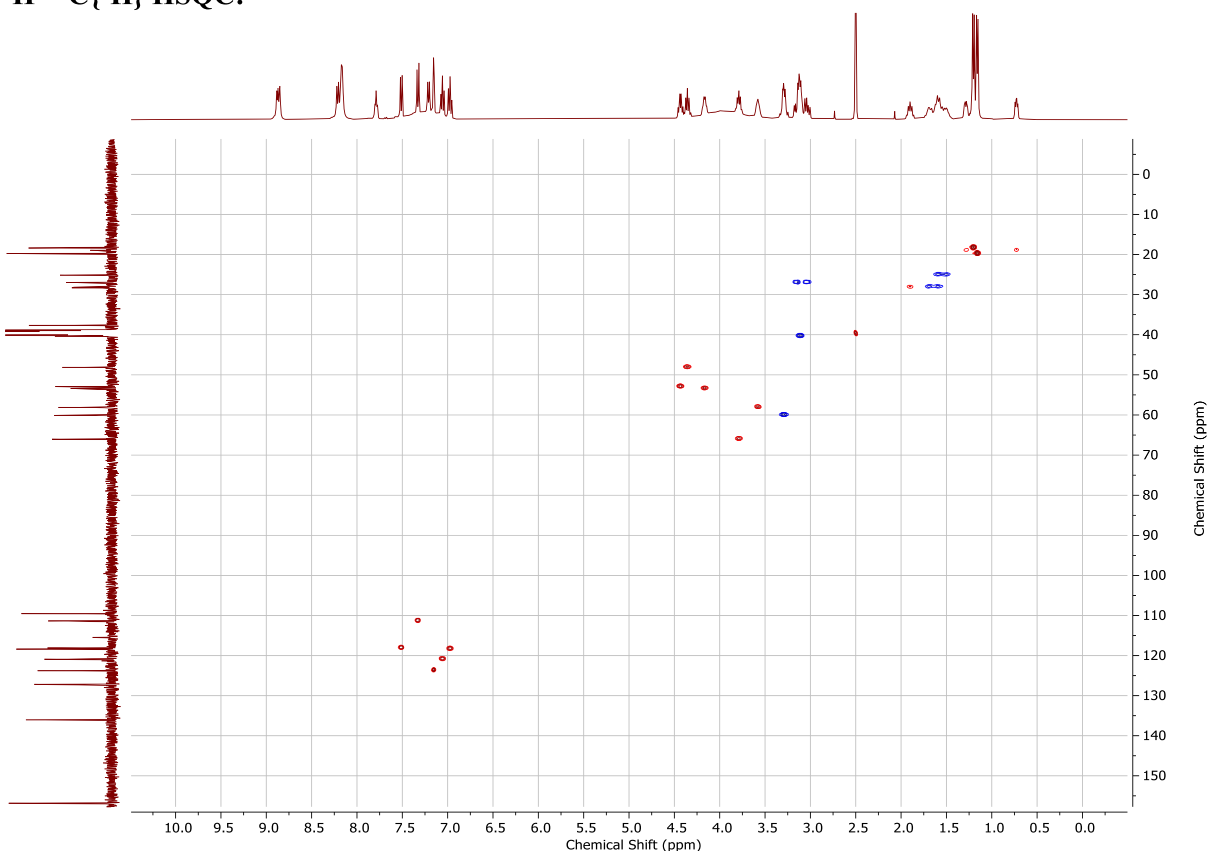

(±)-**SI-1** - ethyl (1*RS*,2*RS*)-2-(((*tert*-butyldimethylsilyl)oxy)methyl)-1-carbamoylcyclopropane-1-carboxylate

^{1}H NMR (400 MHz, CDCl$_3$):

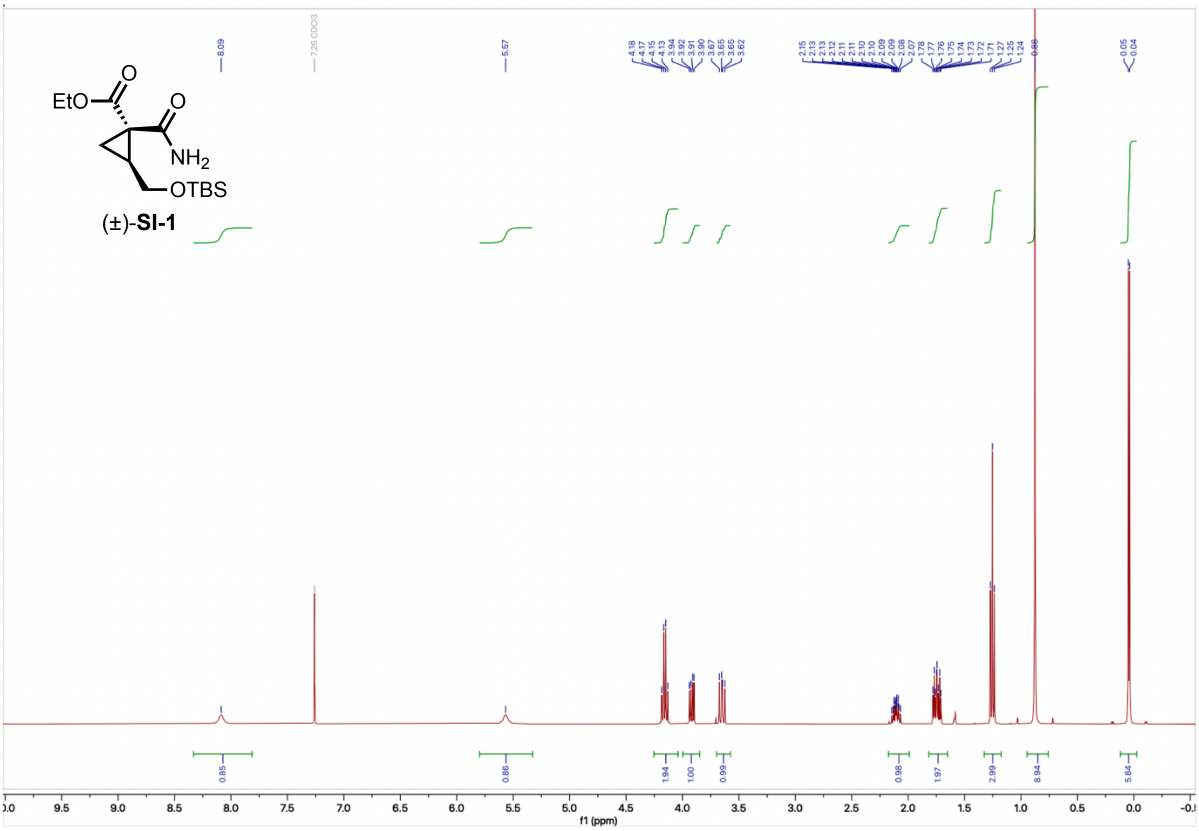

^{13}C{^{1}H} NMR (101 MHz, CDCl$_3$):

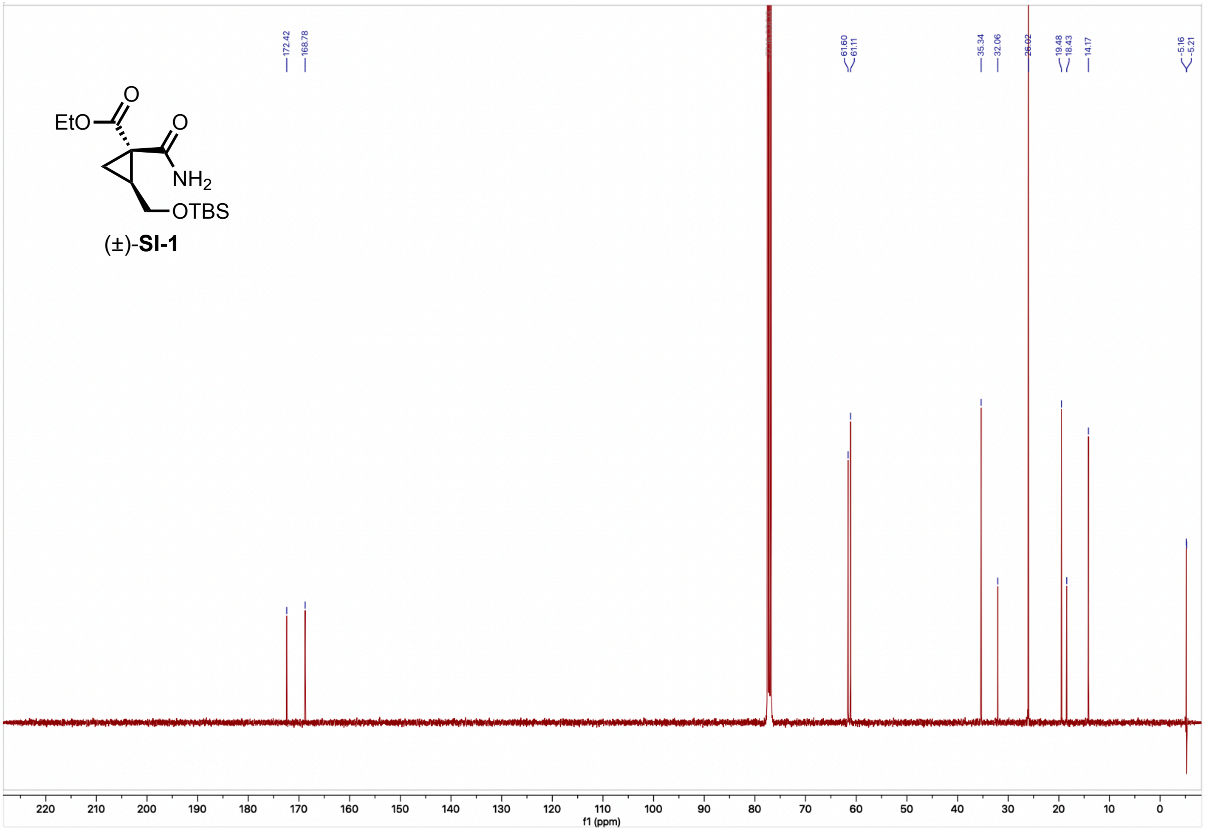

(±)-**SI-2** - ethyl (1*SR*,2*RS*)-1-((*tert*-butoxycarbonyl)amino)-2-(((*tert*-butyldimethylsilyl)oxy)methyl)cyclopropane-1-carboxylate

^{1}H NMR (400 MHz, CDCl$_3$, rotamers present):

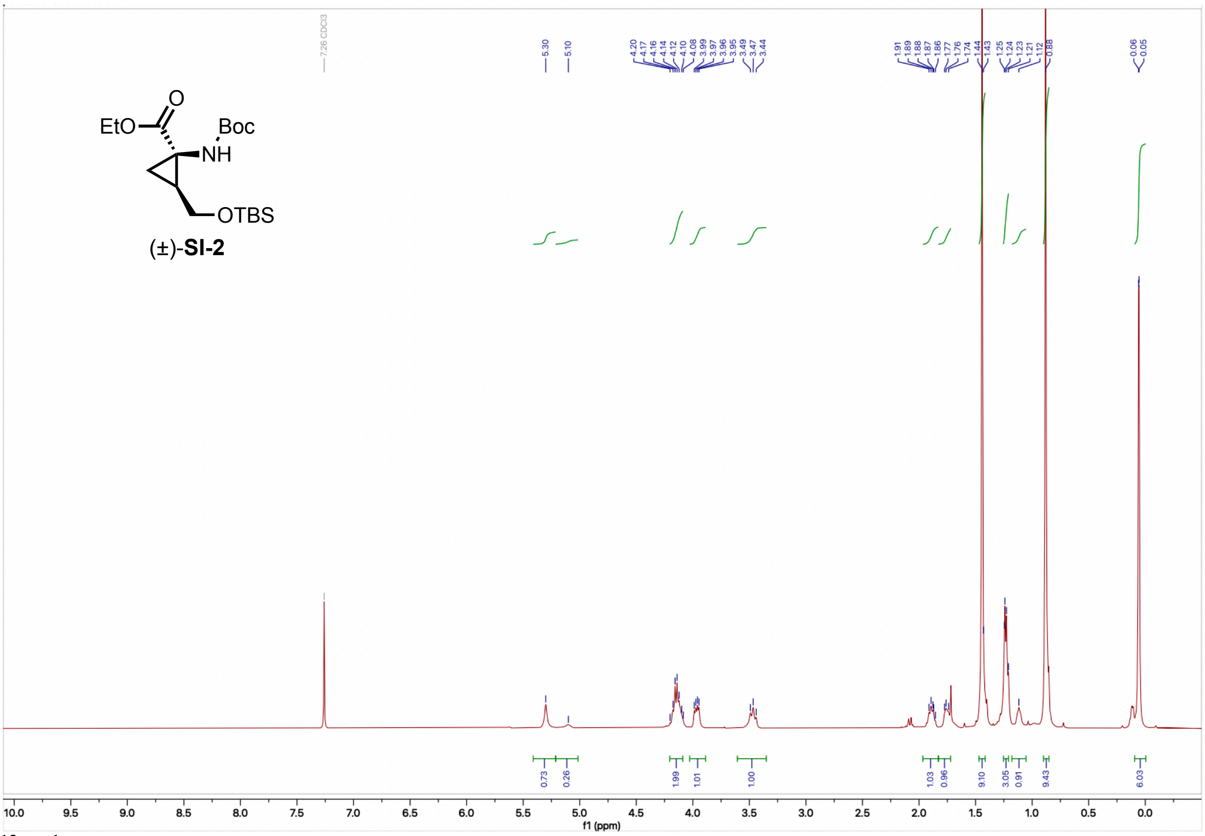

^{13}C{^{1}H} NMR (101 MHz, CDCl$_3$):

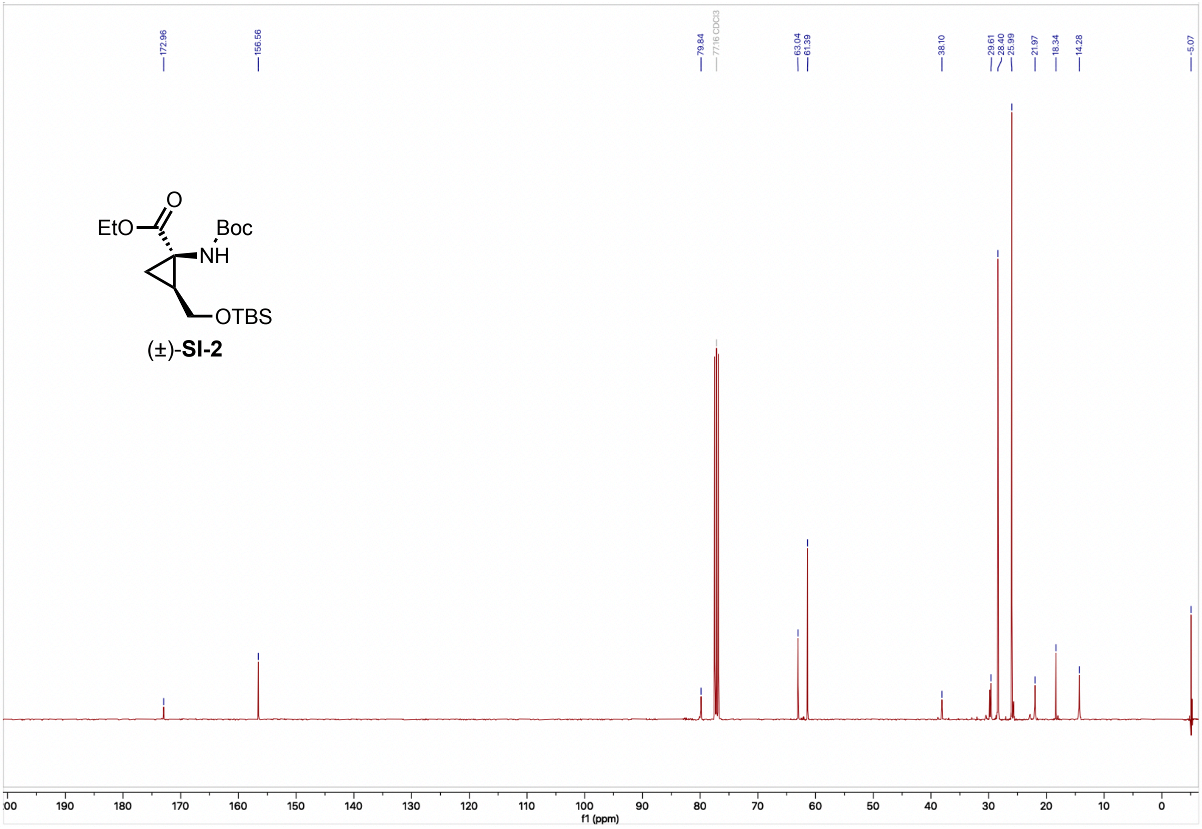

Supplement: Supplementary file 1 — ol5c01341_si_001.pdf [file ol5c01341_si_001.pdf]
